# Macrophage-Centric Phenotypic Screening Identifies Tetrazolone-Based HDAC6 Inhibitors That Reprogram the Tumor Immune Microenvironment and Improve Immune Checkpoint Blockade

**DOI:** 10.1021/acs.jmedchem.5c02453

**Published:** 2026-05-29

**Authors:** Nithya Gajendran, Manasa Suresh, Sebastian J. Marquez R., Sruthi Mohan, Tim Ponsot, David Quiceno-Torres, Mario A. Noboa, Bryan T. Weselman, Xintang Li, Marie Durr, Zora Novakova, Mike Schutkowski, Matias Hepp, Satish Noonepalle, Cyril Barinka, Duncan J. Wardrop, Alejandro Villagra

**Affiliations:** † 8368 Georgetown University, Washington, District of Columbia 20057, United States; ‡ 14681 University of Illinois Chicago, Chicago, Illinois 60607, United States; § Laboratory of Structural Biology, Institute of Biotechnology of the Czech Academy of Sciences, BIOCEV, Prumyslova 595, 252 50 Vestec, Czech Republic; ∥ Charles Tanford Protein Center, Department of Enzymology, Institute of Biochemistry and Biotechnology, 9176 Martin-Luther-University of Halle-Wittenberg, 06120 Halle (Saale), Germany; ⊥ Laboratorio de Investigación en Ciencias Biomédicas, Departamento de Ciencias Básicas y Morfología, Facultad de Medicina, Universidad Católica de la Santísima Concepción, 2850 Concepción, Chile

## Abstract

Tumor-associated macrophages (TAMs) play a pivotal role in shaping the tumor microenvironment (TME) and influencing the outcomes of immunotherapy. However, most drug screening strategies emphasize tumor cell cytotoxicity and neglect immune effector modulation. Here, we describe a macrophage-centric phenotypic screening platform to identify selective HDAC6 inhibitors that reprogram TAMs toward an antitumor phenotype. Building on the HDAC6 inhibitor SS-208, we synthesized a novel class of tetrazolone-based compounds with potent selectivity and minimal cytotoxicity. Among these, SM-06-09 emerged as a lead candidate, showing subnanomolar HDAC6 inhibition, enhanced macrophage phagocytosis, antigen presentation, and T-cell activation in vitro. In a syngeneic melanoma model, SM-06-09 suppressed tumor growth and promoted M1-like TAM polarization. Combination with anti-PD-1 therapy further enhanced immune infiltration, increased effector memory and central memory T-cells, and improved antitumor efficacy. This study establishes a functional screening framework for identifying immunomodulatory compounds and supports the clinical potential of macrophage-targeted HDAC6 inhibitors as adjuncts to immune checkpoint blockade.

## Graphical abstract

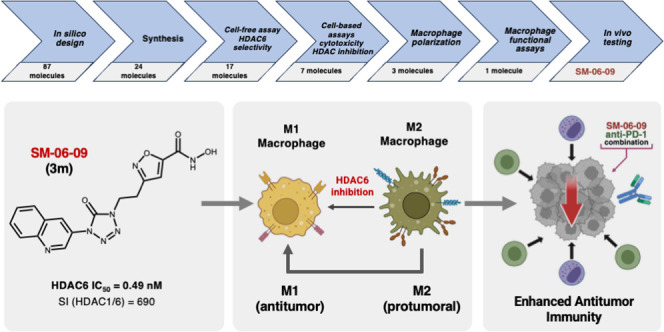

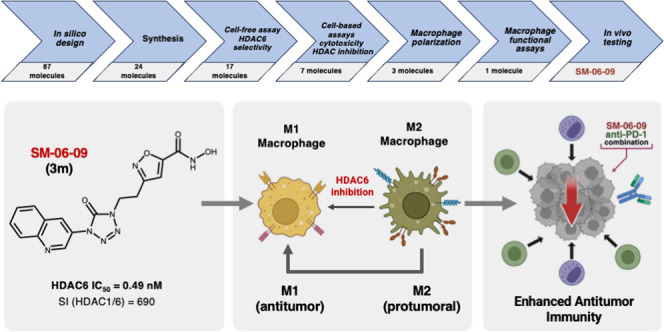

## Introduction

Besides cancer cells, the tumor microenvironment (TME) comprises multiple supportive cell types that create a niche conducive to the initiation and progression of cancer. Among these, tumor-associated macrophages (TAMs) play a central role by interacting with cancer cells and contributing to both local tumor growth and metastatic dissemination, ultimately correlating with poor patient outcomes. Most TAMs exhibit an anti-inflammatory, M2-like phenotype and secrete growth-promoting and angiogenic factors that support tumor progression and resistance to immunotherapy.^1,2^ However, the macrophage phenotype is highly plastic and largely influenced by cytokine cues within the TME, allowing for dynamic switching between protumoral M2-like, antitumoral M1-like, or hybrid states. Consequently, strategies aimed at reprogramming TAMs toward a M1-like phenotype have garnered significant attention as a means to enhance antitumor immunity while minimizing off-target effects and systemic toxicity.^3–5^

Current anticancer drug screening pipelines utilize patient-derived tumor samples or established cell lines, with a primary focus on the cytotoxic effects of therapeutics on cancer cells.^6,7^ While this method allows for a stringent cancer-centered cytotoxic pipeline, the effects of the screened drugs on immune cells are rarely examined.^6,7^ Given the significant role of TAMs in tumor growth and therapeutic outcomes,^8^ it is essential to study the effects of such anticancer candidate drugs on macrophage viability, phenotype, and function.

Histone deacetylases (HDACs) are known to regulate the acetylation of both histone and nonhistone proteins, silencing tumor suppressor genes and activating signaling pathways that drive carcinogenesis.^9,10^ The role of HDACs in cell proliferation and survival has been extensively studied.^11–13^ However, their involvement in regulating immune-related pathways is not entirely understood.^14^ Pan-HDAC inhibitors (pan-HDACis) have been used as anticancer agents in clinical settings. Despite the initial enthusiasm, pan-HDACis have shown limited clinical success due to dose-limiting toxicities, making long-term therapy impractical.^15^ To address this limitation, our group has developed HDAC6 inhibitors (HDAC6is) that are both highly potent and exhibit minimal cytotoxicity. Aberrant HDAC6 expression has been observed in various malignancies, including breast cancer, neuroblastoma, and melanoma.^4,16,17^ Among the 11 HDAC family members, HDAC6 is particularly notable for its ability to deacetylate a broad range of substrates and modulate immune synapse formation within the TME. Pan-cancer scoring of tumor-specific HDAC6 expression across 32 cancers supports the therapeutic potential of targeting HDAC6.^18^ We have demonstrated that HDAC6 inhibition reduces the prevalence of M2-like macrophages and promotes a shift toward a pro-inflammatory M1 phenotype, characterized by increased production of inflammatory cytokines that contribute to the metabolic reprogramming of cancer cells.^19,20^ More broadly, HDAC6is enhance antitumor immune responses,^21^ improve anti-PD-1 immune checkpoint blockade (ICB),^19,20^ and prevent tumor recurrence following radiotherapy.^22^ Combining HDAC6is with anti-CD47 antibodies has been shown to potentiate antitumor activity through TAM reprogramming and activation of natural killer (NK) cells.^5,23^ In a recent study, the novel HDAC6i SS-208 (AVS100, currently in Phase I clinical trials), when combined with anti-PD-1 ICB, resulted in complete tumor remission in murine melanoma models.^20^ Despite these advances, systemic screening of HDAC6is for their ability to reprogram macrophages toward an antitumoral phenotype remains an underexplored avenue. While the large-scale screening models currently used for HDACis have optimized high-throughput biochemical and cellular assays,^24,25^ they do not address the effect on immune cells, specifically macrophages.

The canonical pharmacophore for zinc-dependent HDAC inhibitors comprises three elements: a zinc-binding group (ZBG), typically a hydroxamic acid that coordinates the catalytic Zn^2+^ ion in a bidentate fashion; a hydrophobic linker that threads through the substrate access tunnel; and a surface-recognition cap group that engages the rim of the active site (Figure 1).^26,27^ Among the determinants of HDAC6 selectivity, interaction between the cap and the L1-loop pocket, formed by residues H463, P464, F583, and L712, has been identified as particularly consequential,^28^ alongside the aromatic crevice defined by F583 and F643, which accommodates heterocyclic linker elements.^29,30^ Structurally, SS-208 (**1**) comprises an isoxazole-3-hydroxamate ZBG, a four-atom amide-containing linker, and a 3,4-dichlorophenyl cap.^28^ The ultrahigh-resolution (1.15 Å) crystal structure of *Danio rerio* HDAC6 catalytic domain 2 (drHDAC6-CD2) with SS-208 (PDB: 6R0K)^31^ confirmed bidentate Zn^2+^ coordination by the hydroxamate, a hydrogen bond between the amide carbonyl and S531 at the L1/L2 loop interface, and engagement of the dichlorophenyl cap with the solvent-exposed L1-loop pocket. SS-208 exhibits an IC_50_ of 11.6 nM against HDAC6 with ∼120-fold selectivity over HDAC1 ^20,28^ Critically, replacement of the amide linker with ether or alkyl groups, as in des-amide SS-208 (**2**), resulted in pronounced loss of HDAC6 potency (IC_50_ = 1980 nM), supporting a functional role for the amide in maintaining key polar interactions within the binding channel.^28^

**Figure 1:**
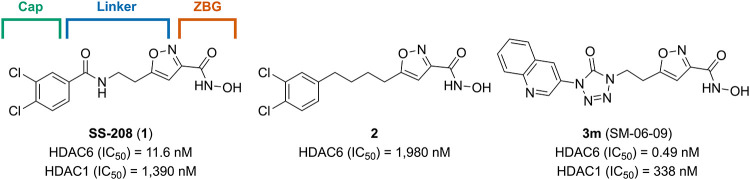
Design of tetrazolone-based HDAC6 inhibitors. SS-208 (**1**) (AVS-100, currently in Phase 1 clinical trials) comprises an isoxazole-3-hydroxamate zinc-binding group (ZBG), a four-atom amide-containing linker, and a 3,4-dichlorophenyl cap group (IC_50_ = 11.6 nM, HDAC6). Replacement of the amide linker with an alkyl chain in des-amide analog **2**, resulted in a >160-fold loss of potency (IC_50_ = 1980 nM), establishing the requirement for a hydrogen-bond-competent linker. Compound **3m** (SM-06-09), the most potent analog in the tetrazolone series, incorporates a 1,4-disubstituted tetrazolone as a potentially more metabolically stable amide bioisostere, retaining a carbonyl hydrogen-bond acceptor while introducing partial aromatic character and conformational restriction (IC_50_ = 0.49 nM, HDAC6; SI = 690 over HDAC1).

Despite its importance for binding, the amide linker presents a recognized metabolic liability through proteolytic and microsomal hydrolysis.^32,33^ To address this limitation while preserving the spatial and electronic features necessary for HDAC6 binding, we considered 1,4-disubstituted 1,4-dihydro-5*H*-tetrazol-5-ones (tetrazolones) as bioisosteric replacements for the amide of SS-208. Although most widely exploited as carboxylic acid bioisosteres,^34–36^ tetrazolones possess properties that make them well-suited as amide surrogates: a carbonyl group with hydrogen-bond acceptor capacity,^37^ an electron-deficient heterocyclic ring with partial aromatic character,^38^ and intrinsic metabolic robustness.^39^ While a wide range of heterocyclic linkers have been employed in HDAC6i design,^40^ to our knowledge, tetrazolones have not previously been investigated as linker motifs in HDAC inhibitor design.

Herein, we describe the structure–activity relationships of a focused library of tetrazolone-based hydroxamic acid HDAC6 inhibitors (**3a**–**3q**) and the identification of compound **3m** (SM-06-09) as a subnanomolar HDAC6 inhibitor (IC_50_ = 0.49 nM) with enhanced isoform selectivity (SI = 690 over HDAC1). To evaluate these compounds beyond conventional biochemical and cytotoxicity end points, we established a screening platform to assess the impact of HDAC6is on macrophage phenotype and function. Of the 17 compounds screened, nine were advanced to comprehensive macrophage modulation studies. The lead compound **3m** demonstrated the highest potency by significantly reducing M2-like polarization and enhancing the M1-like macrophage functional activity both in vitro and in the SM1 murine melanoma model. Notably, combining **3m** with anti-PD-1 antibodies further augmented antitumor efficacy, and transcriptomic and proteomic profiling provided mechanistic insight into the anticancer properties of this lead compound.

## Results

### Design Rationale

The design of compounds **3a**–**3q** was guided by the cocrystal structure of SS-208 bound to drHDAC6-CD2 (PDB: 6R0K),^28^ which reveals the spatial and polar requirements for productive binding. The amide carbonyl of SS-208 forms a hydrogen bond with S531 (N···O distance ≈ 3.3 Å), a residue at the L1/L2 loop interface whose engagement is associated with HDAC6-selective inhibition.^41–43^ Ablation of this polar contact through ether or alkyl replacement abolished potency, confirming that a hydrogen-bond-competent functional group at this position is essential.

The tetrazolone ring was selected as a bioisosteric linker replacement on the basis of several complementary properties. Crystallographic and computational analyses of substituted tetrazolones have established that the ring possesses partial aromatic character, arising from electron delocalization within the N–N=N–N–C­(=O) framework (Figure 2).^38^ The carbonyl group retains substantial hydrogen-bond acceptor capacity (C=O bond lengths of 1.22–1.23 Å),^38,44,45^ and may therefore preserve the S531 interaction critical to the parent series. The planarity of the five-membered heterocycle imposes conformational restriction on the linker, potentially improving shape complementarity within the narrow substrate tunnel. The electron-deficient aromatic surface may additionally engage in π-stacking interactions with the F583–F643 aromatic crevice,^40,46^ a feature absent from the nonaromatic amide linker of SS-208 (Figure 2). Finally, Duncton and co-workers have demonstrated that tetrazolones confer improved metabolic stability relative to the parent functional groups in other medicinal chemistry contexts.^35,36^ The regioselective N-4 alkylation of tetrazolones, facilitated by oxygen-mediated stabilization of electron density at the 4-position,^45,47^ provides straightforward synthetic access to 1,4-disubstituted analogues. The general pharmacophore of the target compounds thus retains the isoxazole-3-hydroxamic acid ZBG of SS-208, which favors bidentate Zn^2+^ coordination and hydrogen bonding to the conserved active-site residues H573, H574, and Y745, while replacing the four-atom amide linker with a 1,4-disubstituted tetrazolone **3** (Figure 2). The N-1 nitrogen of the tetrazolone is connected to the isoxazole-bearing alkyl chain, and the N-4 nitrogen bears the aryl cap group, positioning the tetrazolone carbonyl in a region analogous to that occupied by the amide C=O of SS-208. Three structural axes were systematically varied: (i) the methylene chain length connecting the isoxazole to the tetrazolone, (ii) the identity and substitution pattern of the aryl cap, and (iii) the zinc-binding group heterocycle.

**Figure 2:**
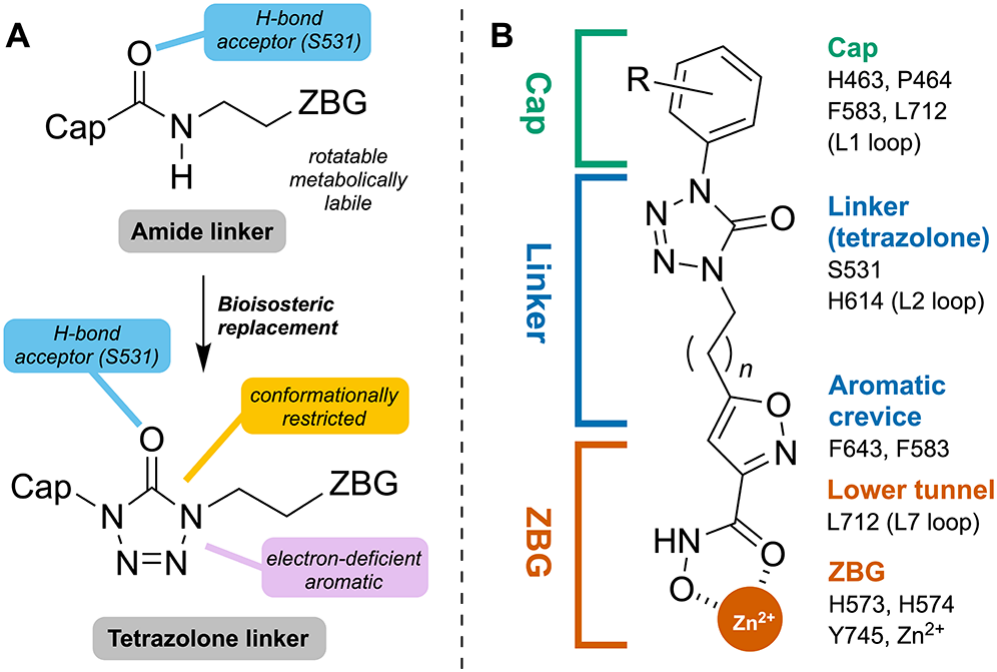
Design rationale for tetrazolone-based HDAC6 inhibitors. (A) Bioisosteric replacement of the amide linker in SS-208 (**1**) with a 1,4-disubstituted tetrazolone. Both linkers present a carbonyl hydrogen-bond acceptor positioned to engage gatekeeper residue S531 at the L1/L2 loop interface. The tetrazolone additionally introduces conformational restriction through ring planarity, reducing the rotatable bond count relative to the acyclic amide and thereby lowering the entropic penalty associated with adopting the bound conformation. The electron-deficient, partially aromatic character of the nitrogen-rich heterocyclic framework may further complement the narrow, preorganized F583–F643 aromatic crevice of HDAC6, for which favorable desolvation entropy has been identified as a key determinant of isoform selectivity. The amide linker, by contrast, is freely rotatable and metabolically labile. (B) General pharmacophore of target compounds **3a**–**3q** mapped onto the HDAC6 catalytic tunnel. Color-coded brackets denote the three pharmacophore zones: the aryl cap (green), which occupies the L1-loop pocket defined by H463, P464, F583, and L712; the tetrazolone linker (blue), whose carbonyl engages S531 and whose ring nitrogen is proximal to H614 in the L2 loop; and the isoxazole-hydroxamate ZBG (orange), which coordinates the catalytic Zn^2+^ ion and forms hydrogen bonds with H573, H574, and Y745. The isoxazole ring resides within the aromatic crevice formed by F643 and F583, and the lower tunnel is lined by L712 of the L7 loop. The three structural axes varied across the series, methylene chain (*n*), aryl cap identity (R), and ZBG heterocycle, are indicated.

To prioritize candidates from this design space for synthesis, a virtual library of 87 tetrazolone analogues was enumerated and subjected to structure-based filtering. The drHDAC6-SS-208 cocrystal structure (PDB: 6R0K) was selected as the docking receptor because it provides the catalytic domain conformation preorganized for this inhibitor chemotype at high resolution (1.15 Å); the available human HDAC6 CD2 structure (PDB: 5EDU; 2.79 Å),^48^ which was cocrystallized with the structurally unrelated inhibitor trichostatin A, affords a less relevant active-site conformation and substantially lower coordinate precision. This choice is further justified by the complete conservation of catalytic channel residues, including the zinc-coordinating motif, Y745, H610, D612, and S531, between the *D. rerio* and *Homo sapiens* orthologues. The library was docked into drHDAC6-CD2 using Schrödinger Glide in SP mode; poses were ranked by GlideScore and inspected for bidentate Zn^2+^ coordination and hydrogen bonding to Y745, H610, and D612. Candidates were further filtered for a GlideScore < −8.5 kcal mol^–1^, a selectivity margin > 1.8 kcal mol^–1^ over HDAC1 (PDB: 4BKX), and physicochemical properties consistent with cytosolic penetration, ultimately yielding 17 priority candidates for synthesis and biological evaluation.

### Chemistry

HDAC inhibitors bearing isoxazole-3-hydroxamate ZBGs (**3a**–**3o**) were prepared from the corresponding aryl tetrazolones **4** via a three-step sequence (Scheme 1). Selective N-4 alkylation with the appropriate alkynyl tosylate afforded **5**, which underwent [2 + 3] cycloaddition with the nitrile oxide generated in situ from ethyl nitroacetate and DABCO to provide isoxazole esters **6** with complete regioselectivity.^49^ Conversion to the target hydroxamic acids **3** was accomplished by treatment of **6** with aqueous hydroxylamine in the presence of catalytic KCN or NaOH.^50^ The thiadiazole variant **3p** was accessed from **4a** through a distinct route. Conjugate addition of **4a** to methyl acrylate furnished **7**, which was converted to **8** upon treatment with hydrazine. Subsequent condensation of hydrazide **8** with ethyl chlorooxoacetate, thionation with Lawesson’s reagent gave methyl ester **9**, which, upon treatment with hydroxylamine, furnished **3p**. The triazole analog **3q** was prepared from **4a** via *N*-alkylation with 1-azido-2-bromoethane, copper-catalyzed [3 + 2] cycloaddition of azide **10** with methyl propiolate, and aminolysis of ester **11** with hydroxylamine. The requisite aryl tetrazolones **4** were synthesized from the corresponding aroyl chlorides by the one-pot method of Duncton.^35^

**Scheme 1:**
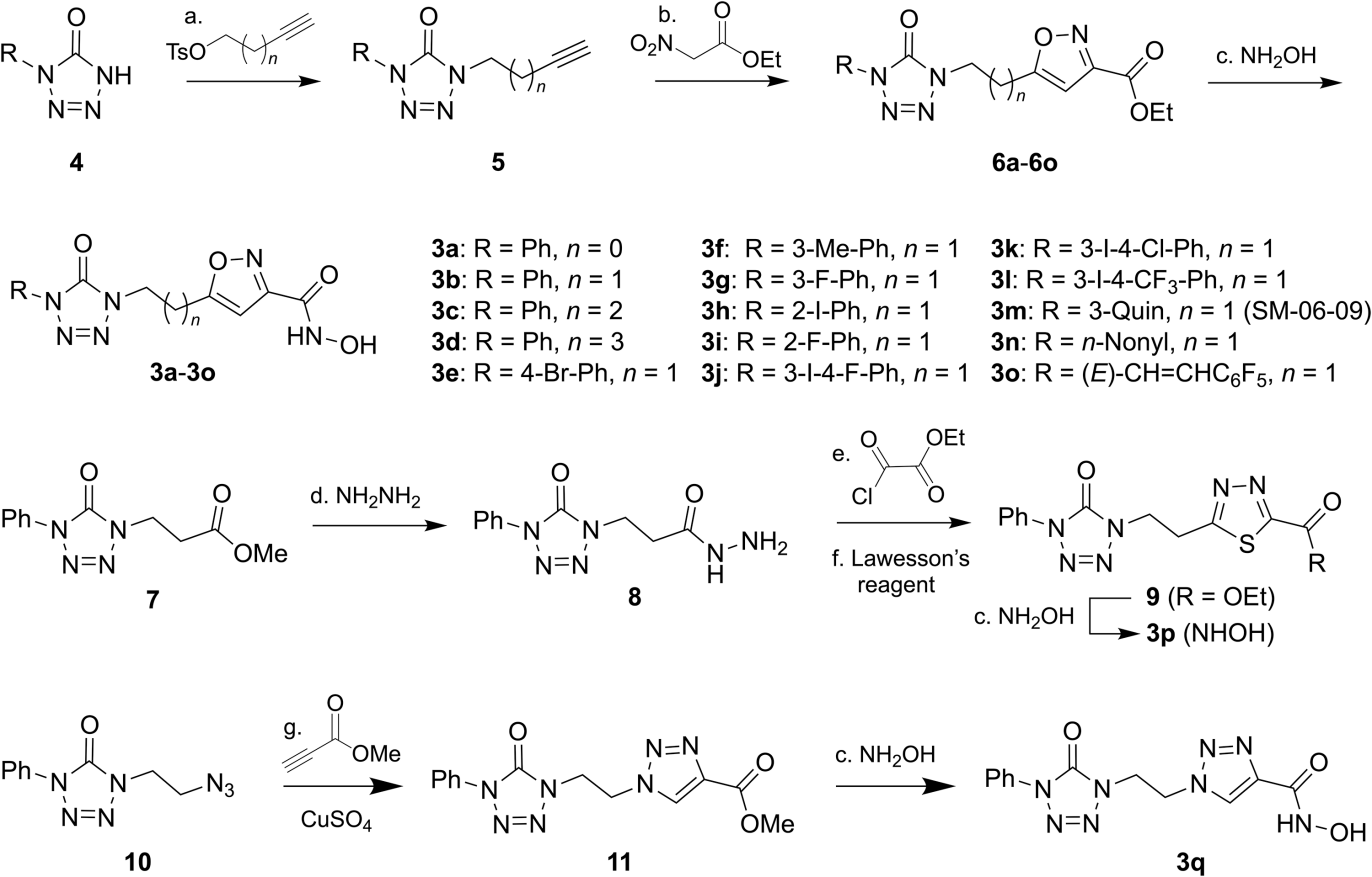
Synthesis of Compounds **3a**–**3q** ^a^

a Reagents and conditions: (a) *N*-alkylating agent, Cs_2_CO_3_, DMF, 60 °C, 12 h, 29–86%; (b) ethyl nitroacetate, DABCO, EtOH, 100 °C, 12 h, 23–69%; (c) NH_2_OH (50 wt % in H_2_O), KCN (or NaOH), THF, MeOH, 25 °C, 43–85%. (d) NH_2_NH_2_·H_2_O, EtOH, 80 °C, 12 h; (e) Ethyl chlorooxoacetate, DCM, pyridine, 25 °C, 12 h; (f) Lawesson’s reagent, PhMe, 75 °C, 3 h, 29% (3 steps); (g) CuSO_4_·5H_2_O, ascorbic acid, *t*-BuOH, H_2_O, 25 °C, 4 h, 59%.

### In Vitro Enzymatic Evaluation and Structure Activity Relationships

The influence of the methylene chain connecting the isoxazole-3-hydroxamate ZBG to the tetrazolone was evaluated through the homologue series **3a**–**3d**, each bearing an unsubstituted phenyl cap (Table 1). Among these, the ethylene-linked analog **3b** (IC_50_ = 4.2 nM) was approximately 7–9-fold more potent than the methylene (**3a**, 29.1 nM), propylene (**3c**, 26.6 nM), and butylene (**3d**, 36.3 nM) homologues. Compound **3b** also exhibited the highest ligand efficiency (LE = 0.50, Schrödinger QikProp)^51,52^ of the entire series, indicating that the two-carbon spacer provides the optimal vector to position the phenyl cap for productive engagement with the HDAC6 surface rim. The decline in LE from **3b** (0.50) to **3c** (0.43) to **3d** (0.41) underscores that additional methylene units dilute binding quality without conferring productive interactions. All subsequent SAR comparisons were therefore conducted within the ethylene-linked subseries.

**Table 1:**
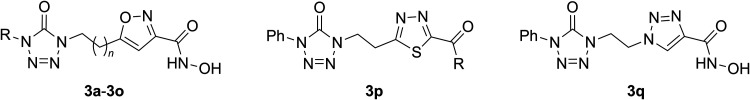
Structure and HDAC6 Inhibitory Activity of Tetrazolones **3a**–**3q** and Reference Compound SS-208 (**1**)

Cmpd	R	*n*	HDAC6 IC_50_ (nM)^a^	pIC_50_	HAC^b^	QPlogP_o/w_ ^c^ ^,^ ^d^	LE^c^ ^,^ ^e^	LipE^c^ ^,^ ^f^
**3a**	Ph	0	29.1 ± 1.1	7.54	22	–0.62	0.47	8.16
**3b**	Ph	1	4.2 ± 1.6	8.38	23	–0.13	0.50	8.51
**3c**	Ph	2	26.6 ± 1.8	7.58	24	0.1	0.43	7.48
**3d**	Ph	3	36.3 ± 3.4	7.44	25	0.49	0.41	6.95
**3e**	4-Br-Ph	1	2.2 ± 1.5	8.66	24	0.4	0.49	8.26
**3f**	3-Me-Ph	1	2.8 ± 2.1	8.55	24	0.14	0.49	8.41
**3g**	3-F-Ph	1	3.5 ± 2.1	8.46	24	0.1	0.48	8.36
**3h**	2-I-Ph	1	194.7 ± 68.0	6.71	24	0.47	0.38	6.24
**3i**	2-F-Ph	1	7.5 ± 3.1	8.12	24	0.06	0.46	8.06
**3j**	4-F-3-I-Ph	1	1.8 ± 0.5	8.74	25	0.68	0.48	8.06
**3k**	4-Cl-3-I-Ph	1	1.3 ± 0.3	8.89	25	0.87	0.49	8.02
**3l**	3-I-4-CF_3_-Ph	1	3.1 ± 0.7	8.51	28	1.33	0.42	7.18
**3m**	3-Quin	1	0.49 ± 0.01	9.31	27	0.08	0.47	9.23
**3n**	*n*-Nonyl	1	27.0 ± 16.3	7.57	26	1.22	0.4	6.35
**3o**	(*E*)-CH=CHC_6_F_5_	1	4.7 ± 2.6	8.33	30	1.27	0.38	7.06
**3p**	Ph	—	37.0 ± 3.4	7.43	23	–0.15	0.44	7.58
**3q**	Ph	—	198.9 ± 139.8	6.70	23	–0.55	0.40	7.25
SS-208^g^	3,4-Cl_2_-Ph	—	11.6 ± 2.1	7.92	22	2.07	0.49	5.85

a IC_50_ values are expressed as mean ± SD of three independent experiments, each obtained from curve-fitting of a 10-point enzymatic assay (30 μM top concentration, 3-fold serial dilution) against HDAC6. pIC_50_ = −log_10_[IC_50_ (M)].

b HAC (heavy atom count).

c ADME properties predicted by QikProp.

d QPlogP_o/w_: predicted octanol/water partition coefficient (neutral species).

e LE (ligand efficiency) defined as 1.37 × pIC_50_/HAC; LE > 0.30 generally acceptable; > 0.40 strong.

f LipE (lipophilic efficiency) defined as pIC_50_ - QPlogP_o/w_; LipE > 5 acceptable; > 6 strong.

g IC_50_ data extracted taken from Reference 28.

With the ethylene linker established as optimal, the effects of aryl ring substitution on HDAC6 potency were examined. Introduction of a *para*-bromine (**3e**, IC_50_ = 2.2 nM) provided a modest 1.9-fold improvement over the unsubstituted parent **3b**, consistent with additional van der Waals contacts in the solvent-exposed region of the rim. In contrast, *meta*-substitution with either methyl (**3f**, 2.8 nM) or fluorine (**3g**, 3.5 nM) was essentially neutral. These results indicate that the HDAC6 surface rim tolerates modest steric elaboration at the *meta* and *para* positions without significant perturbation of potency. Substitution at the *ortho* position proved far more consequential. The *ortho*-fluoro analog **3i** (IC_50_ = 7.5 nM) suffered a 1.8-fold loss relative to **3b**, while the *ortho-*iodo compound **3h** (IC_50_ = 194.7 nM) sustained a 46-fold potency reduction-the most dramatic single-substituent effect in the series. The magnitude of this loss for **3h** is consistent with a steric clash between the bulky iodine at the *ortho* position and the tetrazolone plane, which may force rotation of the aryl cap and preclude productive engagement with the L1-loop surface rim. The smaller *ortho*-fluorine in **3i** produced a qualitatively similar but attenuated effect, reflecting a steric penalty on cap positioning without the complete binding mode disruption inferred for the larger iodine.

In light of the detrimental effect of *ortho-*iodine placement in **3h**, the behavior of the 3,4-disubstituted iodophenyl series (**3j**, **3k**, **3l**) was striking. In these compounds, the iodine occupies the position *meta* to the tetrazolone nitrogen rather than the detrimental *ortho* position of **3h**, and a second halogen or pseudohalogen (F, Cl, or CF_3_) is appended at the *para* position. This regiochemical shift proved transformative: all three compounds achieved sub-5 nM potency, with **3k** (4-Cl-3-I, IC_50_ = 1.3 nM), **3j** (4-F-3-I, 1.8 nM), and **3l** (3-I-4-CF_3_, 3.0 nM) ranking as the second, third, and fourth most potent members of the series. Within this subseries, **3k** (4-Cl) exhibited the highest LE (0.49), while the trifluoromethyl analog **3l** suffered in both LE (0.41) and lipophilic efficiency (LipE = 7.18 vs 8.02 for **3k**),^53^ reflecting the molecular weight and lipophilicity cost of the CF_3_ group. Although **3l** retains potent HDAC6 inhibition, the diminished efficiency metrics suggest limited headroom for further optimization. The contrast between the 46-fold potency loss for *ortho*-iodo **3h** and the sub-2 nM potency of *meta*-iodo **3k** underscores the critical dependence of cap group activity on regiochemistry rather than halogen identity per se.

Replacement of the monocyclic phenyl cap with a 3-quinolinyl group yielded compound **3m** (SM-06-09), which emerged as the most potent inhibitor in the series with a subnanomolar IC_50_ of 0.49 nM-a 2.7-fold improvement over the next best compound (**3k**, 1.3 nM) and a 10-fold enhancement relative to the parent phenyl **3b**. The design of **3m** drew inspiration from Lee and co-workers, who demonstrated that quinolinylmethyl-substituted benzohydroxamic acids can achieve extraordinary HDAC6 selectivity (IC_50_ = 0.29 nM; 4,000–43,000-fold over other isoforms).^54^ The fused bicyclic framework of the quinolinyl cap presents an expanded van der Waals surface for L1-loop shape complementarity, while the endocyclic nitrogen provides an additional hydrogen-bond acceptor.^46,55^

Compound **3m** achieved the highest lipophilic efficiency (LipE = 9.23) of any analog tested, reflecting the favorable combination of subnanomolar potency with the low computed lipophilicity (QPlogP_o/w_ = 0.08, Schrödinger QikProp) of the quinoline system. This value is among the highest reported for any HDAC6 inhibitor and is consistent with on-target activity rather than nonspecific hydrophobic binding. Importantly, **3m** achieves its potency without sacrificing ligand efficiency (LE = 0.47, comparable to the best-in-class values for **3b** and **3k**), demonstrating that the additional ring nitrogen atom contributes productively to binding rather than merely increasing molecular surface area.

To probe the requirement for aromatic character in the cap, two nonstandard variants were evaluated. The *n*-nonyl chain of **3n** (IC_50_ = 27.0 nM) resulted in a 6.5-fold potency loss relative to the phenyl baseline **3b**, accompanied by markedly reduced LE (0.40) and LipE (6.35). These data confirm that aromatic π-character in the cap is important for productive engagement with the HDAC6 surface rim. The (*E*)-pentafluorostyryl cap of **3o** (IC_50_ = 4.7 nM) was more potent than **3n**, although its LE (0.37) was the lowest of the series, suggesting that potency is driven by extensive surface contact rather than efficient binding. The perfluorinated aromatic ring was included to probe potential second-sphere interactions with H614, as Olaoye and co-workers have demonstrated crystallographically that a perfluorinated cap group can engage this residue in drHDAC6.^56^ However, the unfavorable LE argues against this scaffold as a starting point for further optimization.

The critical role of the isoxazole-3-hydroxamate ZBG was demonstrated through two heterocyclic replacements. The 1,2,4-thiadiazole analog **3p** (IC_50_ = 37.0 nM) and 1,2,3-triazole analog **3q** (IC_50_ = 198.9 nM) suffered 9- and 47-fold potency losses, respectively, relative to the matched isoxazole **3b**. The triazole **3q** additionally exhibited a Verber-rule violation (PSA = 155 Å^2^; cutoff < 140 Å^2^), consistent with compromised physicochemical properties. These results confirm that the isoxazole is not merely a scaffold for hydroxamate presentation but participates directly in the Zn^2+^ chelation geometry and hydrogen-bonding network required for high-affinity HDAC6 binding.

Across the series, ligand efficiency and lipophilic efficiency provided substantially better discrimination of experimental potency than did computational docking scores. LE values ranged from 0.38 (**3o**) to 0.50 (**3b**), with the most potent compounds (**3m**, **3k**, **3e**) consistently achieving LE ≥ 0.47-values that compare favorably with established selective HDAC6 inhibitors such as Tubastatin A (LE = 0.44).^57^ The exceptional LipE of **3m** (9.23) reflects a pharmacological profile consistent with efficient, on-target binding.

### Molecular Modeling Studies

To gain insight into the binding modes of compounds in this series, molecular docking calculations were performed using Glide SP (Schrödinger Release 2024-4)^58,59^ against the CD2 catalytic domain of *Danio rerio* HDAC6 (PDB: 6R0K).^28^ The receptor was prepared using the Protein Preparation Wizard with hydrogen-bond optimization at pH 7.0 and restrained minimization (RMSD convergence = 0.30 Å). Ligands were prepared with LigPrep at pH 7.0 ± 2.0,^60^ generating all possible stereoisomers, ionization states, and metal-binding states. A receptor-based Zn–O/N metal-coordination constraint was applied during grid generation; crystallographic waters were removed prior to docking (for further details, see Supporting Information). The discussion below focuses on the linker homologues **3a**-**3d** (*n* = 1–4) and the lead compound **3m**, whose modeled poses are illustrated in Figure 3 (see also Figure S1, Figure S2).^61^

**Figure 3:**
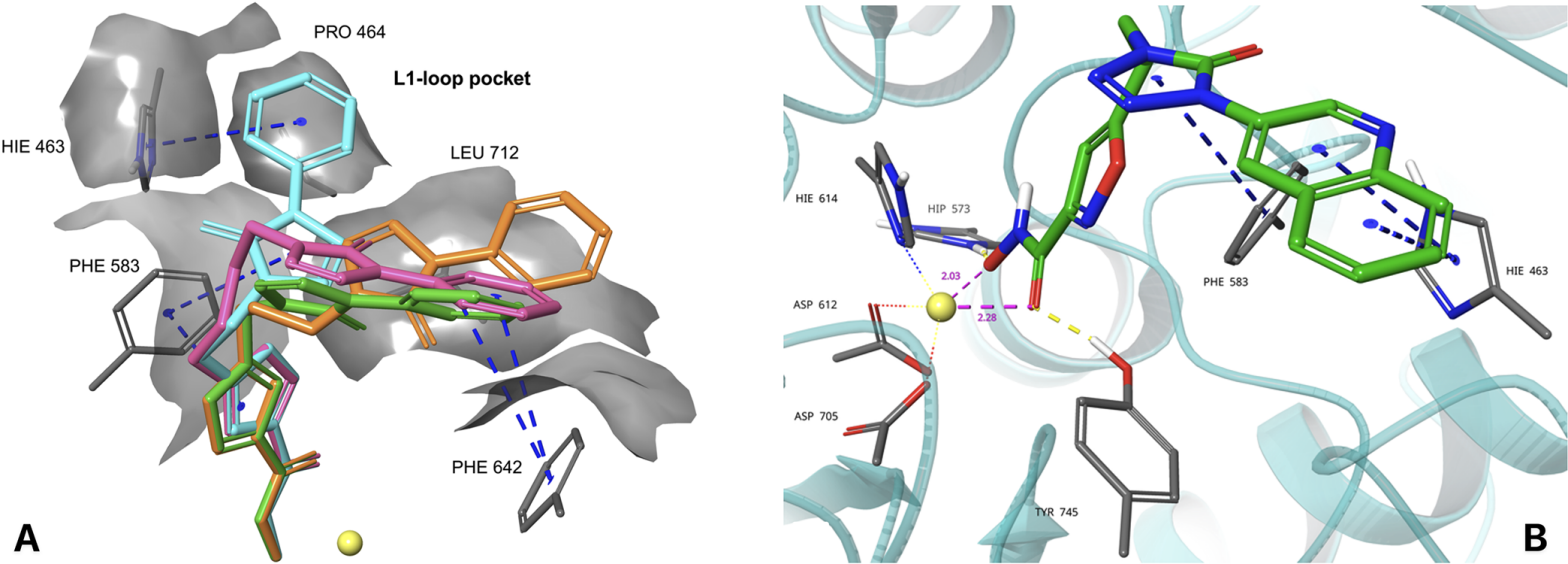
Glide SP docking analysis of tetrazolone-based HDAC6 inhibitors against drHDAC6-CD2 (PDB: 6R0K). (A) Superposition of the modeled binding poses of the phenyl-capped linker homologues **3a**–**3d** (*n* = 1–4) within the catalytic tunnel of drHDAC6-CD2. All four compounds share a common bidentate hydroxamate-Zn^2+^ chelation geometry (not shown for clarity); the view is focused on the divergent trajectories of the phenyl cap group as a function of methylene spacer length. The L1-loop pocket residues (H463, P464, F583, L712) are rendered as a semitransparent molecular surface (gray). Compounds are colored by linker length: **3a** (*n* = 1, green), **3b** (*n* = 2, cyan), **3c** (*n* = 3, orange), and **3d** (*n* = 4, salmon). The two-carbon linker of **3b** (IC_50_ = 4.2 nM) directs the phenyl cap toward H463 (∼5.3 Å) and F583 (∼5.3 Å) at the L1-loop surface, whereas the shorter linker of **3a** redirects the cap deeper into the F583/F642 aromatic crevice (F583 ∼ 3.9 Å), and the longer linkers of **3c** and **3d** each engage only a single aromatic contact (F583 or F642, respectively). Docking was performed using Glide SP (Schrödinger 2024-4) with a Zn–O/N metal-coordination constraint; crystallographic waters were removed prior to grid generation. Distances are measured between ring centroids. (B) Detailed active-site view of the docked pose of the lead compound **3m** (IC_50_ = 0.49 nM; GlideScore = −9.34; *E*_model_ = −104.4). The hydroxamic acid maintains bidentate Zn^2+^ coordination (Zn–O1 = 2.03 Å, Zn–O2 = 2.28 Å), with hydrogen bonds to Y745 (OH···O = 1.53 Å) and H573 (NH···O = 1.63 Å). The tetrazolone carbonyl is positioned within hydrogen-bonding distance of S531 (O···O = 3.3 Å), a gatekeeper residue unique to HDAC6 among the Class IIb zinc-dependent HDACs. The 3-quinolinyl cap engages H463 through dual π–π contacts (centroid distances of 4.9 and 5.5 Å to the pyridine and benzene rings, respectively), consistent with expanded L1-loop engagement relative to the monocyclic phenyl cap of **3b** (panel A). Extending from the cap, the tetrazolone linker and isoxazole ring are sandwiched between the phenyl side chains of F583 and F643, which define the aromatic crevice lining the substrate access channel (isoxazole O19···F583 CE2 = 3.1 Å; methylene C8···F643 CD1 = 3.4 Å). Key hydrogen bonds are shown as black dashed lines; cap-residue π-contacts as gray dashed lines. The protein backbone is rendered as a cartoon (light gray); active-site residues are shown as sticks with carbon atoms colored wheat.

Across all five modeled compounds, the hydroxamic acid adopted a bidentate coordination geometry to the catalytic Zn^2+^ ion, forming a five-membered chelate consistent with the coordination mode observed crystallographically in HDAC6-hydroxamate complexes.^55,62^ In every pose, the hydroxamate carbonyl was positioned within hydrogen-bonding distance of Y745, and the zinc-bound oxyanion appeared to accept a hydrogen bond from H573. The conservation of these interactions across all analogs confirms that the isoxazole-hydroxamic acid warhead maintains the canonical metal-coordination geometry regardless of linker length or cap identity.

Systematic variation of the methylene spacer between the tetrazolone nitrogen and the isoxazole ring (*n* = 1–4, corresponding to **3a**–**3d**) provided an opportunity to examine how linker length influences the predicted positioning of the phenyl cap group relative to the L1-loop pocket (H463, P464, F583, L712) at the rim of the catalytic tunnel (Figure 3A).^63,64^ Among these four analogs, the two-carbon homologue **3b** (IC_50_ = 4.2 nM) exhibited the most favorable modeled pose, with the phenyl cap directed toward H463 (∼5.3 Å) and F583 (∼5.3 Å) at the L1-loop surface, establishing dual π-contacts that are consistent with productive cap-rim engagement. The shorter methylene linker of **3a** (IC_50_ = 29.1 nM) redirected the cap deeper into the F583/F642 aromatic crevice, compressing the F583 contact distance to approximately 3.9 Å and potentially disfavoring the optimal stacking geometry available to **3b**. The longer propylene (**3c**) and butylene (**3d**) homologues each retained only a single aromatic contact (F583 or F642, respectively) and incurred increasing rotatable-bond penalties. These trends suggest that the two-carbon spacer provides the optimal vector for productive cap-rim engagement: shorter linkers misdirect the cap into a suboptimal region of the aromatic crevice, while longer linkers impose conformational entropy costs that attenuate the modeled surface contacts-consistent with the 5- to 6-fold potency advantage of **3b** over its homologues.

The modeled pose of the lead compound **3m** (IC_50_ = 0.49 nM; GlideScore = −9.34; *E*_model_ = −104.4) is depicted in Figure 3B. The hydroxamic acid maintains the canonical bidentate Zn^2+^ coordination (Zn–O_1_ = 2.03 Å, Zn–O_2_ = 2.28 Å), with hydrogen bonds to Y745 (O···O = 1.53 Å) and H573 (N···O = 1.63 Å). Notably, the tetrazolone carbonyl is positioned within hydrogen-bonding distance of S531 (O···O = 3.3 Å), a gatekeeper residue situated at the L1/L2 loop interface that is unique to HDAC6 among the class IIb Zn^2+^-dependent HDACs. Crystallographic evidence confirms that the corresponding residues in class I isozymes are structurally displaced, and inhibitors that form hydrogen bonds with S531 tend to display enhanced HDAC6 selectivity and potency. This interaction is consistent with the design hypothesis that the tetrazolone carbonyl preserves the polar contact maintained by the amide linker of SS-208.^46,63,65^

The 3-quinolinyl cap of **3m** engages H463 through dual contacts (centroid distances of 4.9 and 5.5 Å to the pyridine and benzene rings, respectively) reflecting the expanded π-surface area of the fused bicyclic cap relative to the monocyclic phenyl group of **3b**. That this bifurcated H463 engagement was unique to **3m** among all analogs modeled may reflect the greater propensity of the electron-deficient quinoline ring to participate in π–π stacking interactions. The wider rim of the HDAC6 substrate pocket, relative to other HDAC isozymes, is known to accommodate sterically bulky aryl caps that enhance inhibitor affinity and selectivity through engagement with the lipophilic L1-loop pocket (H463, P464, F583, L712).^46,65,66^ Extending from the cap, the tetrazolone linker and isoxazole ring are sandwiched between the phenyl rings of F583 and F643, which define the aromatic crevice lining the HDAC6 substrate access channel. The isoxazole oxygen and nitrogen make close contacts with F583 (O19···CE2 = 3.1 Å; N18···CE2 = 3.1 Å), while the methylene linker and tetrazolone pack against F643 (C8···CD1 = 3.4 Å; C14···CE2 = 3.5 Å). This dual aromatic engagement positions the inhibitor along the channel axis in a geometry that recapitulates features of the Tubastatin A binding mode in HDAC6 (PDB: 6THV).^67^ More broadly, the introduction of nitrogen-containing heterocyclic caps has been shown to enhance HDAC6 inhibitory activity to varying degrees.^40,68^

Additionally, a putative weak C–H···O hydrogen bond was observed between the quinoline C8–H and the side chain carboxylate oxygen (OD2) D460 (C···O ∼ 3.4 Å; C–H···O angle ∼ 140°).^69,70^ While such interactions contribute modestly to binding affinity, their recurrence in high-affinity HDAC6 inhibitor complexes suggests a stabilizing role.

Examination of the Glide SP Score versus experimental pIC_50_ for all 17 tetrazolone analogs (**3a**–**3q**) revealed poor linear correlation (Pearson *r* = −0.41), which is consistent with recognized limitations of empirical scoring functions for zinc metalloenzymes,^71,72^ where the metal-coordination energy term can dominate the composite score, compressing the dynamic range available for discriminating among analogs that share a common zinc-binding warhead. In contrast, ligand efficiency (LE, Pearson *r* = 0.57, Schrödinger QikProp) and lipophilic efficiency (LipE, Pearson *r* = 0.62) provided substantially better discrimination. These observations suggest that, in this case, efficiency metrics may be more reliable than docking scores for prioritizing compounds within the congeneric hydroxamate series.

While these docking studies provide valuable insight into the binding modes and structure–activity relationships of this inhibitor series, the static nature of rigid-receptor molecular docking does not account for protein flexibility, explicit solvation effects, or entropic contributions to binding. The modeled poses should therefore be regarded as structural hypotheses that guided compound prioritization rather than definitive binding geometries. Future induced-fit docking or molecular dynamics simulations may further refine these models.

### HDAC6 Selectivity Profiling of Compound **3m**

To assess isoform selectivity, **3m** was profiled against a panel of zinc-dependent human HDAC isoforms (HDAC1-11) in cell-free enzymatic assays (Table 2). Compound **3m** inhibited HDAC6 with an IC_50_ of 0.49 ± 0.01 nM and displayed pronounced selectivity over all Class I, IIa, and IV isoforms (SI ≥ 349). Among the Class IIb enzymes, however, **3m** exhibited cross-reactivity with HDAC10 (IC_50_ = 16.9 ± 1.0 nM; SI = 35), an observation consistent with the shared catalytic domain architecture of the two Class IIb family members.

**Table 2:**
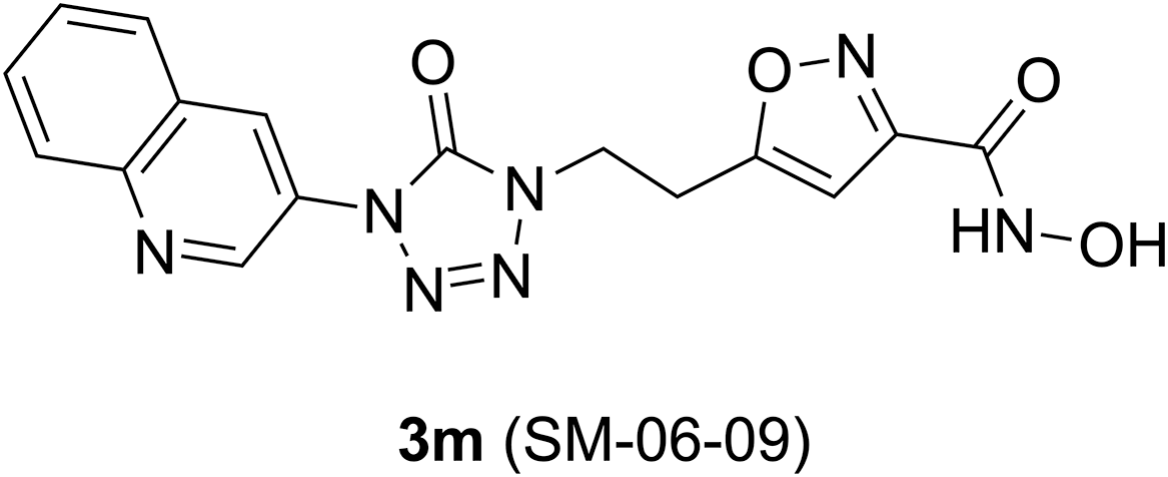
HDAC Profile of Compound **3m** (SM-06-09)

Isoform	Class	IC_50_ (nM)^a^	SI^b^
HDAC6	IIb	0.49 ± 0.01	
HDAC10	IIb	16.9 ± 1.0	35
HDAC2	I	173 ± 68	349
HDAC3	I	258 ± 35	522
HDAC1	I	338 ± 95	690
HDAC8	I	453 ± 118	916
HDAC7	IIa	4,443 ± 1424	8,975
HDAC5	IIa	7,602 ± 1313	15,360
HDAC4	IIa	12,415 ± 2850	25,080
HDAC9	IIa	26,035 ± 9723	52,600
HDAC11	IV	1891 ± 244	3,859

a IC_50_ values are expressed as mean ± SD of two independent experiments. Human HDACs 1–9 and 11 were heterologously expressed in HEK293T cells, affinity-purified by streptactin chromatography, and assayed at 37 °C in HEPES buffer (50 mM HEPES, 140 mM NaCl, 10 mM KCl, pH 7.4, 1 mg/mL BSA, 1 mM TCEP) using Ac-GAK-Ac-AMC (HDAC1–3, 6), Boc-Lys­(TFA)-AMC (HDAC4, 5, 7, 9, 11), or N8-acetylspermidine-fluorescein (HDAC10) as substrates (10 μM each). Fluorescence was quantified by plate reader (λ_EX_/λ_EM_ = 365/440 nm) or RP-HPLC (λ_EX_/λ_EM_ = 492/516 nm) for HDAC10. IC_50_ values were determined by nonlinear regression (GraphPad Prism).

b Selectivity index (SI) defined as IC_50_(isoform)/IC_50_(HDAC6).

The moderate HDAC10 activity of **3m** merits comment. Unlike HDAC6, which deacetylates acetyl-l-lysine substrates, HDAC10 is a polyamine deacetylase with maximal catalytic activity against N8-acetylspermidine, a selectivity dictated by the electrostatic gatekeeper E274 and the steric constriction imposed by the 3_10_ helix bearing the P­(E,A)­CE motif.^73^ Despite these substrate-level differences, the zinc-binding channel and active site contour of HDAC10 share considerable structural homology with HDAC6 CD2, and precedent exists for Class IIb cross-reactivity: Tubastatin A, one of the most widely used HDAC6-selective tool compounds, also displays nanomolar HDAC10 inhibition.^57^ The 35-fold selectivity observed for **3m** indicates that HDAC6 remains the primary target by a substantial margin, while the ancillary HDAC10 activity could prove therapeutically relevant in oncology contexts where dual HDAC6/10 inhibition has been shown to suppress autophagic survival responses.^74^

Against the Class I enzymes, selectivity indices of 349–916 were observed (HDAC2, IC_50_ = 173 nM; HDAC3, 258 nM; HDAC1, 338 nM; HDAC8, 453 nM), consistent with the structural differences between the HDAC6 catalytic domain-notably the wider L1-loop topology and the F583/F643 aromatic crevice and the more constrained Class I active sites. That the highest selectivity within this class was observed for HDAC8 (SI = 916) is consistent with the unique structural features of HDAC8, including its comparatively restricted substrate access tunnel.

The Class IIa isoforms were essentially spared, with IC_50_ values ranging from 4,000 to 26,000 nM (SI ≈ 9,000–53,000 for HDAC7, 5, 4, and 9, respectively), in accord with the attenuated deacetylase activity of these enzymes arising from the conserved tyrosine-to-histidine substitution in the catalytic site. HDAC11, the sole Class IV isoform, was also minimally affected (IC_50_ = 1,891 ± 244 nM; SI ≈ 3,900).

Collectively, these data establish **3m** as a potent and selective HDAC6 inhibitor with a well-defined isoform selectivity hierarchy: HDAC6 ≫ HDAC10 (35-fold) ≫ Class I (349-916-fold) ≫ HDAC11 (∼3,900-fold) ≫ Class IIa (>9,000-fold). The selectivity profile compares favorably with established HDAC6 tool compounds and positions **3m** as a chemical probe suitable for the dissection of HDAC6-mediated biology.

### Screening of Tetrazolones for Ultraspecific HDAC6 Inhibition with Minimum Cytotoxicity

Having identified 17 priority tetrazolone candidates through the computational and enzymatic analyses described above, we next assessed their potency, HDAC6-versus-HDAC1 selectivity, cytotoxicity, and cellular target engagement in the RAW264.7 murine macrophage cell line. These selected compounds were prescreened for their potency and HDAC6 vs HDAC1 selectivity in vitro using purified enzymes (Figure 4A, Figure S3A). After confirming their selectivity toward HDAC6, the compounds were evaluated for inhibition of the total deacetylase activity in the RAW264.7 murine macrophage cell line using the HDAC-Glo assay (Promega) (Figure 4B, Figure S3B). Most compounds exhibited a dose-dependent HDAC inhibition, with IC_50_ values ranging from 625 to 7,500 nM, except for compound **3f**. Notably, **3b** showed ∼50% inhibition at 10 μM, **3l** at 5 μM, and SM-06-09 (**3m**) at 1.25–2.5 μM. At 10 μM, SM-06-09 (**3m**) inhibited nearly 85% of the total HDAC activity (Figure 4B). The established HDAC6 inhibitor, Nexturastat A (NextA), and the pan-HDAC inhibitor, trichostatin A (TSA), were included as positive controls.

**Figure 4:**
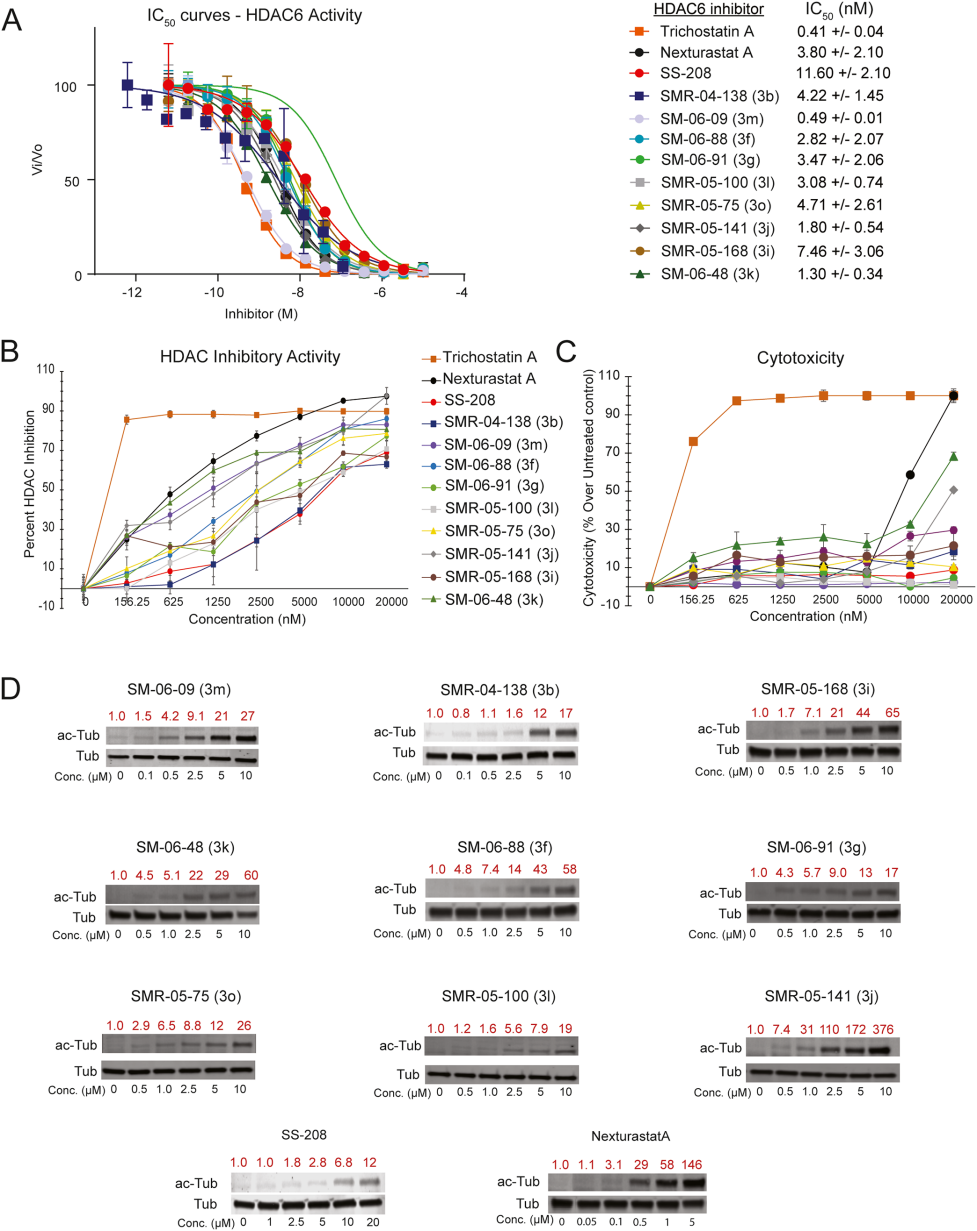
Screening of tetrazolones with selective HDAC6 inhibition activity. (**A**) Full-dose response inhibition curves for compounds with higher than 50% inhibitory activity under screening conditions. Corresponding IC_50_ values were calculated from the inhibition curves using the GraphPad Prism software (GraphPad Software, San Diego, CA, USA). Data are plotted as mean values ± SD from three independent experiments (*n* = 3), (**B**) Determination of deacetylase activity using HDAC-Glo (Promega) analysis in RAW264.7 macrophages at indicated concentrations of HDACis. (**C**) Cytotoxicity assay of HDACis at indicated concentrations using Cytotox (Promega). (**D**) Immunoblot analysis was performed in RAW264.7 macrophages treated with different concentrations of candidate HDAC6 inhibitors for tubulin and acetyl tubulin, and the band intensities were quantified in Image Studio.

To evaluate cytotoxicity, the compounds were tested in RAW264.7 cells using the CellTox Green Cytotoxicity Assay (Promega) (Figure 4C, Figure S3C). All tetrazolones exhibited minimal cytotoxicity (<10%) across the entire concentration range tested (0.15–20 μM). A few compounds (**3k**, **3j**, and **3f**) showed slightly increased cytotoxicity (up to ∼15%), but only at higher concentrations (>5 μM). These findings indicate that tetrazolones are largely nontoxic to RAW264.7 macrophages within the inhibitory concentration range.

Given that HDAC6 specifically deacetylates α-tubulin, its inhibition was further evaluated by measuring acetylated α-tubulin levels. RAW264.7 cells were treated with increasing concentrations of the compounds (0.5–10 μM) for 24 h, followed by quantification of acetylated α-tubulin via Western blot analysis. Nine compounds induced a dose-dependent increase in tubulin acetylation (Figure 4D), indicating effective HDAC6 inhibition, while the remaining seven showed only modest or negligible effects (Figure S3D). Well-characterized HDAC6 inhibitors, including NextA and SS-208 (AVS100), were used as benchmarks. Based on the combined data (high HDAC inhibitory potency in vitro and in cells, and minimal cytotoxicity), **3b** and SM-06-09 (**3m**), two of the best-performing candidates, were further profiled in vitro against a panel of 11 zinc-dependent HDAC isoforms. The data confirmed the excellent selectivity and potency of the inhibitors for human HDAC6 (Figure 5E).

**Figure 5:**
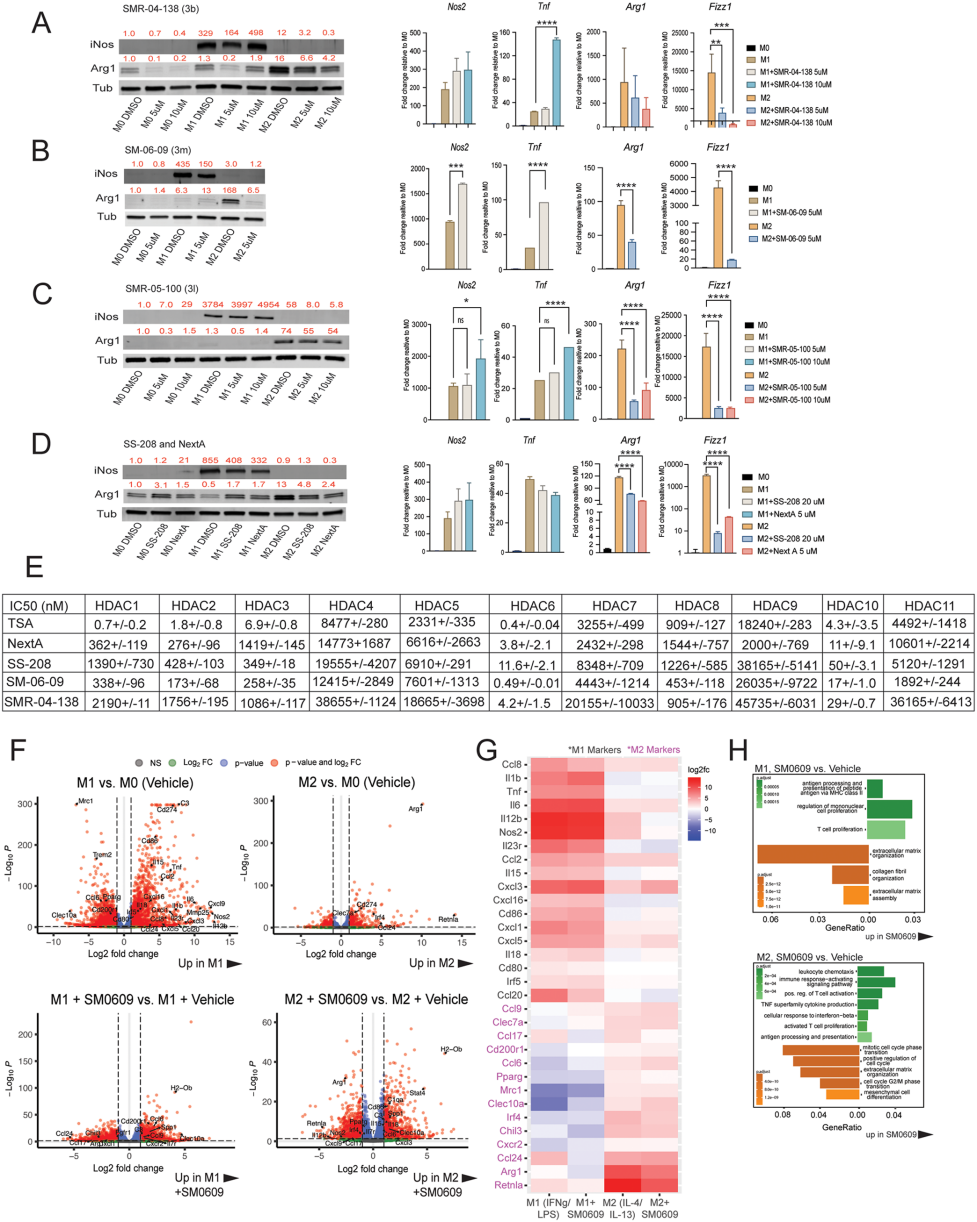
HDAC6 inhibition by tetrazolones increases pro-inflammatory M1 phenotype in bone marrow-derived macrophages. Bone marrow-derived macrophages were treated with selected HDAC6 inhibitors and polarized to M1-like (100 ng/mL of LPS and 50 ng/mL of IFN-γ) and M2-like (20 ng/mL of IL-4 and IL-13 each) phenotypes. Immunoblot and qRT-PCR analyses for (**A**) **3b** (**B**) **3m**, (**C**) **3l** (**D**) **SS-208 and NextA** showing changes in levels of iNOS and Arg1 (in immunoblot) and genes associated with M1 (*iNOS, Tnf-*α,) and M2 (*Arg1and Fizz1*) phenotype of macrophages (in qRT-PCR). Data plotted are mean values ± SD from technical replicates (*n* = 3); **P < 0.01, ***P < 0.001, ****P < 0.0001, one-way ANOVA. (**E**) Summary of IC_50_ values for SM-06-09 and SMR-04-138 along with the controls, TSA, NextA, and SS-208. (**F**) Volcano plots displaying significantly differentially expressed genes (log2FC > 1.0 or ←1.0 and adjusted *p* < 0.05) between unpolarized, untreated BMDMs (M0) and those polarized to M1 or M2, with and without SM-06-09 treatment. (**G**) Heatmap displaying log2FC (from M0) of selected markers of M1 or M2 macrophages. (**H**) Select macrophage-related functional enrichment terms of significantly up- (green) and down- (orange) regulated genes between M1 macrophages with and without SM-06-09 (**3m**) treatment and M2 macrophages with and without SM-06-09 (**3m**) treatment.

Although the tetrazolone derivatives display subnanomolar potency against purified HDAC6, upregulation of acetyl-α-tubulin in RAW264.7 cells was most pronounced at concentrations of 2.5 μM and above (Figure 4D), revealing a substantial offset between biochemical and cellular potency. This discrepancy is consistent with the known contribution of cellular permeability, subcellular compartmentalization of HDAC6, and time-dependent target engagement to the apparent potency of hydroxamic acid–based inhibitors in whole-cell assays and underscores the importance of complementary target-engagement studies to bridge this gap.

### Tetrazolones Influence the Phenotype of Bone Marrow-Derived Macrophages

Based on the above screening, nine compounds, **3m** (SM-06-09), **3b**, **3i**, **3k**, **3f**, **3g**, **3o**, **3l**, and **3j**, were further evaluated for their ability to modulate macrophage phenotype. BMDMs from wild-type C57BL/6 mice were treated overnight with compounds at 5 and/or 10 μM concentrations, followed by a half dose 2 h before polarization into an M1 or M2 phenotype. With BMDMs, we used 5 μM of SM-06-09 (**3m**) as the 10 μM dose reduced their viability. Cells were harvested after 16 h (for qPCR) and 24 h (for Western blot) of polarization to quantify the levels of M1- and M2-like macrophage markers by qPCR and Western blot assays. Treatment with **3b**, **3m** (SM-06-09), and **3l** showed a significant increase in the expression of the M1 marker, *Tnf*, and a decrease in the expression of M2 markers, *Arg1* and *Fizz1* (Figure 5A–C). SS-208 and NextA were used as positive controls for HDAC6is (Figure 5D). Changes in *Nos2* expression, also a marker of M1 macrophages, were only modest with **3b** treatment, and a significant increase was observed with SM-06-09 (**3m**) and **3l** treatments. At the protein level, treatment by **3b** (Figure 5A), SM-06-09 (**3m**) (Figure 5B), and **3l** (Figure 5C) resulted in a significant decline in Arg1 protein. These results confirmed that HDAC6 inhibition decreased the anti-inflammatory M2-like phenotype of macrophages. The levels of iNOS protein either increased or remained the same after treatment with these tetrazolones. Similar, but to a lesser extent, dose-dependent effects on M1 and M2 markers expression were observed in BMDMs treated with lower concentrations of **3b** and SM-06-09 (0.1, 0.5, and 1 μM) (Figure S4). However, at the protein level, SM-06-09 was more effective in reducing Arg1 at lower concentrations when compared to **3b**. To confirm the selectivity at cellular level, we quantified the acetyl-histone 3 (ac-H3) levels in RAW264.7 cells treated with 3b, SM-06-09 (**3m**) and 3l (Figure S4E). No significant changes in ac-H3 levels were observed, including at different concentrations of the lead compound, SM-06-09 (Figure S4F). The remaining six tetrazolones had no significant effect on reducing the M2-like macrophage phenotype, especially at protein levels (Figure S5). To determine the longevity of HDAC6 inhibition by a single dose of SM-06-09 (**3m**) (5 μM), acetyl α-tubulin levels were determined by Western blot at different time points post-treatment in BMDMs. SS-208 (20 μM) and NextA (5 μM) were included as positive controls for comparison. A single dose of SM-06-09 (**3m**) showed sustained inhibition of HDAC6 (with measurable levels of acetyl α-tubulin) for 5 days (120 h) in comparison to around 6 days (144 h) with NextA and 48 h with SS-208 treatment (Figure S6).

Bulk RNA sequencing was performed on BMDMs polarized to M1 and M2, with vehicle or SM-06-09 treatment, to evaluate the effect of this compound on the broad gene expression of macrophages. Macrophages polarized to M1 that were treated with SM-06-09 maintained high pro-inflammatory marker expression, and, in some cases, the proinflammatory markers were even increased (*Il1b*, *Cxcl3*) (Figure 5F,G). Macrophages polarized to M2 that were treated with SM-06-09 had decreased expression of anti-inflammatory markers, such as *Arg1* and *Retnla* (*Fizz1*), as well as an increase in chemokines, including *Cxcl3* and *Cxcl5*, compared to the M2 vehicle-treated group. Notably, *C3*, involved in the complement-driven immune response, was highly differentially expressed in the M2 macrophages treated with SM-06-09 (**3m**) (Figure 5G), indicating an increase in complement-driven inflammatory responses.

Functional enrichment analysis of differentially expressed genes between M1 macrophages treated with SM-06-09 and vehicle controls suggests that HDAC6 inhibition increases gene expression associated with antigen presentation and processing, as well as T-cell and macrophage proliferation, and decreases gene expression involved in extracellular matrix (ECM) and collagen assembly (Figure 5H, Figure S7A, Figure S7C). Enrichment of differentially expressed genes between M2 macrophages treated with SM-06-09 and vehicle controls revealed an increase in genes associated with antigen presentation and processing, as well as T-cell activation and inflammatory pathways such as TNF signaling and the IFN response. HDAC6 inhibition resulted in a decrease in gene expression related to ECM organization, mesenchymal differentiation, and proliferative processes (Figure 5H, Figure S7B, Figure S7D), as previously reported for breast cancer models.^21^

Collectively, these data demonstrate that HDAC6 inhibition by the tetrazolone series shifts the macrophage phenotype from an immunosuppressive M2-like state toward a pro-inflammatory M1-like state, with SM-06-09 (**3m**) exhibiting the most pronounced modulation at lower concentrations relative to **3b** and **3l**. It should be noted, however, that the M1/M2 classification represents a simplification of the macrophage activation spectrum; macrophages are highly plastic and can adopt hybrid phenotypes shaped by microenvironmental cues, a complexity examined at subpopulation-level resolution in the single-cell analyses described below.

### SM-06-09 Enhances M1-like Macrophage Function

Based on its superior effect on macrophage phenotype at relatively low concentrations and its longer-lasting HDAC6 inhibition, we selected SM-06-09 (**3m**) to further evaluate its effects on macrophage functions. Pro-inflammatory M1-like macrophages are better phagocytic antigen-presenting cells than M2-like macrophage.^75^ Also, previous studies from our group have demonstrated that HDAC6 inhibition enhances phagocytic activity.^5,22^ BMDMs isolated from UBC-GFP mice and polarized to the M1 phenotype were cocultured with SM1 murine melanoma cells stained with Cell Trace Violet and pH-rodo dyes. Images captured at 30 min intervals showed a significant increase in phagocytosis by M1 macrophages treated with SM-06-09, which was notably higher than the phagocytosis rate observed with NextA treatment compared to the M1 macrophages (Figure 6A). Phagocytosis rate was calculated as a percentage of GFP macrophages with phagosomes (indicated with white arrow heads) containing fragments of pH-rodo stained SM1 melanoma cells that fluoresce only in the acidic pH of phagosomes, as shown in Figure 6B. A schematic of the assay is shown in Figure 6C.

**Figure 6:**
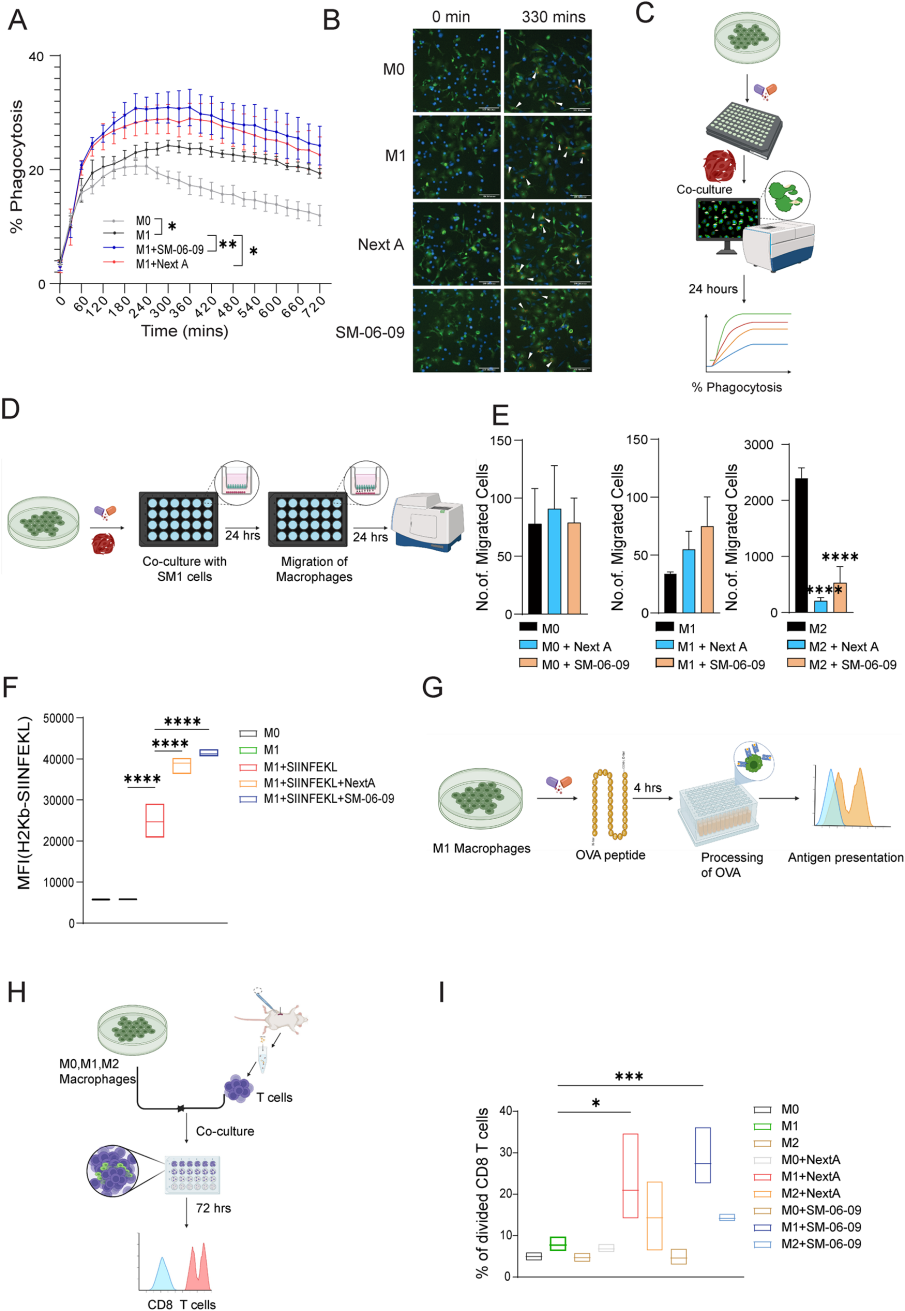
Select tetrazolones that modulate macrophage phenotype with HDAC6 inhibition, enhance phagocytic capacity, antigen presentation, T cell proliferation in M1-like macrophages, and reduce migration of M2 macrophages. (**A**) Percentage of phagocytosis in M1 macrophages treated with Next A and SM-06-09. (**B, C**) Representative images of phagocytosis assay performed using BMDMs from GFP mice (in green) were cocultured with SM1 murine melanoma cells stained with CellTrace Violet (in blue) and pHrodo (in orange in phagosomes when phagocytosed) at a 2:1 ratio. Scale bars represent 100 μm. (**D, E**) The cell numbers of M0, M1, and M2 types of BMDMs from GFP mice migrated to the bottom well. (**F, G**) Bone marrow-derived M1 macrophages were treated with OVA peptide for 4 h in the presence and absence of NextA and SM-06-09. The expression of H2Kb SIINFEKL complex was assessed by flow cytometry. (**H, I**) Murine splenic T cells were cocultured with bone marrow-derived macrophages polarized to M1 and M2 phenotypes, pretreated with NextA and SM-06-09. T-cell proliferation was measured by dividing CD8 T-cells after 72 h. *n* = 4, *P < 0.05, **P < 0.01, ***P < 0.001, ****P < 0.0001, one-way ANOVA.

In addition to tissue-resident macrophages, the infiltration of circulating monocytes that differentiate into macrophages contributes to the TAM population, which mainly exhibits an M2-like phenotype. We have previously shown that treatment with HDAC6 inhibitors reduces the migration of M2-like macrophages toward the tumor cells.^22^ To determine if SM-06-09 (**3m**) affects macrophage migration, GFP-BMDMs were seeded in respective transwell inserts and treated with SM-06-09 (**3m**) before polarization into M1-like and M2-like macrophages (Figure 6D). They were then cocultured with SM1 melanoma cells at the bottom of a 24-well transwell plate. NextA treatment was used as a control. After 24 h of coculture, treatment with SM-06-09 and NextA significantly reduced the number of GFP-expressing M2-like macrophages migrating to the bottom wells. We did not observe a notable difference in migration of unpolarized M0 macrophages treated with SM-06-09 (**3m**) and NextA; however, there was a trend of increasing migration among M1 macrophages treated with HDAC6is (Figure 6E).

To assess the effect of SM-06-09 (**3m**) on antigen presentation, we used the well-established chicken ovalbumin (OVA) SIINFEKL peptide model. Upon exposure of M1 macrophages to the OVA peptide, the peptide undergoes antigen processing and is presented on MHC class I, leading to the formation of peptide-MHC class I complex with the ability to cross-present to T cells. Our study focused on assessing whether M1-polarized macrophages present antigens more efficiently to T cells. M1 macrophages were treated with the OVA peptide for a period ranging from 4 to 24 h. At 4- and 8-h time points, we observed a significant presentation of H2Kb-SIINFEKL, as illustrated in the supplementary figure (Figure S8). Importantly, SM-06-09 (**3m**) significantly increased antigen presentation by M1 macrophages over a 4-h period (Figure 6F,G). This highlights the potential of M1 macrophages to activate T-cells. To prove this, we cocultured murine BMDMs and splenic T-cells. M1-like macrophages treated with NextA and SM-06-09 showed significantly increased division of T-cells (Figure 6H,I). In contrast, naïve and M2-like macrophages did not play a significant role in T-cell activation following pretreatment with the HDAC6 inhibitor. The enhanced MHC class I antigen presentation observed upon SM-06-09 (**3m**) treatment, in conjunction with the pro-inflammatory macrophage phenotype, is expected to create conditions conducive to robust CD8+ T-cell activation. Indeed, activated CD8+ T-cells can produce cytokines that reinforce the M1 phenotype, establishing a positive feedback loop between innate immune reprogramming and adaptive antitumor immunity.^76^ Taken together, these functional data show enhanced phagocytosis, reduced M2-like macrophage migration, increased antigen cross-presentation, and augmented T-cell proliferation-position SM-06-09 (**3m**) as a multifunctional modulator of the macrophage-T-cell axis.

### SM-06-09 Reduces the Tumor Burden in the SM1 Murine Melanoma Model

Given its potent HDAC6 inhibition, negligible cytotoxicity profile, and enhanced ability to modulate macrophage phenotype and function in vitro, SM-06-09 (**3m**) was further investigated in an in vivo study to evaluate its anticancer efficacy. SM1 cells were injected subcutaneously (0.75 × 10^∧^6 cells) into the right flanks of C57BL/6 female mice (6–8 weeks old), and tumor growth was monitored until palpable; then the mice were randomized into different groups. The dosing schedule was based on previous studies, and the treatment continued until the control animals reached a total volume of 2000 mm^3^. SM-06-09 (**3m**) was found to have statistically significant suppression of tumor growth relative to the vehicle control (Figure S9A, Figure S9B). At the end of the study, to understand the impact of SM-06-09 on the TME, we performed immune profiling on the tumor cell suspension. Flow cytometry revealed that SM-06-09 (**3m**) significantly suppressed the expression of M2-like macrophages (Figure S9C). This phenotypic shift suggests that SM-06-09 (**3m**) has a tumor-suppressive effect and also reconditions the TME to support antitumor immunity. The suppression of M2-like macrophages observed upon SM-06-09 treatment is significant in light of the well-established role of M2-polarized TAMs in maintaining a pro-tumoral, immunosuppressive TME through the secretion of growth factors, angiogenic mediators, and immunomodulatory cytokines.^77,78^ By reducing this immunosuppressive population, SM-06-09 (**3m**) may relieve a critical barrier to effective antitumor immunity. These findings are consistent with previous reports on the role of HDAC6 in regulating cytoskeleton dynamics, myeloid cell activation, and cytokine signaling.^79^ This study also highlights the potential of SM-06-09 in combination with immune checkpoint inhibitors, providing a strong rationale for advancing the compound to a translational study.

### SM-06-09 Increases the Efficacy of Anti-PD-1 Therapy in the Syngeneic SM1 Murine Melanoma Model

We previously reported that combining hydroxamate-based HDAC6is, Nexturastat A, and AVS100, with anti-PD-1 therapy, was effective in suppressing tumors in the immunocompetent SM1 murine melanoma model compared to single-arm therapies.^19,20^ Therefore, in this study, we assessed whether the combination of tetrazolone-based SM-06-09 and anti-PD-1 therapy would replicate our earlier findings. Oral daily administration of SM-06-09 (**3m**) at 25, 50, and 100 mg/kg doses to mice bearing SM1 tumors indicated 100 mg/kg as an optimal dose (Figure S10A, Figure S10B). No side effects, including changes in body weight or toxicity in the liver and spleen, were observed at this dose based on poststudy organ observations (Figure S10C, Figure S10D).

For the combination study, mice received daily oral administration of vehicle or SM-06-09 (**3m**) and i.p. injections of IgG or anti-PD-1 antibodies three times a week at a dose of 10 mg/kg. Tumor measurements were recorded on alternate days, and body weights were recorded weekly. The tumor growth kinetics in Figure 7A indicate that the combination of SM-06-09 and anti-PD-1 resulted in improved tumor reduction compared to SM-06-09 and anti-PD-1 used alone. Figure 7B shows the sizes of representative tumors measured in the tumor growth kinetics. Phenotyping of the immune cell infiltrate was performed by flow cytometry after harvesting the tumors at the end point. Overall, the combination therapy increased the infiltration of CD45+ total immune cells compared to control and single-arm therapies, as shown in Figure 7C. This increase in CD45+ total immune cells also resulted in an increase in the percentage of F4/80+ tumor macrophages. More importantly, the combination therapy increased the percentage of antitumor M1-like macrophages, while it did not significantly alter the tumor-promoting M2-like macrophages. Consequently, this increased the M1/M2 macrophage ratio with combination therapy. Although there is a slight increase in the M1/M2 ratio with single-arm therapies, the magnitude of increase with the combination therapy is much higher. With respect to the tumor-infiltrated lymphoid cells, we observed an overall increase in CD3+ cells that resulted in an increase of both CD8 and CD4 T-cells; however, the percentage of infiltrated CD8 T-cells was much higher than CD4 T-cells (Figure 7D). Also, the combination therapy resulted in an increase in the percentage of CD44HiCD62LLo CD8 effector memory T-cells, and more importantly, CD44HiCD62LHi CD8 central memory cells compared to single-arm therapy groups. We further analyzed the tumor immune infiltrate by mass cytometry on the Fluidigm Helios platform. Figure 8A represents the dimensionality reduction of the Opt-SNE assay, demonstrating the clear separation of CD45+ tumor immune infiltrate into macrophages, myeloid-derived suppressor cells (MDSCs), CD4 T-cells, and CD8 T-cells.

**Figure 7:**
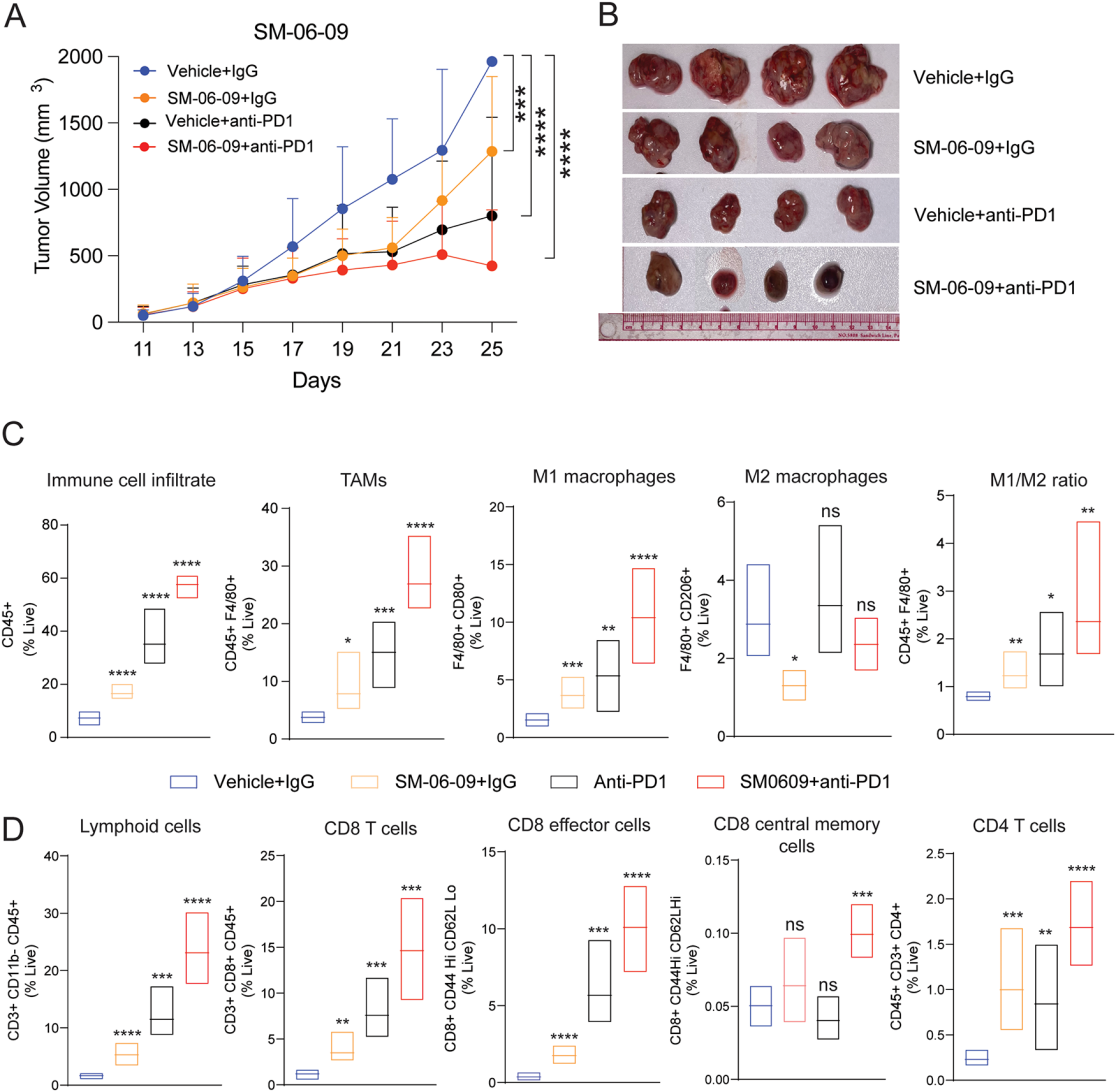
Combining SM-06-09 and anti-PD-1 antibody enhances the antitumor effect in SM1 murine melanoma model. Combining SM-06-09 (100 mg/kg, oral, daily dosing) with anti-PD-1 antibodies (10 mg/kg, twice a week, i.p. dosing) enhanced the antitumor effects compared to mono treatments in the SM1 murine melanoma model. At the end of the study, tumors were harvested for immunotyping with flow cytometry. (**A**) Kinetics of tumor growth (*n* = 10) and (**B**) representative tumor images. ****P < 0.0001, two-way ANOVA. Flow cytometry characterization of tumor-associated macrophages (**C**), and lymphoid cells (**D**). ns, not significant, *P < 0.05, **P < 0.01, ***P < 0.001, ****P < 0.0001, unpaired *t* test was performed with each treatment group compared to the Vehicle + IgG group.

**Figure 8:**
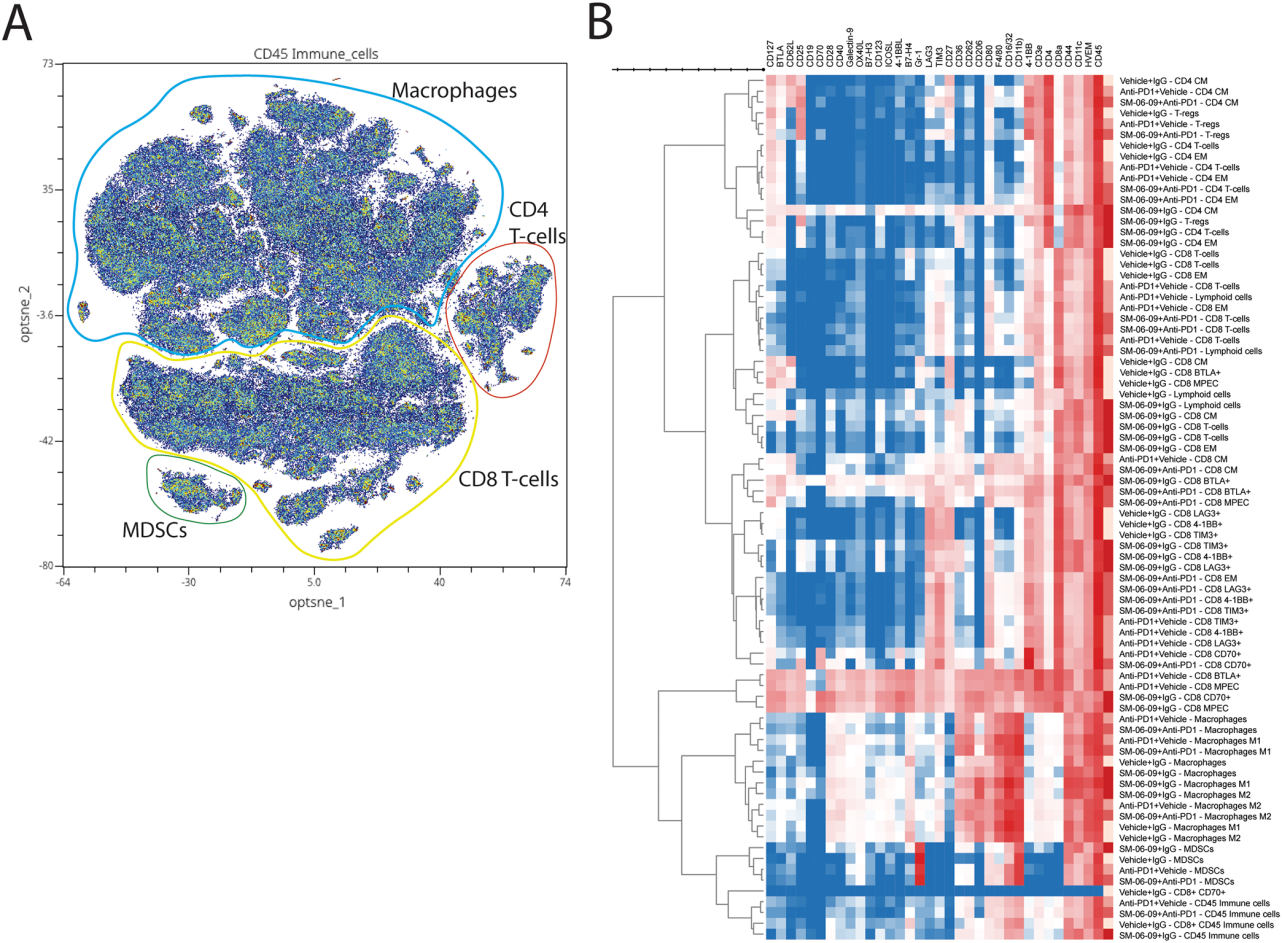
Mass cytometry characterization of tumor-associated immune cells by CyToF. (**A**) Mass Cytometry analysis of CD45+ tumor immune infiltrate represented as an opt-SNE plot. (**B**) Heatmap illustrating the clustering of various immune cell populations across treatment groups. *n* = 6 per group, *P < 0.05, **P < 0.01, ***P < 0.001, ****P < 0.0001, unpaired *t* test was performed with each treatment group compared to the Vehicle + IgG group.

Analysis of gated populations by cluster analysis indicated a clear separation between CD3+ lymphoid cells and CD11b+ myeloid cells in the heatmap (Figure 8B). Dendrogram sub-branches further indicate the presence of distinct CD4 T-cell populations positive for CD4 and CD25, representing regulatory T-cells (T-regs), CD44HiCD62LLo CD4 effector memory (EM) cells, and CD44HiCD62LHi CD4 central memory (CM) cells. With CD8 T-cells, Anti-PD-1 and SM-06-09+Anti-PD-1 groups had the highest CD8 EM T-cells, while CD44HiCD62LHi CD4 CM cells were primarily present in the Anti-PD-1 group. In the myeloid compartment, Gr-1+ MDSCs were present in all the treatment groups along with CD206+ M2 macrophages. However, antitumor CD80+ M1 macrophages were significantly present in the Anti-PD-1 and SM-06-09+Anti-PD-1 groups. The increase in the number of CD80+ M1-like macrophages in the Anti-PD-1 and SM-06-09+Anti-PD-1 groups, accompanied by a concomitant decrease in CD206+ M2-like macrophages, as shown in the feature plots in Figure S11, represents a shift in the tumor immune landscape. The increased M1/M2 macrophage ratio observed with combination therapy is consistent with a shift toward an antitumor immune landscape, as M1-like macrophages exhibit pro-inflammatory characteristics, enhanced antigen presentation, and the capacity to recruit cytotoxic CD8+ T-cells and NK cells to the TME.^80^ Notably, a higher M1/M2 ratio among TAMs has been associated with improved overall survival across multiple cancer types,^81^ lending clinical relevance to the preclinical findings reported here. In conclusion, SM-06-09 (**3m**), in combination with anti-PD-1, significantly reduced tumor growth by increasing the M1/M2 macrophage ratio and enhancing infiltration of antitumor CD8 effector memory cells.

### The Combination of SM-06-09 and Anti-PD-1 Therapy Enhances the Tumor Immune Landscape for Immune-Mediated Tumor Killing

Single-cell RNA sequencing data comparing the immune cell types within tumors treated with SM-06-09 (**3m**), anti-PD-1, or combination reveals macrophages as the most abundant immune cell type (Figure 9A,B). After creating a subset of tumor-associated macrophages (TAMs), we examined the expression of various M1 and M2 macrophage markers and observed an increase in M1 markers, including *Il1b*, *Ccl8*, *Cxcl3*, *Nos2*, *Cd80*, and *Cd86*, in tumors treated with SM-06-09 (**3m**) (Figure 9C). In tumors treated with anti-PD-1 therapy, M1 markers such as *Tnf*, *Il1b*, *Cxcl16*, and *Cxcl1* increased, and expression of most M2 markers decreased. The combination group showed the lowest expression of most M2 markers and mixed expression of M1 markers (Figure 9C). To look beyond the M1-M2 paradigm, we identified eight distinct subpopulations of TAMs and labeled them based on their putative functions, as determined by the enrichment of differentially expressed genes (Figure 9D,E). SM-06-09 (**3m**) increased the proportion of both inflammatory and phagocytic TAMs, as did anti-PD-1 therapy alone. The combination, however, increased a population of macrophages that expressed genes related to epithelial-mesenchymal transition (EMT) and ECM breakdown, such as *Mmp9.* This subpopulation warrants further investigation. Overall, each therapy reduced the proportion of TAMs involved in angiogenesis and fibrosis, classic functions of pro-tumoral macrophages (Figure 9E). Trajectory analysis of monocytes and macrophages showed a monocyte node and two main macrophage nodes, one correlating with a more “M2-like” signature and the other “M1-like” (Figure 9F, Figure S11). Vehicle-treated tumors show a relatively even temporal gradient to from monocytes to M1- or M2-like macrophages, with slightly further differentiated cells reflecting “M2-like” nature. However, in tumors treated with combination therapy, the M1 node is associated with further differentiated cells, with a trajectory indicating monocyte-derived M2-like macrophages further evolve toward the M1-like node (Figure 9F). These results suggest that combining a-PD-1 and HDAC6i shifts previously differentiated M2-like macrophages in the tumor toward an M1-like signature rather than causing monocytes to directly differentiate to M1-like macrophages.

**Figure 9:**
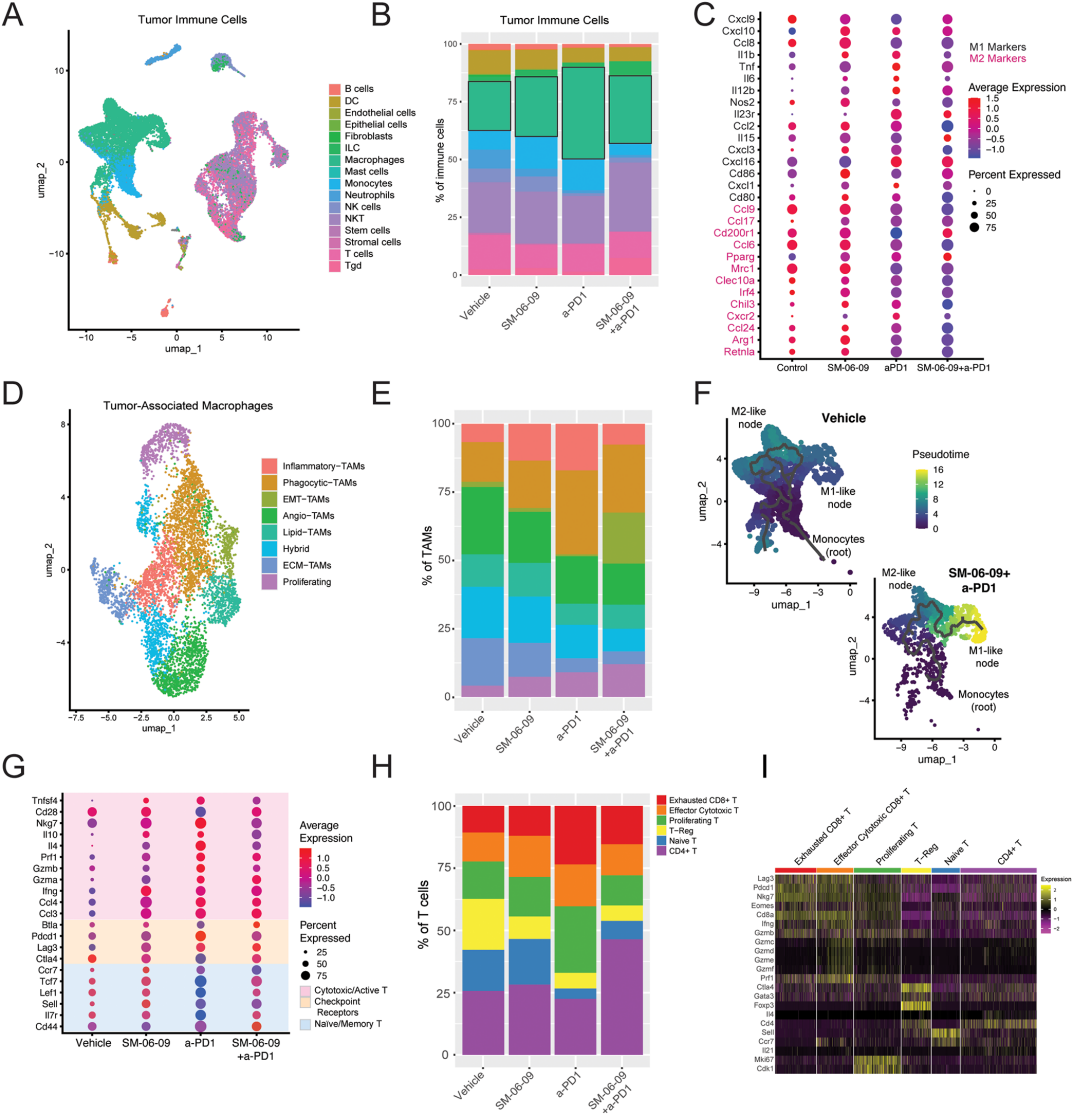
Single-cell transcriptome and mass cytometry analysis of SM1 melanoma tumors from animals administered with SM-06-09 and anti-PD-1 antibodies alone or in combination. (**A**) UMAP displaying all tumor immune cell types, and (**B**) quantification of immune cell proportion by treatment group. (**C**) Dot plot of M1 and M2 marker expression in macrophages from tumors of each treatment group. Color indicates relative expression level; size indicates percentage of macrophages with expression. (**D**) UMAP and (**E**) proportions of tumor-associated macrophages, grouped by subpopulations and treatment type. (**F**) Trajectory analysis using Monocle 3 indicating change in monocyte-macrophage fate with combination therapy. (**G**) Expression of cytotoxic T cell markers, T cell checkpoint receptors, and naïve T cell markers within tumor-infiltrating T cells, grouped by treatment. (**H**) Proportions of T cell subtypes by treatment group. (**I**) Heatmap displaying markers associated with each T cell subtype.

To evaluate the effects of these treatments on T-cells, we isolated a subset of tumor-infiltrating T cells and assessed the expression of naïve T cell markers, cytotoxic T cell markers, and checkpoint receptors. Anti-PD-1 therapy increased cytotoxic T cell markers and decreased naïve T cell markers; however, in combination, this effect was not as pronounced (Figure 9G). Additionally, we observed a decrease in regulatory T cells (T-Regs) in all three treatment groups, with the smallest in the combination therapy (Figure 9H) and identified six distinct T cell subpopulations based on differentially expressed markers (Figure 9I). The reduction in T-regs is possibly mediated by macrophages and their secreted cytokines, which inhibit T-cell differentiation. Additionally, combination-treated tumors have the highest proportion of CD4+ helper T cells, essential for sustaining the antitumor immune response (Figure 9H). While clear changes in T-cell populations accompany both HDAC6 inhibition and immune checkpoint blockade, the T-cell gene-expression changes appear to be driven primarily by anti-PD-1 therapy. The comparatively modest alterations in T-cell activity observed with SM-06-09 (**3m**) alone are consistent with an indirect mechanism mediated by macrophage reprogramming rather than a direct effect of HDAC6 inhibition on T-cells, further supporting a combined approach.^82,83^ More broadly, the subpopulation-level resolution afforded by single-cell transcriptomics reveals that TAM heterogeneity extends well beyond the conventional M1/M2 framework, and a deeper analysis of how HDAC6 inhibition and checkpoint blockade modulate specific TAM functional states, rather than bulk polarization markers, will be critical for understanding the full spectrum of immune remodeling within the tumor microenvironment.

## Discussion and Conclusions

Current anticancer immunotherapeutic strategies, including immune checkpoint inhibitors, have primarily focused on reviving T-cell effector functions. While they have shown promising outcomes in solid tumors, a significant percentage of patients are either unresponsive or develop resistance to these therapies, emphasizing that novel anticancer interventions are warranted.^84^ The growing understanding of tumor-associated innate immune cells, including dendritic cells, myeloid-derived suppressor cells, and TAMs, and their crosstalk with other immune and nonimmune cells within the TME, has generated an interest in using them as tools or targets for driving anticancer responses.^82^ However, macrophage-based pipelines for screening novel anticancer therapeutics are underdeveloped. Here, we developed and screened structurally novel HDAC6 inhibitors, identifying a lead compound, SM-06-09, that enhances the functional properties of pro-inflammatory macrophages and exhibits a superior anticancer effect by reducing protumoral M2-like macrophages.

Pan-HDAC inhibitors that are currently approved for clinical trials have direct cytotoxic effects on cancer cells but are associated with severe side effects, leading to treatment discontinuation.^85^ Therefore, our group has focused on isoform-specific HDAC6 inhibitors with minimal cytotoxicity and has extensively characterized their antitumor activity via modulation of macrophage phenotype and function. The novel tetrazolone derivatives developed were highly selective for HDAC6 inhibition with minimal cytotoxicity. Among them, nine candidates showed a dose-dependent increase in acetyl-tubulin, a substrate and target of HDAC6, confirming effective HDAC6 inhibition. While the compounds displayed subnanomolar inhibitory values against purified HDAC6, the upregulation of acetyl-tubulin is strikingly higher starting at 2.5 μM. We acknowledge this large difference in biochemical inhibition versus cellular activity, and believe several factors like cellular permeability, localization, target engagement, and time-dependent effects likely contribute to this gap. To investigate this further, future studies will incorporate cellular thermal shift assays, NanoBRET binding assays, and live-cell imaging. The classification of macrophages into M1 and M2 phenotypes is somewhat simplistic, as they are extremely plastic and can switch between or exhibit hybrid phenotypes shaped by cues from the surrounding environment.

The tetrazolone-based hydroxamic acids **3a**–**3q** display a coherent structure–activity relationship governed by three principal determinants: linker geometry, cap-rim complementarity, and ZBG coordination quality. The ethylene linker provides the optimal spatial vector for cap positioning, and aryl cap groups are strongly preferred over aliphatic chains, with the 3-quinolinyl cap of **3m** achieving the highest potency (IC_50_ = 0.49 nM) and an exceptional LipE of 9.23. Substitution on the phenyl cap is tolerated at the *para* and *meta* positions but is deleterious at the *ortho* position, where steric clash disrupts productive binding, a finding illustrated by the 46-fold potency loss for *ortho*-iodo **3h**. The isoxazole-3-hydroxamate ZBG is essential and cannot be productively replaced by thiadiazole or triazole alternatives. Collectively, these findings establish the tetrazolone linker as a viable bioisosteric replacement for the amide linker of SS-208,^28^ yielding compounds with improved or retained HDAC6 potency and favorable efficiency metrics. Among the selected nine candidate drugs, **3b**, SM-06-09 (**3m**), and **3l**, tested at 5 and 10 μM concentrations in BMDMs, modulated the macrophage phenotype as observed by changes in the levels and expression of M1 (iNOS, TNF-α) and M2 (Arg1 and Fizz1) markers. Specifically, SM-06-09 (**3m**) appeared more potent, with significant inhibition of the M2-like phenotype at lower concentrations.

It is now well established that TAMs with a predominantly anti-inflammatory M2-like phenotype play a crucial role in promoting a pro-tumor, immunosuppressive TME by secreting growth and angiogenic factors.^77,78^ Antigen cross-presentation is essential for initiating CD8+ T-cell responses, and the increased expression of MHC class I antigen presentation observed in our analysis, in conjunction with the coexistence of pro-inflammatory macrophages, creates a TME conducive to enhanced CD8+ T-cell activation. Activated CD8+ T-cells can also influence macrophage function by producing cytokines and other stimulatory signals. CD8+ T-cells can further drive macrophages toward an M1 phenotype, amplifying the response against tumor cells.^76^ Furthermore, the proliferation of CD8 T-cells is suppressed when cocultured with M2 macrophages. M1-like macrophages, on the other hand, exhibit pro-inflammatory characteristics, enhanced antigen presentation and phagocytic activity, and recruit other antitumor immune cells, such as CD8+ T-cells and NK-cells, to the TME.^80^ The higher M1/M2 ratio of TAMs is associated with better overall survival in cancer patients.^81^

Our single-cell RNA sequencing analysis reveals a more complex heterogeneity in TAMs. Each treatment type modulates the expression of different M1 and M2 macrophage markers to varying degrees, and clustering analysis reveals several distinct TAM subpopulations. With HDAC6 inhibition and immune checkpoint inhibition, there is a decrease in macrophages associated with pro-tumoral activities such as angiogenesis, immunosuppression, and fibrosis, and an increase in macrophages that promote inflammation and phagocytosis. Additionally, trajectory analysis indicates that combination therapy shifts previously differentiated M2-like macrophages to an M1-like state, rather than altering monocyte differentiation directly. This supports the current paradigm that more pro-inflammatory macrophages and fewer pro-tumoral macrophages are associated with tumor reduction.^82,83^ However, a deeper analysis of the heterogeneity of TAMs and how different antitumor therapies alter them is a critical area for future investigation, as changes in more specific subpopulations reflect more nuanced alterations in macrophage activity. A new approach to evaluating TAM functional changes from anticancer therapies beyond M1 and M2 is necessary. While there are clear changes in T-cell populations in tumors treated with HDAC6 inhibitors and/or immune checkpoint inhibitors, the changes in T-cell gene expression appear primarily driven by ICB. We believe that the modest changes to T-cell activity in tumors treated with an HDAC6 inhibitor alone are mainly due to alterations in macrophage interactions with T-cells, rather than a direct effect, supporting a combined approach involving HDAC inhibition and immune checkpoint therapy.

Taken together, using a macrophage-specific screening pipeline, we identified SM-06-09 (**3m**) as a next-generation HDAC6-selective inhibitor that reprograms TAMs, enhances antigen presentation, and promotes antitumor immunity without causing direct cytotoxicity. These data provided a precise framework and preclinical rationale for the clinical translation of selective HDAC6is compounds in combination with immune checkpoint blockade to overcome immunosuppression and improve therapeutic outcomes in solid tumors. Our future studies will focus on the translational relevance of SM-06-09 (**3m**) in human TAMs and on delineating the downstream signaling pathways.

## Experimental Section

### Chemistry

#### General Methods

All nonaqueous reactions were carried out in oven- or flame-dried glassware under a dry nitrogen atmosphere. Anhydrous dichloromethane (DCM), methanol (MeOH), and tetrahydrofuran (THF) were passed through a solvent dispensing system under a dry argon atmosphere. Unless otherwise noted, all other reagents and starting materials were purchased from commercial vendors and used without further purification. Unless otherwise stated, all reactions were magnetically stirred and monitored by analytical thin-layer chromatography (TLC) using glass-backed silica gel plates with an F254 indicator. Visualization was accomplished using UV light (254 nm), a potassium permanganate solution, or a phosphomolybdic acid solution. Purification by flash column chromatography was performed with SiliaFlash F60 (40–63 μm, 60 Å) from Silicycle Inc. Yields refer to chromatographically and spectrographically pure compounds unless otherwise noted. All melting points were determined in unsealed Pyrex capillaries with a Thomas-Hoover Unimelt melting point apparatus and are uncorrected. Infrared spectra were recorded using a Nicolet iS5 FTIR spectrophotometer. ^1^H NMR and ^13^C­(12) NMR spectra were recorded on a Bruker Avance 400 (400 MHz, ^1^H, 100 MHz, ^13^C) or a Bruker Avance 500 (500 MHz, ^1^H, 125 MHz, ^13^C) spectrometer. Chemical shift values (δ) are reported in ppm relative to residual chloroform (δ 7.26 ppm for ^1^H; 77.5 ppm for ^13^C), methanol (δ 3.31 ppm for ^1^H; δ 49.2 ppm for ^13^C), acetone (δ 2.05 ppm for 1H; δ 29.9 ppm for ^13^C), and dimethyl sulfoxide (δ 2.50 ppm for ^1^H; δ 39.5 ppm for ^13^C); Multiplicities are indicated by s (singlet), d (doublet), t (triplet), q (quartet), p (pentet), h (heptet), m (multiplet) and br (broad) app (apparent). The identification of ^1^H and ^13^C signals was achieved using a combination of ^1^H, ^13^C, DEPT, COSY, HMBC, HMQC, and NOESY experiments.

High-resolution electrospray ionization mass spectra (HRMS ESI-TOF and EI-TOF) were obtained on a Waters Micromass Q-Tof Ultima instrument at the University of Illinois Mass Spectrometry Laboratory. High-resolution electron ionization mass spectra (HRMS-ESI) were obtained on a Waters Micromass 70-VSE instrument at the same facility. Mass spectral data are reported as the *m*/*z* ratio in Daltons, with <5 ppm error.

Preparative HPLC was used in the purification of all final compounds using a Waters 2545 Binary Gradient Module equipped with a Waters 2489 UV/Visible Detector under the following conditions: column, Waters XSelect CSH C18 (150 × 19 mm, 5 μm); mobile phase, 5–95% acetonitrile/water containing 0.05% TFA at a flow rate of 10.0 mL/min; gradient, 0–5 min 5% B, 5–15 min 5–95% B, 15–20 min 95% B; UV detection at 254 nm. Analytical HPLC was used to determine the purity of all final compounds using the same system fitted with a Waters XSelect CSH C18 analytical column (150 × 4.6 mm, 5 μm) under the following conditions: mobile phase, 5–95% acetonitrile/water containing 0.05% TFA at a flow rate of 1.0 mL/min; gradient, 0–5 min 5% B, 5–15 min 5–95% B, 15–20 min 95% B; UV detection at 254 nm. All final compounds were ≥95% pure as determined by analytical HPLC.

### General Synthetic Methods

#### General Procedure A

An oven-dried round-bottom flask (25 mL) equipped with a magnetic stirring bar and a condenser was sequentially charged with aroyl chloride (5 mmol, 1 equiv) and trimethylsilyl azide (2.60 mL, 20 mmol, ρ = 0.876 g/mL, 4 equiv) (CAUTION!). The mixture was placed under N_2_, heated to 100 °C and stirred for 12 h. After cooling to ambient temperature (25 °C), the mixture was concentrated *in vacuo*, and the residue partitioned between EtOAc (15 mL) and H_2_O (15 mL). The organic phase was separated and extracted with saturated aqueous NaHCO_3_ solution (4 × 15 mL). The aqueous extracts were combined, the pH adjusted to <3 using aqueous HCl (6 M) and then extracted with EtOAc (3 × 20 mL). The combined extracts were dried (MgSO_4_) and concentrated *in vacuo* to afford *N*-aryl tetrazolone, which was used without further purification.

#### General Procedure B

An oven-dried round-bottom flask (25 mL) equipped with a magnetic stirring bar was charged with powdered, anhydrous Cs_2_CO_3_ (1.17 g, 3.6 mmol, 1.2 equiv), evacuated under reduced pressure and flushed with N_2_. A solution of *N*-aryl tetrazolone (3.0 mmol, 1.0 equiv) and alkylating agent (3.9 mmol, 1.3 equiv) in anhydrous DMF (6.0 mL, 0.3 M) was then added dropwise. The stirred reaction mixture was heated at 60 °C for 12 h, cooled to ambient temperature (25 °C), and then diluted with EtOAc (10 mL). The reaction mixture was washed sequentially with H_2_O (3 × 10 mL) and brine (10 mL), dried (Na_2_SO_4_), and concentrated *in vacuo*. The remaining residue was purified by column chromatography on silica gel to provide the *N*-alkyl-*N-*aryl tetrazolone.

#### General Procedure C

An oven-dried round-bottom flask (25 mL) equipped with a magnetic stirring bar was charged with ethyl nitroacetate (444 μL, ρ = 1.20 g/mL, 4.0 mmol, 2.0 equiv), DABCO (17 mg, 0.15 mmol, 0.1 equiv), alkyne (2.0 mmol, 1.0 equiv), and anhydrous EtOH (3.0 mL, 0.67 M). The vessel was flushed with N_2_, sealed, and heated at 100 °C for 12 h. After cooling to ambient temperature (25 °C), the reaction was concentrated *in vacuo*, and the remaining residue taken up in EtOAc (10 mL), washed sequentially with H_2_O (3 × 10 mL) and brine (10 mL), dried (Na_2_SO_4_), and then concentrated *in vacuo*. The residue was purified by flash chromatography on silica gel (hexanes/EtOAc) to provide ethyl isoxazole-3-carboxylate.

#### General Procedure D

To a stirred solution of isoxazole-3-carboxylate (1 mmol, 1.0 equiv) in a mixture of THF and MeOH (1:1, 2.0 mL) at ambient temperature (25 °C) was added a solution of KCN (18 mg, 0.28 mmol, 0.28 equiv) in aqueous hydroxylamine (0.6 mL, 50% solution in H_2_O). After 12 h, the reaction mixture was diluted with CH_2_Cl_2_ (3.0 mL) and the biphasic mixture extracted with H_2_O (3 × 3.0 mL). The aqueous phase was acidified to pH 4 with aqueous HCl (1.0 M), and the resulting precipitate removed by filtration. The precipitate was washed sequentially with H_2_O (3 × 5 mL) and hexanes (3 × 5 mL), dried *in vacuo*, and then purified by preparative HPLC using the methods noted to provide hydroxamic acid.

#### General Procedure E

To a stirred solution of isoxazole-3-carboxylate (1.0 mmol, 1.0 equiv) in MeOH (2.0 mL) at ambient temperature (25 °C) was sequentially added dropwise, a solution of hydroxylamine hydrochloride (625 mg, 9.0 mmol, 9.0 equiv) in MeOH (1.0 mL) and aqueous NaOH (6.0 M, 0.2 mL, 1 mmol, 1.0 equiv). After 24 h, the reaction mixture was diluted with H_2_O (5.0 mL), acidified to pH 6 with aqueous HCl (2 M) then extracted with EtOAc (3 × 5 mL). The combined extracts were dried (Na_2_SO_4_) and concentrated *in vacuo*, and the remaining residue purified by preparative HPLC using the methods noted to provide hydroxamic acid.

### Preparation of Compounds **3a**–**3q**

#### Compounds **5a**–**5o** were Synthesized According to General Procedure B

##### 4-Phenyl-1-(prop-2-yn-1-yl)-1,4-dihydro-5*H*-tetrazol-5-one (**5a**)

Colorless oil, 51% yield; FTIR (neat) ν_max_ 3266, 1679, 1638, 1596, 1556, 1494, 1455, 1440, 1361, 1332, 1290 cm^–1^; ^1^H NMR (500 MHz, CDCl_3_) δ 7.93 (d, *J* = 7.6 Hz, 2H), 7.50 (t, *J* = 7.52 Hz, 2H), 7.38 (t, *J* = 7.4 Hz, 1H), 4.82 (d, *J* = 2.6 Hz, 2H), 2.46 (t, *J* = 2.6 Hz, 1H); ^13^C­{^1^H} NMR (126 MHz, CDCl_3_) δ 129.6, 129.2, 125.7, 123.2, 121.7, 77.8, 75.8, 60.9. HRMS (ESI/Q-TOF) *m*/*z*: [M + H]^+^ calcd for C_10_H_9_N_4_O: 201.0776; found 201.0778.

##### 1-(But-3-yn-1-yl)-4-phenyl-1,4-dihydro-5*H*-tetrazol-5-one (**5b**)

Colorless oil, 52% yield; FTIR (neat) ν_max_ 3298, 1723, 1597, 1563, 1502, 1461, 1388, 1293, 1156, 1126, 1098 cm^–1^; ^1^H NMR (500 MHz, CDCl_3_) δ 7.94 (d, *J* = 8.5 Hz, 2H), 7.50 (t, *J* = 7.5 Hz, 2H), 7.38 (t, *J* = 7.6 Hz, 1H), 4.21 (t, *J* = 7.0 Hz, 2H), 2.84–2.76 (m, 2H), 2.05 (s, 1H); ^13^C­{^1^H} NMR (126 MHz, CDCl_3_) δ 148.9, 134.6, 129.4, 127.7, 121.6, 119.2, 79.0, 71.2, 43.6, 18.6. HRMS (ESI/Q-TOF) *m*/*z*: [M + H]^+^ calcd for C_11_H_11_N_4_O: 215.0933; found 215.0925.

##### 1-(Pent-4-yn-1-yl)-4-phenyl-1,4-dihydro-5*H*-tetrazol-5-one (**5c**)

Colorless oil, 80% yield; FTIR (neat) ν_max_ 3209, 3075, 2969, 2167, 1725, 1572, 1465, 1365, 1254, 1130, 980, 895 cm^–1^; ^1^H NMR (500 MHz, CDCl_3_) δ 7.93 (d, *J* = 8.7 Hz, 2H), 7.48 (t, *J* = 7.5 Hz, 2H), 7.35 (t, *J* = 7.4 Hz, 1H), 4.14 (t, *J* = 7.0 Hz, 2H), 2.34 (dt, *J* = 6.9, 2.7 Hz, 2H), 2.13–2.08 (m, 2H), 2.01 (t, *J* = 2.7 Hz, 1H); ^13^C­{^1^H} NMR (126 MHz, CDCl_3_) δ 149.1, 134.6, 129.4, 127.7, 119.2, 82.1, 69.7, 43.9, 27.1, 15.8. HRMS (ESI/Q-TOF) *m*/*z*: [M + H]^+^ calcd for C_12_H_13_N_4_O 229.1089; found 229.1090.

##### 1-(Hex-5-yn-1-yl)-4-phenyl-1,4-dihydro-5*H*-tetrazol-5-one (**5d**)

Colorless oil, 71% yield; FTIR (neat) ν_max_ 3100, 3096, 2955, 1755, 1560, 1480, 1388, 1250, 1125, 988 cm^–1^; ^1^H NMR (500 MHz, CDCl_3_) δ 7.95 (d, *J* = 7.6 Hz, 2H), 7.50 (t, *J* = 7.4 Hz, 2H), 7.37 (t, *J* = 7.4 Hz, 1H), 4.07 (t, *J* = 7.0 Hz, 2H), 2.30 (dt, *J* = 7.0, 2.7 Hz, 2H), 2.13–2.08 (m, 2H), 1.98 (t, *J* = 2.7 Hz, 1H), 1.68–1.62 (m, 2H); ^13^C­{^1^H} NMR (126 MHz, CDCl_3_) δ 149.1, 134.7, 129.4, 127.7, 119.2, 83.3, 69.1, 44.5, 27.4, 25.1, 17.8. HRMS (ESI/Q-TOF) *m*/*z*: [M + H]^+^ calcd for C_13_H_15_N_4_O: 243.1246, found 243.1245.

##### 1-(4-Bromophenyl)-4-(but-3-yn-1-yl)-1,4-dihydro-5*H*-tetrazol-5-one (**5e**)

Colorless oil 70%: FTIR (neat) ν_max_ 3291, 3025, 2969, 1725, 1563, 1469, 1306, 1207, 1159, 977, 816 cm^–1^; ^1^H NMR (500 MHz, CDCl_3_) δ 7.88 (app dt, *J* = 9.0, 2.1 Hz, 2H), 7.63 (app dt, *J* = 9.0, 2.1 Hz, 2H), 4.21 (t, *J* = 7.0 Hz, 2H), 2.80 (dt, *J* = 7.0, 2.7 Hz, 2H), 2.06 (t, *J* = 2.7 Hz, 1H); ^13^C­{^1^H} NMR (126 MHz, CDCl_3_) δ 148.6, 133.7, 132.5, 121.2, 120.5, 78.8, 71.3, 43.7, 18.6. HRMS (ESI/Q-TOF) *m*/*z*: [M + H]^+^ calcd for C_11_H_10_BrN_4_O: 293.0038; found 293.0034.

##### 1-(But-3-yn-1-yl)-4-(*m*-tolyl)-1,4-dihydro-5*H*-tetrazol-5-one (**5f**)

Colorless oil, 73%: FTIR (neat) ν_max_ 3156, 3011, 2989, 1730, 1525, 1430, 1377, 1267, 1125, 989, 824 cm^–1^; ^1^H NMR (500 MHz, CDCl_3_) δ 7.76–7.72 (m, 2H), 7.38 (t, *J* = 4.8 Hz, 1H), 7.19 (d, *J* = 7.6 Hz, 1H), 4.21 (t, *J* = 7.1 Hz, 2H), 2.80 (dt, *J* = 7.1, 2.7 Hz, 2H), 2.43 (s, 3H), 2.06 (t, *J* = 2.7 Hz, 1H); ^13^C­{^1^H} NMR (126 MHz, CDCl_3_) δ 148.9, 139.6, 134.5, 129.2, 128.6, 119.9, 116.5, 79.0, 71.2, 43.6, 21.5, 18.6. HRMS (ESI/Q-TOF) *m*/*z*: [M + H]^+^ calcd for C_12_H_13_N_4_O: 229.1089 found 229.1087.

##### 1-(But-3-yn-1-yl)-4-(3-fluorophenyl)-1,4-dihydro-5*H*-tetrazol-5-one (**5g**)

Colorless oil, 69% yield; FTIR (neat) ν_max_ 3295, 1728, 1509, 1464, 1403, 1359, 1297, 1271 cm^–1^; ^1^H NMR (500 MHz, CDCl_3_) δ 7.82–7.76 (m, 2H), 7.49–7.45 (m, 1H), 7.09–7.05 (m, 1H), 4.21 (t, *J* = 7.0 Hz, 2H), 2.80 (dt, *J* = 7.0, 2.7 Hz, 2H), 2.06 (t, *J* = 2.7 Hz, 1H); ^13^C­{^1^H} NMR (126 MHz, CDCl_3_) δ 163.9, 161.9, 148.6, 130.9 (d, *J* = 9.1 Hz), 114.5 (d, *J* = 21.2 Hz), 114.3 (d, *J* = 3.3 Hz), 106.6 (d, *J* = 27.2 Hz), 78.8, 71.3, 43.6, 18.6. HRMS (ESI/Q-TOF) *m*/*z*: [M + H]^+^ calcd for C_11_H_10_FN_4_O: 233.0839; found 233.0833.

##### 1-(But-3-yn-1-yl)-4-(2-iodophenyl)-1,4-dihydro-5*H*-tetrazol-5-one (**5h**)

Colorless oil, 69% yield; FTIR (neat) ν_max_ 3246, 1714,1566, 1480, 1456, 1442, 1354, 1298, 1227 cm^–1^; ^1^H NMR (500 MHz, CDCl_3_) δ 7.97 (dd, *J* = 8.0,1.4 Hz, 1H), 7.49 (dt, *J* = 7.9, 1.4 Hz 1H), 7.40 (dd, *J* = 7.82, 1.68 Hz, 1H), 7.22 (dt, *J* = 8.0, 1.7 Hz, 1H), 4.20 (t, *J* = 7.0 Hz, 2H), 2.80 (td, *J* = 7.0, 2.7 Hz, 2H), 2.07 (t, *J* = 2.7 Hz, 1H); ^13^C­{^1^H} NMR (126 MHz, CDCl_3_) δ 149.1, 140.3, 135.7, 132.0, 129.5, 129.0, 96.3, 79.1, 71.4, 43.8, 18.7. HRMS (ESI/Q-TOF) *m*/*z*: [M + H]^+^ calcd for C_11_H_10_IN_4_O: 340.9899; found 340.9899.

##### 1-(But-3-yn-1-yl)-4-(2-fluorophenyl)-1,4-dihydro-5*H*-tetrazol-5-one (**5i**)

Colorless oil, 66% yield; FTIR (neat) ν_max_ 3295, 1728, 1509, 1464, 1403, 1359, 1297, 1271 cm^–1^; ^1^H NMR (500 MHz, CDCl_3_) δ 7.53 (td, *J* = 7.6, 1.8 Hz, 1H), 7.49–7.44 (m, 1H), 7.27 (tdd, *J* = 8.5, 4.5, 1.4 Hz, 2H), 4.19 (t, *J* = 7.0 Hz, 2H) 2.79 (td, *J* = 7.0, 2.6 Hz, 2H), 2.07 (t, *J* = 2.7 Hz, 1H); ^13^C­{^1^H} NMR (126 MHz, CDCl_3_) δ 157.2, 155.4, 149.3, 132.2 (d, *J* = 7.9 Hz), 127.0, 125.3 (d, *J* = 4.1 Hz), 121.3 (d, *J* = 12.0 Hz), 117.1 (d, *J* = 19.1 Hz), 79.2, 71.4, 44.4, 19.2. HRMS (ESI/Q-TOF) *m*/*z*: [M + H]^+^ calcd for C_11_H_10_FN_4_O: 233.0839; found 233.0841.

##### 1-(But-3-yn-1-yl)-4-(4-fluoro-3-iodophenyl)-1,4-dihydro-5*H*-tetrazol-5-one (**5j**)

White solid, 74% yield; mp 80–84 °C; FTIR (neat) ν_max_ 3288, 3276, 1712, 1591, 1488, 1439, 1419, 1399, 1376, 1352 cm^–1^; ^1^H NMR (500 MHz, CDCl_3_) δ 8.36 (dd, *J* = 5.3, 2.6 Hz, 1H), 7.94 (ddd, *J* = 9.0, 4.3, 2.7 Hz, 1H), 7.17 (dd, *J* = 9.0, 7.2 Hz, 1H), 4.19 (t, *J* = 6.9 Hz, 2H), 2.79 (td, *J* = 7.0, 2.7 Hz, 2H), 2.06–2.04 (m, 1H); ^13^C­{^1^H} NMR (101 MHz, CDCl_3_) δ 162.0, 160.2, 149.0, 132.3, 130.1, 121.1 (d, *J* = 7.7 Hz), 116.04 (d, *J* = 25.8 Hz), 82.0 (d, *J* = 27.7 Hz), 79.2, 71.0, 44.0, 19.4. HRMS (ESI/Q-TOF) *m*/*z*: [M + H]^+^ calcd for C_11_H_9_FIN_4_O: 358.9805; found 358.9803.

##### 1-(But-3-yn-1-yl)-4-(4-chloro-3-iodophenyl)-1,4-dihydro-5*H*-tetrazol-5-one (**5k**)

Colorless oil, 75% yield; FTIR (neat) ν_max_ 3291, 3025, 2969, 1725, 1563, 1469, 1306, 1207, 1159, 977, 816 cm^–1^; ^1^H NMR (500 MHz, CDCl_3_) δ 8.49 (d, *J* = 2.5 Hz, 1H), 7.95 (dd, *J* = 8.8, 2.6 Hz, 1H), 7.54 (d, *J* = 8.8 Hz, 1H), 4.20 (t, *J* = 7.0 Hz, 2H), 2.79 (dt, *J* = 6.9, 2.7 Hz, 2H), 2.06 (t, *J* = 2.7 Hz, 1H); ^13^C­{^1^H} NMR (126 MHz, CDCl_3_) δ 148.4, 137.7, 133.4, 130.0, 129.6, 128.0, 119.6, 98.4, 78.8, 71.4, 67.4, 43.7, 18.5. HRMS (ESI/Q-TOF) *m*/*z*: [M + H]^+^ calcd for C_11_H_9_ClIN_4_O: 374.9510; found 374.9507.

##### 1-(But-3-yn-1-yl)-4-(3-iodo-4-(trifluoromethyl)­phenyl)-1,4-dihydro-5*H*-tetrazol-5-one (**5l**)

Yellow oil, 29% yield; ^1^H NMR (500 MHz, CDCl_3_) δ 7.83–7.78 (m, 2H), 7.35 (d, *J* = 8.4 Hz, 1H), 4.21 (t, *J* = 7.0 Hz, 2H), 2.84–2.76 (m, 2H), 2.05 (t, *J* = 2.7 Hz, 1H); ^13^C­{^1^H} NMR (126 MHz, CDCl_3_) δ 166.4, 161.38, 145.0, 143.0, 129.9, 128.0, 117.3, 71.5, 67.4, 44.0, 18.6.

##### 1-(But-3-yn-1-yl)-4-(quinolin-3-yl)-1,4-dihydro-5*H*-tetrazol-5-one (**5m**)

Colorless oil, 75% yield; FTIR (neat) ν_max_ 3209, 3015, 2969, 2165, 1727, 1435, 1365, 1228, 1159, 1078, 988, 908 cm^–1^; ^1^H NMR (500 MHz, CDCl_3_) δ 9.59 (d, *J* = 2.6 Hz, 1H), 8.79 (d, *J* = 3.5 Hz, 1H), 8.15 (d, *J* = 9.4 Hz, 1H), 7.91 (d, *J* = 8.2 Hz, 1H), 7.78–7.75 (m,1H), 7.64–7.61 (m, 1H), 4.26 (t, *J* = 7.0 Hz, 2H), 2.84 (dt, *J* = 7.0, 2.7 Hz, 2H), 2.08 (t, *J* = 2.7 Hz, 1H); ^13^C­{^1^H} NMR (126 MHz, CDCl_3_) δ 148.9, 146.9, 142.0, 130.1, 129.5, 128.2, 128.2, 127.9, 127.1, 124.3, 78.8, 71.4, 43.8, 18.6. HRMS (ESI/Q-TOF) *m*/*z*: [M + H]^+^ calcd for C_14_H_12_N_5_O: 266.1042; found 266.1039.

##### 1-(But-3-yn-1-yl)-4-nonyl-1,4-dihydro-5*H*-tetrazol-5-one (**5n**)

Colorless oil, 68% yield; FTIR (neat) ν_max_ 3290, 2995, 1601, 1599, 1532, 1456, 1389, 1356, 1294 cm^–1^; ^1^H NMR (500 MHz, CDCl_3_) δ 4.11 (t, *J* = 7.2 Hz, 2H), 3.93 (t, *J* = 7.2 Hz, 2H), 2.73 (dt, *J* = 7.0, 2.7 Hz, 2H), 2.0 (t, *J* = 2.7 Hz, 1H), 1.85–1.78 (m, 2H), 1.36–1.22 (m, 12H), 0.86 (t, *J* = 6.9 Hz, 3H); ^13^C­{^1^H} NMR (126 MHz, CDCl_3_) δ 150.6, 79.1, 71.0, 45.0, 43.4, 31.7, 29.0, 28.9, 28.4, 26.3, 22.6, 18.6, 14.0. HRMS (ESI/Q-TOF) *m*/*z*: [M + H]^+^ calcd for C_14_H_25_N_4_O: 265.2028 found 265.1434.

##### (*E*)-1-(But-3-yn-1-yl)-4-(2-(perfluorophenyl)­vinyl)-1,4-dihydro-5*H*-tetrazol-5-one (**5o**)

White solid, 71% yield; mp 87–90 °C; FTIR (neat) ν_max_ 3280, 1727, 1656, 1655, 1496, 1403, 1189, 1165, 1146, 954 cm^–1^; ^1^H NMR (500 MHz, CDCl_3_) δ 7.65 (d, *J* = 15.0 Hz, 1H), 7.30 (dd, *J* = 9.5, 15.0 Hz, 1H), 4.18 (t, *J* = 7.0 Hz, 2H), 2.77 (dt, *J* = 7.0, 2.7 Hz, 2H), 2.05 (t, *J* = 2.7 Hz, 1H); ^13^C­{^1^H} NMR (126 MHz, CDCl_3_) δ 148.2, 144.7 (d, *J* = 248.3 Hz), 141.4, 140.4–135.6 (m), 129.8, 124.8, 109.5, 104.2, 78.6, 71.4, 43.7, 18.5. HRMS (ESI/Q-TOF) *m*/*z*: [M + H]^+^ calcd for C_13_H_8_F_5_N_4_O: 331.0618; found 331.0602.

#### Compounds **6a**–**6o** were Synthesized According to General Procedure C

##### Ethyl 5-((5-Oxo-4-phenyl-4,5-dihydro-1*H*-tetrazol-1-yl)­methyl)­isoxazole-3-carboxylate (**6a**)

Colorless oil, 57% yield; ^1^H NMR (500 MHz, CDCl_3_) δ 7.86 (dt, *J* = 8.3, 1.7 Hz, 1H), 7.45–7.48 (m, 2H), 7.34–7.37 (m, 1H), 6.79 (s, 1H), 5.39 (s, 2H), 4.45–4.32 (m, 2H), 1.37 (t, *J* = 7.1 Hz, 3H); FTIR (neat) ν_max_ 3289, 2161, 2127, 1745, 1597, 1562, 1493, 1292, 1256, 1174, 1115, 805,769 cm^–1^; ^13^C­{^1^H} NMR (126 MHz, CDCl_3_) δ 166.1, 160.3, 159.3, 156.8, 148.5, 134.2, 129.5, 128.2, 119.4, 104.9, 62.5, 39.9, 14.0. HRMS (ESI/Q-TOF) *m*/*z*: [M + H]^+^ calcd for C_14_H_14_N_5_O_4_: 316.1046; found 316.1049.

##### Ethyl 5-(2-(5-Oxo-4-phenyl-4,5-dihydro-1*H*-tetrazol-1-yl)­ethyl)­isoxazole-3-carboxylate (**6b**)

White solid, 61% yield; mp 86–89 °C; FTIR (neat) ν_max_ 2969, 1738, 1592, 1559, 1365, 1229, 1217 cm^–1^; ^1^H NMR (400 MHz, CDCl_3_) δ 7.91 (d, *J* = 7.6 Hz, 2H), 7.54–7.46 (m, 2H), 7.38 (t, *J* = 7.5 Hz, 1H), 6.58 (s, 1H), 4.48–4.37 (m, 2H), 3.46 (t, *J* = 7.0 Hz, 2H), 1.40 (t, *J* = 7.2 Hz, 3H); ^13^C­{^1^H} NMR (101 MHz, CDCl_3_) δ 170.00, 159.72, 156.62, 148.74, 134.43, 129.43, 127.92, 119.31, 103.17, 62.23, 42.44, 25.84, 14.11. HRMS (ESI/Q-TOF) *m*/*z*: [M + H]^+^ calcd for C_15_H_16_N_5_O_4_: 330.1203; found 330.1189.

##### Ethyl 5-(3-(5-Oxo-4-phenyl-4,5-dihydro-1*H*-tetrazol-1-yl)­propyl)­isoxazole-3-carboxylate (**6c**)

White solid, 66% yield; mp 77–79 °C; FTIR (neat) ν_max_ 3130, 2983, 1726, 1708, 1596, 1460, 1388, 1250, 1168, 1062, 989, 934, 824 cm^–1^. ^1^H NMR (500 MHz, CDCl_3_) δ 7.92–7.91 (m, 2H), 7.51–7.48 (m, 2H), 7.37 (app t, *J* = 7.5 Hz, 1H),6.52 (s, 1H), 4.41 (q, *J* = 7.1 Hz, 2H), 4.11 (t, *J* = 6.8 Hz, 2H), 2.96 (t, *J* = 7.5 Hz, 2H), 2.35–2.30 (m, 2H), 1.39 (t, *J* = 7.1 Hz, 3H); ^13^C­{^1^H} NMR (126 MHz, CDCl_3_) δ 174.1, 160.0, 156.5, 149.0, 134.5, 129.4, 127.8, 119.3, 102.3, 62.1, 43.9, 26.2, 23.8, 14.1. HRMS (ESI/Q-TOF) *m*/*z*: [M + H]^+^ calcd for C_16_H_18_N_5_O_4_: 344.1359; found 344.1356.

##### Ethyl 5-(4-(5-Oxo-4-phenyl-4,5-dihydro-1*H*-tetrazol-1-yl)­butyl)­isoxazole-3-carboxylate (**6d**)

White solid, 50% yield; mp 62–64 °C; FTIR (neat) ν_max_ 3137, 2990, 1708, 1504, 1459, 1386, 1271, 1167, 1099, 1056, 988, 830 cm^–1^; ^1^H NMR (500 MHz, CDCl_3_) δ 7.92–7.90 (m, 2H), 7.49–7.46 (m, 2H), 7.36–7.33 (m, 1H), 6.42 (s, 1H), 4.41 (q, *J* = 7.2 Hz, 2H), 4.05 (t, *J* = 6.9 Hz, 2H), 2.88 (t, *J* = 7.6 Hz, 2H), 1.98–1.92 (m, 2H), 1.86–1.79 (m, 2H), 1.38 (t, *J* = 7.2 Hz, 3H); ^13^C­{^1^H} NMR (126 MHz, CDCl_3_) δ 174.3, 160.0, 156.4, 150.8, 149.0, 134.6, 127.7, 119.2, 101.8, 62.2, 62.1, 44.3, 27.7, 26.0, 24.3, 15.6, 14.2. HRMS (ESI/Q-TOF) *m*/*z*: [M + H]^+^ calcd for C_17_H_20_N_5_O_4_: 358.1515; found 358.1510.

##### Ethyl 5-(2-(4-(4-Bromophenyl)-5-oxo-4,5-dihydro-1*H*-tetrazol-1-yl)­ethyl)­isoxazole-3-carboxylate (**6e**)

White solid, 61% yield; mp 102–104 °C; FTIR (neat) ν_max_ 3138, 3024, 2985, 1724, 1604, 1484, 1373, 1281, 1178, 1095, 986, 859 cm^–1^; ^1^H NMR (500 MHz, CDCl_3_) δ 7.84 (app dt, *J* = 9.0, 2.1 Hz, 2H), 7.62 (app dt, *J* = 9.0, 2.2 Hz, 2H), 6.58 (s, 1H), 4.45–4.39 (m,4H), 3.45 (t, *J* = 7.0 Hz, 2H), 1.40 (t, *J* = 7.1 Hz, 3H); ^13^C­{^1^H} NMR (126 MHz, CDCl_3_) δ 169.9, 159.7, 156.6, 148.5, 133.5, 132.6, 121.4, 120.6, 103.2, 62.3, 42.5, 25.8, 14.1. HRMS (ESI/Q-TOF) *m*/*z*: [M + H]^+^ calcd for C_15_H_15_BrN_5_O_4_: 408.0307; found 408.0301.

##### Ethyl 5-(2-(5-Oxo-4-(*m*-tolyl)-4,5-dihydro-1*H*-tetrazol-1-yl)­ethyl)­isoxazole-3-carboxylate (**6f**)

Colorless oil, 69% yield; FTIR (neat) ν_max_ 3126, 2989, 1670, 1539, 1433, 1368, 1270, 1135, 1198, 980, 889 cm^–1^; ^1^H NMR (500 MHz, CDCl_3_) δ 7.72–7.68 (m, 2H), 7.37 (t, *J* = 7.8 Hz, 1H), 7.19 (d, *J* = 6.0 Hz, 1H), 6.58 (s, 1H), 4.44–4.39 (m,4H), 3.45 (t, *J* = 7.0 Hz, 2H), 2.42 (s, 3H), 1.40 (t, *J* = 7.1 Hz, 3H); ^13^C­{^1^H} NMR (126 MHz, CDCl_3_) δ 170.0, 159.7, 156.6, 148.8, 139.6, 134.3, 129.2, 128.7, 119.9, 116.5, 103.2, 62.2, 42.4, 25.8, 21.5, 14.1. HRMS (ESI/Q-TOF) *m*/*z*: [M + H]^+^ calcd for C_16_H_18_N_5_O_4_: 344.1359; found 344.1356.

##### Ethyl 5-(2-(4-(3-fluorophenyl)-5-oxo-4,5-dihydro-1*H*-tetrazol-1-yl)­ethyl)­isoxazole-3-carboxylate (**6g**)

White solid, 85% yield; mp 82–84 °C; FTIR (neat) ν_max_ 3138, 2996, 1647, 1558, 1424, 1380, 1276, 1154, 1065, 992, 884 cm^–1^; ^1^H NMR (400 MHz, CDCl_3_) δ 7.77–7.71 (m, 2H), 7.48–7.43 (m, 1H), 7.06 (app.dt, *J* = 9.3, 2.6 Hz, 1H), 6.57 (s, 1H), 4.43–4.39 (m, 4H), 3.44 (t, *J* = 7.8 Hz, 2H), 1.39 (t, *J* = 7.1 Hz, 3H); ^13^C­{^1^H} NMR (110 MHz, CDCl_3_) δ 169.9, 163.8, 161.9, 159.7, 156.6, 148.4, 135.6 (d, *J* = 10.6 Hz), 130.9 (d, *J* = 9.1 Hz), 114.7 (d, *J* = 21.2 Hz), 114.3 (d, *J* = 3.5 Hz), 106.6 (d, *J* = 27.2 Hz), 103.2, 62.2, 42.5, 25.8, 14.1. HRMS (ESI/Q-TOF) *m*/*z*: [M + H]^+^ calcd for C_15_H_15_FN_5_O_4_: 348.1108; found 348.1111.

##### Ethyl 5-(2-(4-(2-Iodophenyl)-5-oxo-4,5-dihydro-1*H*-tetrazol-1-yl)­ethyl)­isoxazole-3-carboxylate (**6h**)

Colorless oil, 56% yield; FTIR (neat) ν_max_ 2997, 1725, 1560, 1476, 1222, 1095, 1017, 730 cm^–1^; ^1^H NMR (500 MHz, CDCl_3_) δ 7.98 (dd, *J* = 8.0, 1.4 Hz, 1H), 7.50 (td, *J* = 7.7, 1.4 Hz, 1H), 7.40 (dd, *J* = 7.9, 1.6 Hz, 1H), 7.28–7.20 (m, 1H), 6.60 (s, 1H), 4.46–4.38 (m, 4H), 3.51 (t, *J* = 6.9 Hz, 2H), 1.40 (t, *J* = 7.1 Hz, 3H); ^13^C­{^1^H} NMR (126 MHz, CDCl_3_) δ 170.0, 159.8, 156.6, 149.0, 140.3, 135.6, 132.1, 129.5, 129.0, 103.5, 96.2, 62.3, 42.7, 25.8, 14.1. HRMS (ESI/Q-TOF) *m*/*z*: [M + H]^+^ calcd for C_15_H_15_IN_5_O_4_: 456.0169; found 456.0174.

##### Ethyl 5-(2-(4-(2-Fluorophenyl)-5-oxo-4,5-dihydro-1*H*-tetrazol-1-yl)­ethyl)­isoxazole-3-carboxylate (**6i**)

White solid, 62% yield; mp 87–91 °C; FTIR (neat) ν_max_ 3018, 2993, 1720, 1613, 1587, 1509, 1469, 1231, 1188, 1095, 782 cm^–1^; ^1^H NMR (400 MHz, CDCl_3_) δ 7.55–7.43 (m, 2H), 7.33–7.23 (m, 2H), 6.57 (s, 1H), 4.46–4.36 (m, 4H), 3.45 (t, *J* = 7.0 Hz, 2H), 1.39 (t, *J* = 7.1 Hz, 3H); ^13^C­{^1^H} NMR (110 MHz, CDCl_3_) δ 170.0, 159.7, 158.6, 156.6, 155.1, 149.2, 131.7 (d, *J* = 7.8 Hz), 127.2, 124.9 (d, *J* = 4.0 Hz), 121.0 (d, *J* = 11.9 Hz),117.2 (d, *J* = 19.0 Hz), 103.2, 62.2, 42.7, 25.8, 14.1. HRMS (ESI/Q-TOF) *m*/*z*: [M + H]^+^ calcd for C_15_H_15_FN_5_O_4_: 348.1108; found 348.1100.

##### Ethyl 5-(2-(4-(4-Fluoro-3-iodophenyl)-5-oxo-4,5-dihydro-1*H*-tetrazol-1-yl)­ethyl)­isoxazole-3-carboxylate (**6j**)

White solid, 51% yield; mp 104–107 °C; FTIR (neat) ν_max_ 3152, 3098, 2999, 1712, 1604, 1593, 1567, 1517, 1487, 1470, 1460 cm^–1^; ^1^H NMR (500 MHz, CDCl_3_) δ 8.34 (dd, *J* = 5.3, 2.6 Hz, 1H), 7.93 (ddd, *J* = 9.0, 4.3, 2.7 Hz, 1H), 7.18 (dd, *J* = 9.0, 7.2 Hz, 1H), 6.58 (s, 1H), 4.46–4.39 (m, 4H), 3.45 (t, *J* = 6.6 Hz, 2H), 1.41 (t, *J* = 7.2 Hz, 3H); ^13^C­{^1^H} NMR (126 MHz, CDCl3) δ 169.8, 160.8 (d, *J* = 273.4 Hz), 156.6, 148.4, 131.3, 130.0, 120.9 (d, *J* = 7.9 Hz), 116.1 (d, *J* = 25.9 Hz), 103.2, 62.3, 42.6, 25.8, 14.1. HRMS (ESI/Q-TOF) *m*/*z*: [M + H]^+^ calcd for C_15_H_14_FIN_5_O_4_: 474.0075; found 474.0073.

##### Ethyl 5-(2-(4-(4-Chloro-3-iodophenyl)-5-oxo-4,5-dihydro-1*H*-tetrazol-1-yl)­ethyl)­isoxazole-3-carboxylate (**6k**)

White solid, 65% yield; mp 110–112 °C; FTIR (neat) ν_max_ 3195, 3034, 2993, 1723, 1592, 1487, 1378, 1233, 1165, 1082, 994, 968, 872 cm^–1^; ^1^H NMR (500 MHz, CDCl_3_) δ 8.43 (t, *J* = 2.5 Hz, 1H), 7.91 (dt, *J* = 8.8, 2.5 Hz, 1H), 7.53 (dd, *J* = 8.8, 2.3 Hz, 1H), 6.57 (s, 1H), 4.43–4.38 (comp m, 4H), 3.44 (dt, *J* = 6.9, 2.8 Hz, 2H), 1.39 (app dt, *J* = 7.2, 2.2 Hz, 3H); ^13^C­{^1^H} NMR (126 MHz, CDCl_3_) δ 169.8, 159.7, 156.6, 148.3, 137.9, 133.2, 130.1, 129.7, 119.7, 103.2, 98.4, 62.3, 42.6, 25.8, 14.1. HRMS (ESI/Q-TOF) *m*/*z*: [M + H]^+^ calcd for C_15_H_14_ClIN_5_O_4_: 489.9779; found 489.9776.

##### Ethyl 5-(2-(4-(3-Iodo-4-(trifluoromethyl)­phenyl)-5-oxo-4,5-dihydro-1*H*-tetrazol-1-yl)­ethyl)­isoxazole-3-carboxylate (**6l**)

Yellow oil, 23% yield; ^1^H NMR (500 MHz, CDCl_3_) δ 7.75 (t, *J* = 9.7 Hz, 2H), 7.33 (d, *J* = 7.9 Hz, 1H), 6.58 (s, 1H), 4.46–4.39 (m, 4H), 3.45 (t, *J* = 6.6 Hz, 2H), 1.42–1.38 (m, 3H); ^13^C­{^1^H} NMR (126 MHz, CDCl_3_) δ 169.7, 159.7, 156.6, 150.74, 145.3, 131.2, 127.4, 117.4, 103.3, 62.3, 42.7, 25.7, 14.1; HRMS (ESI/Q-TOF) *m*/*z*: [M + H]^+^ calcd for C_16_H_14_F_3_IN_5_O_4_: 524.0043; found 524.0040.

##### Ethyl 5-(2-(5-Oxo-4-(quinolin-3-yl)-4,5-dihydro-1*H*-tetrazol-1-yl)­ethyl)­isoxazole-3-carboxylate (**6m**)

White solid, 53% yield; mp 110–112 °C; FTIR (neat) ν_max_ 3153, 3077, 2984, 2165, 1728, 1472, 1370, 1239, 1153, 1022, 964, 853 cm^–1^; ^1^H NMR (500 MHz, CDCl_3_) δ 9.48 (d, *J* = 2.6 Hz, 1H), 8.77 (d, *J* = 2.6 Hz, 1H), 8.16 (d, *J* = 8.5 Hz, 1H), 7.92 (d, *J* = 8.2 Hz, 1H), 7.80–7.76 (m, 1H), 7.65–7.62 (m, 1H), 6.61 (s, 1H), 4.48–4.40 (m, 4H), 3.49 (t, *J* = 7.1 Hz, 2H), 1.40 (t, *J* = 7.1 Hz, 3H); ^13^C­{^1^H} NMR (126 MHz, CDCl_3_) δ 169.8, 159.7, 156.7, 148.8, 146.9, 141.9, 130.2, 129.5, 128.2, 128.0, 127.1, 124.5, 103.3, 62.3, 42.7, 25.8, 14.1. HRMS (ESI/Q-TOF) *m*/*z*: [M + H]^+^ calcd for C_18_H_17_N_6_O_4_: 381.1311; found 381.1307.

##### Ethyl 5-(2-(4-Nonyl-5-oxo-4,5-dihydro-1*H*-tetrazol-1-yl)­ethyl)­isoxazole-3-carboxylate (**6n**)

Colorless oil, 47% yield; FTIR (neat) ν_max_ 2980, 2926, 2856, 1723, 1650, 1597, 1564, 1519, 1462, 1338 cm^–1^; ^1^H NMR (400 MHz, CDCl_3_) δ 6.52 (s, 1H), 4.41 (q, *J* = 7.1 Hz, 2H), 4.31 (t, *J* = 7.0 Hz, 2H), 4.21 (q, *J* = 7.1 Hz, 2H) 3.91 (t, *J* = 7.2 Hz, 2H), 3.38 (t, *J* = 7.0 Hz, 2H), 1.39 (t, *J* = 7.2 Hz, 3H), 1.33–1.20 (m, 10H), 0.86 (t, *J* = 5.9 Hz, 3H); ^13^C­{^1^H} NMR (101 MHz, CDCl_3_) δ 170.2, 159.7, 150.8, 150.5, 103.1, 62.3, 62.2, 45.1, 42.3, 31.7, 29.0, 28.4, 26.3, 25.9, 22.6, 14.2, 14.1. HRMS (ESI/Q-TOF) *m*/*z*: [M + H]^+^ calcd for C_18_H_30_N_5_O_4_: 380.2292; found 380.2222.

##### Ethyl (*E*)-5-(2-(5-oxo-4-(2-(perfluorophenyl)­vinyl)-4,5-dihydro-1*H*-tetrazol-1-yl)­ethyl)­isoxazole-3-carboxylate (**6o**)

White solid, 65% yield; mp 94–97 °C; FTIR (neat) ν_max_ 3142, 2993, 1725, 1651, 1595, 1521, 1496, 1411, 1141, 1100 cm^–1^; ^1^H NMR (500 MHz, CDCl_3_) δ 7.65 (d, *J* = 15.1 Hz, 1H), 7.30 (d, *J* = 15.1 Hz, 1H), 6.57 (s, 1H), 4.47–4.36 (m, 4H), 3.44 (t, *J* = 6.9 Hz, 2H), 1.41 (t, *J* = 7.1 Hz, 3H); ^13^C NMR (126 MHz, CDCl_3_) δ 169.7, 159.7, 156.6, 148.1, 124.6, 104.6, 103.3, 62.3, 42.6, 25.7, 14.1. HRMS (ESI/Q-TOF) *m*/*z*: [M + H]^+^ calcd for C_17_H_13_F_5_N_5_O_4_: 446.0888; found 446.0880.

##### Methyl 3-(5-Oxo-4-phenyl-4,5-dihydro-1*H*-tetrazol-1-yl)­propanoate (**7**)

Colorless oil, 64% yield; FTIR (neat) ν_max_ 2953, 1721, 1649, 1502, 1500, 1438, 1386, 1355, 1154 cm^–1^; ^1^H NMR (500 MHz, CDCl_3_) δ 7.97–7.87 (m, 2H), 7.55–7.42 (m, 2H), 7.40–7.30 (m, 1H), 4.33 (t, *J* = 7.0 Hz, 2H), 3.72 (s, 3H), 2.94 (t, *J* = 7.0 Hz, 2H); ^13^C­{^1^H} NMR (126 MHz, CDCl_3_) δ 170.1, 149.3, 135.4, 129.2, 128.4, 119.6, 52.7, 41.8, 33.1. HRMS (ESI/Q-TOF) *m*/*z*: [M + H]^+^ calcd for C_11_H_13_N_4_O_3_: 249.0988; found 249.0986.

##### Ethyl 5-(2-(5-Oxo-4-phenyl-4,5-dihydro-1*H*-tetrazol-1-yl)­ethyl)-1,3,4-thiadiazole-2-carboxylate (**9**)

Step 1: An oven-dried round-bottom flask (25 mL) equipped with a magnetic stirring bar and reflux condenser was sequentially charged with **7** (496 mg, 2.0 mmol, 1 equiv) in EtOH (10 mL, 0.2 M) and hydrazine monohydrate (390 mg, 8.0 mmol, ρ = 1.029 g/mL, 4.0 equiv). The mixture was then heated at reflux for 12 h, cooled to ambient temperature (25 °C), and concentrated *in vacuo*. The remaining residue was taken up in EtOAc (20 mL) and washed with H_2_O (3 × 5 mL). The organic phase was separated, dried (Na_2_SO_4_), and concentrated *in vacuo*. The residue **8** was used in Step 2 without further purification.

Step 2: An oven-dried reaction vial (25 mL) was charged with a magnetic stirring bar, and the residue (**8**) generated in Step 1 (248 mg, 1.0 mmol). The vial was sealed with a septum cap, evacuated, backfilled with N_2_ (three cycles), and cooled to 0 °C. Anhydrous DCM (5 mL) and pyridine (2.0 mmol, 2.0 equiv) were sequentially added via syringe. Ethyl chlorooxoacetate (168 μL, 1.5 mmol, ρ = 1.22 g/mL, 1.5 equiv) was added via syringe, and the mixture stirred at ambient temperature (25 °C) for 14 h. The reaction was then diluted with DCM (20 mL), washed with aqueous HCl (3 × 2 mL, 1.0 M), dried (Na_2_SO_4_), and concentrated *in vacuo*. The residue was used in Step 3 without further purification.

Step 3. The residue generated in Step 2 was taken up in PhMe (5 mL), treated with Lawesson’s reagent (202 mg, 0.5 mmol, 0.5 equiv), and heated at 75 °C for 3 h. After cooling to ambient temperature (25 °C), the reaction was quenched with H_2_O and extracted with EtOAc (3 × 10 mL). The combined extracts were dried (Na_2_SO_4_), concentrated *in vacuo*, and the remaining residue purified by column chromatography on silica gel to provide **9**: white solid, 29% yield (3 steps): mp 80–83 °C; FTIR (neat) ν_max_ 2985, 1730, 1647, 1595, 1459, 1384, 1261, 1173, 1021, 967, 859 cm^–1^; ^1^H NMR (500 MHz, CDCl_3_) δ 7.91–7.89 (m, 2H), 7.52–7.48 (m, 2H), 7.38 (app tt, *J* = 7.4, 1.2 Hz, 1H), 4.57 (t, *J* = 7.0 Hz, 2H), 4.51 (q, *J* = 7.1 Hz, 2H), 3.82–3.78 (m, 2H), 1.45 (t, *J* = 7.1 Hz, 3H); ^13^C­{^1^H} NMR (126 MHz, CDCl_3_) δ 169.2, 161.1, 158.4, 148.8, 134.4, 129.4, 128.0, 119.4, 63.5, 43.7, 28.9, 14.1. HRMS (ESI/Q-TOF) *m*/*z*: [M + H]^+^ calcd for C_14_H_15_N_6_O_3_S: 347.0926; found 347.0924.

##### 1-(2-Azidoethyl)-4-phenyl-1,4-dihydro-5*H*-tetrazol-5-one (**10**)

Colorless oil, 62% yield; FTIR (neat) ν_max_ 3050, 2995, 2092, 1710, 1650, 1465, 1390, 1150 cm^–1^; ^1^H NMR (500 MHz, CDCl_3_) δ 7.94 (d, *J* = 7.5 Hz, 2H), 7.51 (t, *J* = 7.5 Hz, 2H), 7.39 (t, *J* = 7.4 Hz, 1H), 4.22 (t, *J* = 5.7 Hz, 2H), 3.80 (t, *J* = 6.0 Hz, 2H); ^13^C­{^1^H} NMR (126 MHz, CDCl_3_) δ 129.4, 127.9, 119.4, 48.7, 44.2. HRMS (ESI/Q-TOF) *m*/*z*: [M + H]^+^ calcd for C_9_H_10_N_7_O: 232.0947; found 232.0944.

##### Methyl 1-(2-(5-Oxo-4-phenyl-4,5-dihydro-1*H*-tetrazol-1-yl)­ethyl)-1*H*-1,2,3-triazole-4-carboxylate (**11**)

To a solution of **10** (462 mg, 2.0 mmol, 1 equiv) in *t-*BuOH (10.0 mL) and H_2_O (5.0 mL) was added methyl propiolate (252.2 mg, 270 μL, ρ = 0.945 g/mL 3.0 mmol, 1.3 equiv), CuSO_4_·5H_2_O (8 mg, 0.05 mmol, 2.5 mol %), and ascorbic acid (44 mg, 0.25 mmol, 0.15 equiv). The mixture was stirred at ambient temperature (25 °C) for 4 h then concentrated *in vacuo* to remove *t*-BuOH. The residue was partitioned between H_2_O (20 mL) and EtOAc (20 mL), the phases separated, and the aqueous phase extracted with EtOAc (3 × 10 mL). The combined extracts were dried (MgSO_4_), concentrated *in vacuo*, and the residue purified by flash chromatography on silica gel (hexanes/EtOAc, 5:1) to provide **11**: white solid, 59% yield; mp 161–164 °C; FTIR (neat) ν_max_ 3126, 1728, 1595, 1562, 1544, 1465, 1455, 1241, 1050, 1030 cm^–1^; ^1^H NMR (500 MHz, DMSO-*d*_6_) δ 8.87 (s, 1H), 7.79 (dd, *J* = 8.6, 1.2 Hz, 1H), 7.56 (dd, *J* = 8.6, 7.4 Hz, 2H), 7.46–7.40 (m, 1H), 4.86 (t, *J* = 5.5 Hz, 2H), 4.53 (t, *J* = 5.6 Hz, 2H), 3.81 (s, 3H); ^13^C­{^1^H} NMR (126 MHz, DMSO-*d*_6_) δ 161.1, 148.9, 139.1, 134.5, 130.3, 130.1, 128.4, 119.8, 52.3, 48.5, 44.9, 40.5. HRMS (ESI/Q-TOF) *m*/*z*: [M + H]^+^ calcd for C_13_H_14_N_7_O_3_: 316.1158; found 316.1160.

#### Compounds **3a**-**3q** were Synthesized According to General Procedure D or E

##### *N*-Hydroxy-5-((5-oxo-4-phenyl-4,5-dihydro-1*H*-tetrazol-1-yl)­methyl)­isoxazole-3-carboxamide (**3a**)

White solid, 70% yield; mp 185–187 °C; FTIR (neat) ν_max_ 3232, 3093, 2969, 1736, 1651, 1508, 1468, 1409, 1373, 1216, 1160, 1028, 995, 847 cm-1; ^1^H NMR (500 MHz, DMSO-*d*_6_) δ 7.84 (d, *J* = 7.7 Hz, 2H), 7.57 (t, *J* = 8.0 Hz, 2H), 7.44 (t, *J* = 7.5 Hz, 1H), 6.78 (s, 1H), 5.49 (s, 2H).; ^13^C­{^1^H} NMR (126 MHz, DMSO-*d*_6_) δ 166.03, 159.42, 156.59, 152.45, 148.64, 134.52, 130.01, 128.52, 120.27, 103.71. HRMS (ESI/Q-TOF) *m*/*z*: [M + H]^+^ calcd for C_12_H_11_N_6_O_4_: 303.0842; found 303.0845. HPLC *t*_R_ = 13.75 min, purity 99.0% (254 nm).

##### *N*-Hydroxy-5-(2-(5-oxo-4-phenyl-4,5-dihydro-1*H*-tetrazol-1-yl)­ethyl)­isoxazole-3-carboxamide (**3b**)

White solid, 61% yield; mp 179–181 °C; FTIR (neat) ν_max_ 3177, 1705, 1645, 1591, 1498, 1456, 1397, 1207, 868 cm^–1^; ^1^H NMR (500 MHz, CDCl_3_) δ 7.80 (d, *J* = 8.3 Hz, 2H), 7.60 (t, *J* = 7.8 Hz, 2H), 7.39 (t, *J* = 7.3 Hz, 1H), 6.67 (s, 1H), 4.39 (t, *J* = 6.7 Hz, 2H), 3.40 (t, *J* = 6.7 Hz, 2H); ^13^C­{^1^H} NMR (126 MHz, CDCl_3_) δ 171.1, 158.0, 156.8, 156.4, 148.9, 134.6, 130.0, 128.4, 120.0, 102.4, 42.8, 25.6. HRMS (ESI/Q-TOF) *m*/*z*: [M + H]^+^ calcd for C_13_H_13_N_6_O_4_: 317.0998; found 317.0990. HPLC *t*_R_ = 13.75 min, purity 99.0% (254 nm).

##### *N*-Hydroxy-5-(3-(5-oxo-4-phenyl-4,5-dihydro-1*H*-tetrazol-1-yl)­propyl)­isoxazole-3-carboxamide (**3c**)

White solid, 76% yield; mp 154–156 °C; FTIR (neat) ν_max_ 3229, 2946, 1718, 1662, 1504, 1451, 1422, 1148, 1071, 1058, 988, 930, 801 cm^–1^; ^1^H NMR (500 MHz, DMSO-*d*_6_) δ 9.56 (s, 1H), 7.84 (d, *J* = 7.6 Hz, 2H), 7.56 (t, *J* = 7.7 Hz, 2H), 7.43 (t, *J* = 7.4 Hz, 1H), 6.46 (s, 1H), 4.04 (t, *J* = 6.8 Hz, 2H), 2.89 (t, *J* = 7.5 Hz, 2H), 2.19–2.14 (m, 2H); ^13^C­{^1^H} NMR (126 MHz, DMSO-*d*_6_) δ172.7, 159.2, 157.2, 149.1, 134.7, 130.0, 128.3, 120.1, 101.0, 44.2, 26.0, 23.4. HRMS (ESI/Q-TOF) *m*/*z*: [M + H]^+^ calcd for C_14_H_15_N_6_O_4_: 331.1155; found 331.1154. HPLC *t*_R_ = 14.53 min, purity 98.5% (254 nm).

##### *N*-Hydroxy-5-(4-(5-oxo-4-phenyl-4,5-dihydro-1*H*-tetrazol-1-yl)­butyl)­isoxazole-3-carboxamide (**3d**)

White solid, 77% yield; mp 142–145 °C; FTIR (neat) ν_max_ 3213, 3090, 2985, 1724, 1667, 1595, 1462, 1231, 1145, 1030, 918, 761 cm^–1^; ^1^H NMR (500 MHz, DMSO-*d*_6_) δ 9.60 (s, 1H), 7.85 (d, *J* = 7.7 Hz, 2H), 7.56 (t, *J* = 7.9 Hz, 2H), 7.42 (t, *J* = 7.4 Hz, 1H), 6.45 (s, 1H), 4.01 (t, *J* = 6.8 Hz, 2H), 2.83 (t, *J* = 7.5 Hz, 2H), 1.86–1.80 (m, 2H), 1.75–1.70 (m, 2H); ^13^C­{^1^H} NMR (126 MHz, DMSO-*d*_6_) δ 173.8, 158.8, 157.1, 149.0, 134.7, 130.0, 128.3, 120.1, 100.9, 44.5, 27.6, 25.7, 24.3. HRMS (ESI/Q-TOF) *m*/*z*: [M + H]^+^ calcd for C_15_H_17_N_6_O_4_: 345.1311; found 345.1310. HPLC *t*_R_ = 14.93 min, purity 98.4% (254 nm).

##### 5-(2-(4-(4-Bromophenyl)-5-oxo-4,5-dihydro-1*H*-tetrazol-1-yl)­ethyl)-*N*-hydroxyisoxazole-3-carboxamide (**3e**)

White solid, 78% yield; mp 183–185 °C; FTIR (neat) ν_max_ 3243, 3115, 1709, 1650, 1492, 1416, 1387, 1282, 1160, 1089, 984, 819 cm^–1^; ^1^H NMR (500 MHz, DMSO-*d*_6_) δ 9.18 (br s, 1H), 7.82 (app dt, *J* = 9.1, 2.3 Hz, 2H), 7.77 (app dt, *J* = 9.1, 2.3 Hz, 2H), 6.53 (s, 1H), 4.33 (t, *J* = 6.7 Hz, 2H), 3.34 (t, *J* = 6.7 Hz, 2H); ^13^C­{^1^H} NMR (126 MHz, DMSO-*d*_6_) δ 169.7, 159.4, 157.0, 148.7, 133.9, 133.0, 121.7, 120.8, 102.1, 42.9, 25.5. HRMS (ESI/Q-TOF) *m*/*z*: [M + H]^+^ calcd for C_13_H_12_BrN_6_O_4_: 395.0103; found 395.0092. HPLC *t*_R_ = 13.91 min, purity 99.5% (254 nm).

##### *N*-Hydroxy-5-(2-(5-oxo-4-(*m*-tolyl)-4,5-dihydro-1*H*-tetrazol-1-yl)­ethyl)­isoxazole-3-carboxamide (**3f**)

White solid, 73% yield; mp 128–130 °C; FTIR (neat) ν_max_ 3225, 3116, 3061, 1734, 1649, 1563, 1455, 1381, 1291, 1158, 1088, 990, cm^–1^; ^1^H NMR (500 MHz, DMSO-*d*_6_) δ 8.59 (br s, 1H), 7.65–7.61 (m, 2H), 7.44 (t, *J* = 7.8 Hz, 1H), 7.25 (d, *J* = 7.9 Hz, 1H), 6.53 (s, 1H), 4.33 (t, *J* = 6.7 Hz, 2H), 3.35 (t, *J* = 6.7 Hz, 2H), 2.37 (s, 3H); ^13^C­{^1^H} NMR (126 MHz, DMSO-*d*_6_) δ 170.0, 159.1, 156.9, 148.9, 139.7, 134.5, 129.9, 129.1, 120.4, 117.1, 102.1, 42.8, 25.5, 21.4. HRMS (ESI/Q-TOF) *m*/*z*: [M + H]^+^ calcd for C_14_H_15_N_6_O_4_: 331.1155; found 331.1149. HPLC *t*_R_ = 14.47 min, purity 99.0% (254 nm).

##### 5-(2-(4-(3-Fluorophenyl)-5-oxo-4,5-dihydro-1*H*-tetrazol-1-yl)­ethyl)-*N*-hydroxyisoxazole-3-carboxamide (**3g**)

White solid, 69% yield; mp 165–167 °C; FTIR (neat) ν_max_ 3218, 3148, 3082, 1727, 1669, 1564, 1454, 1380, 1296, 1159, 1068, 985, cm^–1^; ^1^H NMR (500 MHz, DMSO-*d*_6_) δ 9.90 (br s, 1H), 7.74–7.70 (m, 2H), 7.65–7.60 (m, 1H), 7.29 (app dt, *J* = 8.7, 2.6 Hz, 1H), 6.57 (s, 1H), 4.34 (t, *J* = 6.8 Hz, 2H), 3.35 (t, *J* = 6.7 Hz, 2H); ^13^C­{^1^H} NMR (126 MHz, DMSO-*d*_6_) δ 170.1, 163.5, 161.6, 159.0, 156.8, 148.7, 135.9 (d, *J* = 10.7 Hz), 132.1 (d, *J* = 9.25 Hz), 115.2 (d, *J* = 21.0 Hz), 106.8 (d, *J* = 26.8 Hz), 102.2, 42.9, 25.5. HRMS (ESI/Q-TOF) *m*/*z*: [M + H]^+^ calcd for C_13_H_12_FN_6_O_4_: 335.0904; found 335.0901. HPLC *t*_R_ = 14.27 min, purity 96.9% (254 nm).

##### *N*-Hydroxy-5-(2-(4-(2-iodophenyl)-5-oxo-4,5-dihydro-1*H*-tetrazol-1-yl)­ethyl)­isoxazole-3-carboxamide (**3h**)

White solid, 43% yield; mp 167–169 °C; FTIR (neat) ν_max_ 3025, 3015, 2970, 1738, 1728, 1670, 1649, 1508, 1475, 1436 cm^–1^; ^1^H NMR (500 MHz, CD_3_OD) δ 8.06 (dd, *J* = 7.9, 1.3 Hz, 1H), 7.59 (td, *J* = 7.7, 1.4 Hz, 1H), 7.50 (dd, *J* = 7.8, 1.6 Hz, 1H), 7.33 (td, *J* = 7.7, 1.6 Hz, 1H), 6.62 (s, 1H), 4.44 (t, *J* = 6.5 Hz, 2H), 3.49 (t, *J* = 6.5 Hz, 2H); ^13^C­{^1^H} NMR (126 MHz, CD_3_OD) δ 170.4, 157.2, 149.5, 142.2, 139.9, 135.7, 132.1, 129.3, 129.0, 102.1, 96.2, 42.6, 25.2. HRMS (ESI/Q-TOF) *m*/*z*: [M + H]^+^ calcd for C_13_H_12_IN_6_O_4_: 442.9965; found 442.9962. HPLC *t*_R_ = 15.06 min, purity 95.6% (254 nm)

##### 5-(2-(4-(2-Fluorophenyl)-5-oxo-4,5-dihydro-1*H*-tetrazol-1-yl)­ethyl)-*N*-hydroxyisoxazole-3-carboxamide (**3i**)

White solid, 57% yield; mp 167–170 °C; FTIR (neat) ν_max_ 3118, 1718, 1643, 1509, 1458, 1426, 1411, 1390, 1270, 1234, 1158,875 cm^–1^; ^1^H NMR (500 MHz, CD_3_OD) δ 7.63–7.55 (m, 2H), 7.43–7.35 (m, 2H), 6.61 (s, 1H), 4.42 (t, *J* = 6.6 Hz, 2H), 3.46 (t, *J* = 6.6 Hz, 2H); ^13^C­{^1^H} NMR (126 MHz, CD_3_OD) δ 170.2, 157.8 (d, *J* = 27.7 Hz) 155.5, 149.7, 131.9, 127.7, 124.8, 120.8, 116.6 (d, *J* = 19.2 Hz), 102.6, 101.4, 42.7, 25.1. HRMS (ESI/Q-TOF) *m*/*z*: [M + H]^+^ calcd for C_13_H_12_FN_6_O_4_: 335.0904; found 335.0906. HPLC *t*_R_ = 13.40 min, purity 99.0% (254 nm).

##### 5-(2-(4-(4-Fluoro-3-iodophenyl)-5-oxo-4,5-dihydro-1*H*-tetrazol-1-yl)­ethyl)-*N*-hydroxyisoxazole-3-carboxamide (**3j**)

White solid, 77% yield; mp 167–170 °C; FTIR (neat) ν_max_ 3335, 3190, 1725, 1621, 1615 1584, 1572, 1499, 1472, 879 cm^–1^; ^1^H NMR (500 MHz, DMSO-*d*_6_) δ 8.26 (dd, *J* = 5.5, 2.6 Hz, 1H), 7.89–7.83 (m, 1H), 7.46 (t, *J* = 7.8 Hz, 1H), 6.47 (s, 1H), 4.32 (t, *J* = 6.7 Hz, 2H), 3.31 (t, *J* = 6.7 Hz, 2H); ^13^C­{^1^H} NMR (126 MHz, DMSO-*d*_6_) δ 169.2, 161.8, 159.8 (d, *J* = 29.8 Hz), 157.3, 148.8, 131.8, 130.6, 122.5 (d, *J* = 8.3 Hz), 117.0 (d, *J* = 26.2 Hz), 101.9, 83.4 (d, *J* = 28.3 Hz), 42.9, 25.5. HRMS (ESI/Q-TOF) *m*/*z*: [M + H]^+^ calcd for C_13_H_11_FIN_6_O_4_: 460.9870; found 460.9870. HPLC *t*_R_ = 15.13 min, purity 97.9% (254 nm).

##### 5-(2-(4-(4-Chloro-3-iodophenyl)-5-oxo-4,5-dihydro-1*H*-tetrazol-1-yl)­ethyl)-*N*-hydroxyisoxazole-3-carboxamide (**3k**)

White solid, 73% yield; mp 110–112 °C; FTIR (neat) ν_max_ 3232, 3093, 2969, 1736, 1651, 1508, 1468, 1409, 1373, 1216, 1160, 1028, 995, 847 cm^–1^; ^1^H NMR (500 MHz, DMSO-*d*_6_): δ 8.39 (d, *J* = 2.5 Hz, 1H), 7.88 (dd, *J* = 8.7, 2.5 Hz, 1H), 7.76 (d, *J* = 8.8 Hz, 1H), 6.61 (s, 1H), 4.33 (t, *J* = 6.7 Hz, 2H), 3.35 (t, *J* = 6.6 Hz, 2H); ^13^C­{^1^H} NMR (126 MHz, DMSO-*d*_6_) δ 170.4, 158.6, 156.7, 148.7, 136.9, 133.8, 130.5, 130.4, 121.0, 102.3, 100.0, 42.9, 25.5. HRMS (ESI/Q-TOF) *m*/*z*: [M + H]^+^ calcd for C_13_H_11_ClIN_6_O_4_: 476.9575; found 476.9571. HPLC *t*_R_ = 15.71 min, purity 97.9% (254 nm).

##### *N*-Hydroxy-5-(2-(4-(3-iodo-4-(trifluoromethyl)­phenyl)-5-oxo-4,5-dihydro-1*H*-tetrazol-1-yl)­ethyl)­isoxazole-3-carboxamide (**3l**)

Yellow oil, 19% yield; FTIR (neat) ν_max_ 3293, 3112, 2865, 1664, 1511, 1324, 1027 cm^–1^; ^1^H NMR (500 MHz, DMSO-*d*_6_) δ 9.33 (br s, 1H), 8.61 (app. d, *J* = 2.1 Hz, 1H), 8.08 (d, *J* = 8.8 Hz, 1H), 7.95 (d, *J* = 8.7 Hz, 1H), 6.68 (s, 1H), 4.36 (t, *J* = 6.6 Hz, 2H), 3.38 (t, *J* = 6.6 Hz, 2H). ^13^C­{^1^H} NMR (126 MHz, DMSO-*d*_6_) δ 170.9, 158.0, 148.7, 137.9, 131.2, 130.8, 129.5, 118.4, 102.5, 93.9, 42.9, 25.5. HRMS (ESI/Q-TOF) *m*/*z*: [M + H]^+^ calcd for C_14_H_11_F_3_IN_6_O_4_: 510.9838; found 510.9820. HPLC *t*_R_ = 14.70 min, purity 95.6% (254 nm).

##### *N*-Hydroxy-5-(2-(5-oxo-4-(quinolin-3-yl)-4,5-dihydro-1*H*-tetrazol-1-yl)­ethyl)­isoxazole-3-carboxamide (**3m**)

White solid, 78% yield; mp 170–172 °C; FTIR (neat) ν_max_ 3302, 3015, 2945, 1671, 1564, 1424, 1366, 1216, 1139, 1027, 987, 864 cm^–1^; ^1^H NMR (500 MHz, DMSO-*d*_6_) δ 9.60 (br s, 1H), 9.35 (d, *J* = 2.6 Hz, 1H), 8.83 (d, *J* = 2.7 Hz, 1H), 8.16 (d, *J* = 8.2 Hz, 1H), 8.10 (d, *J* = 8.2 Hz, 1H), 7.84 (t, *J* = 6.9 Hz, 1H), 7.71 (t, *J* = 7.0 Hz, 1H), 6.63 (s, 1H), 4.40 (t, *J* = 6.7 Hz, 2H), 3.40 (t, *J* = 6.7 Hz, 2H); ^13^C­{^1^H} NMR (126 MHz, DMSO-*d*_6_) δ 170.2, 158.8, 156.8, 149.1, 146.7, 143.0, 130.8, 129.3, 129.1, 128.4, 128.3, 127.3, 125.7, 102.3, 43.0, 25.5. HRMS (ESI/Q-TOF) *m*/*z*: [M + H]^+^ calcd for C_16_H_14_N_7_O_4_: 368.1107; found 368.1103. HPLC *t*_R_ = 13.43 min, purity 96.3% (254 nm).

##### *N*-Hydroxy-5-(2-(4-nonyl-5-oxo-4,5-dihydro-1*H*-tetrazol-1-yl)­ethyl)­isoxazole-3-carboxamide (**3n**)

White solid, 83% yield; mp 165–168 °C; FTIR (neat) ν_max_ 3180, 3142, 2954, 2921, 2868, 1721, 1649, 1591, 1443 cm^–1^; ^1^H NMR (500 MHz, DMSO-*d*_6_) δ 8.45 (s, 2H), 6.45 (s, 1H), 4.24 (t, *J* = 6.7 Hz, 2H), 3.85 (t, *J* = 7.0 Hz, 2H), 3.27 (t, *J* = 6.7 Hz, 2H), 2.50–2.47 (m, 2H), 1.70–1.63 (m, 2H), 1.28–1.19 (m, 10H), 0.83 (t, *J* = 6.9 Hz, 3H); ^13^C­{^1^H} NMR (126 MHz, DMSO-*d*_6_) δ 169.8, 159.3, 157.0, 150.4, 101.9, 44.6, 42.6, 31.6, 28.9, 28.7, 28.2, 26.1, 25.5, 22.5, 14.4. HRMS (ESI/Q-TOF) *m*/*z*: [M + H]^+^ calcd for C_16_H_27_N_6_O_4_: 367.2094; found 367.2035. HPLC *t*_R_ = 18.14 min, purity 97.9% (254 nm).

##### (*E*)-*N*-Hydroxy-5-(2-(5-oxo-4-(2-(perfluorophenyl)­vinyl)-4,5-dihydro-1*H*-tetrazol-1-yl)­ethyl)­isoxazole-3-carboxamide (**3o**)

White solid, 64% yield; mp 168–171 °C; FTIR (neat) ν_max_ 3212, 2358, 2321, 2260, 1728, 1647, 1496, 1428, 1001 cm^–1^; ^1^H NMR (500 MHz, DMSO-*d*_6_) δ 9.09 (s, 2H), 7.63 (d, *J* = 15.0 Hz, 1H), 7.16 (d, *J* = 15.0 Hz, 1H), 6.50 (s, 1H), 4.31 (t, *J* = 6.7 Hz, 2H), 3.32 (t, *J* = 6.7 Hz, 2H); ^13^C­{^1^H} NMR (126 MHz, DMSO-*d*_6_) δ 170.9, 158.0, 156.4, 148.3 (d, *J* = 19.0 Hz), 145.6, 136.8, 126.2, 109.9, 109.0, 103.1, 102.4, 42.8, 25.4. HRMS (ESI/Q-TOF) *m*/*z*: [M + H]^+^ calcd for C_15_H_10_F_5_N_6_O_4_: 433.0684; found 433.0675. HPLC *t*_R_ = 16.08 min, purity 96.1% (254 nm).

##### *N*-Hydroxy-5-(2-(5-oxo-4-phenyl-4,5-dihydro-1*H*-tetrazol-1-yl)­ethyl)-1,3,4-thiadiazole-2-carboxamide (**3p**)

White solid, 63% yield; mp 178–180 °C; FTIR (neat) ν_max_ 3226, 3050, 2969, 1733, 1719, 1640, 1564, 1418, 1365, 1286, 1164, 1067, 977, 879 cm^–1^; ^1^H NMR (500 MHz, DMSO-*d*_6_) δ 8.60 (s, 2H), 7.82 (d, *J* = 9.8 Hz, 2H), 7.56 (t, *J* = 7.5 Hz, 2H), 7.43 (t, *J* = 7.5 Hz, 1H), 4.42 (t, *J* = 6.7 Hz, 2H), 3.64 (t, *J* = 6.7 Hz, 2H); ^13^C­{^1^H} NMR (126 MHz, DMSO-*d*_6_) δ 167.6, 166.8, 156.8, 148.9, 134.6, 130.1, 128.4, 119.9, 44.1, 28.5. HRMS (ESI/Q-TOF) *m*/*z*: [M + H]^+^ calcd for C_12_H_12_N_7_O_3_S: 334.0722; found 334.0720. HPLC *t*_R_ = 14.18 min, purity 99.0% (254 nm).

##### *N*-Hydroxy-1-(2-(5-oxo-4-phenyl-4,5-dihydro-1*H*-tetrazol-1-yl)­ethyl)-1*H*-1,2,3-triazole-4-carboxamide (**3q**)

White solid, 78%; mp 186–189 °C; FTIR (neat) ν_max_ 3309, 3234, 1730, 1626, 1597, 1570, 1498, 1469, 875 cm^–1^; ^1^H NMR (500 MHz, DMSO-*d*_6_) δ 8.56 (s, 1H), 7.80 (d, *J* = 7.9 Hz, 2H), 7.56 (t, *J* = 7.8 Hz, 2H), 7.43 (t, *J* = 7.42 Hz, 1H), 4.84 (t, *J* = 5.8 Hz, 2H), 4.51 (t, *J* = 5.6 Hz, 2H); ^13^C­{^1^H} NMR (126 MHz, DMSO-*d*_6_) δ158.0, 148.9, 142.3, 134.6, 130.1, 128.4, 127.1, 119.8, 48.2, 45.0. HRMS (ESI/Q-TOF) *m*/*z*: [M + H]^+^ calcd for C_12_H_13_N_8_O_3_: 317.1111; found 317.1109. HPLC *t*_R_ = 15.24 min, purity 95.9% (254 nm)

### Biology

#### Selectivity Screening

Human HDACs 1–11 were heterologously expressed in HEK293T cells, affinity-purified using Streptactin chromatography, and used to determine the potency of tested inhibitors in vitro as described previously.^86,87^ Briefly, optimized concentrations of purified HDACs (HDAC1–9, HDAC11) were preincubated with a dilution series of a given inhibitor in 384-well plates (Corning, NY, USA) in an assay buffer (50 mM HEPES, 140 mM NaCl, 10 mM KCl, pH 7.4 supplemented with 1 mg/mL bovine serum albumin (BSA), and 1 mM tris­(2-carboxyethyl) phosphine (TCEP) in the total volume of 30 μL at 37 °C for 15 min. The enzymatic reaction was started by the addition of 10 μL of Ac-GAK-Ac-AMC (HDAC 1–3, 6) or Boc-Lys (TFA)-AMC (HDAC4,5,7,9,11) substrates (both substrates at 10 μM final concentration, Bachem, Bubendorf, Switzerland) and then terminated following 30 min incubation with 25 μL of a trypsin solution (4 mg/mL stock solution). The released aminomethyl coumarine was quantified using a CLARIOstar plate reader (BMG Labtech GmbH, Ortenberg, Germany) at λ_EX_/λ_EM_ = 365/440 nm. For HDAC10 measurements, N8-acetylspermidine labeled with fluorescein (10 μM final concentration) was used as a substrate in a total volume of 50 μL. Following 30 min incubation, reactions were terminated by the addition of 5 μL of 0.5% acetic acid and centrifuged at 2000*g* at RT for 15 min. Reaction mixtures were analyzed by RP-HPLC with a Kinetex 2.6 μm XB-C18 100 Å column with a fluorescence detector set to λ_EX_/λ_EM_ = 492/516. HDAC inhibition was calculated using corresponding noninhibited reactions as a control. Inhibition data were fitted using a nonlinear regression model in the GraphPad Prism software (GraphPad Software, San Diego, CA, USA).

#### In Vitro Cytotoxicity

To determine the cytotoxicity of the tetrazolones, murine macrophage RAW264.7 cells were seeded at 25,000 cells/well in a 96-well black wall, optically clear flat-bottom tissue culture-treated plate in 80 μL of complete DMEM media (Corning) (containing 10% FBS, 1% Pen-Strep, and 1% nonessential amino acids) and allowed to attach overnight. The next day, the media was replaced with 80 μL of fresh complete DMEM containing the CellTox Green Dye Reagent (Promega) diluted 1:850. A 20 μM concentration of the test compounds was prepared in complete DMEM medium and was further serially diluted 2-fold to obtain concentrations of 10, 5, 2.5, 1.25, 0.625, 0.312, and 0.156 μM. 20 μL of each of these compound dilutions was added to the cells in respective wells and incubated at 37 °C, 5% CO_2_ overnight. The Relative Fluorescence Units were measured after 24 h with the SpectraMax iD5 (Molecular Devices) device using an excitation wavelength of 490 nM and an emission wavelength of 525 nM.

#### In Vitro HDAC Activity

The inhibitory activity of tetrazolones against class I (HDAC1,2,3, and 8) and class II (4,5,7,9,6, and 10) HDACs was measured using the HDAC-Glo kit from Promega. Briefly, RAW264.7 cells were seeded into tissue culture-treated, white, optically clear, flat-bottom 96-well plates at a cell density of 10,000 cells/well in 80 μL of complete DMEM medium and were allowed to attach overnight at 37 °C and 5% CO_2_. 2-fold serially diluted concentrations of the test compounds (20, 10, 5, 2.5, 1.25, 0.625, 0.312, and 0.156 μM) were prepared as described above. 20 μL of these preparations was added to the respective wells. After 1 h of incubation, 15 μL of the HDAC reaction reagent (Promega), containing the substrate and developer, was added to the wells. The Relative Luminescence Units (RLU) reading was collected immediately in the SpectraMax iD5 device using a kinetic protocol at 37 °C with an excitation wavelength of 578 nm.

#### Western Blot and qRT-PCR

RAW264.7 cells were treated with tetrazolones at concentrations of 0.5, 1, 2.5, 5, and 10 μM for 24 h. Cells were lysed in RIPA buffer (Thermo Scientific), supplemented with protease and phosphatase inhibitors, followed by protein quantification using a BCA kit (Thermo Scientific). The protein lysates were analyzed for acetylated tubulin by Western blot. Selected compounds were also tested in bone marrow-derived macrophages (BMDMs) from wild-type C57BL/6 mice. Briefly, the femur and tibia bones were carefully removed from the mouse and completely separated from the muscles. Using scissors, one end of each bone was opened to expose the bone marrow. With the open ends at the bottom, the bones were placed in a 0.6 mL tube with a hole and centrifuged (at 8,000 rpm for 30 s) into a 2 mL Eppendorf tube. The bone marrow cells were cultured in complete RPMI with 20 ng/mL M-CSF for 4 days, followed by 3 days in M-CSF-free medium. Cells were pretreated overnight and again 2 h prior to polarization with either M1-inducing agents (100 ng/mL LPS + 50 ng/mL IFN-γ) or M2-inducing agents (20 ng/mL IL-4 + 20 ng/mL IL-13). Cells were collected in TRIzol for RNA or RIPA buffer for protein analysis. Western blots and qRT-PCR assays were used to quantify M1 and M2 markers. Antibodies and primers are listed in the Table S1 and Table S2.

#### Phagocytosis

BMDMs were isolated from C57BL/6-Tg (UBC-GFP) mice and cultured as described above. On day 5, macrophages were gently scraped down and transferred to a 96-well black plate (12,000 cells/well in 200 μL media). Twenty-4 h later, when the macrophages had attached to the plate, the cells were treated overnight with half the dose (2.5 μM) of the selected tetrazolone, SM-06-09, and Nexturastat A (NextA) as a control, and the other half dose was administered the next day before polarization into the M1 phenotype. After 24 h, SM1 cells (murine melanoma cell line) stained with CellTrace Violet (Thermo Fisher, C34571) and pHrodo Red succinimidyl ester (pHrodo Red SE, Thermo Fisher, P36600) dyes were cocultured with macrophages at a 2:1 ratio (25,000 cells/well). The plate was immediately transferred to the Image Express Pico Imager (Molecular Devices) to capture images every 30 min for 12 h. The results were analyzed by CellReporter Xpress.

#### Antigen Presentation

BMDMs from wild-type C57BL/6 animals were polarized to an M1-like macrophage phenotype on day 6 by treating them with LPS and IFN-γ at the above-specified concentrations. The following day, the M1 macrophages were treated with SM-06-09 or NextA 1 h before OVA peptide treatment. After 4 h of incubation with the OVA peptide, the macrophages were stained for Live/Dead exclusion using the Fixable LIVE/DEAD Aqua Dead Cell Stain Kit (Thermo Fisher Scientific) for 20 min at room temperature. The macrophages were then stained with antibodies against CD80, H2Kb, or H2Kb bound to the SIINFEKL peptide for 30 min. The treatment-associated changes in the presentation of the SIINFEKL peptide were measured using flow cytometry, and the statistical significance of the mean fluorescent intensity was analyzed using GraphPad Prism.

#### T Cell Proliferation

BMDMs were isolated from wild-type C57BL/6 mice, as described above. Cells were pretreated with NextA or SM-06-09 and polarized to M1 and M2-like macrophages. T cells were isolated from mouse spleens using the EasySep Mouse T Cell Isolation Kit (Stemcell Technologies, Cat # 19851). The T cells were stained with the CellTrace Violet Cell Proliferation Kit (Thermo Fisher Scientific, Cat # C34557) at a 1:4000 dilution and activated with Dynabeads Mouse T-cell Activator Cd3/Cd28 (Thermo Fisher, Cat#11452D) according to the manufacturer’s protocol. T cells were supplemented with 1U/L of the recombinant mouse IL-2 cytokine (Thermo Fisher Scientific, Cat # 212-12) in complete RPMI media. Macrophages and T cells were cocultured at a 1:2 ratio for 72 h in a 96-well plate. To determine the changes in T cell proliferation when cocultured with macrophages pretreated with tetrazolones, the cells were stained with propidium iodine and antibodies against CD4 (BV650) and CD8 (APC-Fire 750). The percentage of T cell proliferation was analyzed using FlowJo software.

#### Macrophage Migration

BMDMs from UBC-GFP mice were cultured (100,000 cells/well) in an 8-μm pore-size FluoroBlok 24-multiwell insert system (Corning, 351158). Macrophages were pretreated with a half-dose (2.5 μM) of SM-06-09 overnight and with another half-dose before polarization into M1 and M2 phenotypes. The SM1 melanoma cells were cultured (100,000 cells/well) in a separate 24-well plate (Corning, 353047). After 24 h of polarization, the inset system was transferred to the 24-well plate with SM1 cells. The insert was removed after 24 h, and images were acquired using a Molecular Devices ImageXpress Pico imager. The number of GFP-labeled macrophages that migrated to the bottom well was determined using CellReporter Xpress image acquisition and analysis software, and statistical significance was assessed with GraphPad.

#### In Vivo Studies

Animal studies were conducted in accordance with guidelines and the protocol approved by the Institutional Animal Care and Use Committee (IACUC) at Georgetown University (Protocol # 2022-0016). C57BL/6 mice were obtained from Charles River Laboratories (Bar Harbor, ME, USA). Briefly, SM1 murine melanoma cells (0.75 × 10∧6 cells in 100 μL of PBS) were injected into the flank region of 6–8 weeks old female C57BL/6 mice. Animals were stratified equally into each group when the tumor size was palpable (approximately 5 × 5 mm). In the pilot study, animals were administered 25 mg/kg of SM-06-09 by intraperitoneal (i.p.) injections every other day. The control animals received equivalent doses of vehicle (containing 70%PEG400, 20% ethanol, and 10% Tween 80). Tumor measurements and body weights were obtained once every 2 days and once a week, respectively. Tumor volumes were calculated using caliper measurements using the formula L x W^2^/2. Mice were treated until the tumor sizes in the vehicle group reached a maximum size of 2000 mm^3^. At the end of the study, tumors were harvested for immunophenotyping. In the dose-determining study, animals (*n* = 5–6 per group) received 25, 50, or 100 mg/kg of SM-06-09 administered by oral gavage daily for 2 weeks after the SM1 tumors were palpable. The vehicle for oral administration was prepared with 0.5% carboxymethylcellulose and 0.1% Tween 80. Tumor measurement and body weights were recorded every 2 days. At the end of the study, tumors were harvested for immunotyping. Additionally, spleen, liver, and lungs were harvested to determine potential cytotoxicity and metastasis. In the combination study, animals received oral administration of SM-06-09 (100 mg/kg) or vehicle daily in combination with i.p. injections of anti-PD-1 (10 mg/kg) or IgG antibodies (10 mg/kg) twice a week. Tumor measurements were recorded on alternate days, and body weights were recorded once a week. At the end of the study, tumors were harvested for flow cytometry analysis, CyToF, and single-cell (sc) RNA sequencing. Organs were harvested as described above.

#### Flow Cytometry

Tumors were harvested, and a single-cell suspension of harvested tumors was immediately generated using tumor digestion buffer (containing collagenase I, collagenase IV, DNase I, and hyaluronidase). Cells were stained with antibodies for cell surface markers for macrophages, T-cells, and NK-cells, with a minimum of 1–2 million cells in each panel. All panels included LIVE/DEAD Fixable Aqua Dead Cell Stain (Thermo Fisher Scientific, L34947) and followed the manufacturer’s protocol for determining viability. At least 30,000 events were recorded with BD LSR Fortessa and analyzed by FlowJo software. For the larger combination study, the cells were stained for viability using the Zombie NIR kit (BioLegend, 423105) and with a single panel of antibodies against macrophage, T cell, and NK cell markers. The data was acquired using the Cytek Aurora instrument. The complete list of antibodies is provided in Table S1, and the images of the gating strategy are described in the Supplementary sections.

#### CyTOF

For CyToF, about 3 × 10^6^ live cells were stained with Maxpar and custom metal-conjugated antibodies in Maxpar cell staining buffer (Fluidigm Corp), followed by fixation with freshly made 2% formaldehyde in 1X Maxpar PBS. Finally, the cell suspension was stained with Cell-ID Intercalator-Ir (Standard Biotools, 201192B) in Maxpar Fix and Perm Buffer (Standard Biotools, 201067) for live/dead cell discrimination. The cell suspension was analyzed on a Helios (Standard Biotools) mass cytometer, and data analysis was performed with the OMIQ web platform.

#### Bulk RNA Sequencing

RNA from BMDMs was isolated and submitted to Azenta Life Sciences for Next-Gen Sequencing. Alignment of reads was performed using the HISAT2 alignment pipeline,^88^ using the GRCm38 *Mus musculus* reference genome. The samtools sort tool was used to convert. sam files to binary. bam files. featureCounts was used for quantification of transcripts using the same reference genome.^89^ The DESeq2 pipeline was used for differential gene expression analysis following the standard protocol.^90^ Requirements for significant differential expression included an absolute log2FoldChange value of >1.0 and an adjusted *p*-value of <0.05. For the functional enrichment of significantly differentially expressed genes, the DE gene lists were separated into upregulated (log2FC > 1.0) and downregulated (log2FC ← 1.0) lists. Volcano plots were created using the EnhancedVolcano package.^91^ These genes served as the input to the EnrichR package,^92–94^ using the Gene Ontology Biological Processes (GO-BP) library.

#### Single-Cell RNA Sequencing

One representative tumor from each of the vehicle, SM-06-09, and anti-PD-1 mono- and combination-treatments was selected for sorting CD45+ cells. The single-cell suspensions from these tumors were stained with CD45.2 antibodies. Cytox Blue cell stain was used to distinguish live and dead cells. The stained cells were applied on the Cytex Spectral Flow Cytometer platform to sort CD45+ cells. The sorted cells were counted using a Countess II cell counter (Thermo Fisher Scientific) according to the manufacturer’s instructions. The cells were determined to be suitable for sequencing when the viability was over 80%. 7,000 cells were loaded onto the 10× Chromium system and encapsulated using the Single Cell 3′ Library and Gel Bead kit (10× Genomics). Single-cell gene expression libraries were generated according to the manufacturer’s instructions. The completed libraries were used for pooling and sequencing on NovaSeq X Plus platforms (service by Novogene). Raw FASTQ files from 10× Genomics were analyzed using the cellranger count function through Cellranger v.9.0.1. Normalization and downstream analysis were performed using the Seurat R package.^95^ Primary immune cell types were annotated using SingleR,^96^ using the mouse Immunological Genome Project library (ImmGenData) as the reference.^97^ To label macrophage and T cell subtypes, we investigated differentially expressed genes for each cluster using the “FindAllMarkers” function within Seurat (log2FC > 1.0 and adj *p* < 0.05). Combining known markers of immune cell subtypes and results from EnrichR functional enrichment, we were able to distinguish six T cell subpopulations and eight TAM subpopulations. Trajectory analysis was performed using Monocle 3.^98^

#### Statistical Analysis

All statistical analyses were performed using GraphPad Prism Software (version 10.3.1). The *P* values were determined by unpaired *t* test, one-way or two-way ANOVA, depending on the data set. The error bars are represented as ± standard deviation in each figure. The *P* values <0.05 are represented with an asterisk (*), and the specific statistical test applied is described in the legends of each figure.

## Supplementary Material





## Notes

^1^H and ^13^C­{^1^H} NMR spectra for compounds **5a**–**o**, **6a**–**o**, **7**, **9**-**11** and **3a**–**q;** HPLC trace of compounds **3a**-**q**; Maestro 2D ligand interaction diagrams for compounds **3a**–**3d** (**Figure S1**) and **3m** (**Figure S2**) docked to drHDAC6-CD2 (PDB: 6R0K); screening of nonlead tetrazolones for HDAC6 inhibition (**Figure S3**); HDAC6 inhibition improved the pro-inflammatory M1 phenotype in bone marrow-derived macrophages at nanomolar concentrations (**Figure S4**); selected HDAC6 inhibitors with modest effects on the macrophage phenotype (**Figure S5**); selected tetrazolones show sustained HDAC6 inhibition after a single treatment dose in BMDMs (**Figure S6**); functional enrichment analysis of differentially expressed genes between SM-06-09-treated and untreated BMDMs reveals enhanced inflammatory and tumor immune responses in boh M1- and M2-polarized macrophages (**Figure S7**); time course of Antigen-presentation in M1 macrophages treated with SM0609 (**Figure S8**); SM-06-09 reduces tumor growth and increases M1/M2 macrophage ratio in the TME in the SM1 murine melanoma model (**Figure S9**); dose determination for oral administration of SM-06-09 and the potential side effects in the SM1 melanoma model (**Figure S10**); feature plots representing the distribution of immune cell populations in opt-SNE (**Figure S11**); distinction of monocyte and macrophage nodes for trajectory analysis (**Figure S12**); list of antibodies used in flow cytometry and Western blot assays (**Table S1**);list of primers used for qPCR assay (**Table S2**); list of reagents (**Table S3**) (PDF)
Molecular formula strings including screening data (CSV)

## ∇

AbbVie Inc., 1 N. Waukegan Rd, North Chicago, IL 60064, United States

## ○

Argonne National Laboratory, 9700 Cass Ave, Lemont, IL 60439, United States.

## ◆

Lankenau Institute for Medical Research, Main Line Health, Wynnewood, PA 19096, United States.

## Abbreviations Used

ANOVA: analysis of variance
BCA: bicinchoninic acid (protein assay)
BMDM: bone marrow-derived macrophage
CM: central memory
CyToF: cytometry by time-of-flight (mass cytometry)
DE: differentially expressed
ECM: extracellular matrix
EM: effector memory
EMT: epithelial–mesenchymal transition
GO-BP: Gene Ontology Biological Processes
HDAC: histone deacetylase
HDACi: histone deacetylase inhibitor
HDAC6i: histone deacetylase 6 inhibitor
ICB: immune checkpoint blockade
IFN-γ: interferon γ
iNOS: inducible nitric oxide synthase
LE: ligand efficiency
LipE: lipophilic efficiency
LPS: lipopolysaccharide
M-CSF: macrophage colony-stimulating factor
MDSC: myeloid-derived suppressor cell
MHC: major histocompatibility complex
NK: natural killer
OVA: ovalbumin
RAW264.7: murine macrophage cell line
RMSD: root-mean-square deviation
scRNA-seq: single-cell RNA sequencing
SI: selectivity index
TAM: tumor-associated macrophage
TME: tumor microenvironment
TRIzol: phenol/guanidinium thiocyanate RNA isolation reagent
ZBG: zinc-binding group
