# Supplementary material for: Macrophage-Centric Phenotypic Screening Identifies Tetrazolone-Based HDAC6 Inhibitors That Reprogram the Tumor Immune Microenvironment and Improve Immune Checkpoint Blockade
Source: J Med Chem. 2026 May 29;69(11):12870–97. doi: 10.1021/acs.jmedchem.5c02453 (PMC13266995; doi:10.1021/acs.jmedchem.5c02453)
Supplement: Supplementary file 1 [file jm5c02453_si_001.pdf]

Supporting Information

Macrophage-centric phenotypic screening identifies HDAC6 tetrazolone-based inhibitors that reprogram the tumor immune microenvironment and improve immune checkpoint blockade.

Nithya Gajendran[a,#,] Manasa Suresh[a,#], Sebastian J. Marquez Rodriguez[b,#], Sruthi Mohan[b,#], Tim Ponsot[b], David Quiceno-Torres[a], Mario A. Noboa[b], Bryan Weselman[a], Xintang Li[a], Marie Durr[a], Zora Novakova[c], Mike Schutkowski[d], Matias I. Hepp[e], Satish Noonepalle[a], Cyril Bařinka[c], Duncan J. Wardrop[b,*], and Alejandro Villagra[a,e,*].

Affiliations:

[a] Georgetown University, Washington, DC 20057, USA

[b] University of Illinois Chicago, Chicago, IL 60607, USA

[c] Laboratory of Structural Biology, Institute of Biotechnology of the Czech Academy of Sciences, BIOCEV, Prumyslova 595, 252 50 Vestec, Czech Republic

[d] Charles Tanford Protein Center, Department of Enzymology, Institute of Biochemistry and Biotechnology, Martin-Luther-University of Halle-Wittenberg, 06120 Halle (Saale), Germany

[e] Laboratorio de Investigación en Ciencias Biomédicas, Departamento de Ciencias Básicas y Morfología, Facultad de Medicina, Universidad Católica de la Santísima Concepción, Concepción 2850, Chile

# These authors contributed equally.

* Corresponding Authors

Email: Alejandro.villagra@georgetown.edu. Phone: +1-202-687-4840 (A.V.)

Email: wardropd@uic.edu. Phone: +1-312-355-1035 (D.J.W.)

TABLE OF CONTENTS

1. **Materials and Methods** ………………………………………. .... ………… S4-S5
   1.1 Safety Considerations …………... ………………… S4
   1.2 Preparation of Known Compounds …………...................... ………………… S4
   1.3 **Table S1**. List of antibodies …………..........................………………… S4-S5
   1.4 **Table S2**. List of primers ………… .... ………………… S5

2. **Computational Methods** .... …………S6-S10
   2.1 Docking Studies .... …………………………..S6–S7
   2.2 **Figure S1**. Maestro 2D ligand interaction diagrams for compounds **3a**–**3d** docked to drHDAC6-CD2 (PDB: 6R0K)……………………………………...S8-S9
   2.3 **Figure S2**. Maestro 2D ligand interaction diagrams for compound **3m** docked to drHDAC6-CD2 (PDB: 6R0K). .... …………………………………………….. S10

3. **Biological and Computational Data** .... ………… S11
   3.1 **Table S3**. HDAC6 Inhibitory Activity and Computational Binding Scores for Compounds **3a**–**3q** ……… ..... ……………………………………….. S11

4. **Spectroscopic Characterization**...…S12-S69
   4.1 NMR Spectra (^{1}H and ^{13}C{^{1}H}) for All Compounds .....………………..S12–S60
   4.2 HPLC Chromatograms … ....…………………………………………… S61–S69

5. **HDAC6 screening and functional assays supporting data**................…S70-S77
   5.1 **Figure S3**. Screening of non-lead tetrazolones for HDAC6 inhibiton.S70–S71
   5.2 **Figure S4**. HDAC6 inhibition improved the pro-inflammatory M1 phenotype in bone marrow-derived macrophages at nanomolar concentration.……….........S72
   5.3 **Figure S5**. Selected HDAC6 inhibitors with modest effects on the macrophage phenotype…...…S73-S74
   5.4 **Figure S6**. Selected tetrazolones show sustained HDAC6 inhibiton after a single treatment dose in BMDMs… .... …S75

5.5 **Figure S7**. Functional enrichment analysis of differentially expressed genes between SM-06-09-treated and untreated BMDMs reveal enhanced inflammatory and tumor immune responses in both M1- and M2-polarized macrophages……… …..…………………………………………………………………………… S76–S77
5.6 **Figure S8**. Time course of antigen presentation in M1 macrophages treated with SM-06-09…… .... …S78

6. **In vivo studies supporting data……………….**……………………………S79-S82
6.1 **Figure S9**. SM-06-09 reduces tumor growth and increases M1/M2 macrophage ratio in the SM1 melanoma model……….……………..……….. S79
6.2 **Figure S10**. Dose determination for oral administration of SM-06-09 and the potential side effects in the SM1 melanoma model………………………………S80
6.3 **Figure S11**. Feature plots representing the distribution of immune cell populations in opt-SNE…………………………….……………….............. . .S81
6.4 **Figure S12**. Distinction of monocyte and macrophage nodes for trajectory analysis………………………………………………………..………………S82

7. **References** ……………………………………………………………………….S83

1. Materials & Methods

1.1 Safety Considerations

Trimethylsilyl azide is highly toxic and potentially explosive.[4] The reaction of trimethylsilylazide with Brønsted acids leads to the generation of hydrazoic acid (HN_3), a volatile, highly toxic, and highly explosive substance. While we have not encountered any safety issues using tetrazolones, the products described are potentially explosive. Accordingly, appropriate training and safety precautions must be taken before repeating the experiments described herein. All experiments using trimethylsilylazide were conducted behind a blast shield, with personnel wearing appropriate protective clothing for working with trimethylsilylazide. A blast shield was also employed when concentrating tetrazolone solutions *in vacuo*.

1.2 Preparation of Known Compounds

Prop-2-yn-1-yl 4-methylbenzenesulfonate, but-3-yn-1-yl 4-methylbenzenesulfonate, pent-4-yn-1-yl 4-methylbenzenesulfonate, hex-5-yn-1-yl 4-methylbenzenesulfonate, and 2-azidoethyl 4-methylbenzenesulfonate were prepared according to the method reported by Williams.[5]

1.3 Table S1: List of antibodies.

Antibody	Source	Catalog no
Brilliant Violet 421™ anti-mouse/human CD11b	Biolegend	101251
Brilliant Violet 785™ anti-mouse F4/80	Biolegend	123141
Alexa Fluor® 700 anti-mouse CD3	Biolegend	100216
APC anti-mouse CD80	Biolegend	104714
PE/Cyanine7 anti-mouse CD206 (MMR)	Biolegend	141720
Brilliant Violet 570™ anti-mouse Ly-6C	Biolegend	128030
Brilliant Violet 650™ anti-mouse CD4	Biolegend	100469
PerCP/Cyanine5.5 anti-mouse CD8a	Biolegend	100734
Pacific Blue™ anti-mouse CD25	Biolegend	102022
Brilliant Violet 605™ anti-mouse/human CD44	Biolegend	103047
PE anti-mouse CD62L	Biolegend	104408
Alexa Fluor® 647 anti-mouse CD49b (pan-NK cells)	Biolegend	108912
FITC anti-mouse NK-1.1	Biolegend	108706

APC/Fire™ 750 anti-mouse CD45.2	Biolegend	109852
Brilliant Violet 510™ anti-mouse CD335 (NKp46)	Biolegend	137623
Purified anti-mouse CD16/32	Biolegend	101302
PE anti-mouse H-2Kb	Biolegend	116508
APC anti-mouse H-2Kb bound to SIINFEKL	Biolegend	141606
PE anti-mouse CD80 Antibody	Biolegend	104708
α-Tubulin (DM1A) Mouse mAb	Cell Signaling Technology	3873S
Acetyl-α-Tubulin (Lys40) Antibody	Cell Signaling Technology	3971S
Arginase-1 (E4U1I) Mouse mAb	Cell Signaling Technology	43933S
iNOS rabbit polyclonal antibody	Thermo Fisher	PA3-030A

1.4 Table S2. List of primers.

Gene	Primer	Primer sequence
Nos2	F	GAGACAGGGAAGTCTGAAGCAC
	R	CAGCAGTAGTTGCTCCTCTTC
Tnf	Primer mix	Primer mix was obtained from Qiagen – QT001040006 – Mm_Tnf_1_SG QuantiTect Primer Assay (200) Catalog # 249900
Arg1	Primer mix	Primer mix was obtained from Qiagen – PPM31770C-200 – RT2 qPCR Primer Assay for Mouse Arg1 Catalog # 330001
Fizz1	F	TCCAGCTAACTATCCCTCCACTGT
	R	GGCCCATCTGTTCATAGTCTTGA
β-actin	F	CATTGCTGACAGGATGCAGAAGG
	R	TGCTGGAAGGTGGACAGTGAGG

2. Computational Methods

2.1 Docking Studies

Molecular docking was performed using the Schrödinger Suite (Maestro v14.2.118; MMShare 6.8.118; Schrödinger, LLC, New York, NY, USA; Release 2024-4), employing the Glide docking algorithm (v2024-4.5).[6] Computations were executed on a high-performance Linux workstation (Exxact Tensor TWS-1686525-SMD) running Ubuntu 24.04.3 LTS, equipped with dual Intel® Xeon® Silver 4210 CPUs (40 cores), 192 GB DDR4 RAM, and a multi-GPU configuration (2× NVIDIA Quadro RTX 5000, 1× Quadro P620). Visualization and post-docking analyses were conducted using PyMOL (v2.5) and LigPlot$^+$.

Protein Preparation

The X-ray crystal structure of *Danio rerio* histone deacetylase 6 (HDAC6) catalytic domain 2 (CD2) in complex with the hydroxamate inhibitor SS-208 (PDB ID: 6R0K)[7] was retrieved from the RCSB Protein Data Bank. Protein preparation was carried out using the Protein Preparation Wizard in Maestro.[8] Bond orders were assigned, hydrogens were added, and zero-order bonds were defined between the catalytic Zn^{2+} ion and its coordinating residues to preserve metal coordination geometry during minimization. Protonation states of ionizable residues were predicted using the Epik module at pH 7.0 ± 2.0, and the hydrogen-bonding network was optimized. All crystallographic water molecules were removed. Restrained energy minimization was performed using the OPLS4 force field,[8,9] with heavy atoms restrained to within 0.30 Å RMSD of the crystal structure while allowing hydrogen atoms and nearby side chains to relax.

Ligand Preparation

Ligands were prepared using LigPrep. Salts were removed, and 3D structures were generated and energy minimized using the OPLS4 force field. Protonation and tautomeric states were enumerated with Epik at pH 7.0±2.0. For compounds with undefined stereochemistry, all possible stereoisomers were retained. Ligands were prepared in multiple states for subsequent docking.

Docking Protocol

The co-crystallized ligand SS-208 was extracted and used to define the binding site for docking. A 10×10×10 $Å^3$ receptor grid was generated, centered on the centroid of SS-208. A centroid positional constraint was applied to the hydroxamic acid moiety of SS-208 to guide proper orientation of Zn^{2+}-binding ligands. In addition, a metal-coordination constraint (Zn–O/N) was applied (rewarding ligands that coordinate the catalytic Zn^{2+} ion). Default settings were used for remaining parameters, including van der Waals scaling (0.80) and partial charge cutoff (0.15). Docking was performed using Glide in standard precision (SP) mode with flexible ligand sampling. Up to 10 poses were generated and scored per ligand.

Validation of Docking Protocol

To validate the docking procedure, the co-crystallized ligand SS-208 was re-docked into the HDAC6 active site. The top-scoring pose reproduced the crystallographic binding mode with an RMSD of 0.43 Å (heavy atoms), supporting the reliability of the docking setup and applied constraints.

Scoring and Ranking of Docked Poses

Docked poses were scored using GlideScore and ranked accordingly. The top-ranked pose for each ligand was visually inspected and further evaluated based on coordination with the catalytic Zn^{2+} ion and interactions with conserved active site residues observed in the reference structure, including His573, Asp612, and Tyr745.

Rescoring

Post-docking refinement and rescoring were conducted using Prime MM-GBSA with the OPLS4 force field and the VSGB 2.1 implicit solvent model.[10] For each ligand–protein complex, the ligand and protein residues within 5.0 Å of the ligand were allowed to relax using the local minimization protocol in Prime. This refinement provided estimates of ΔG_{bind} to support structure–activity relationship (SAR) interpretations.

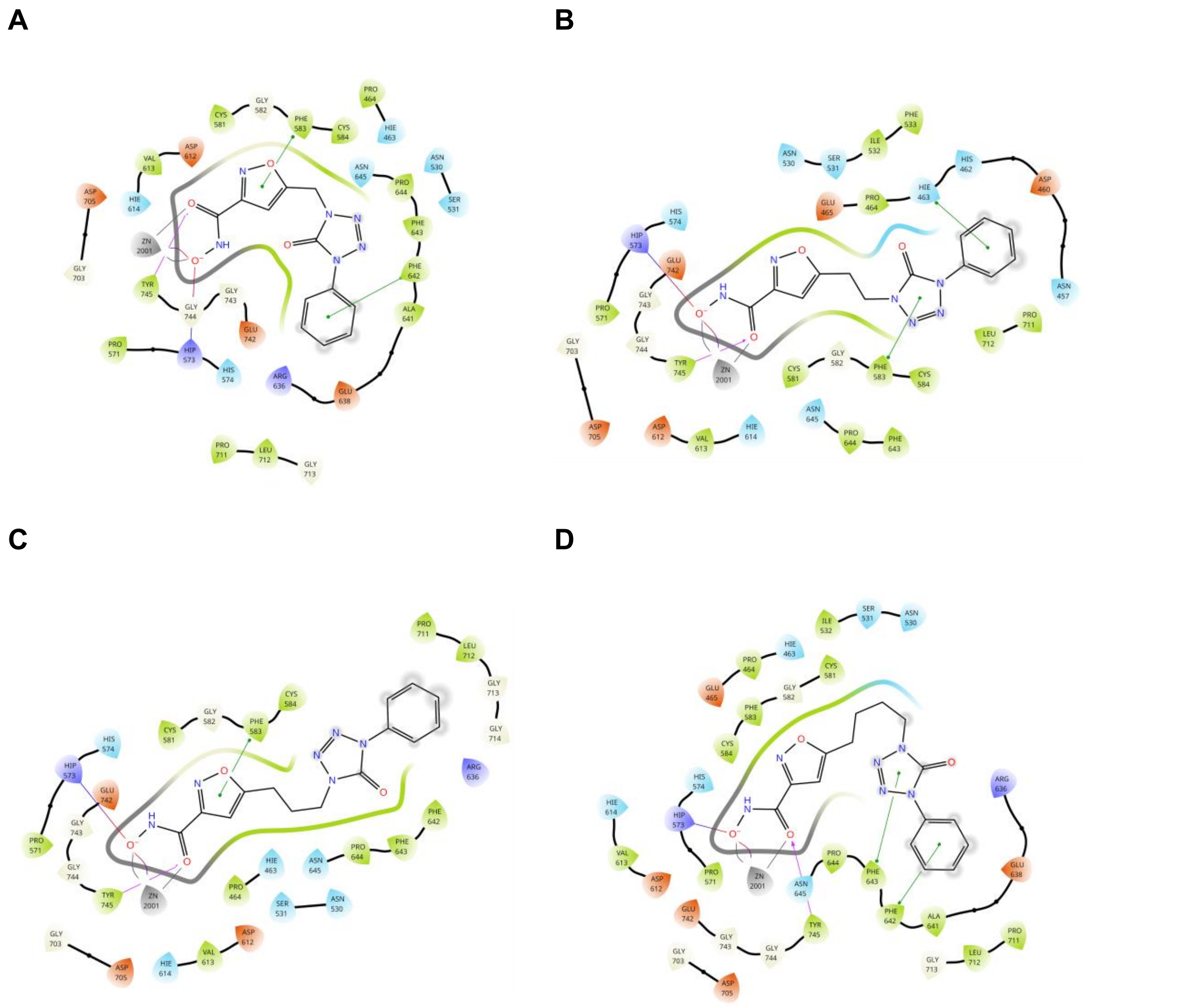

Figure S1. Maestro 2D ligand interaction diagrams for compounds 3a–3d docked to drHDAC6-CD2 (PDB: 6R0K). Two-dimensional ligand interaction diagrams (LIDs) generated by the Maestro Ligand Interaction Diagram module (Schrödinger 2024-4) for the Glide SP docked poses of the phenyl-capped linker homologs **3a**–**3d** in the catalytic domain (CD2) of *Danio rerio* HDAC6. In each panel, the ligand is shown in the center with protein residues arranged by their spatial proximity in the binding site. Color coding: green lines and filled circles denote π–π stacking contacts; purple dashed arrows indicate metal coordination to the catalytic Zn^{2+} ion (ZN 2001); red and blue lines represent hydrogen bonds to charged and neutral residues, respectively. The broad shaded ribbons depict regions of hydrophobic (olive/green) and polar (gray) contact along the protein surface. Residue labels are colored by character: hydrophobic (green), polar (cyan), acidic (orange), basic (violet). **(A)** Compound **3a** (*n* = 1, methylene linker; IC_{50} = 29.1 nM). The hydroxamic acid chelates Zn^{2+} in bidentate fashion, with hydrogen bonds to Y745 and H573. The shorter methylene tether directs the phenyl cap toward F583 and F642, positioning it deeper into the aromatic crevice rather than toward the L1-loop surface rim. A π-contact with F583 (centroid distance ~3.9 Å) and a van der Waals contact with F643 (~4.8 Å) are indicated by green lines. S531 is proximal but not engaged in a direct hydrogen bond to the tetrazolone carbonyl in this pose. **(B)** Compound **3b** (*n* = 2,

ethylene linker; IC_{50} = 4.2 nM). The two-carbon spacer projects the phenyl cap toward the L1-loop surface, generating π-contacts with H463 (~5.3 Å) and F583 (~5.3 Å), indicated by green lines. This dual engagement of the L1-loop pocket, absent in the other homologs, is consistent with the superior potency of **3b** within the sub-series. The tetrazolone carbonyl is positioned within hydrogen-bonding distance of S531 at the L1/L2 loop interface. **(C)** Compound **3c** (*n* = 3, propylene linker; IC_{50} = 26.6 nM). The longer three-carbon linker repositions the phenyl cap away from H463 toward the F583 region, where a single π-contact (~3.9 Å) is maintained (green line). No second aromatic cap contact is recorded. The cap appears to extend toward the L712/P711 region at the periphery of the L1-loop pocket. The loss of the H463 contact relative to **3b** is consistent with the approximately five-fold reduction in potency. **(D)** Compound **3d** (*n* = 4, butylene linker; IC_{50} = 36.3 nM). The four-carbon linker directs the phenyl cap past the F583 contact observed for shorter homologs and toward the F643/F642 aromatic pair at the tunnel rim (green lines). A single cap–residue π-contact with F642 (~4.8 Å) is indicated. The displacement of the cap from the H463/F583 pocket, the region engaged by **3b**, is consistent with the reduced potency of this homolog and with the increasing rotatable-bond penalty (e_{rotb} = 1.13) predicted by the scoring function.

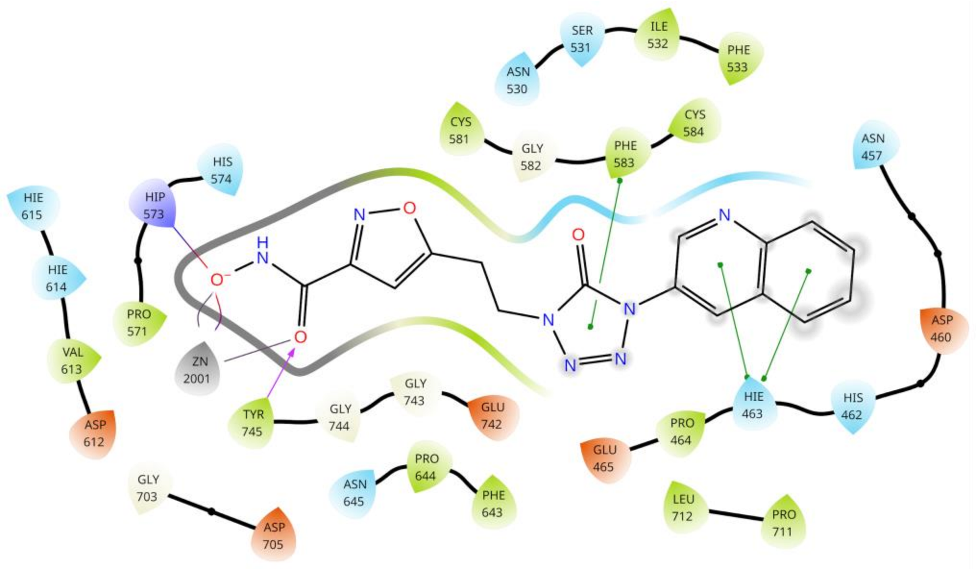

Figure S2. Maestro 2D ligand interaction diagrams for compound 3m docked to drHDAC6-CD2 (PDB: 6R0K). Compound **3m** (*n* = 2, 3-quinolinyl cap; IC_{50} = 0.49 nM). The two-carbon ethylene linker, identical to that of **3b**, projects the expanded quinolinyl cap toward the L1-loop surface. The fused bicyclic ring system engages H463 through two π-contacts (~4.9 and ~5.5 Å; green lines), compared with the single H463 contact of **3b**. A π-contact with F583 (~5.3 Å) is also maintained. The tetrazolone carbonyl retains its hydrogen bond to S531. These additional cap–residue interactions are consistent with the >10-fold potency enhancement of **3m** over **3b** and highlight the benefit of expanded aromatic surface area for L1-loop engagement. All docking was performed using Glide SP (Schrödinger 2024-4) against the drHDAC6-CD2 structure (PDB: 6R0K) with crystallographic waters removed and a Zn–O/N metal-coordination constraint. Residue numbering refers to the drHDAC6-CD2 sequence. Distances are approximate centroid–centroid values derived from the SP docked poses. These interaction diagrams represent computational models; confirmation of the depicted contacts requires co-crystallographic analysis.

3. Biological and Computational Data

Compound	SMILE	HDAC6 IC_{50} (nM)	Docking Score (kcal mol^{-1})	MM-GBSA (kcal mol^{-1})
**3a**	O=C(N(C1=CC=CC=C1)N=N2)N2CC3=CC(C(NO)=O)=NO3	29.1±1.1	-7.864	-33.83
**3b**	O=C(NO)C1=NOC(CCN2C(N(C3=CC=CC=C3)N=N2)=O)=C1	4.2±1.6	-8.035	-33.62
**3c**	O=C(C1=NOC(CCCN2N=NN(C2=O)C3=CC=CC=C3)=C1)NO	26.6±1.8	-7.274	-36.53
**3d**	O=C(C1=NOC(CCCCN2C(N(N=N2)C3=CC=CC=C3)=O)=C1)NO	36.3±3.4	-7.795	-30.63
**3e**	O=C1N(N=NN1CCC2=CC(C(NO)=O)=NO2)C3=CC=C(C=C3)Br	2.2±1.5	-8.388	-31.81
**3f**	O=C1N(N=NN1C2=CC(C)=CC=C2)CCC3=CC(C(NO)=O)=NO3	2.8±2.1	-8.098	-35.54
**3g**	O=C1N(N=NN1C2=CC(F)=CC=C2)CCC3=CC(C(NO)=O)=NO3	3.5±2.1	-8.009	-34.04
**3h**	O=C1N(N=NN1C2=C(C=CC=C2)I)CCC3=CC(C(NO)=O)=NO3	194.7±68	-8.62	-30.94
**3i**	O=C1N(N=NN1C2=C(C=CC=C2)F)CCC3=CC(C(NO)=O)=NO3	7.5±3.1	-7.389	-29.6
**3j**	O=C1N(N=NN1C2=CC(I)=C(C=C2)F)CCC3=CC(C(NO)=O)=NO3	1.8±0.5	-8.2	-32.22
**3k**	O=C1N(N=NN1CCC2=CC(C(NO)=O)=NO2)C3=CC(I)=C(C=C3)Cl	1.3±0.3	-8.589	-37.8
**3l**	O=C1N(N=NN1C2=CC(I)=C(C=C2)C(F)(F)F)CCC3=CC(C(NO)=O)=NO3	3.1±0.7	-8.055	-37.59
**3m**	O=C1N(N=NN1CCC2=CC(C(NO)=O)=NO2)C3=CC4=C(N=C3)C=CC=C4	0.49±0.01	-8.897	-34.14
**3n**	O=C1N(N=NN1CCCCCCCCC)CCC2=CC(C(NO)=O)=NO2	27±16.3	-6.535	-32.36
**3o**	O=C1N(N=NN1/C=C/C2=C(C(F)=C(C(F)=C2F)F)F)CCC3=CC(C(NO)=O)=NO3	4.7±2.6	-7.034	-34.66
**3p**	O=C1N(N=NN1CCC2=NN=C(S2)C(NO)=O)C3=CC=CC=C3	37±3.4	-8.135	-34.27
**3q**	O=C1N(N=NN1C2=CC=CC=C2)CCN3C=C(N=N3)C(NO)=O	198.9±139.9	-8.083	-31.82

Table S3. HDAC6 Inhibitory Activity and Computational Binding Scores for Compounds 3a–3q. Experimental HDAC6 inhibitory potency (IC_{50}, nM) and computed binding scores for compounds **3a**–**3q** targeting the catalytic domain 2 (CD2) of drHDAC6 (PDB ID: 6R0K). Ligands are represented by their SMILES. Docking was performed in Glide (XP mode), and docking scores are reported as GlideScore values (kcal/mol). Post-docking binding free energies (ΔG_{bind}, kcal/mol) were obtained using Prime MM-GBSA with the OPLS4 force field and the VSGB 2.1 solvation model. Docking and MM-GBSA results reflect predicted binding affinities within the HDAC6 active site and are compared with experimental IC_{50} values to assess structure–activity relationships.

4. Spectroscopic Characterization

4.1 NMR Spectra

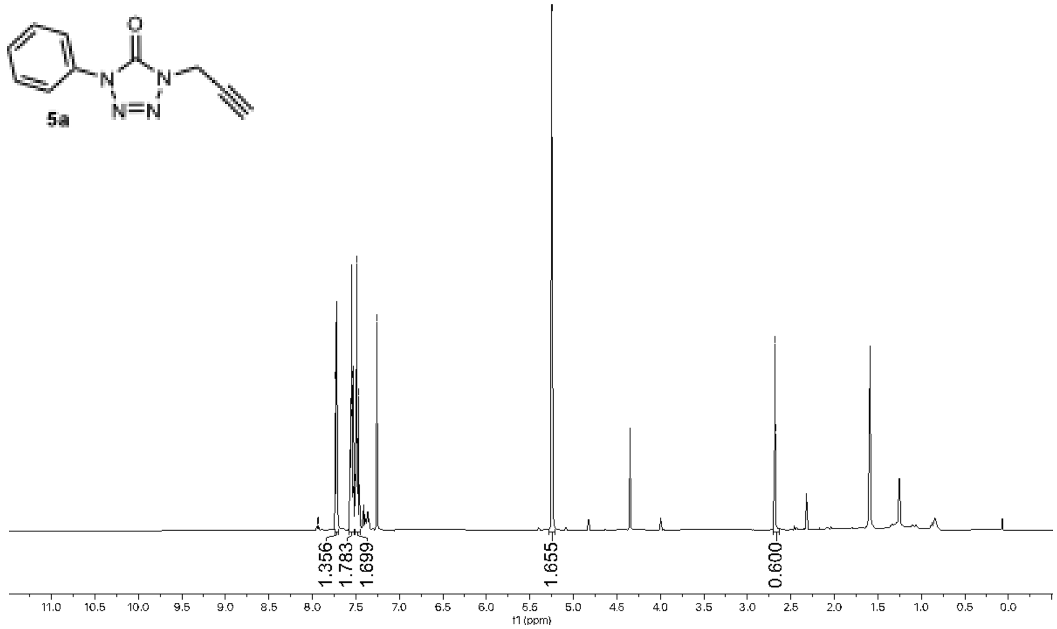

^{1}**H NMR** (500 MHz, $CDCl_3$)

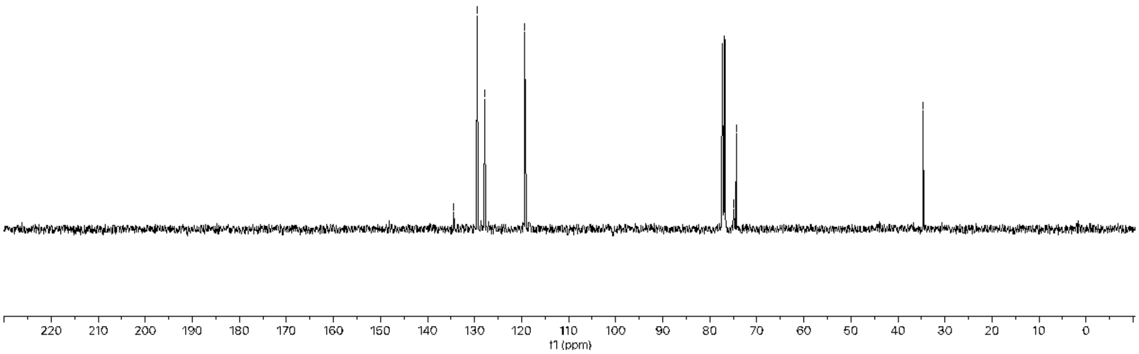

^{13}**C{^{1}H} NMR** (126 MHz, $CDCl_3$)

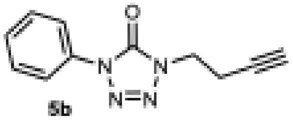

5b

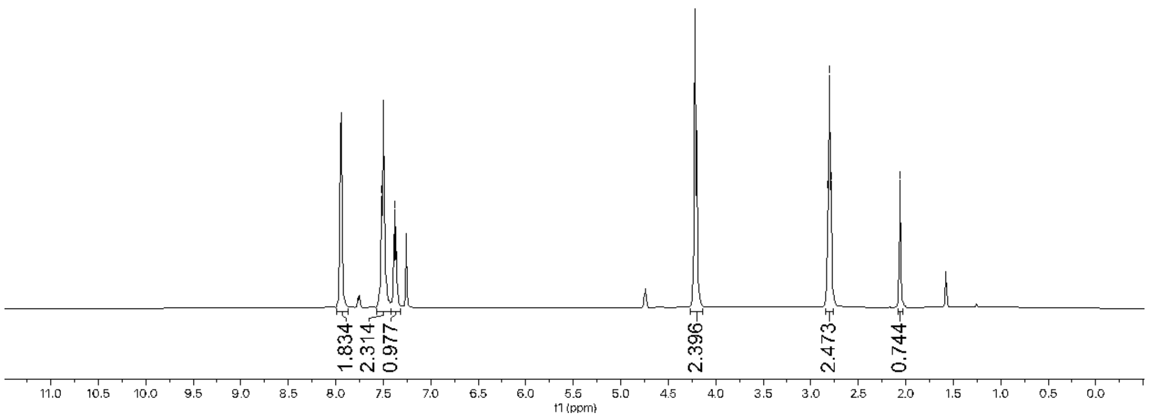

^{1}H NMR (500 MHz, $CDCl_3$)

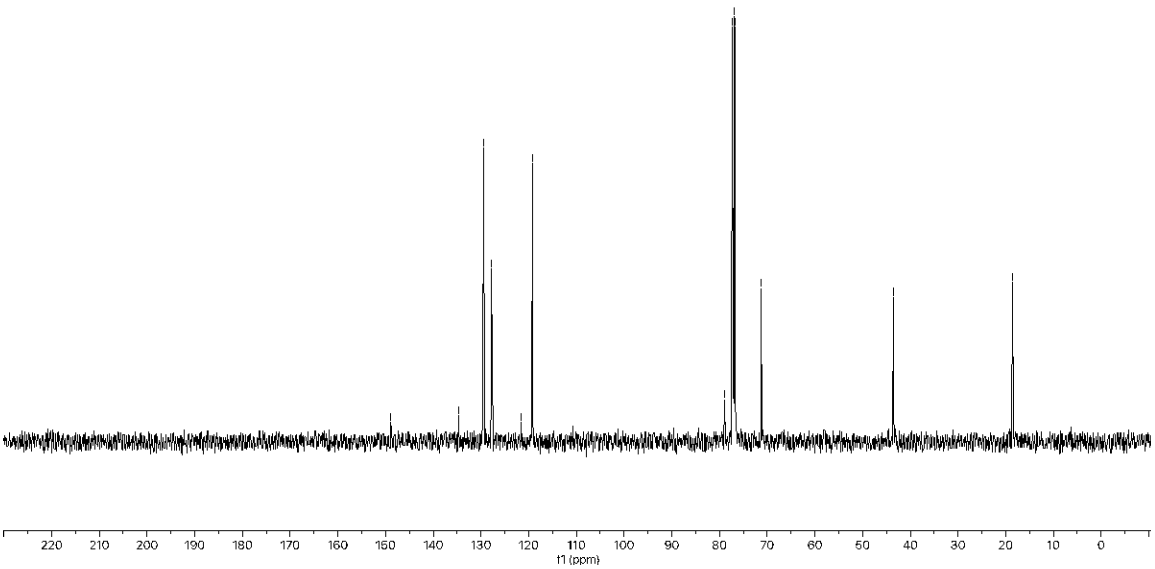

^{13}C{^{1}H} NMR (126 MHz, $CDCl_3$)

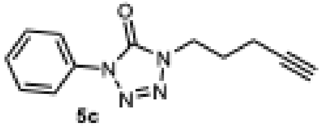

5c

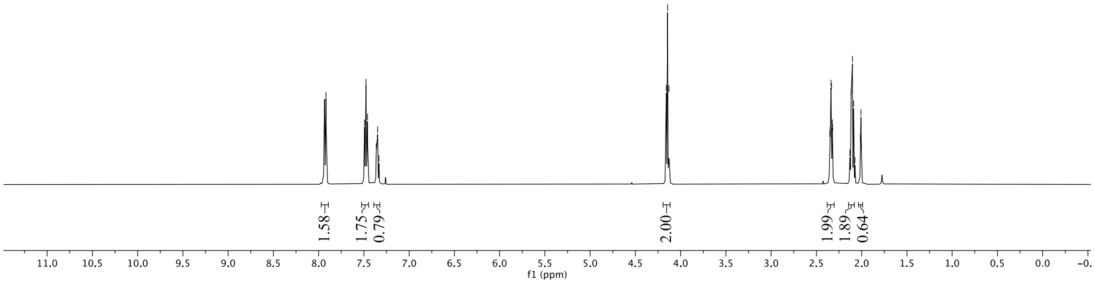

^{1}H NMR (500 MHz, $CDCl_3$)

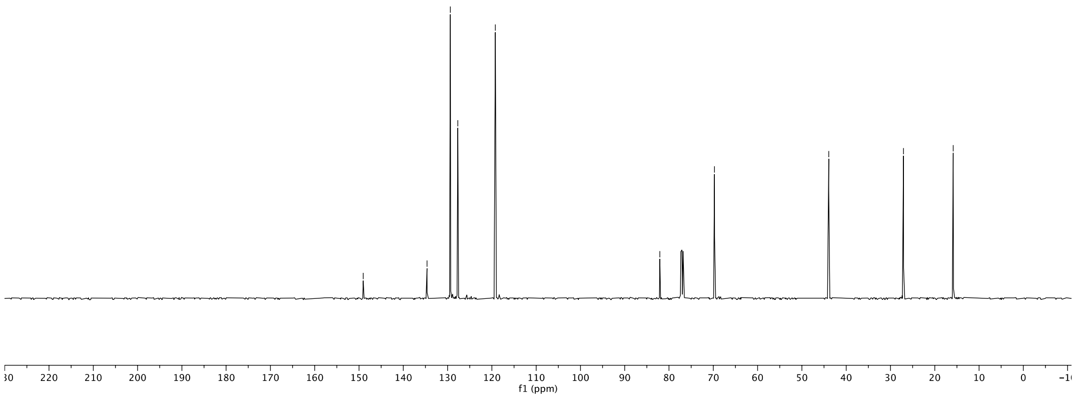

$^{13}C\{^{1}H\}$ NMR (126 MHz, $CDCl_3$)

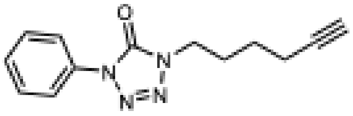

5d

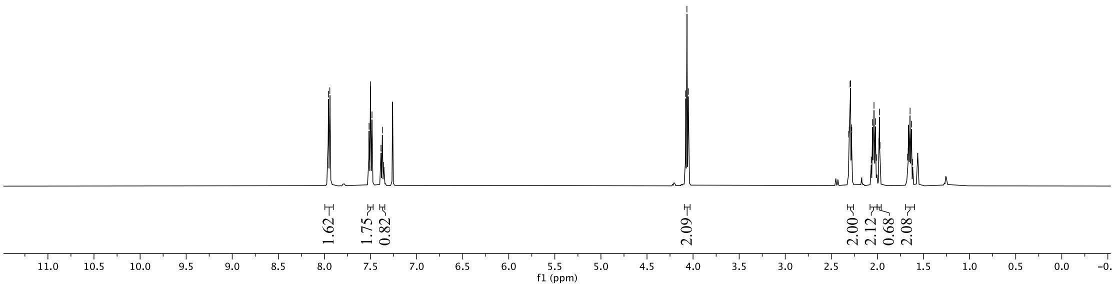

^{1}H NMR (500 MHz, $CDCl_3$)

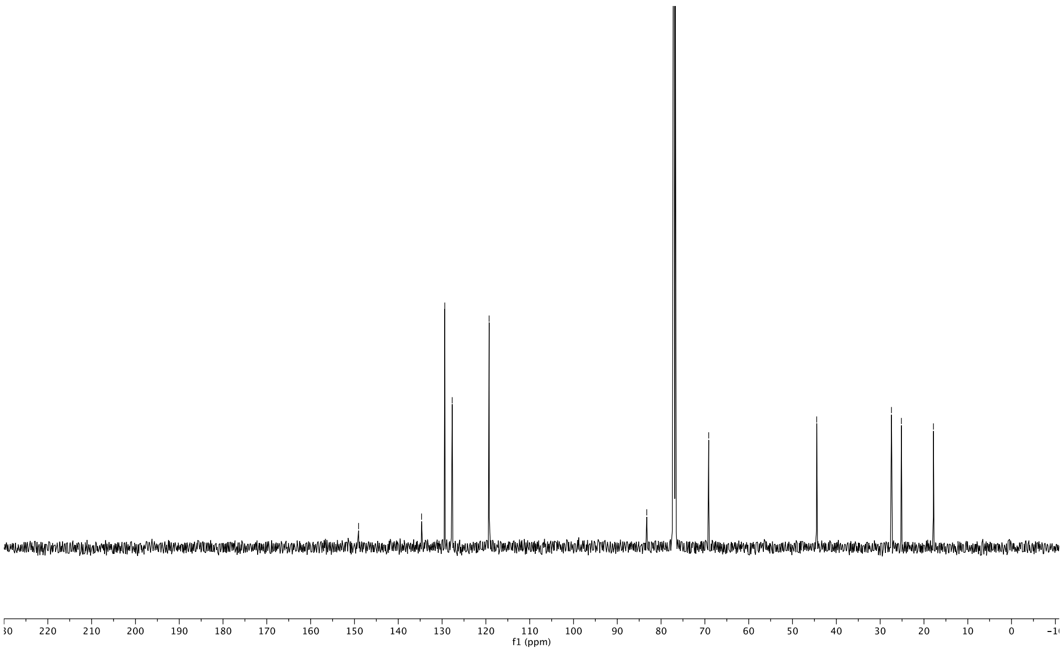

^{13}C{^{1}H} NMR (126 MHz, $CDCl_3$)

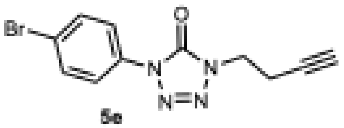

5e

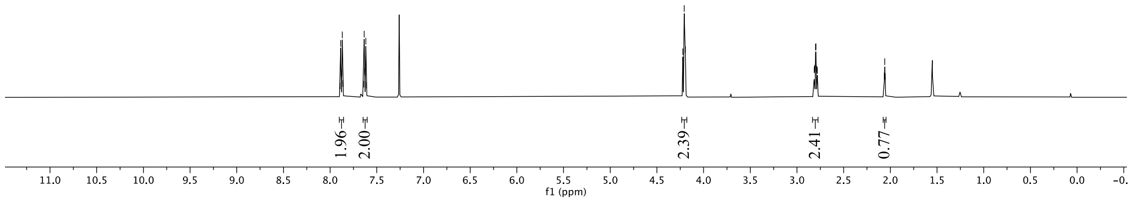

^{1}H NMR (500 MHz, $CDCl_3$)

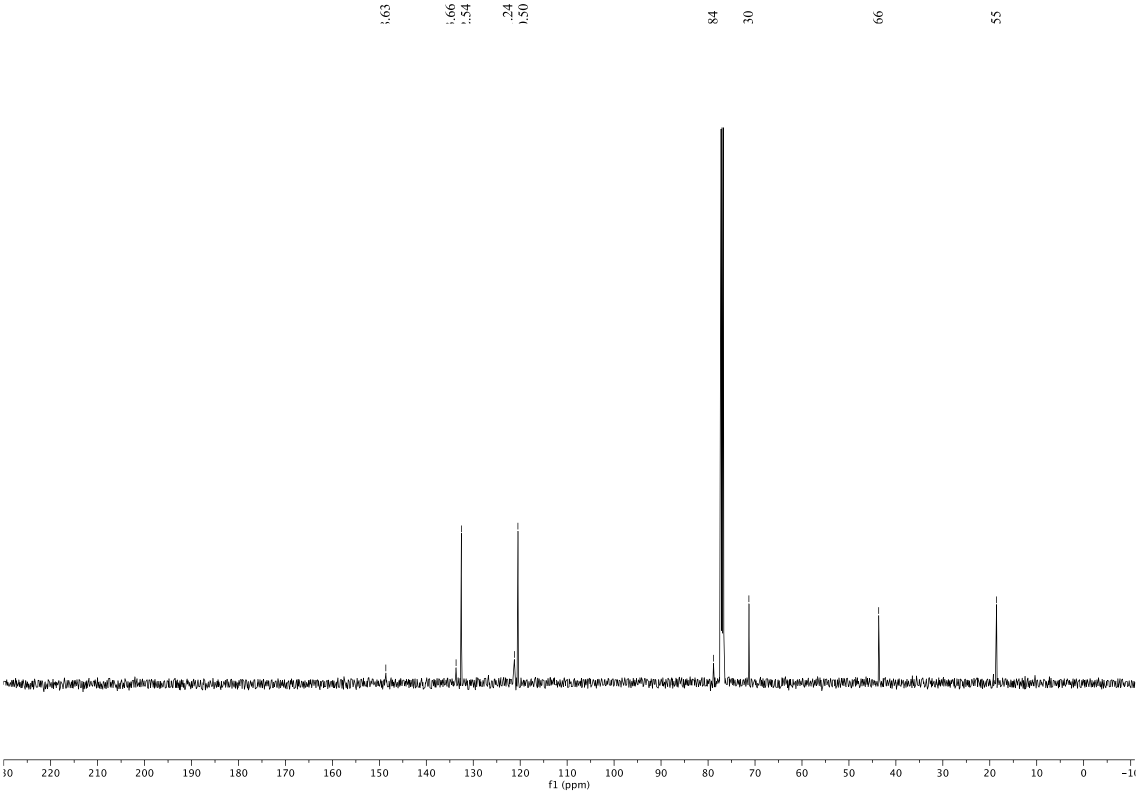

$^{13}C\{^{1}H\}$ NMR (126 MHz, $CDCl_3$)

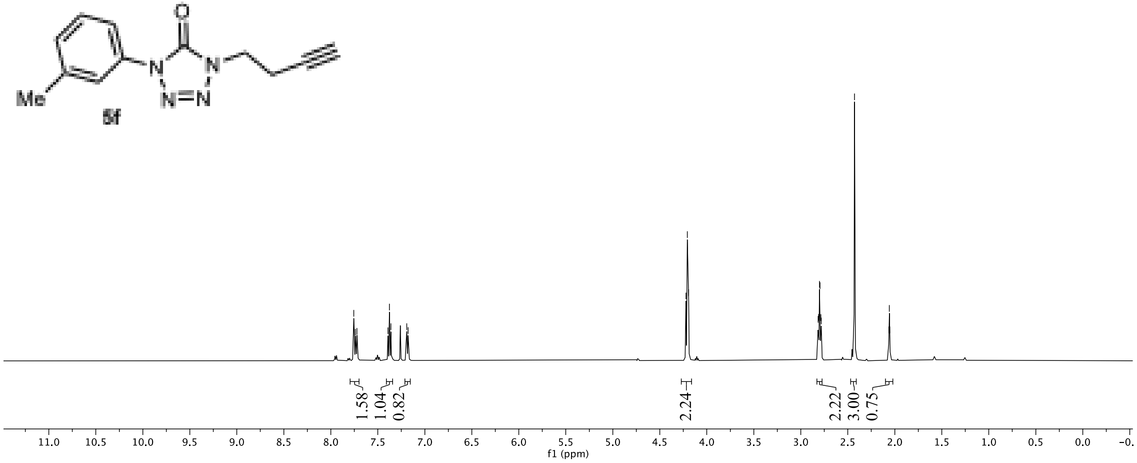

^{1}H NMR (500 MHz, $CDCl_3$)

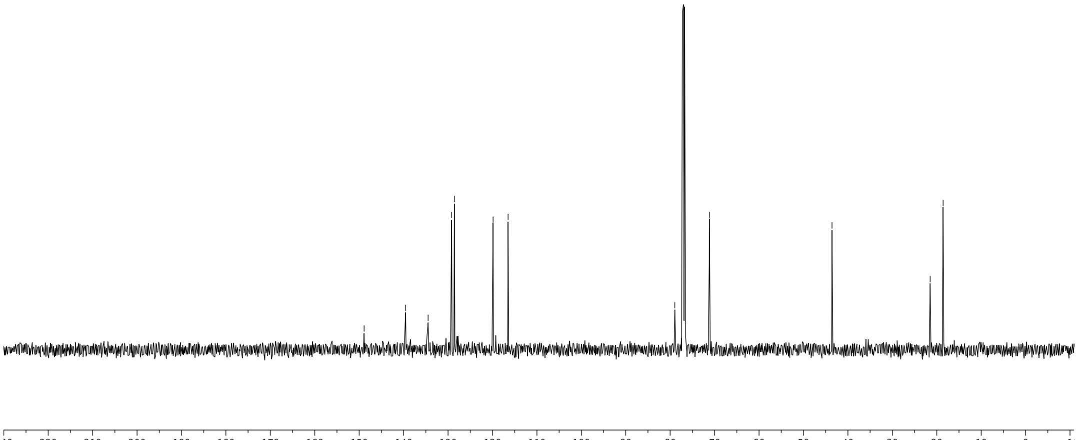

^{13}C{^{1}H} NMR (126 MHz, $CDCl_3$)

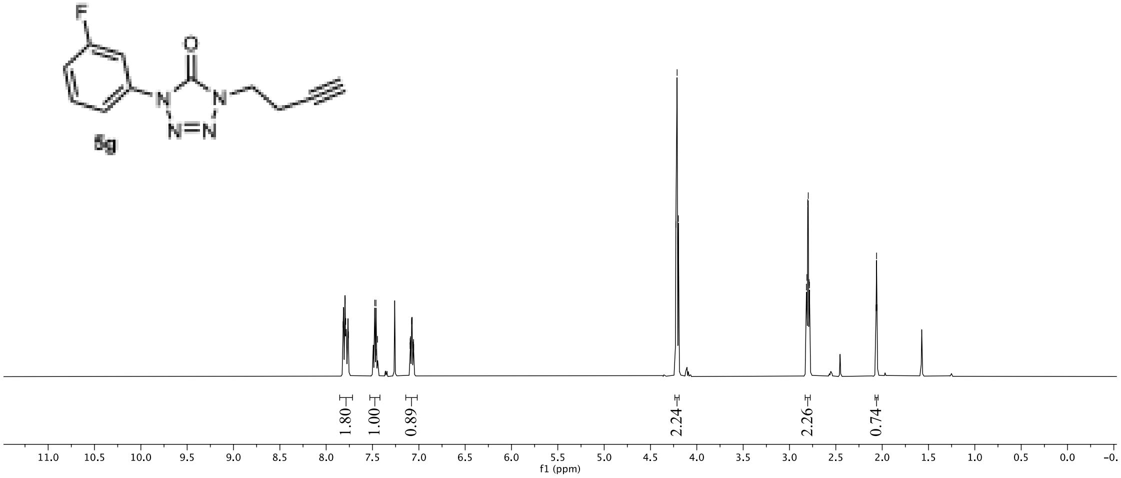

^{1}H NMR (500 MHz, $CDCl_3$)

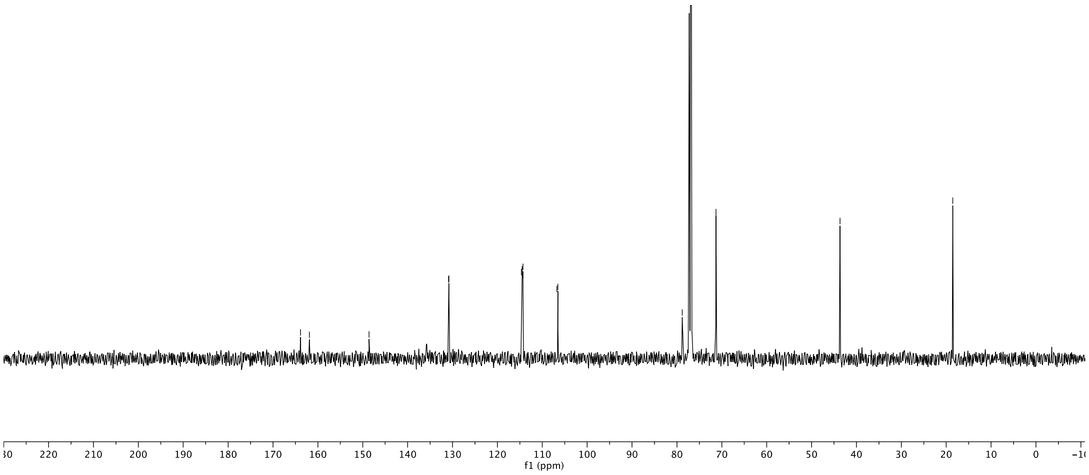

$^{13}C\{^1H\}$ NMR (126 MHz, $CDCl_3$)

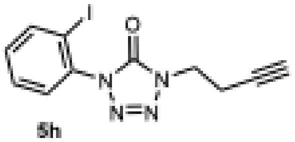

5h

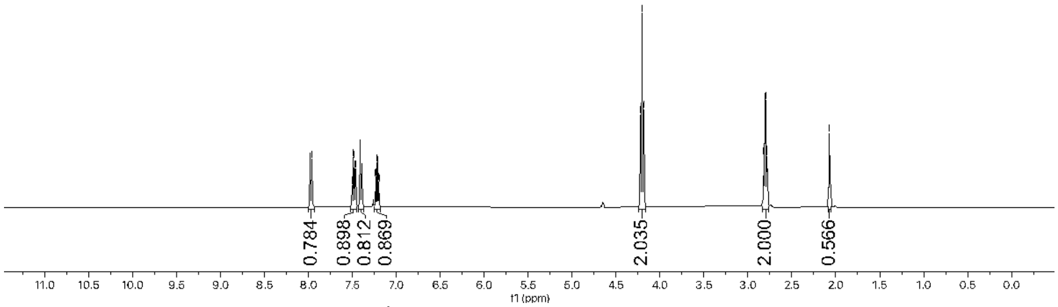

^{1}H NMR (500 MHz, $CDCl_3$)

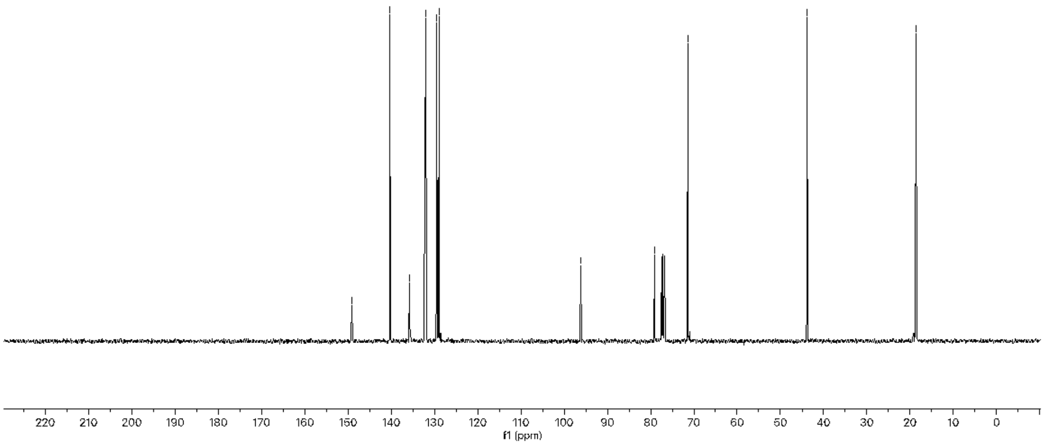

^{13}C{^{1}H} NMR (126 MHz, $CDCl_3$)

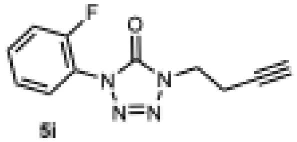

5i

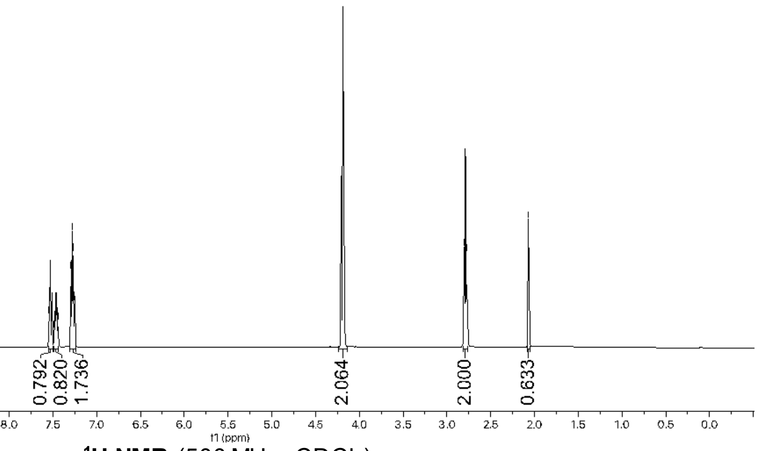

^{1}H NMR (500 MHz, $CDCl_3$)

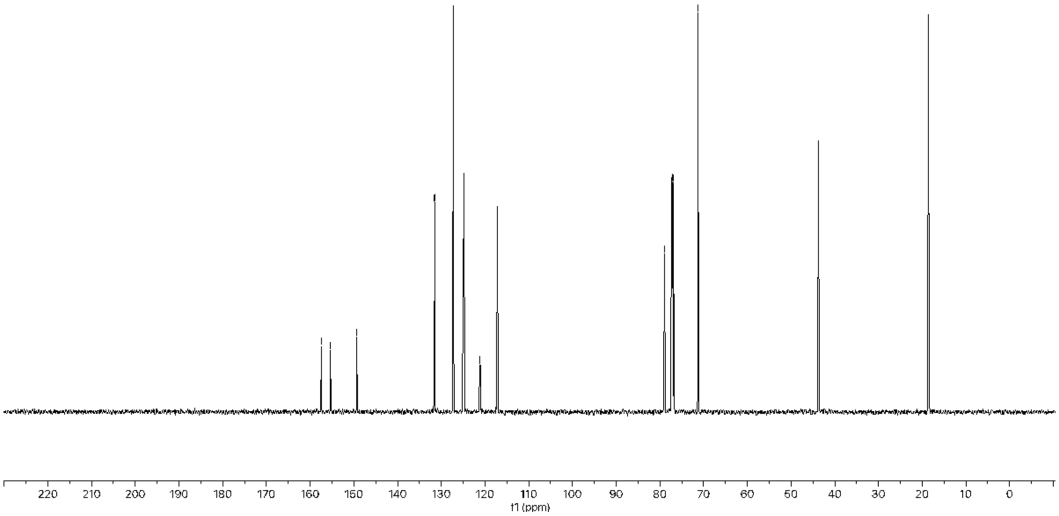

^{13}C{^{1}H} NMR (126 MHz, $CDCl_3$)

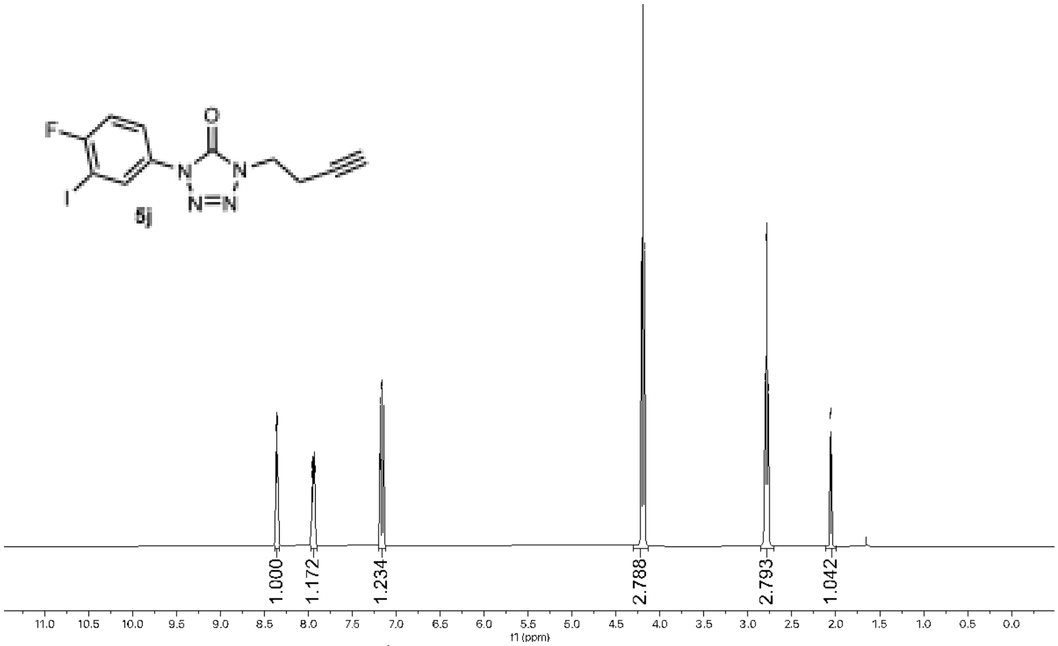

^{1}H NMR (500 MHz, $CDCl_3$)

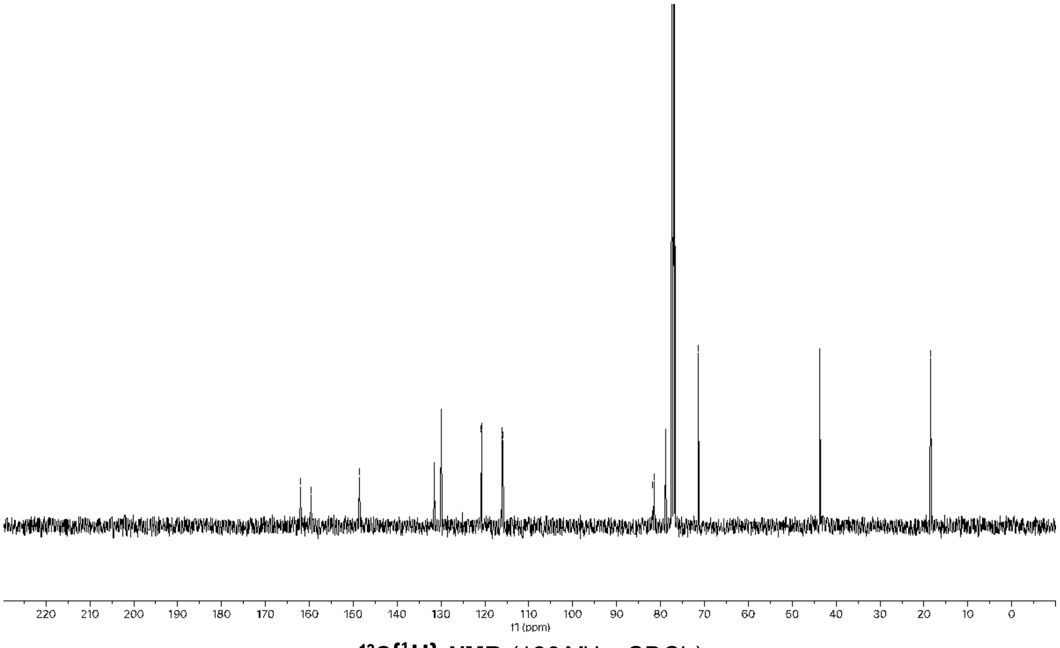

^{13}C{^{1}H} NMR (126 MHz, $CDCl_3$)

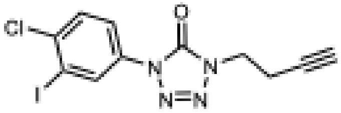

5k

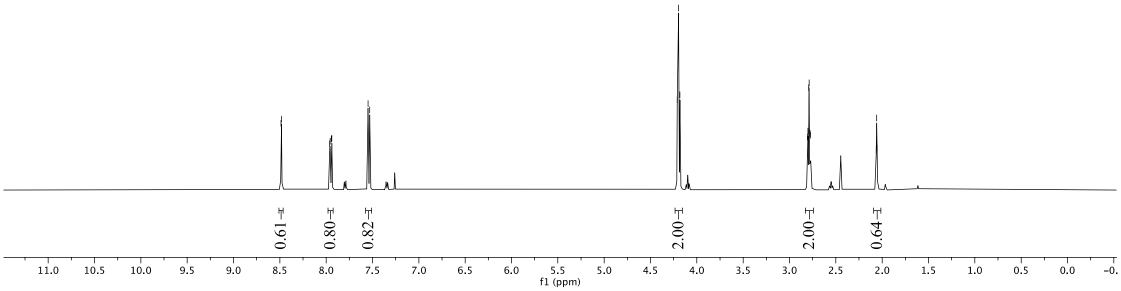

^{1}H NMR (500 MHz, $CDCl_3$)

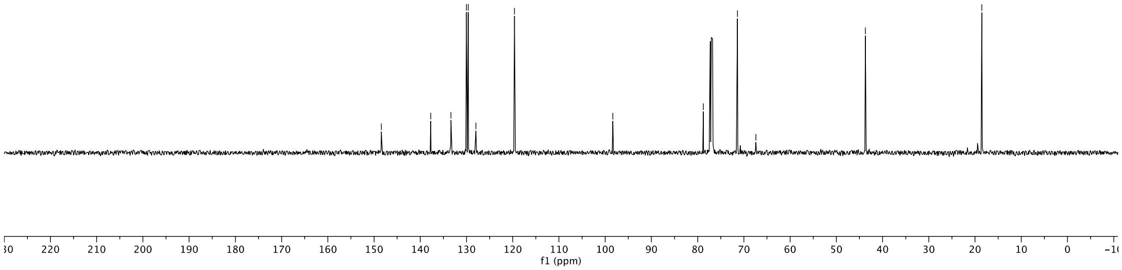

^{13}C{^{1}H} NMR (126 MHz, $CDCl_3$)

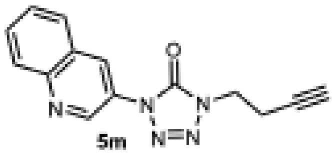

5m

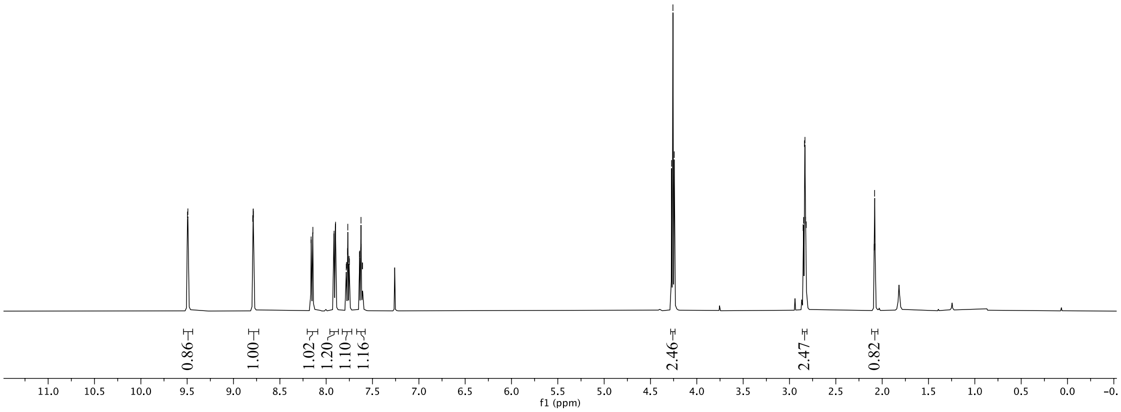

^{1}H NMR (500 MHz, $CDCl_3$)

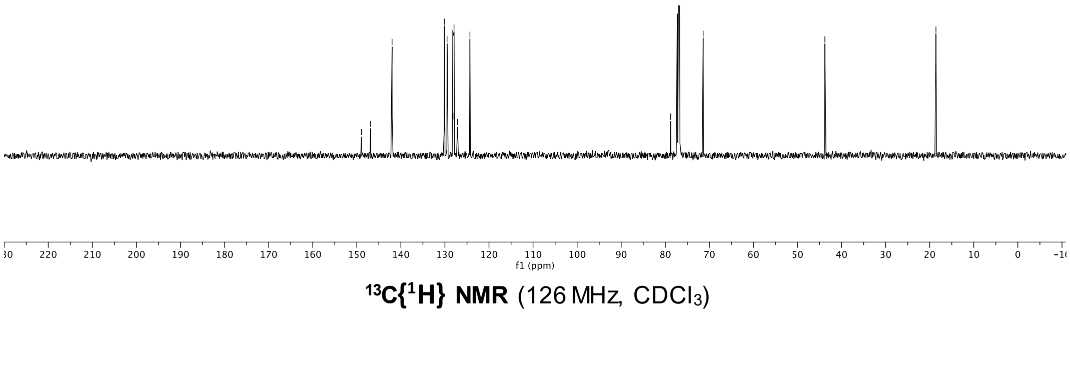

^{13}C{^{1}H} NMR (126 MHz, $CDCl_3$)

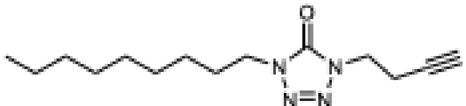

5n

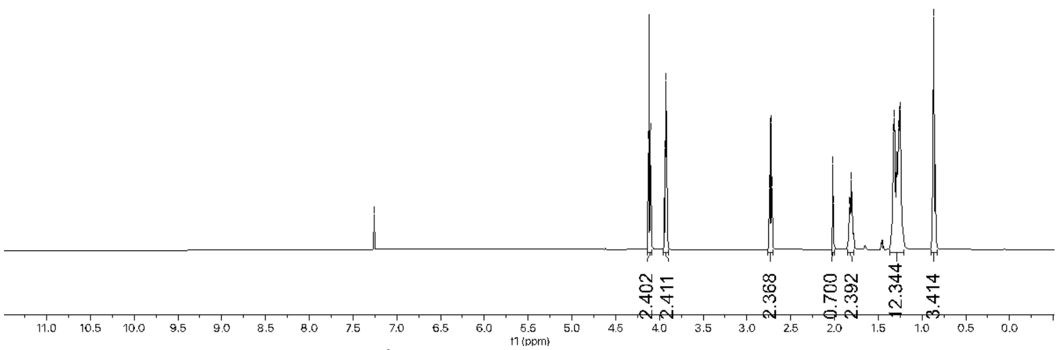

^{1}H NMR (500 MHz, $CDCl_3$)

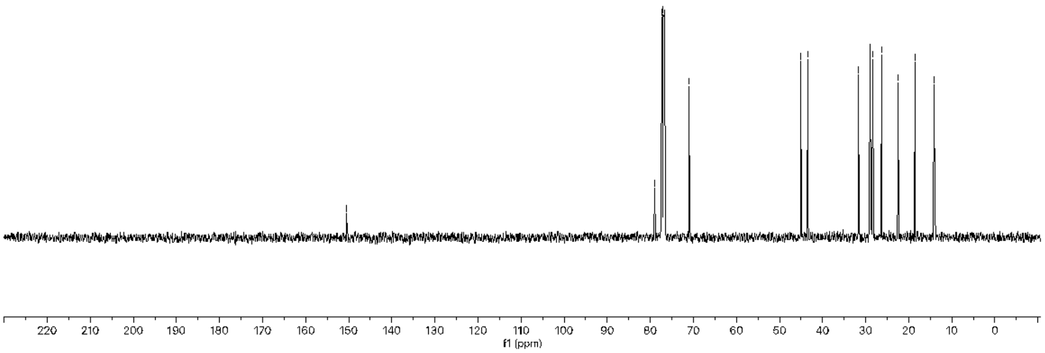

^{13}C{^{1}H} NMR (126 MHz, $CDCl_3$)

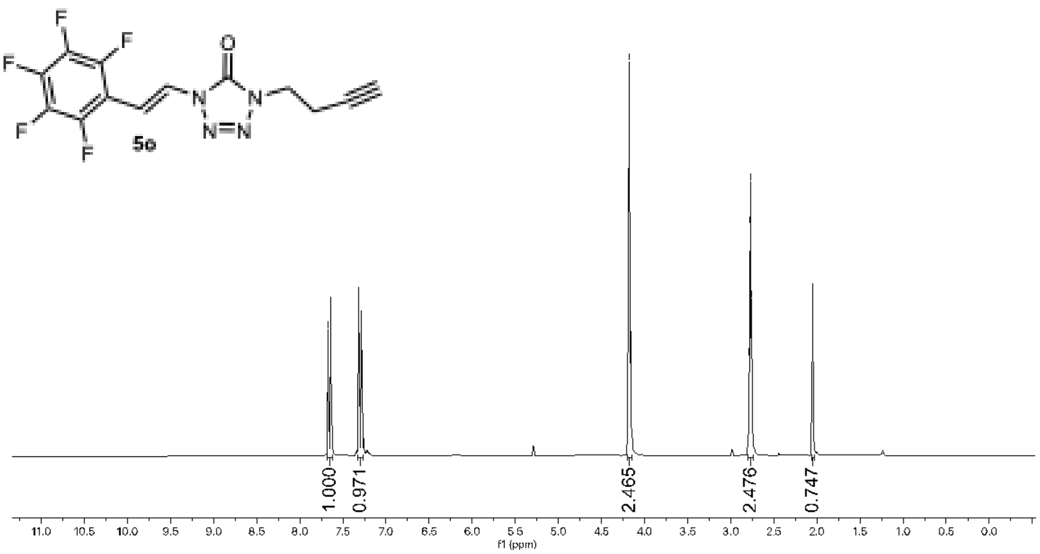

^{1}H NMR (500 MHz, $CDCl_3$)

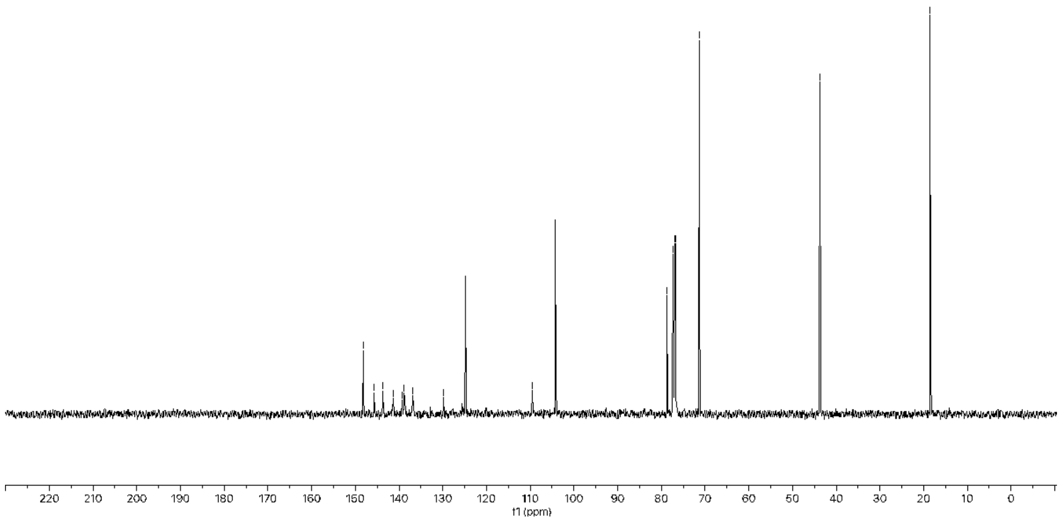

^{13}C{^{1}H} NMR (126 MHz, $CDCl_3$)

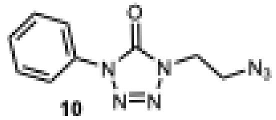

10

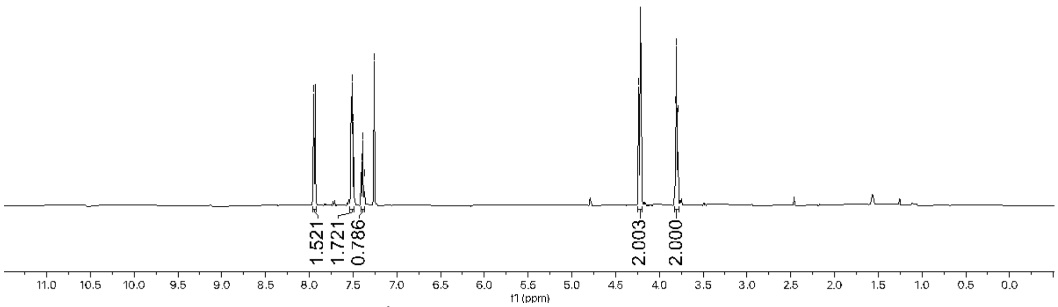

^{1}H NMR (500 MHz, $CDCl_3$)

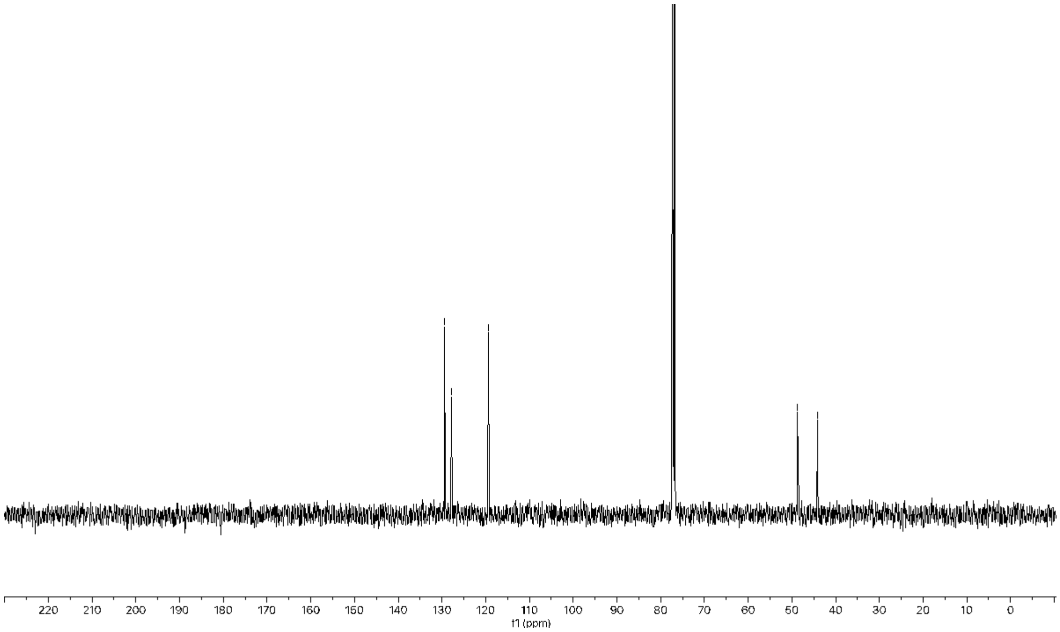

$^{13}C\{^1H\}$ NMR (126 MHz, $CDCl_3$)

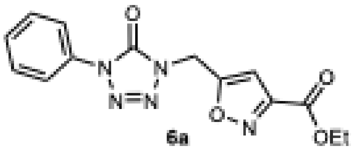

6a

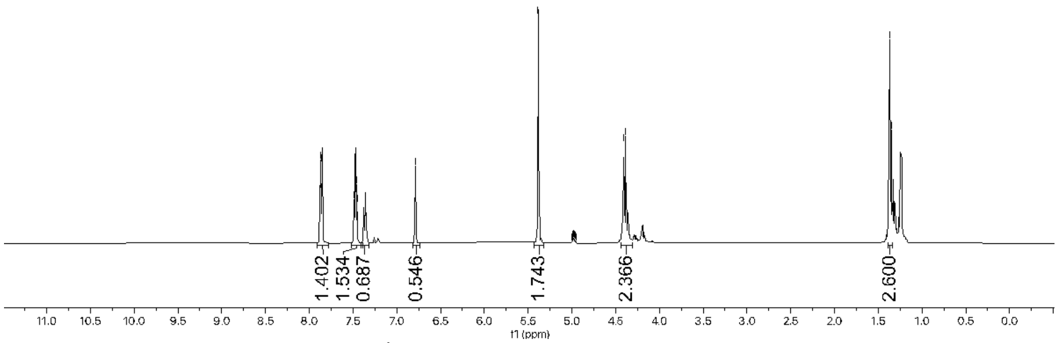

^{1}H NMR (500 MHz, $CDCl_3$)

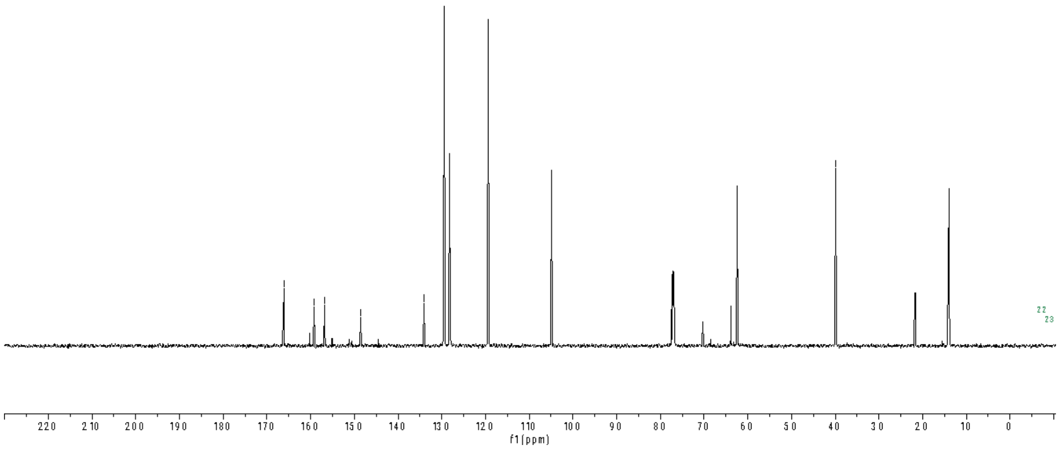

^{13}C{^{1}H} NMR (126 MHz, $CDCl_3$)

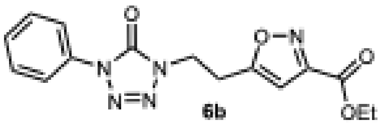

6b

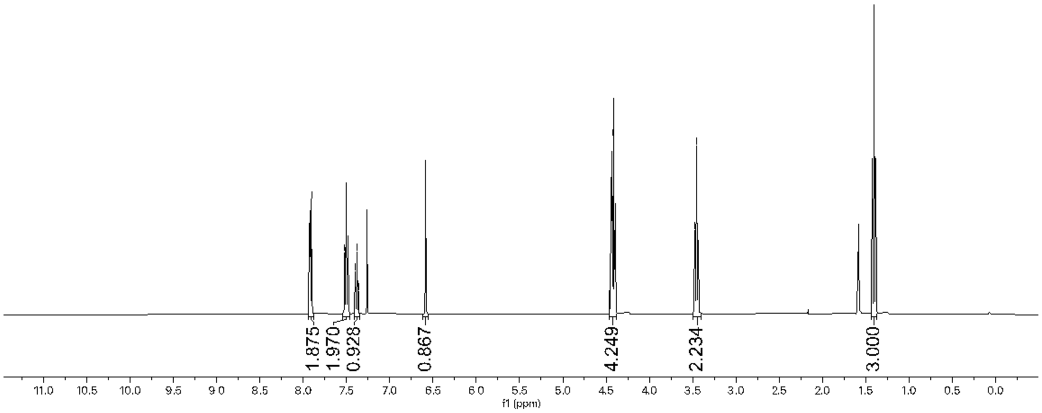

^{1}H NMR (500 MHz, $CDCl_3$)

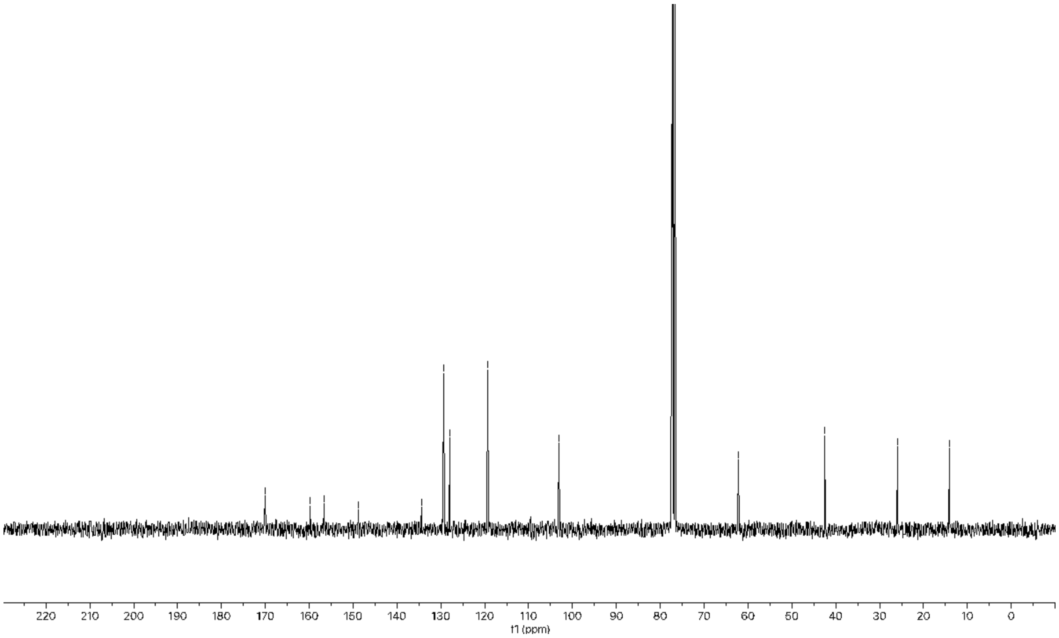

^{13}C{^{1}H} NMR (126 MHz, $CDCl_3$)

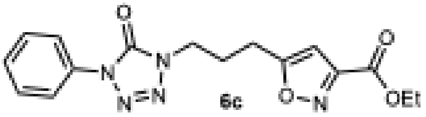

6c

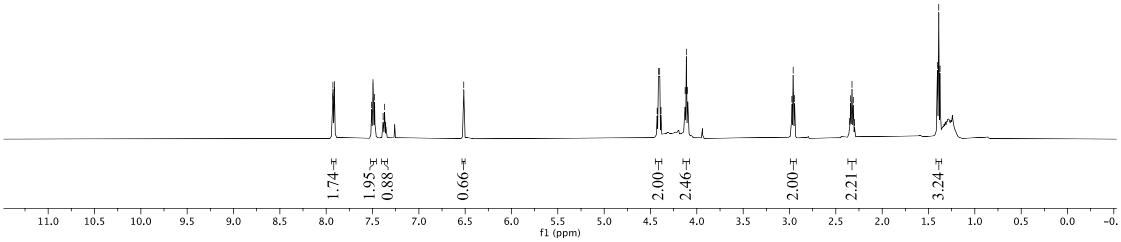

^{1}H NMR (500 MHz, $CDCl_3$)

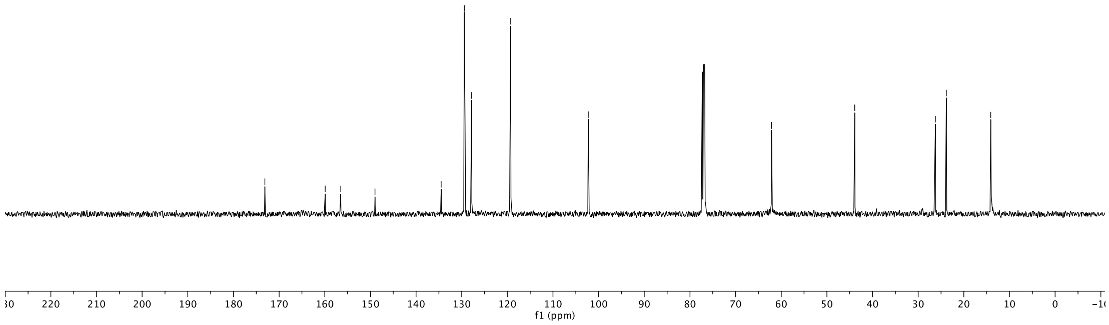

^{13}C{^{1}H} NMR (126 MHz, $CDCl_3$)

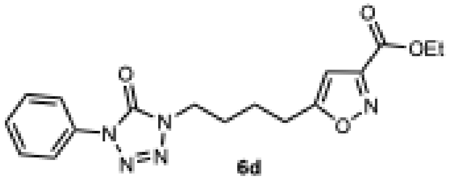

6d

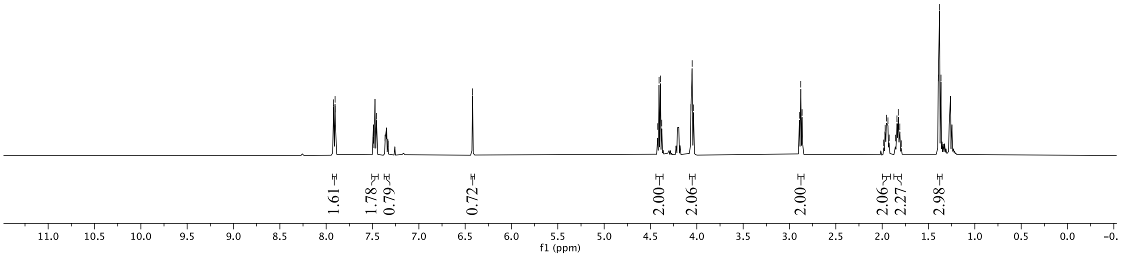

^{1}H NMR (500 MHz, $CDCl_3$)

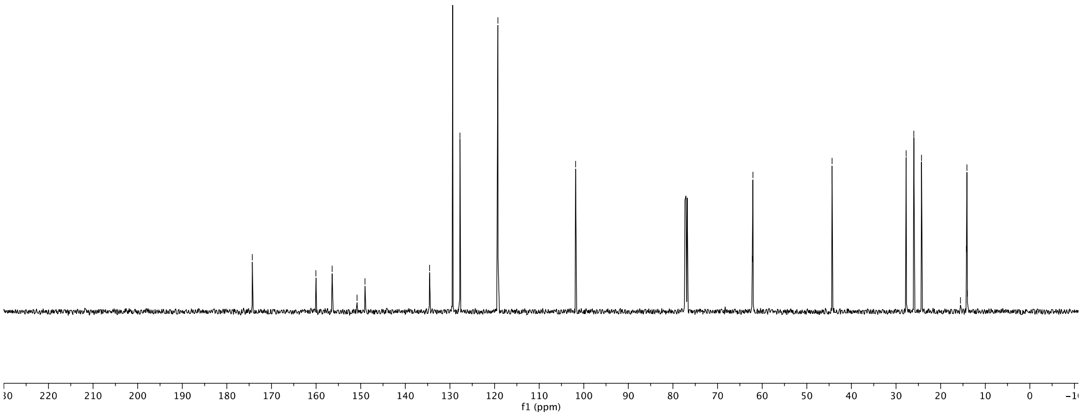

$^{13}C\{^1H\}$ NMR (126 MHz, $CDCl_3$)

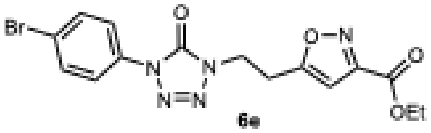

6e

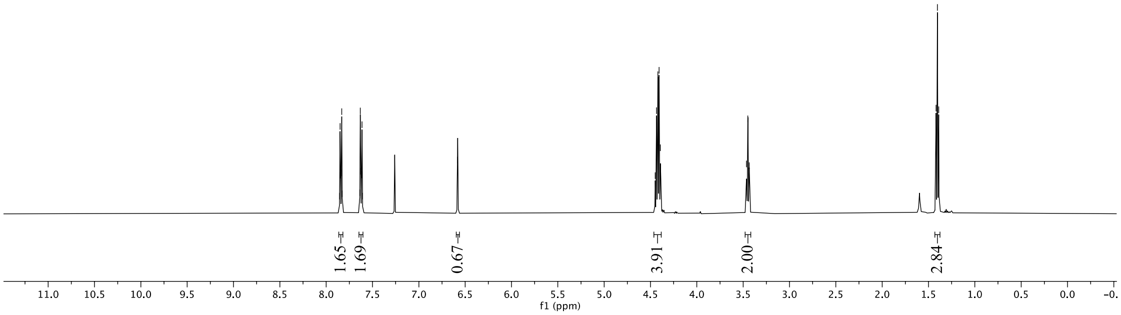

^{1}H NMR (500 MHz, $CDCl_3$)

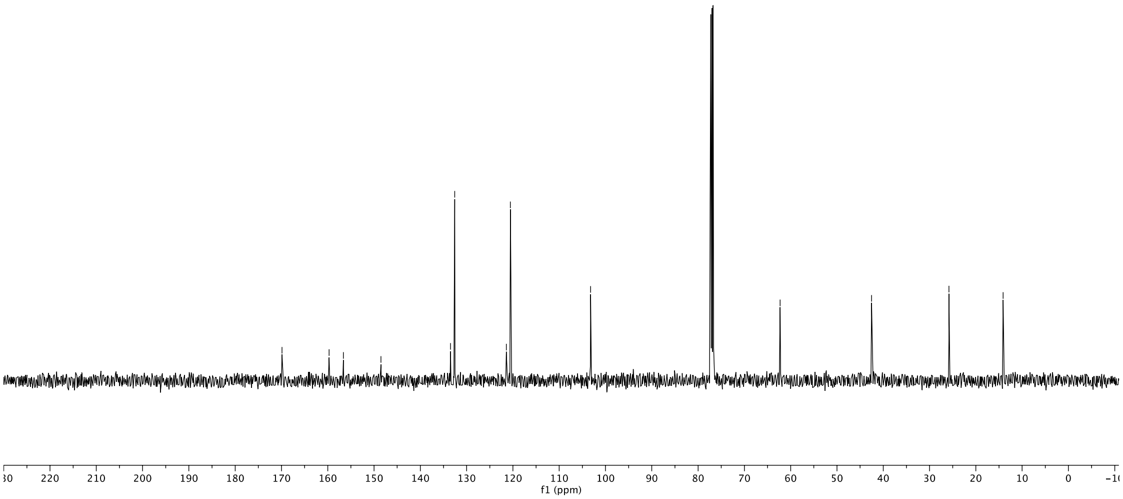

$^{13}C\{^1H\}$ NMR (126 MHz, $CDCl_3$)

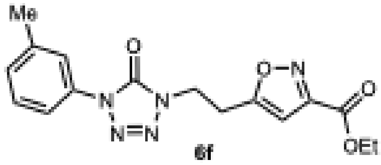

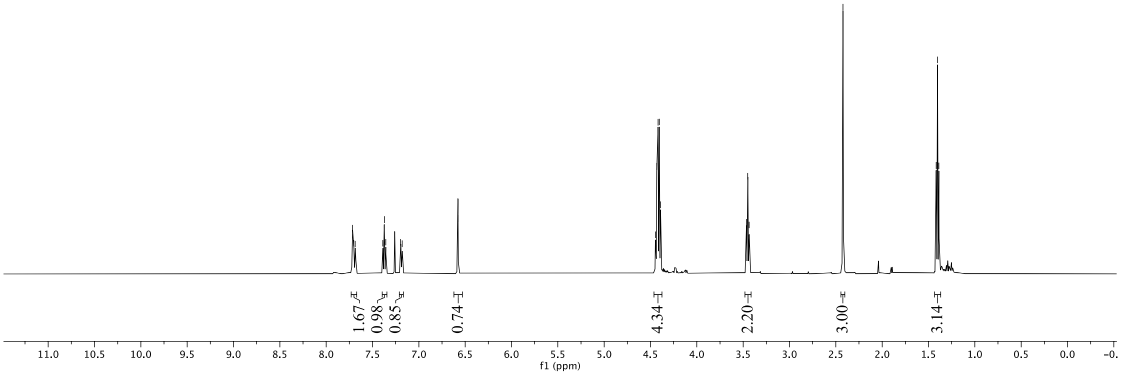

^{1}H NMR (500 MHz, $CDCl_3$)

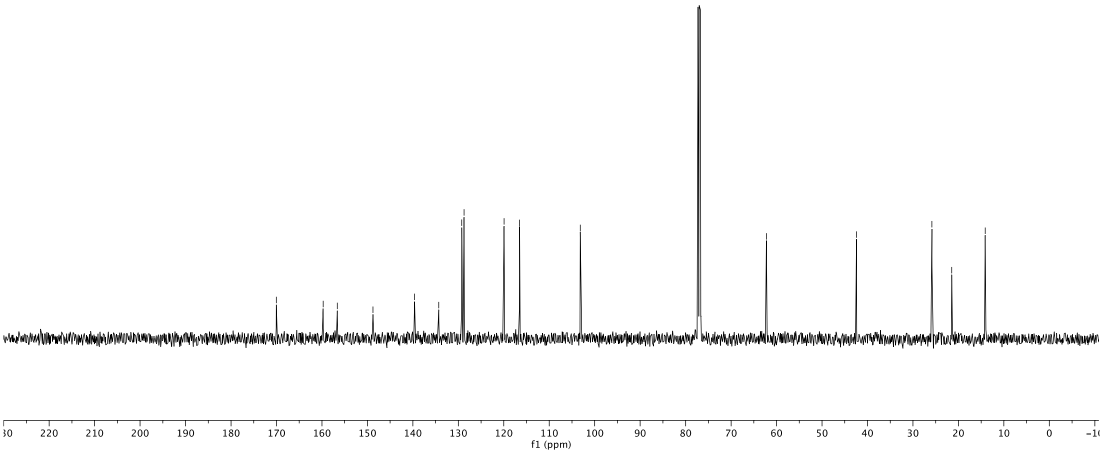

^{13}C{^{1}H} NMR (126 MHz, $CDCl_3$)

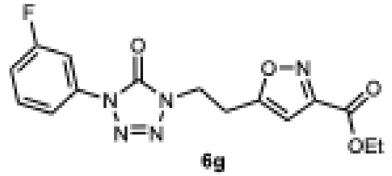

6g

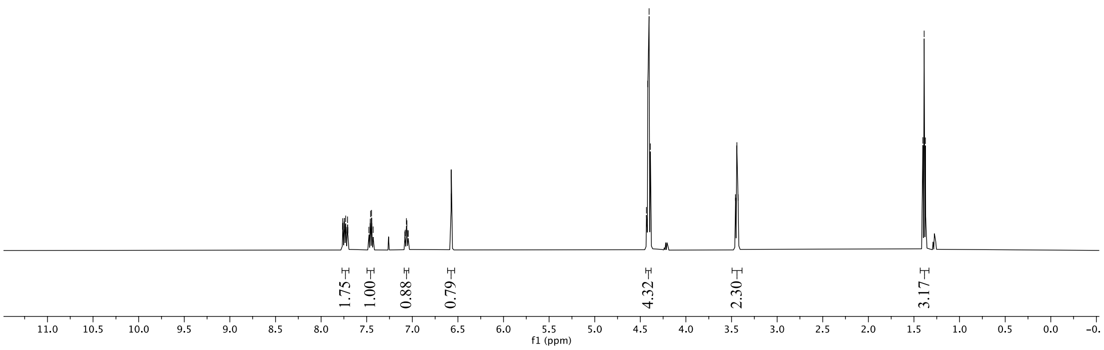

¹H NMR (500 MHz, $CDCl_3$)

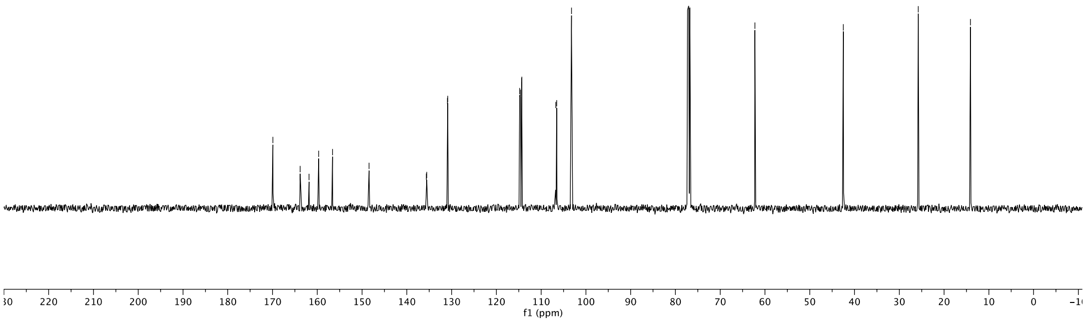

$^{13}C\{^1H\}$ NMR (126 MHz, $CDCl_3$)

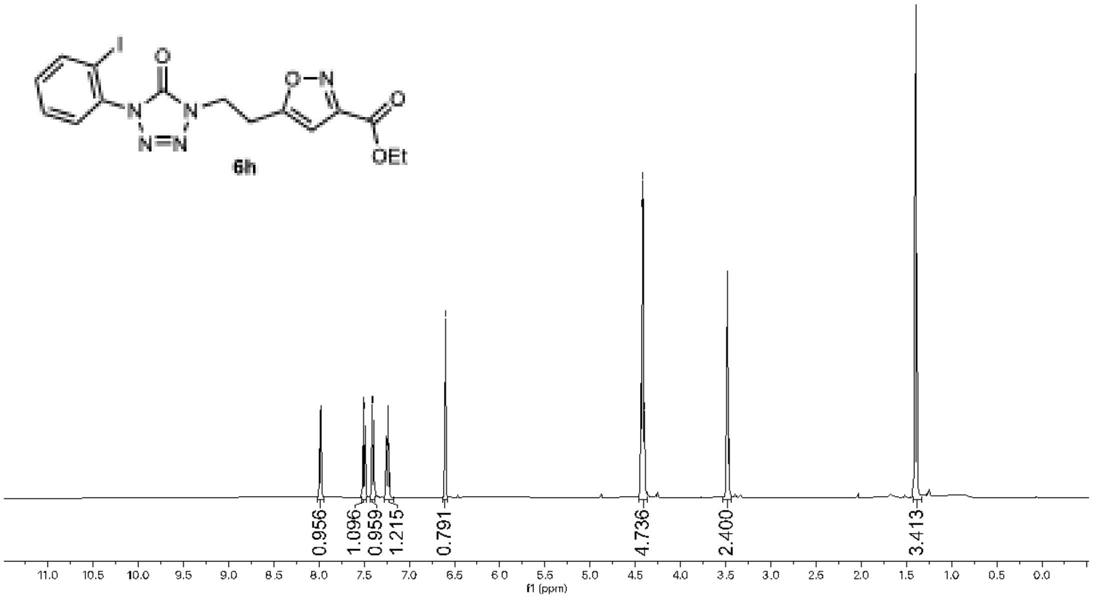

^{1}H NMR (500 MHz, $CDCl_3$)

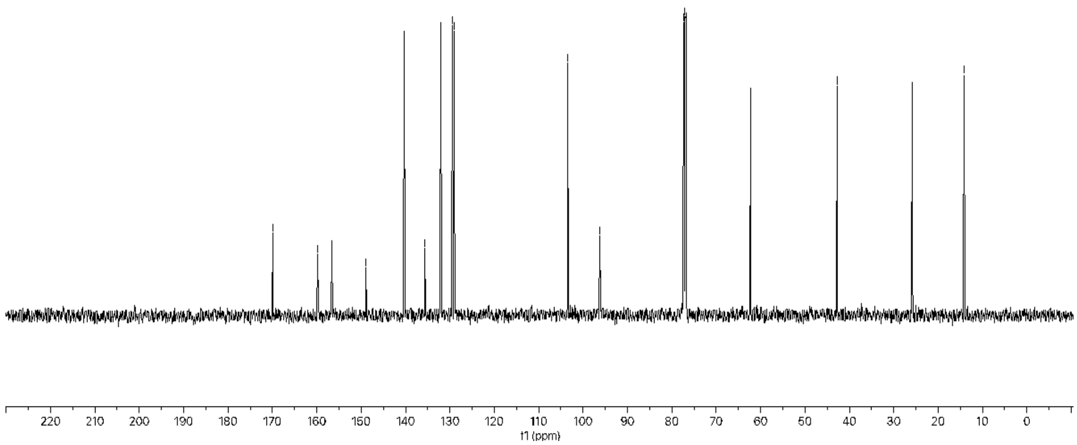

$^{13}C\{^{1}H\}$ NMR (126 MHz, $CDCl_3$)

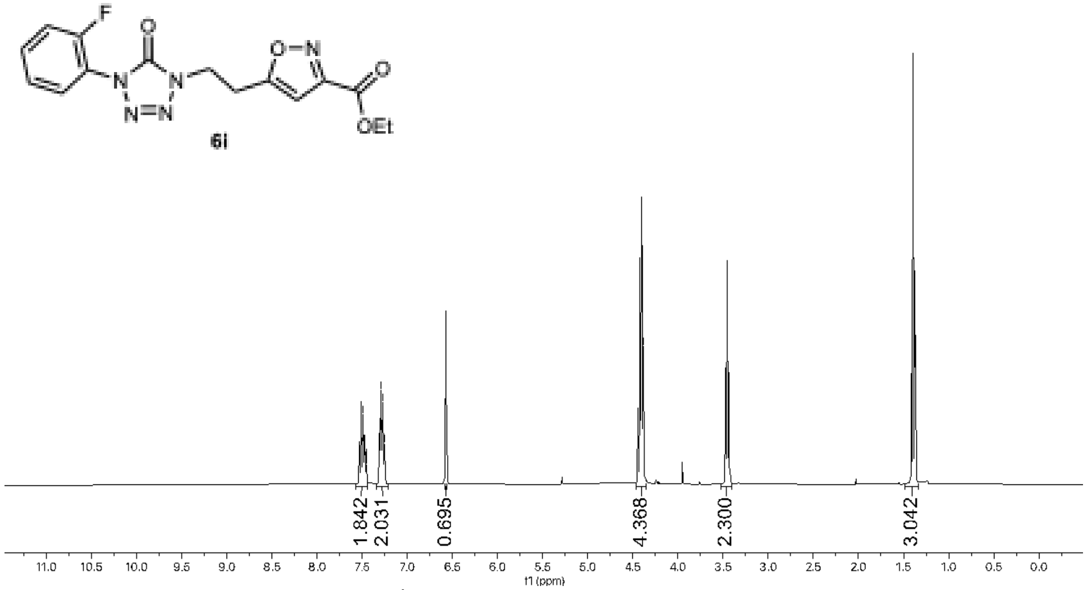

^{1}H NMR (500 MHz, $CDCl_3$)

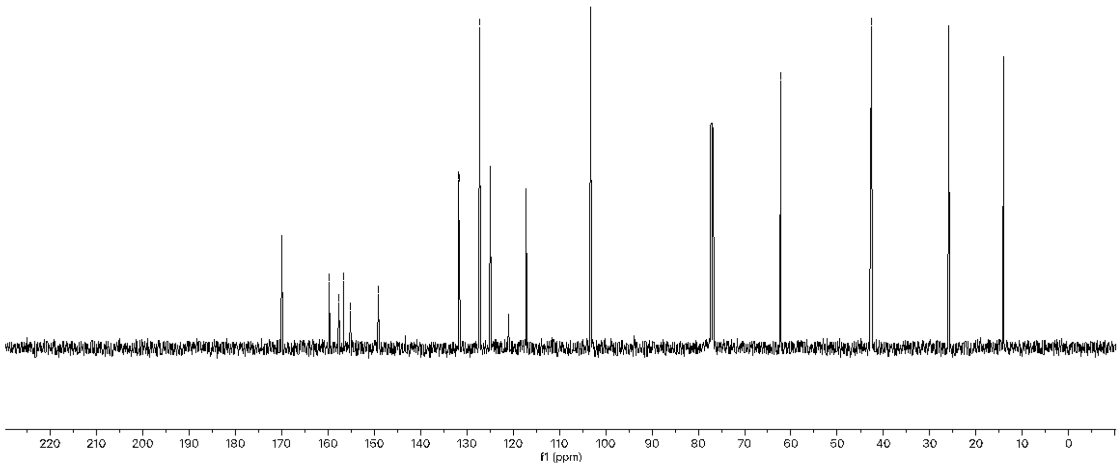

^{13}C{^{1}H} NMR (126 MHz, $CDCl_3$)

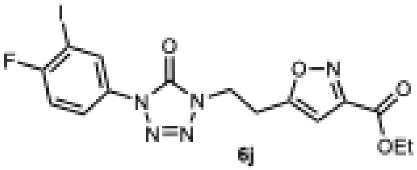

6j

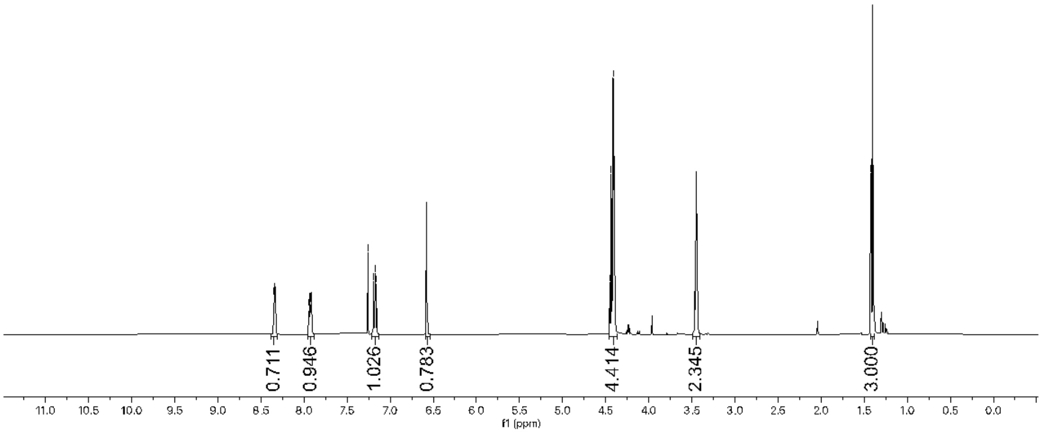

^{1}H NMR (500 MHz, $CDCl_3$)

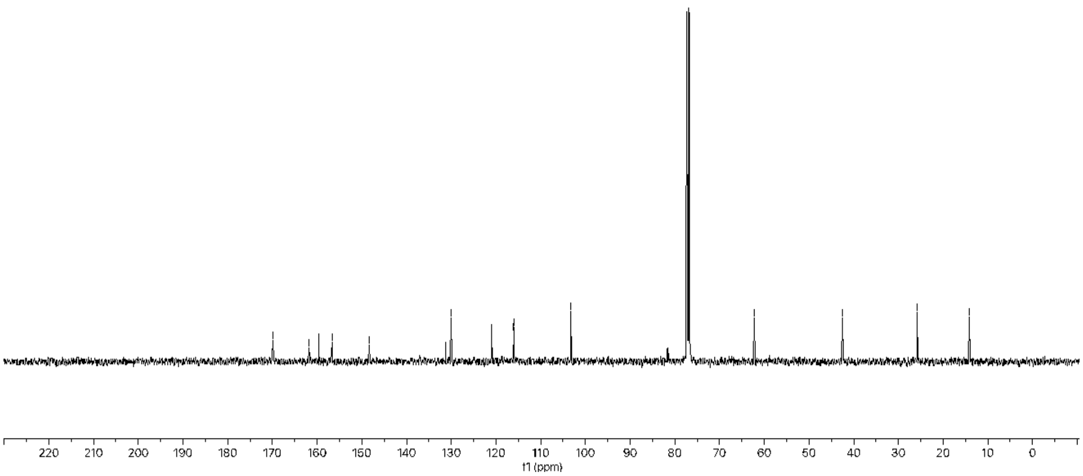

$^{13}C\{^1H\}$ NMR (126 MHz, $CDCl_3$)

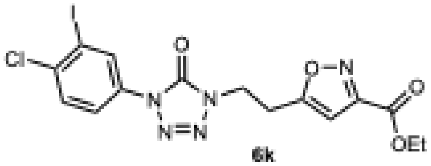

6k

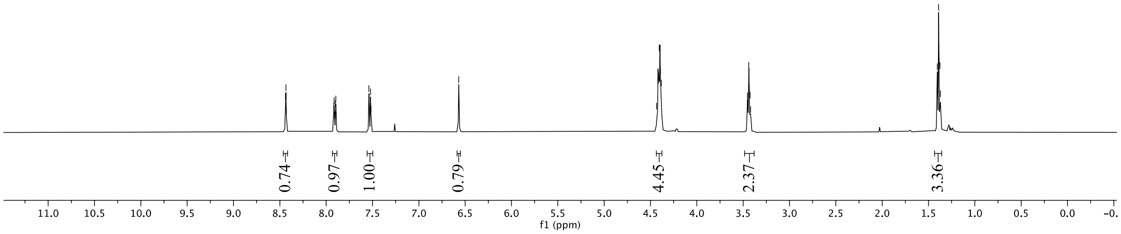

^{1}H NMR (500 MHz, $CDCl_3$)

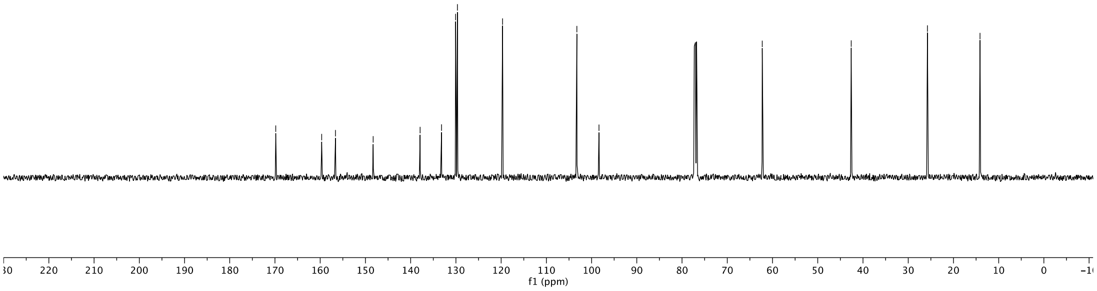

^{13}C{^{1}H} NMR (126 MHz, $CDCl_3$)

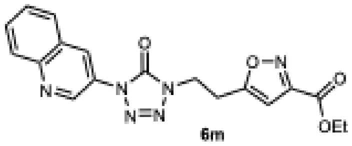

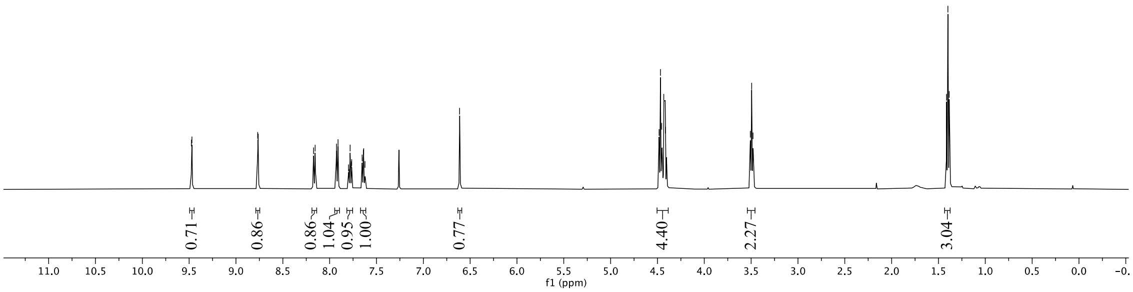

^{1}H NMR (500 MHz, $CDCl_3$)

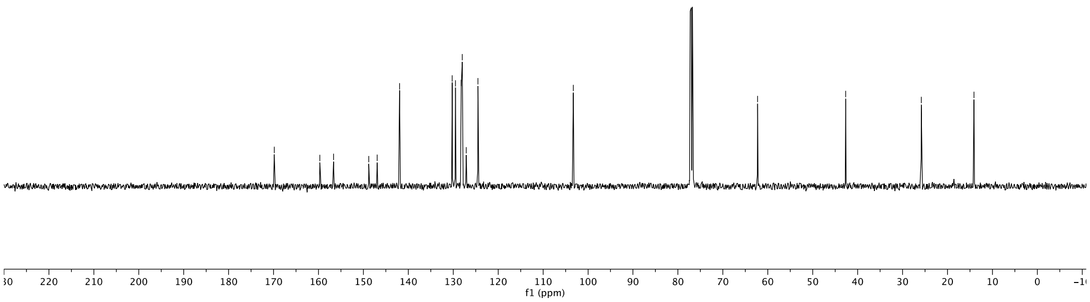

^{13}C{^{1}H} NMR (126 MHz, $CDCl_3$)

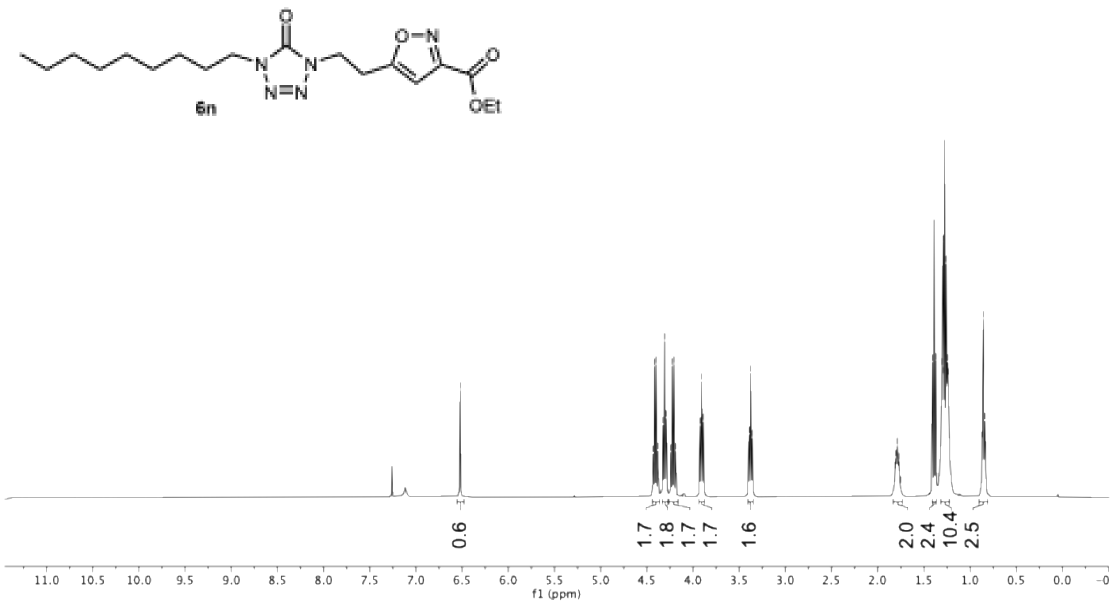

¹H NMR (500 MHz, $CDCl_3$)

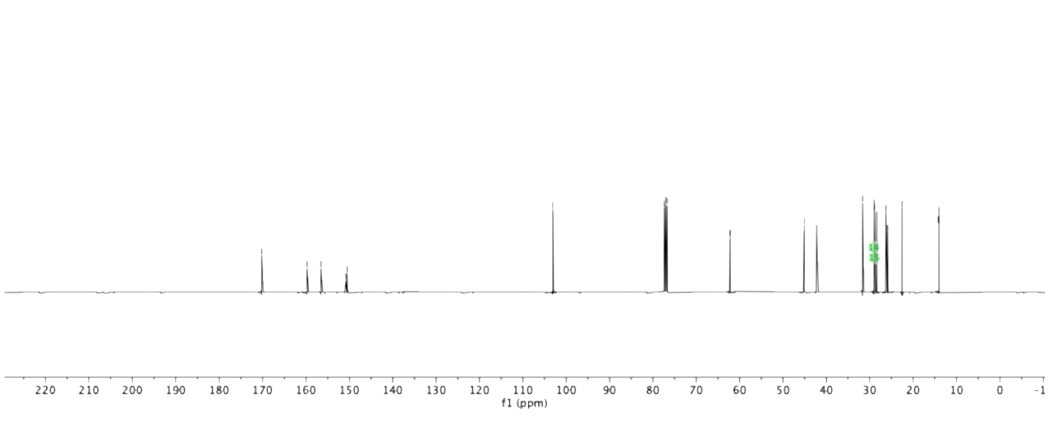

$^{13}C\{^1H\}$ NMR (126 MHz, $CDCl_3$)

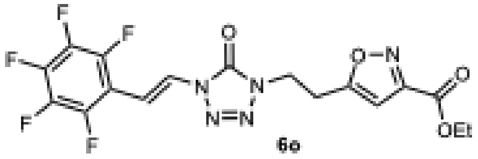

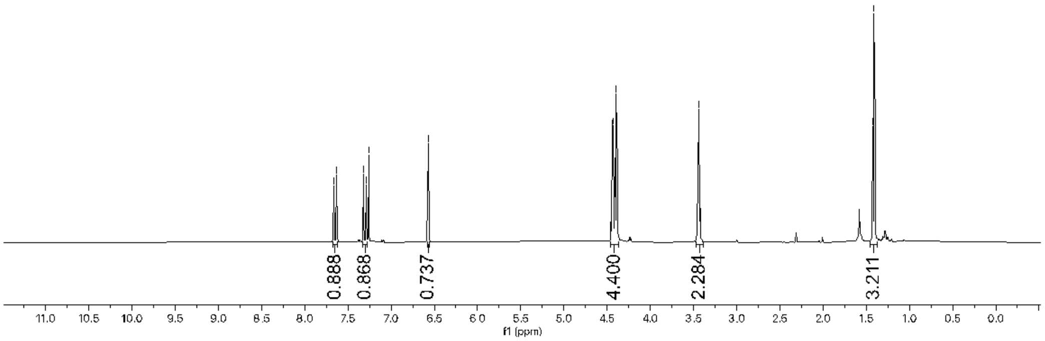

^{1}H NMR (500 MHz, $CDCl_3$)

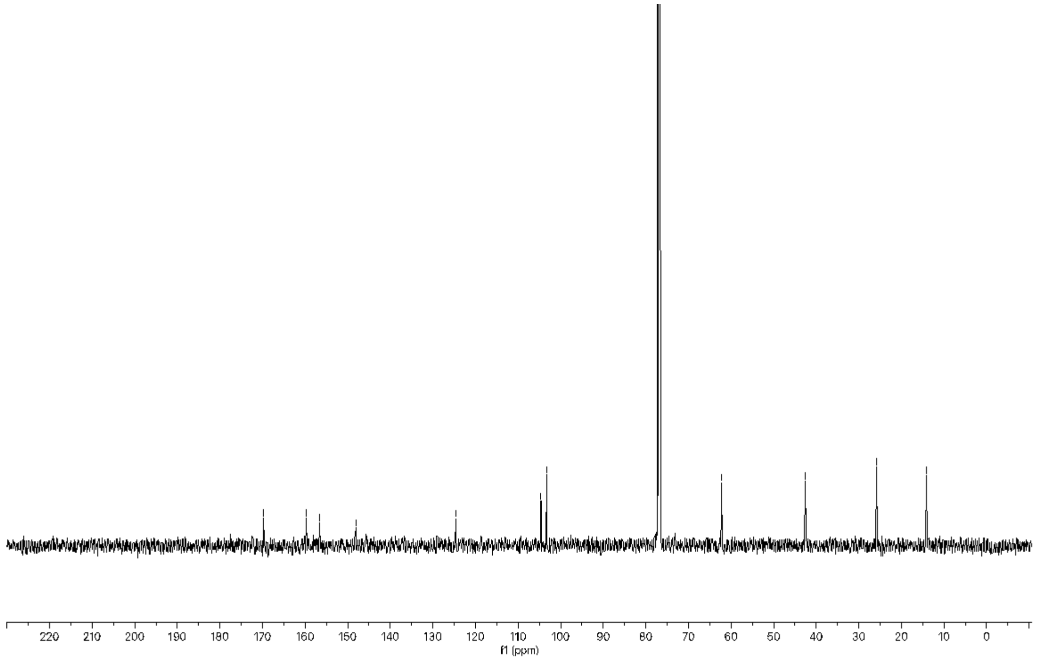

$^{13}C\{^1H\}$ NMR (126 MHz, $CDCl_3$)

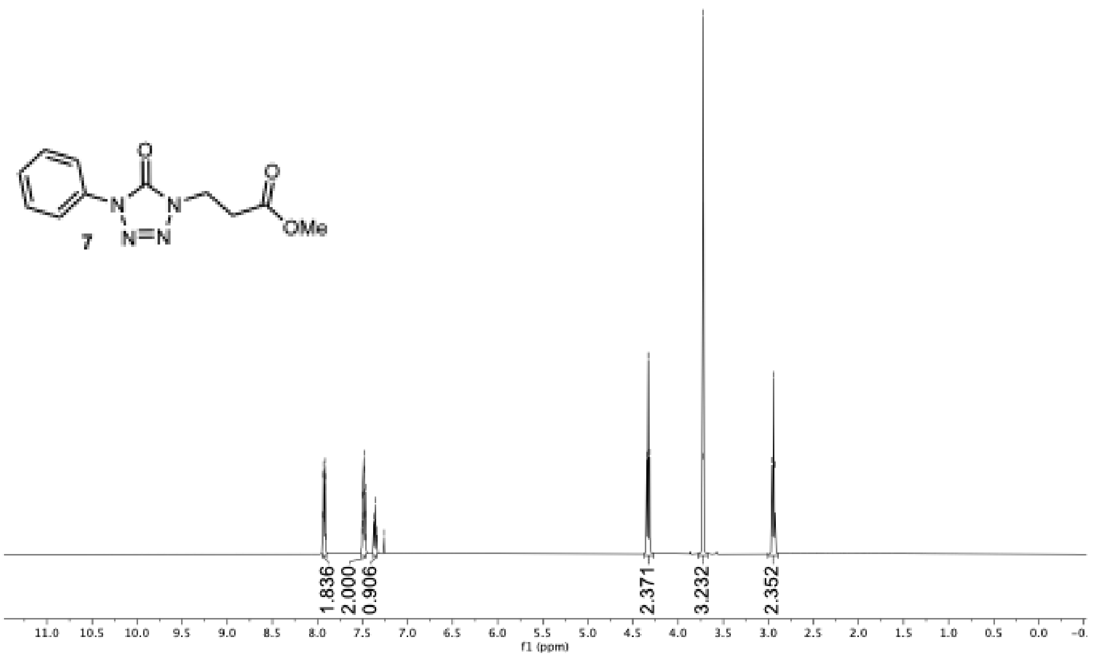

¹H NMR (500 MHz, $CDCl_3$)

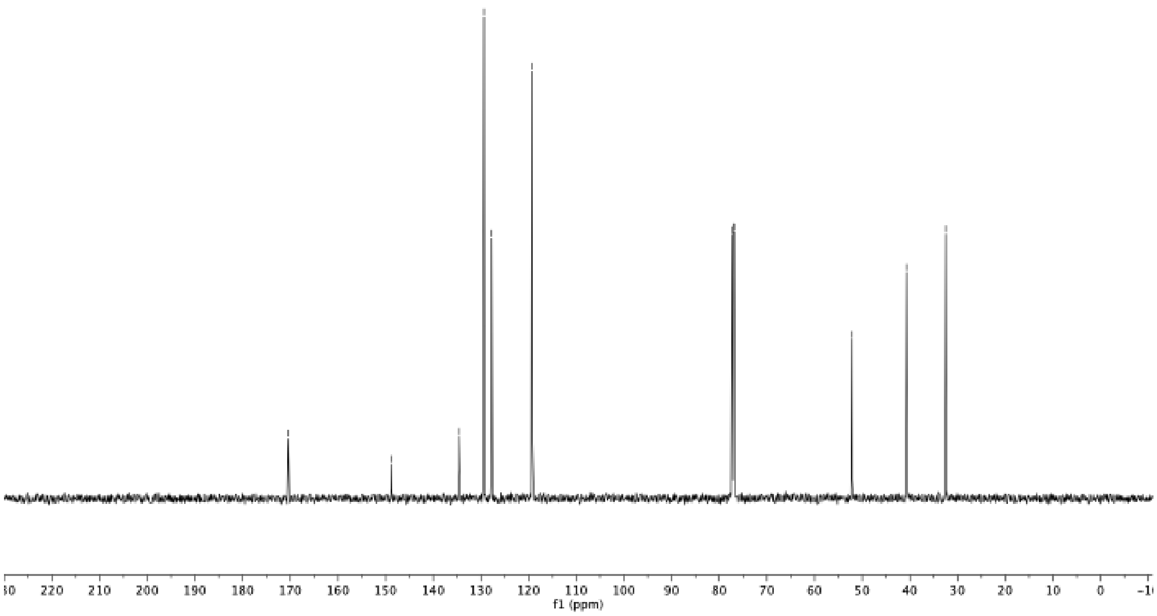

$^{13}C\{^1H\}$ NMR (126 MHz, $CDCl_3$)

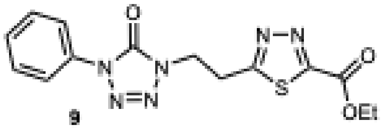

9

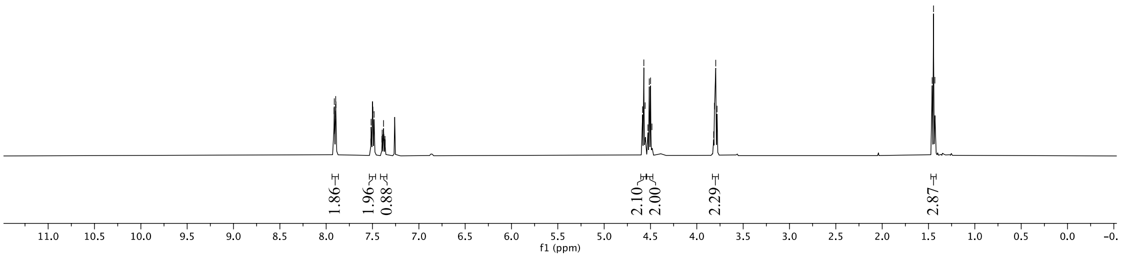

^{1}H NMR (500 MHz, $CDCl_3$)

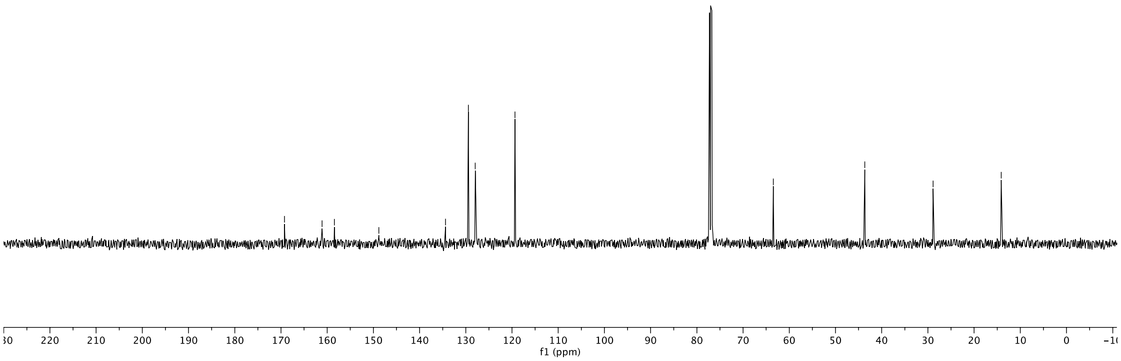

^{13}C{^{1}H} NMR (126 MHz, $CDCl_3$)

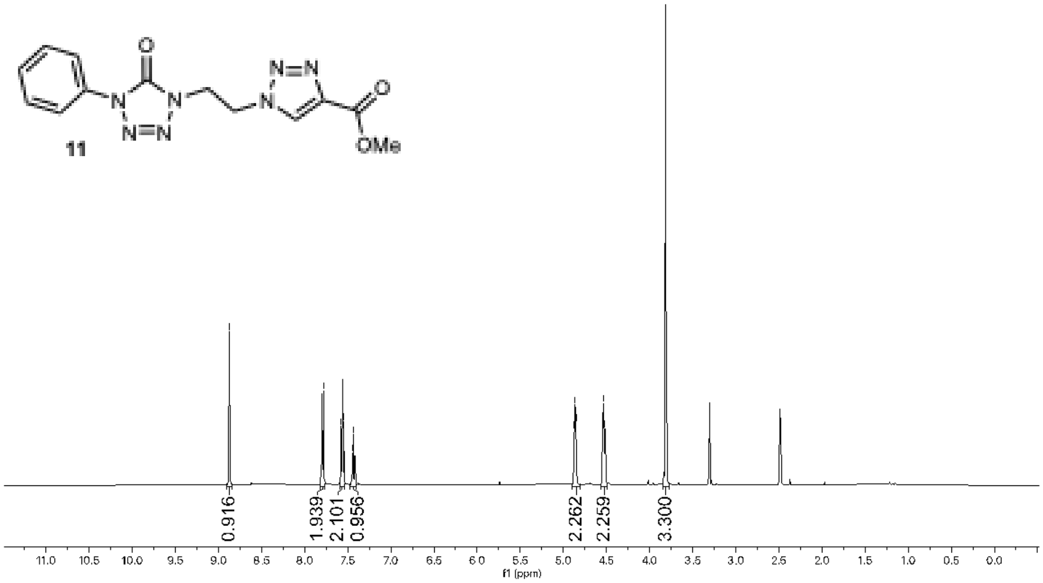

^{1}H NMR (500 MHz, $CDCl_3$)

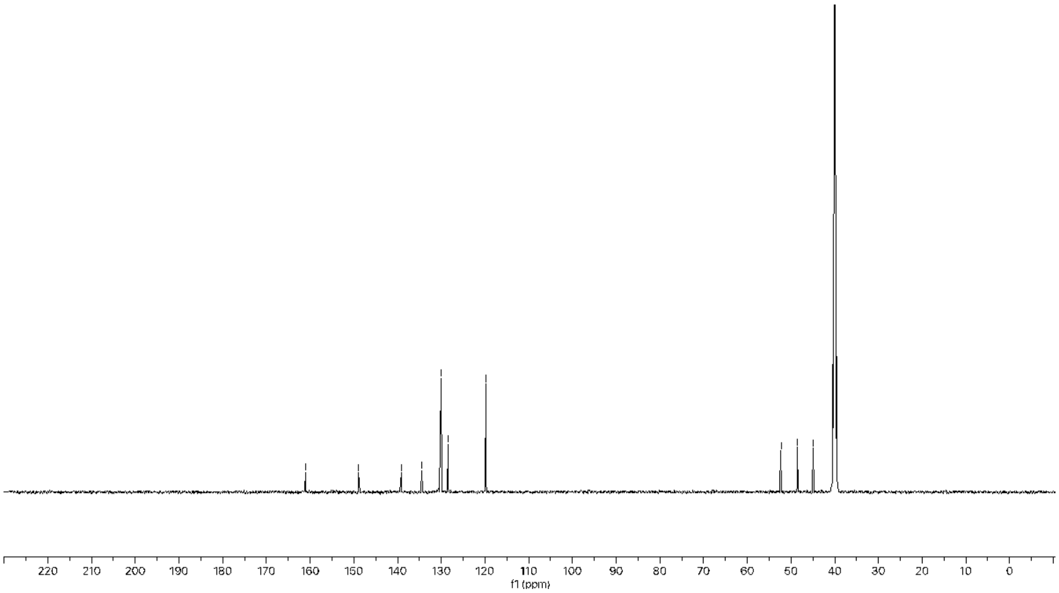

$^{13}C\{^{1}H\}$ NMR (126 MHz, $CDCl_3$)

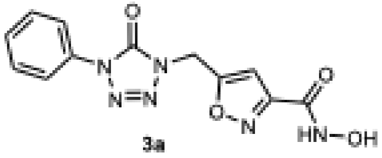

3a

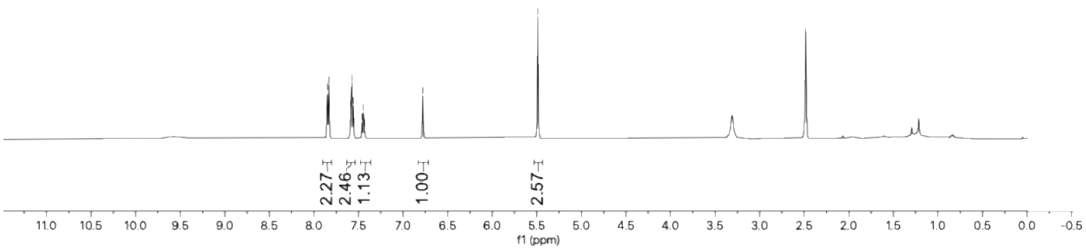

^{1}H NMR (500 MHz, $CDCl_3$)

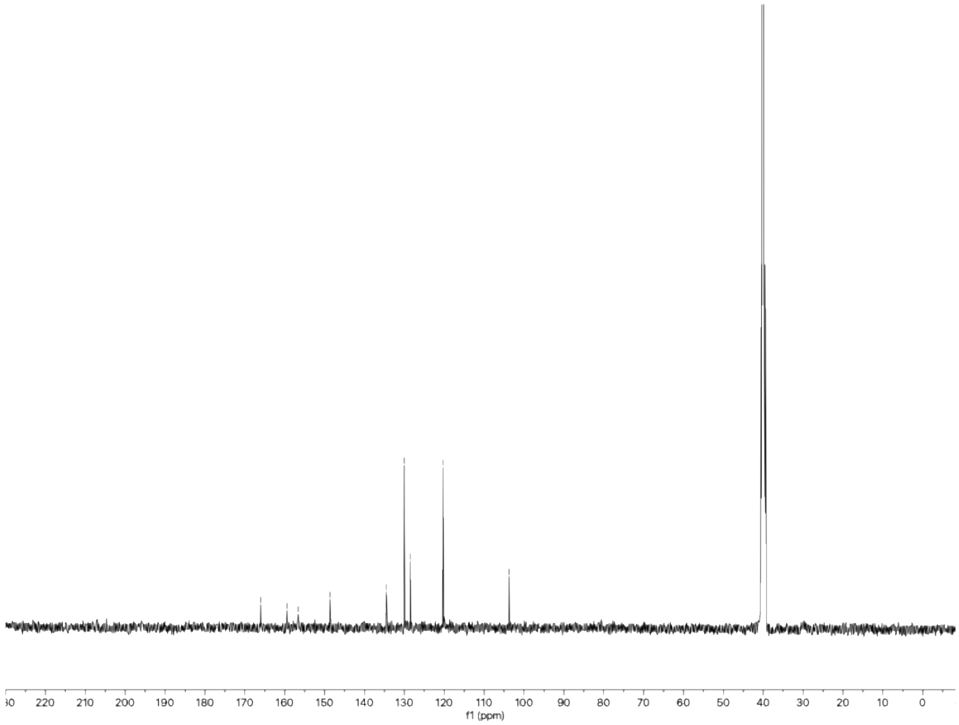

$^{13}C\{^1H\}$ NMR (126 MHz, $CDCl_3$)

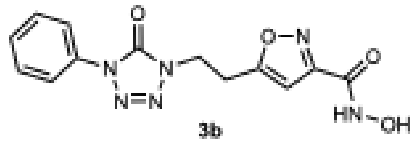

3b

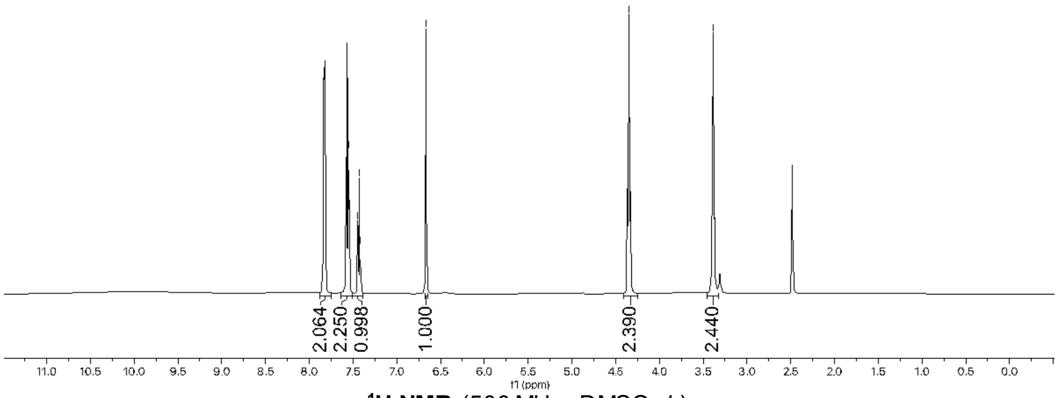

^{1}H NMR (500 MHz, DMSO-d_6)

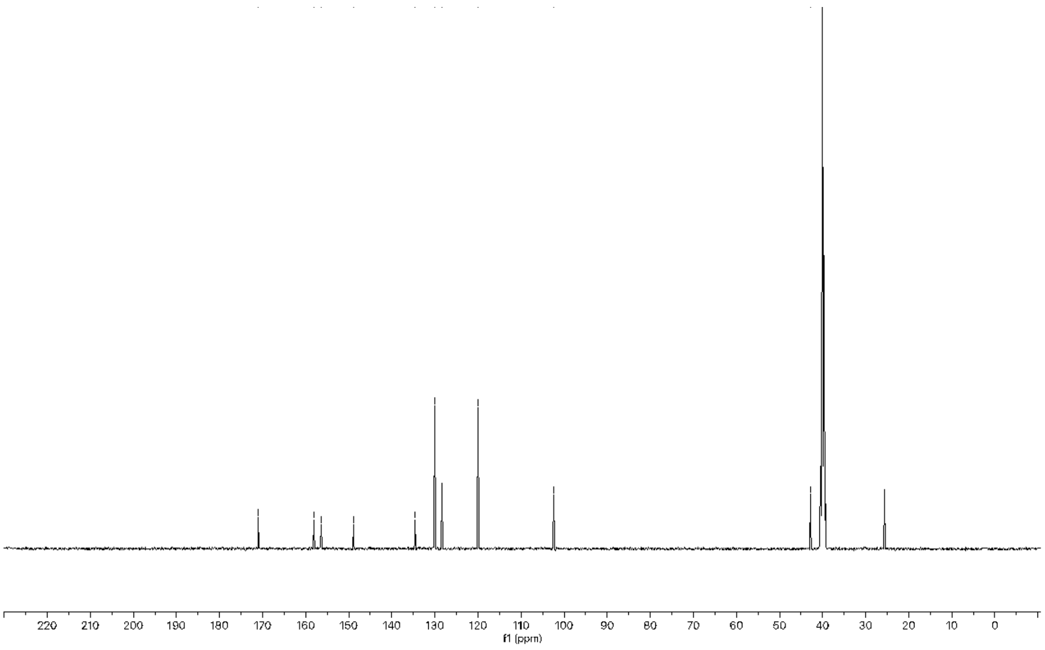

^{13}C{^{1}H} NMR (126 MHz, DMSO-d_6)

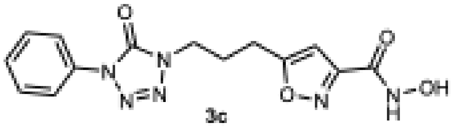

3c

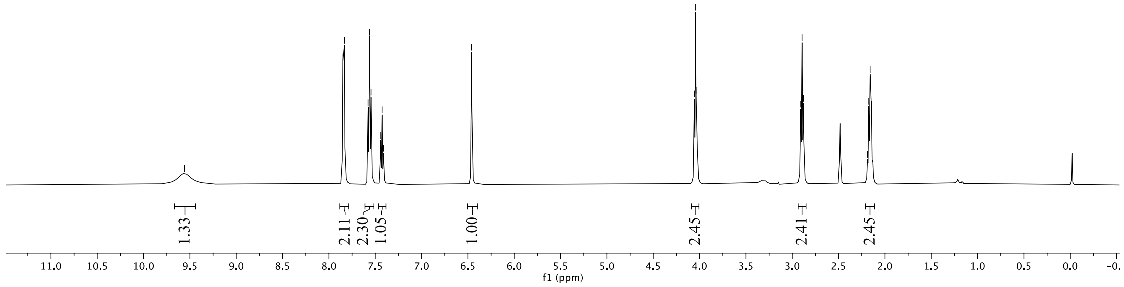

^{1}H NMR (500 MHz, DMSO-d_6)

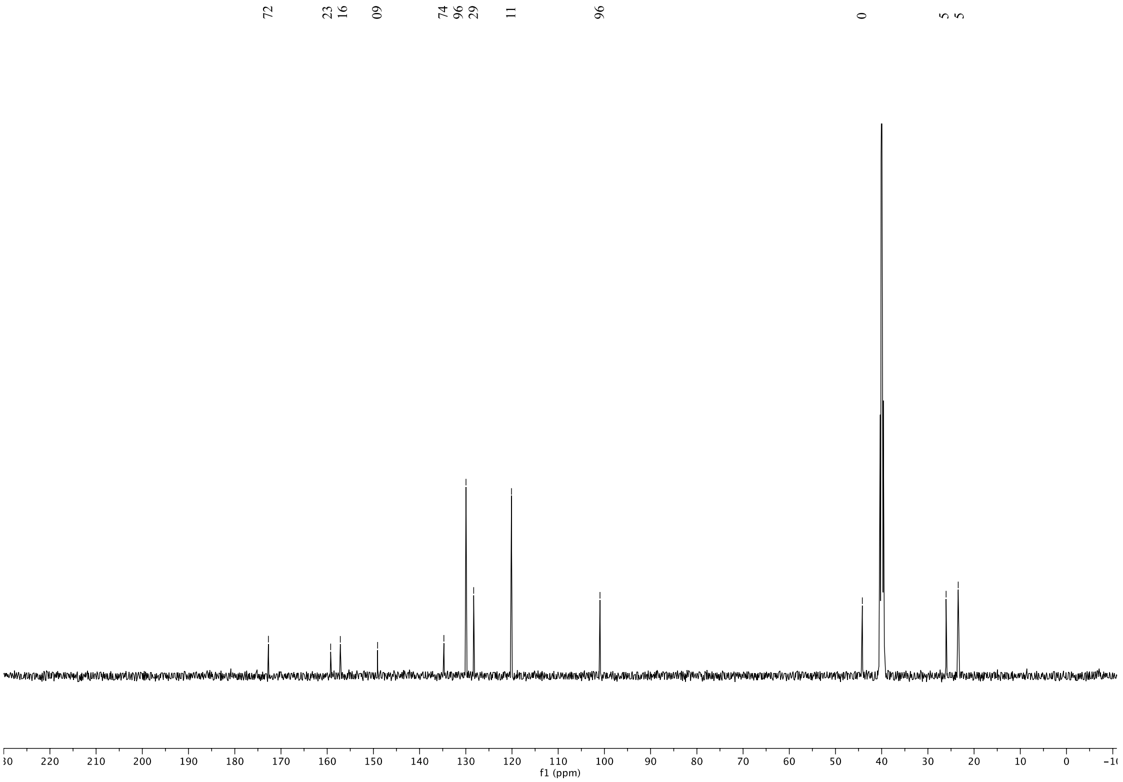

^{13}C{^{1}H} NMR (126 MHz, DMSO-d_6)

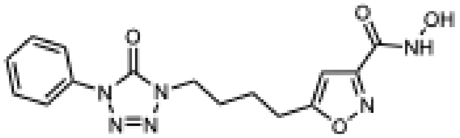

3d

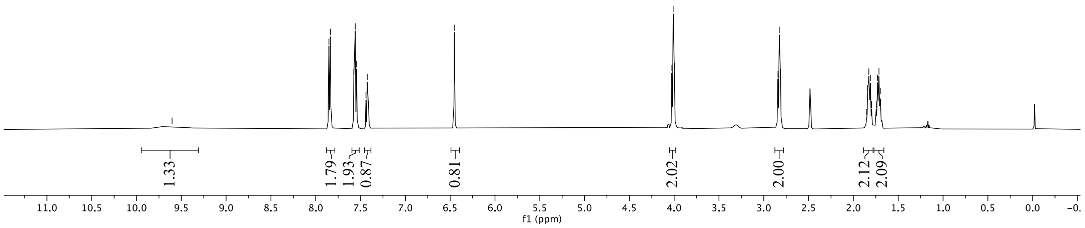

^{1}H NMR (500 MHz, DMSO-d_6)

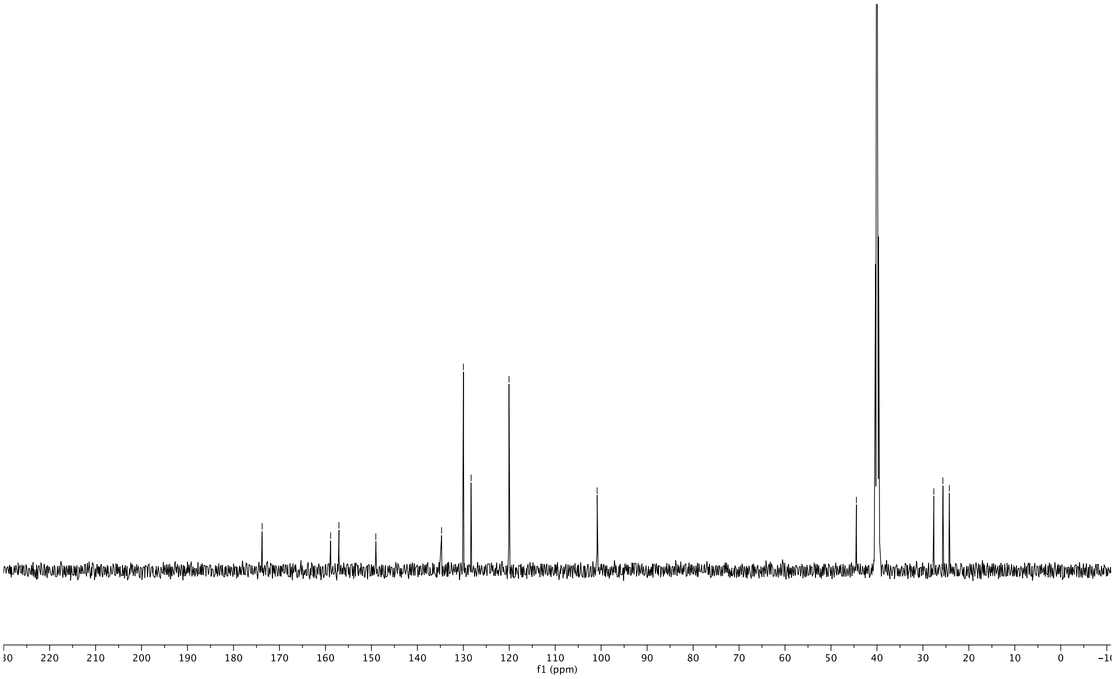

^{13}C{^{1}H} NMR (126 MHz, DMSO-d_6)

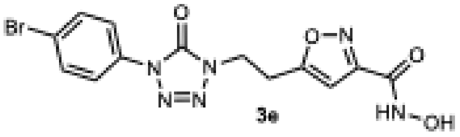

3e

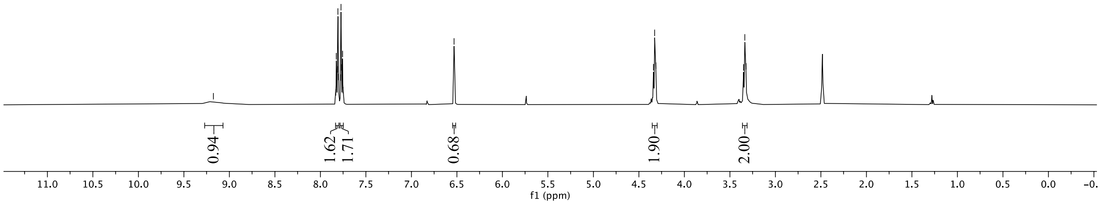

^{1}H NMR (500 MHz, DMSO-d_6)

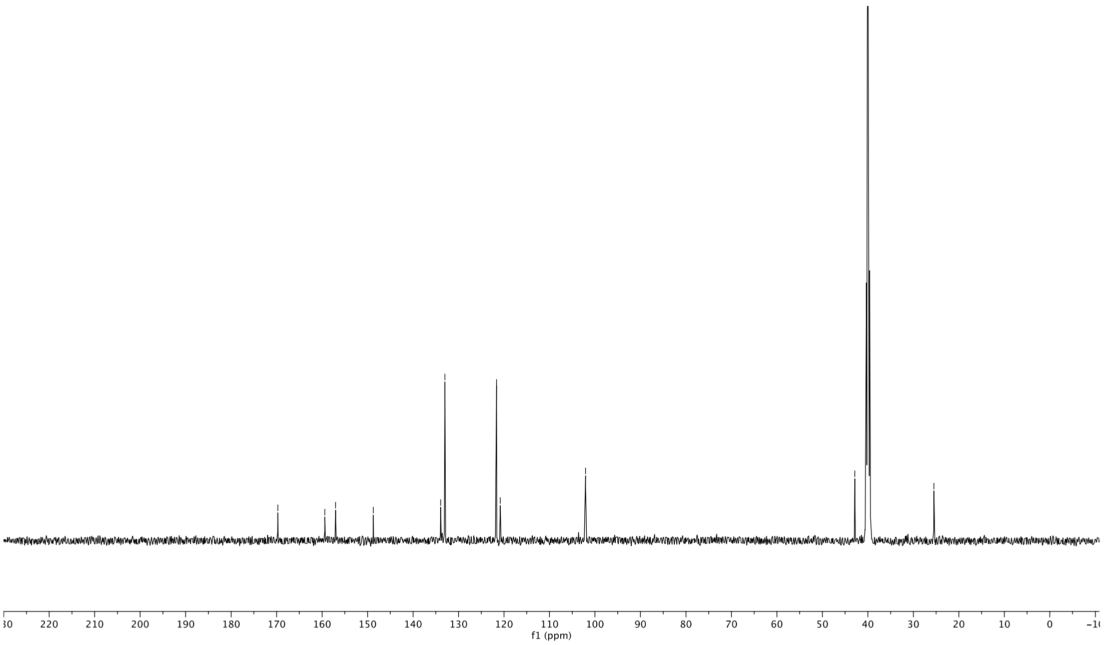

^{13}C{^{1}H} NMR (126 MHz, DMSO-d_6)

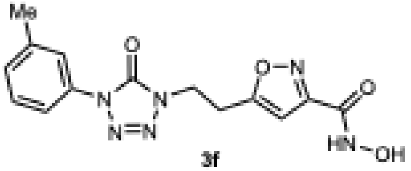

3f

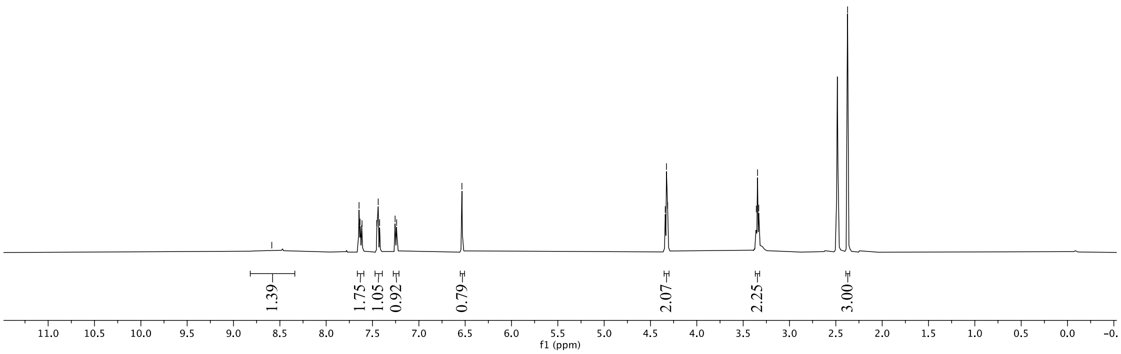

^{1}**H NMR** (500 MHz, DMSO-d_6)

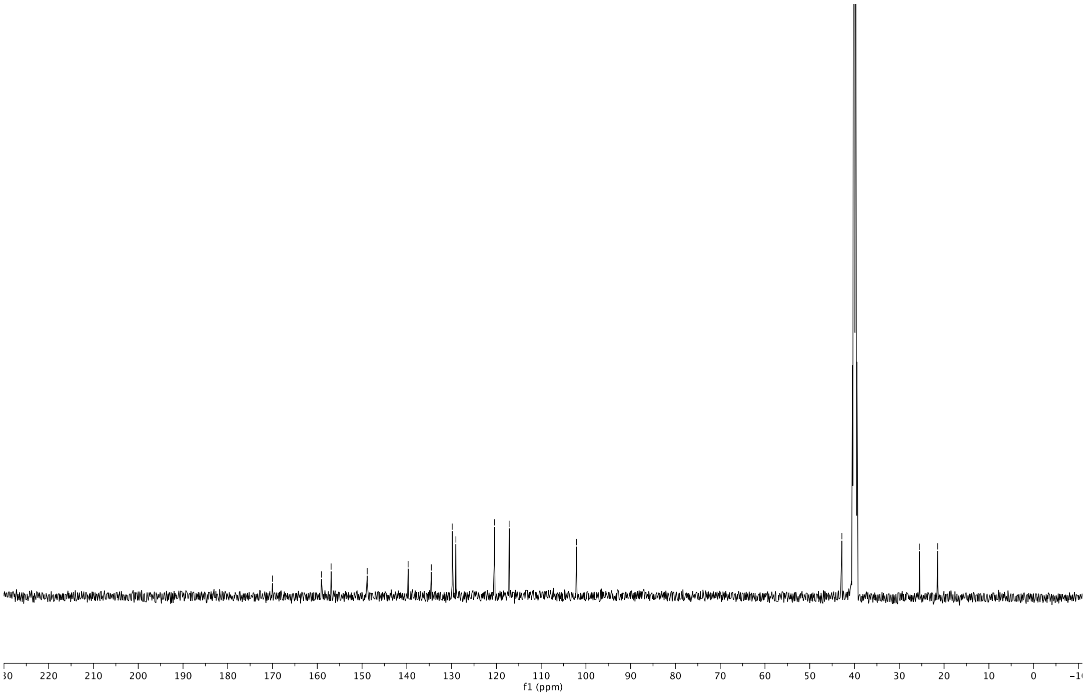

^{13}**C{**^{1}**H} NMR** (126 MHz, DMSO-d_6)

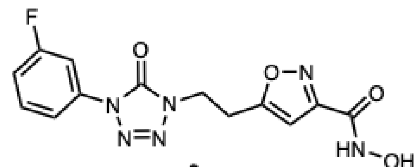

3g

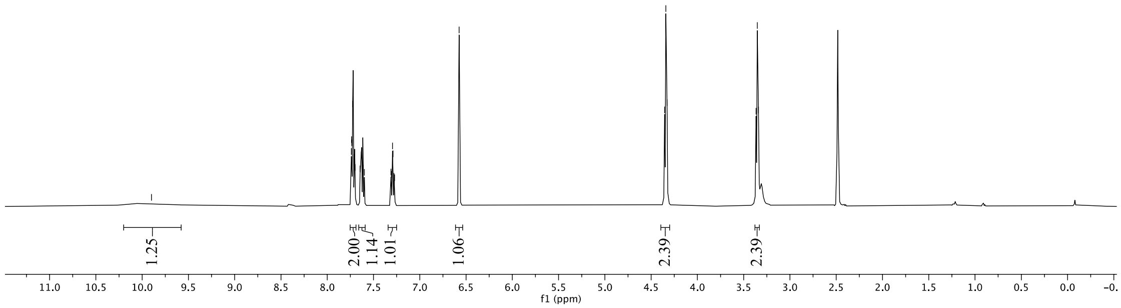

^{1}H NMR (500 MHz, DMSO-d_6)

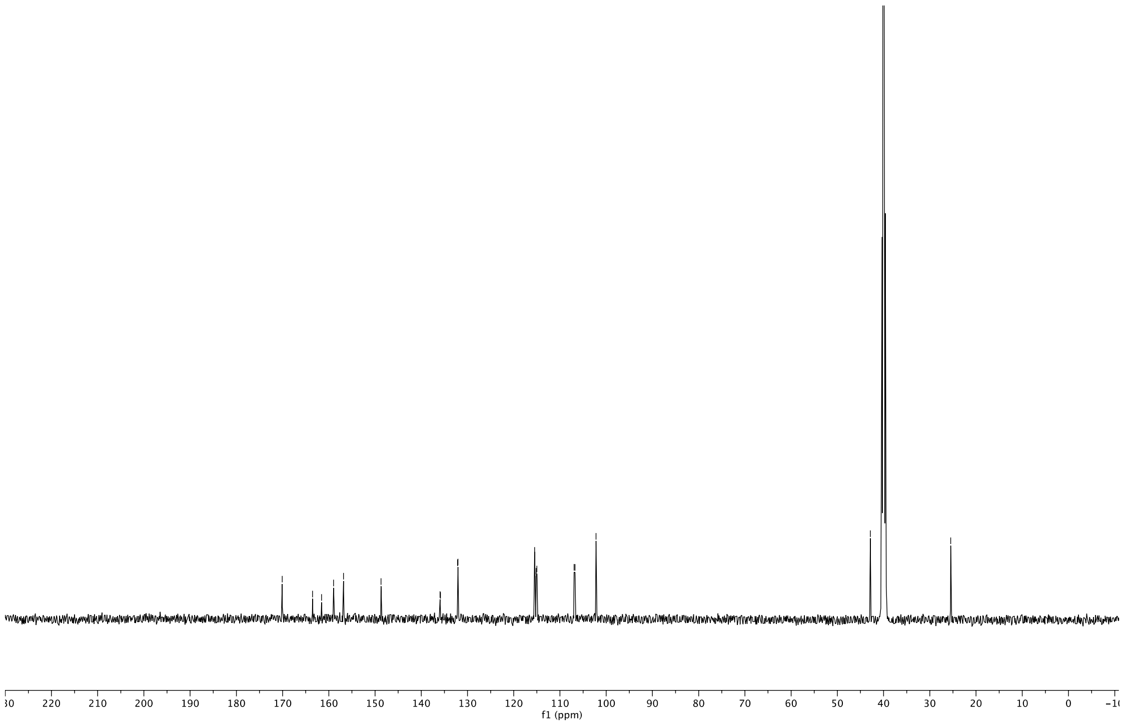

^{13}C{^{1}H} NMR (126 MHz, DMSO-d_6)

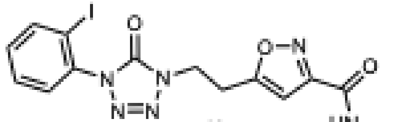

3h

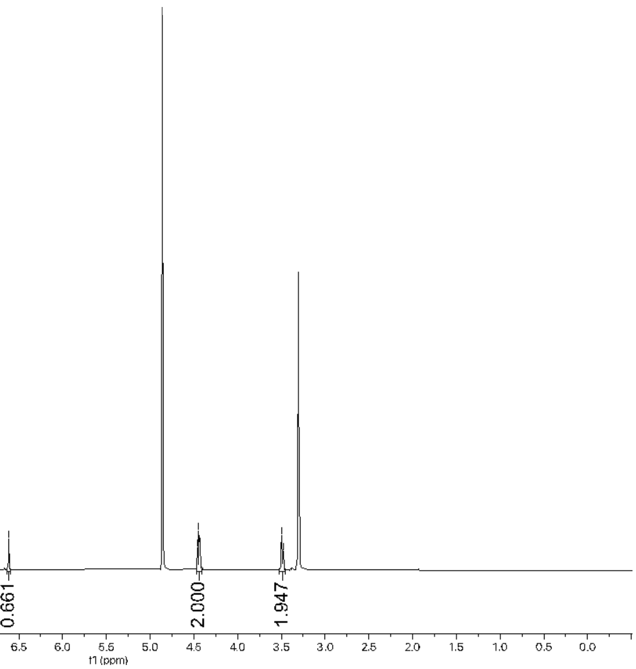

^{1}H NMR (500 MHz, DMSO-d_6)

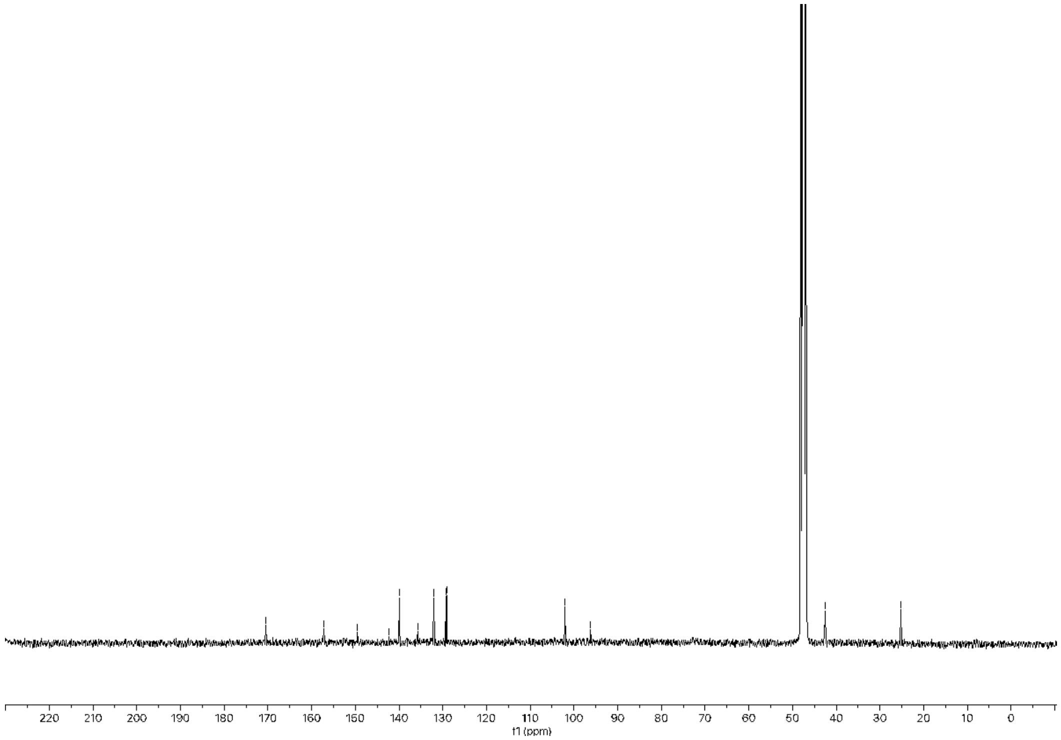

^{13}C{^{1}H} NMR (126 MHz, DMSO-d_6)

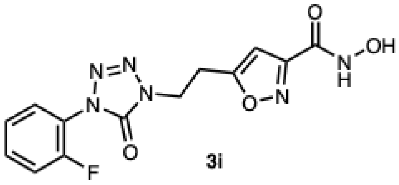

3i

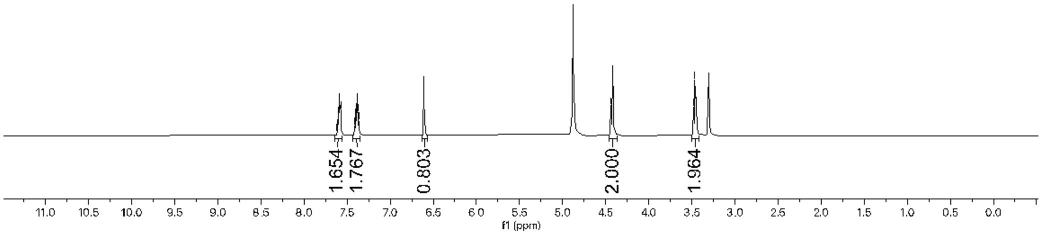

^{1}H NMR (500 MHz, DMSO-d_6)

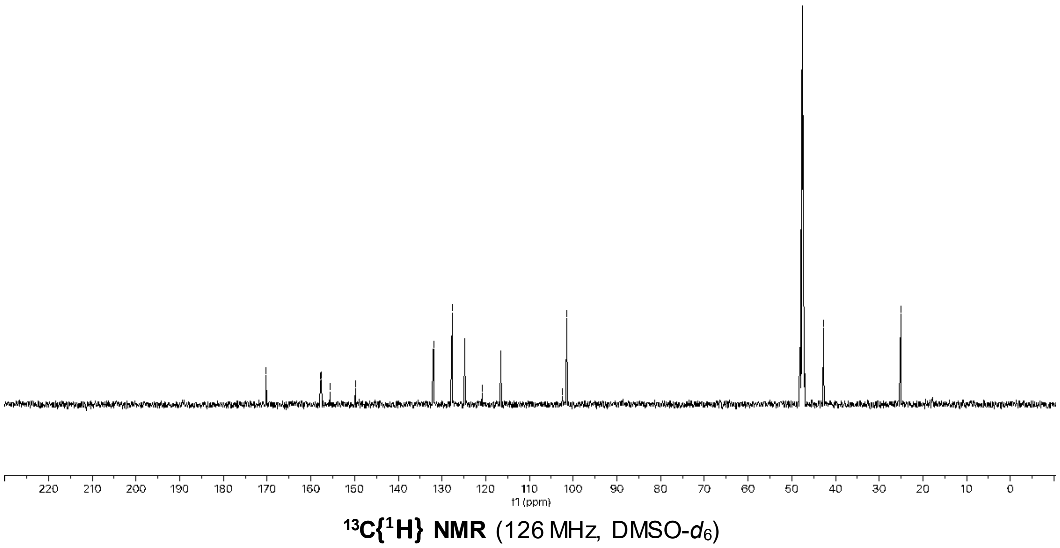

^{13}C{^{1}H} NMR (126 MHz, DMSO-d_6)

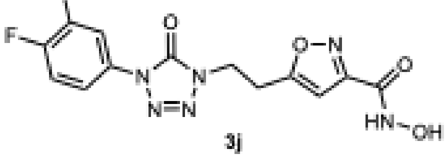

3j

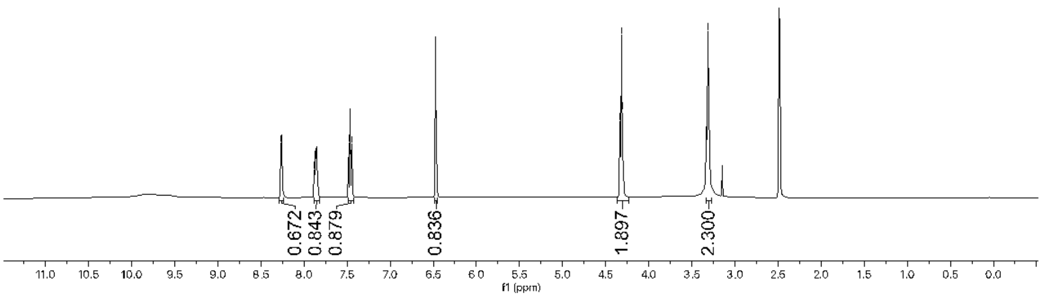

^{1}H NMR (500 MHz, DMSO-d_6)

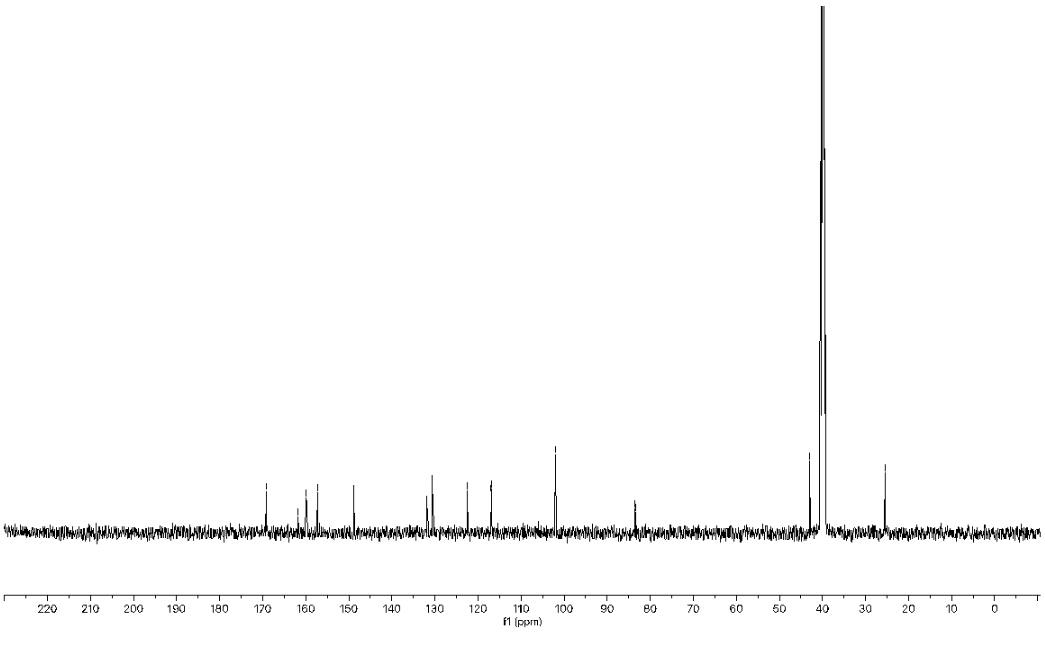

^{13}C{^{1}H} NMR (126 MHz, DMSO-d_6)

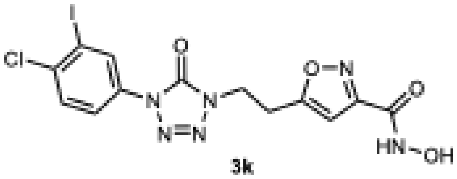

3k

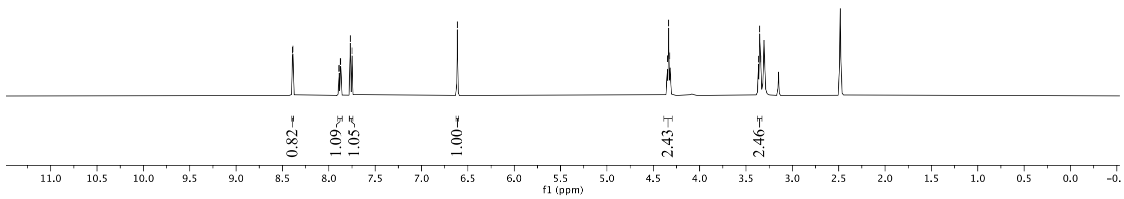

^{1}H NMR (500 MHz, DMSO-d_6)

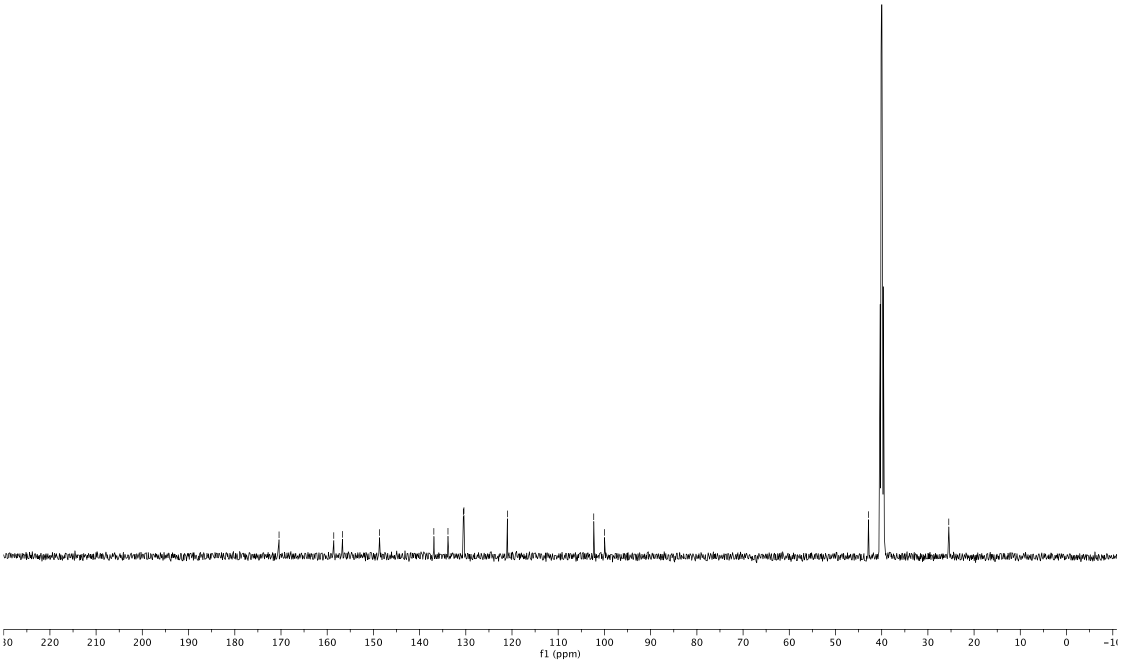

^{13}C{^{1}H} NMR (126 MHz, DMSO-d_6)

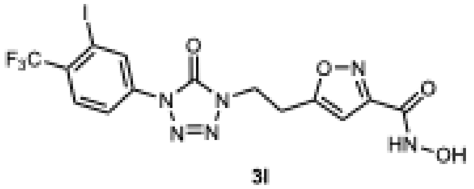

3l

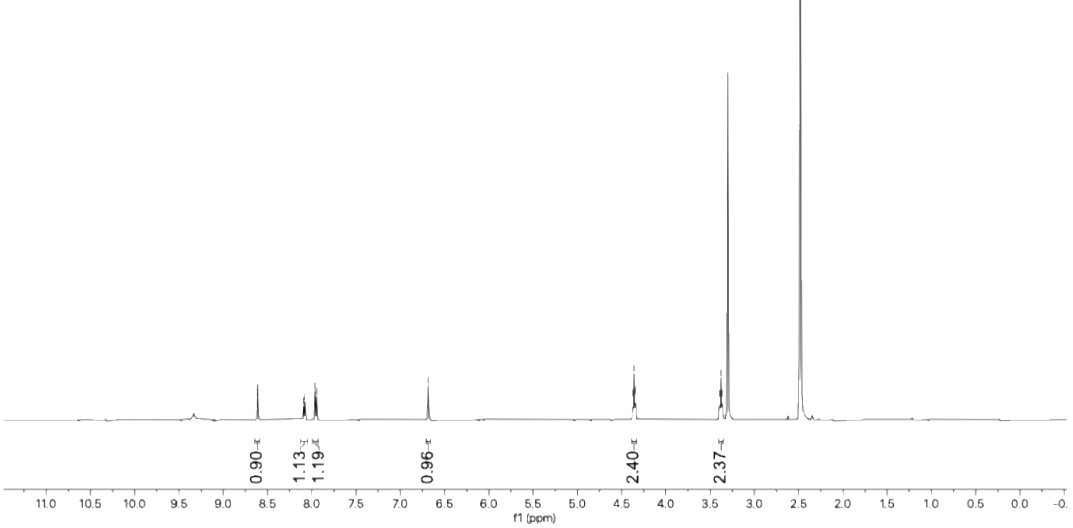

^{1}H NMR (500 MHz, DMSO-d_6)

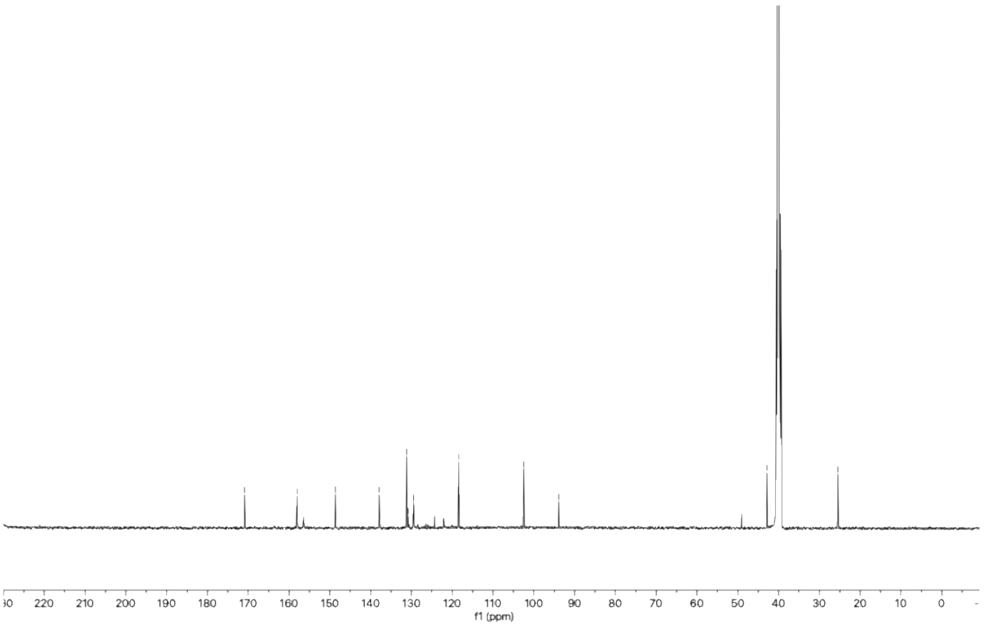

^{13}C{^{1}H} NMR (126 MHz, DMSO-d_6)

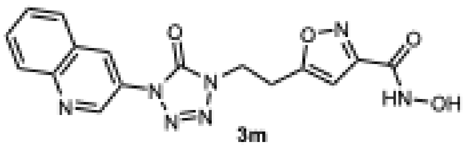

3m

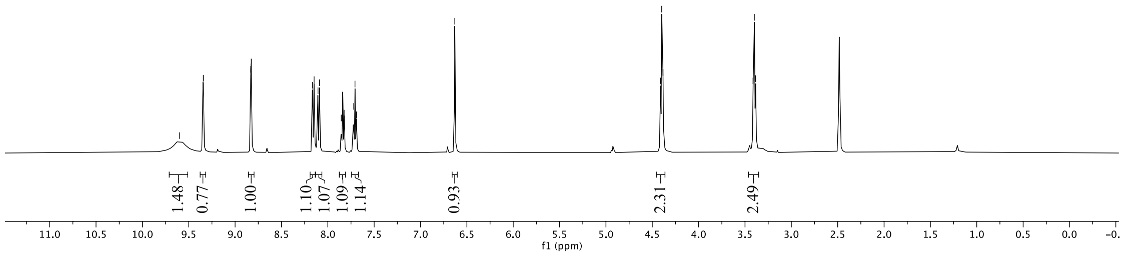

^{1}H NMR (500 MHz, DMSO-d_6)

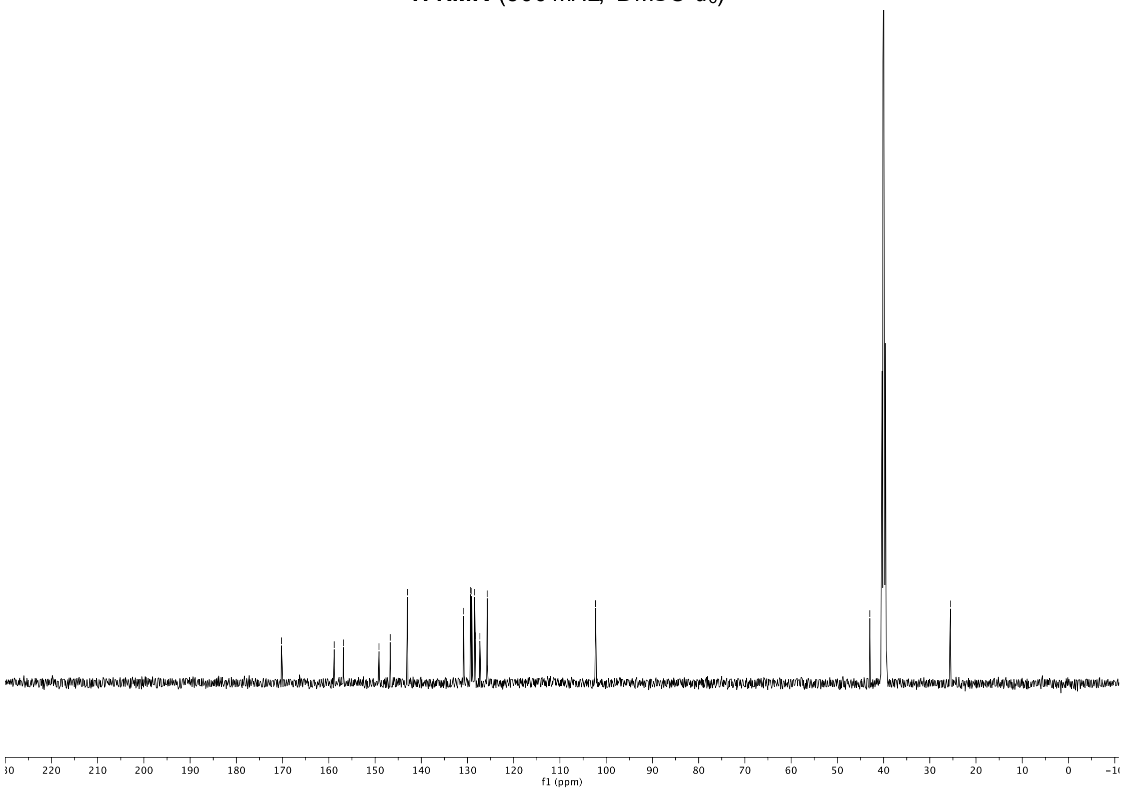

^{13}C{^{1}H} NMR (126 MHz, DMSO-d_6)

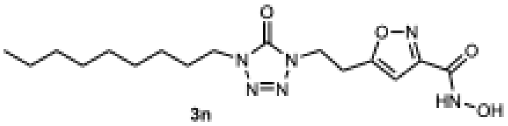

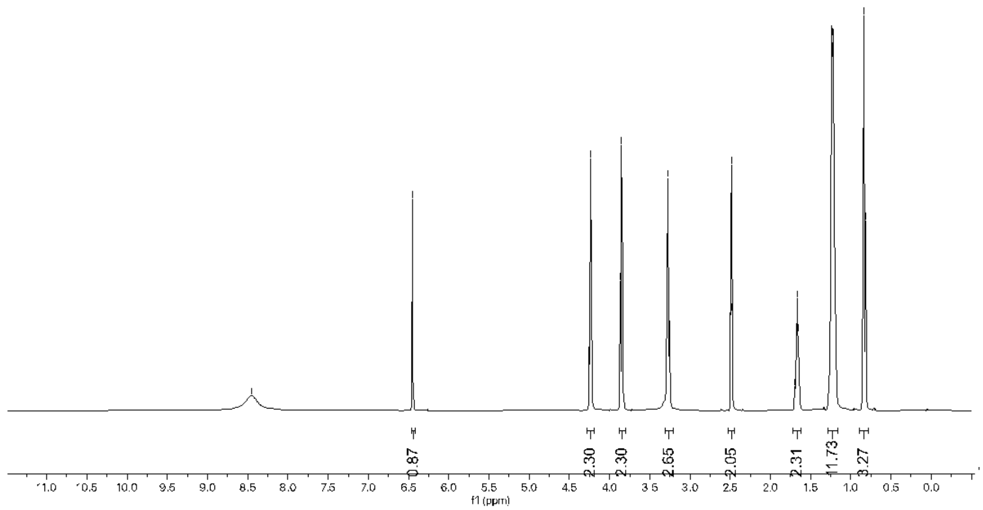

^{1}H NMR (500 MHz, DMSO-d_6)

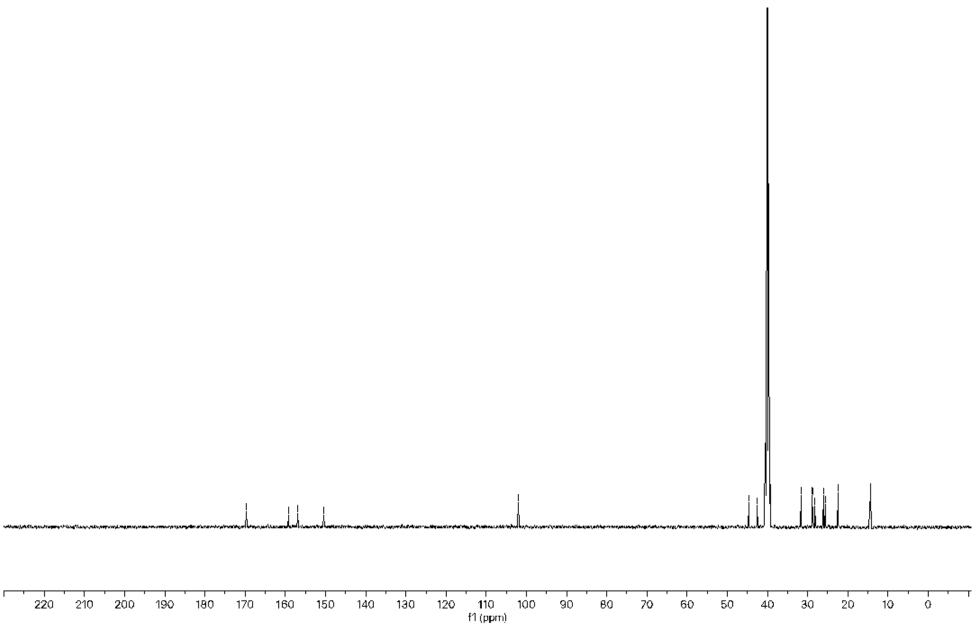

^{13}C{^{1}H} NMR (126 MHz, DMSO-d_6)

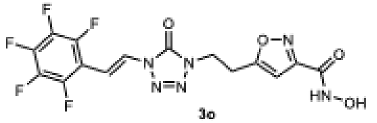

3o

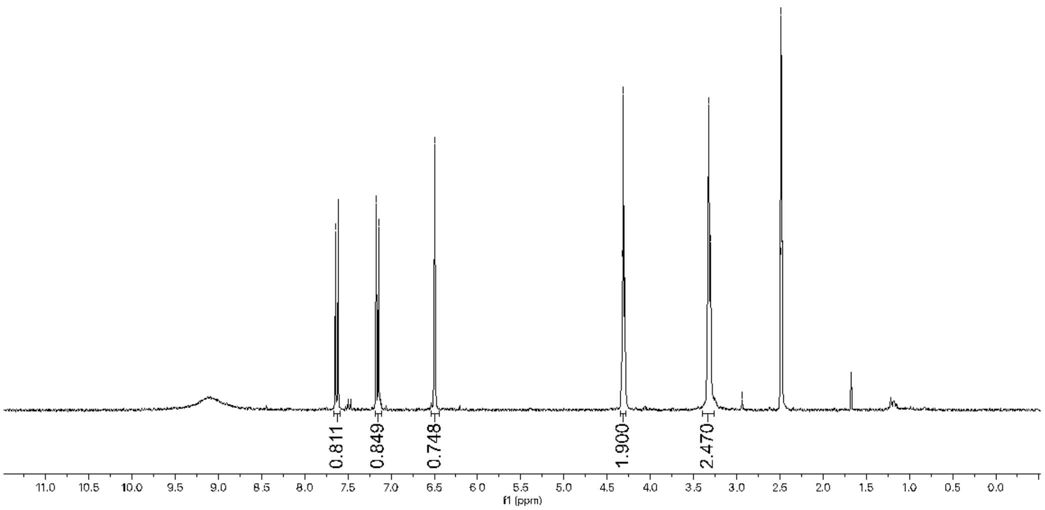

¹H NMR (500 MHz, DMSO-d6)

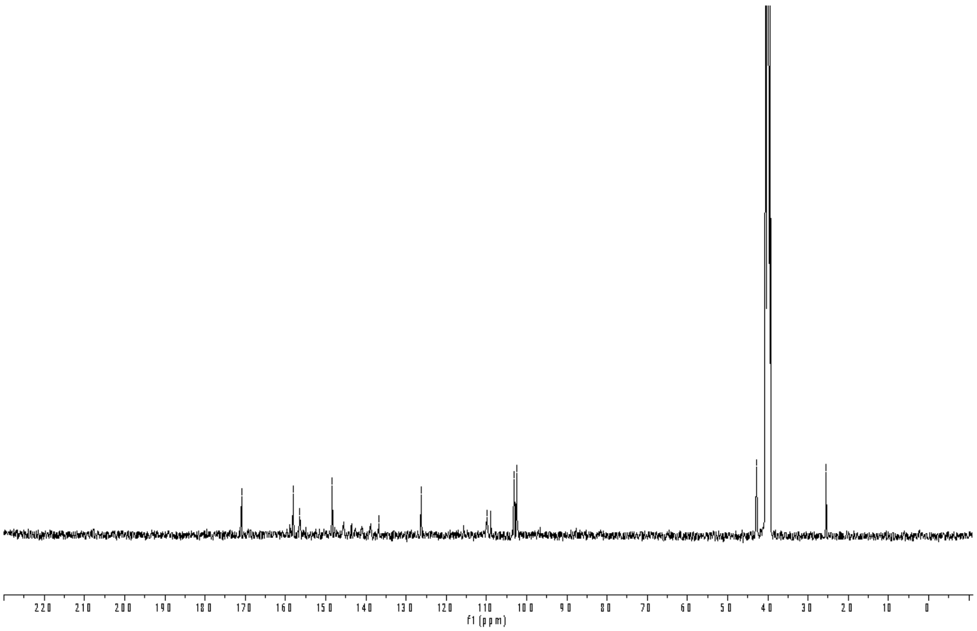

¹³C{¹H} NMR (126 MHz, DMSO-d6)

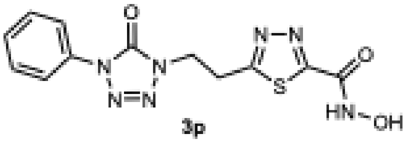

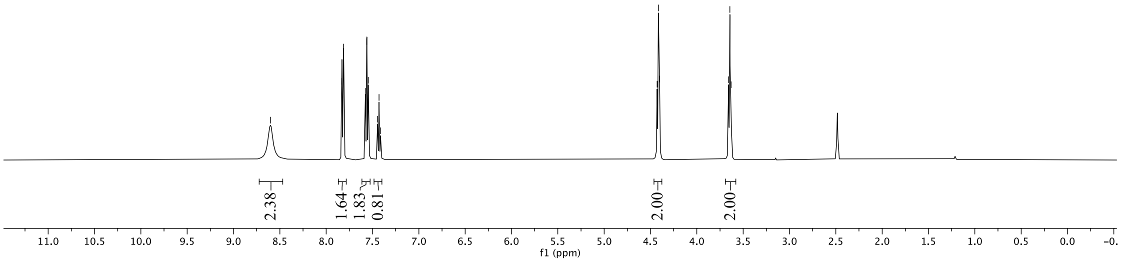

^{1}H NMR (500 MHz, DMSO-d_6)

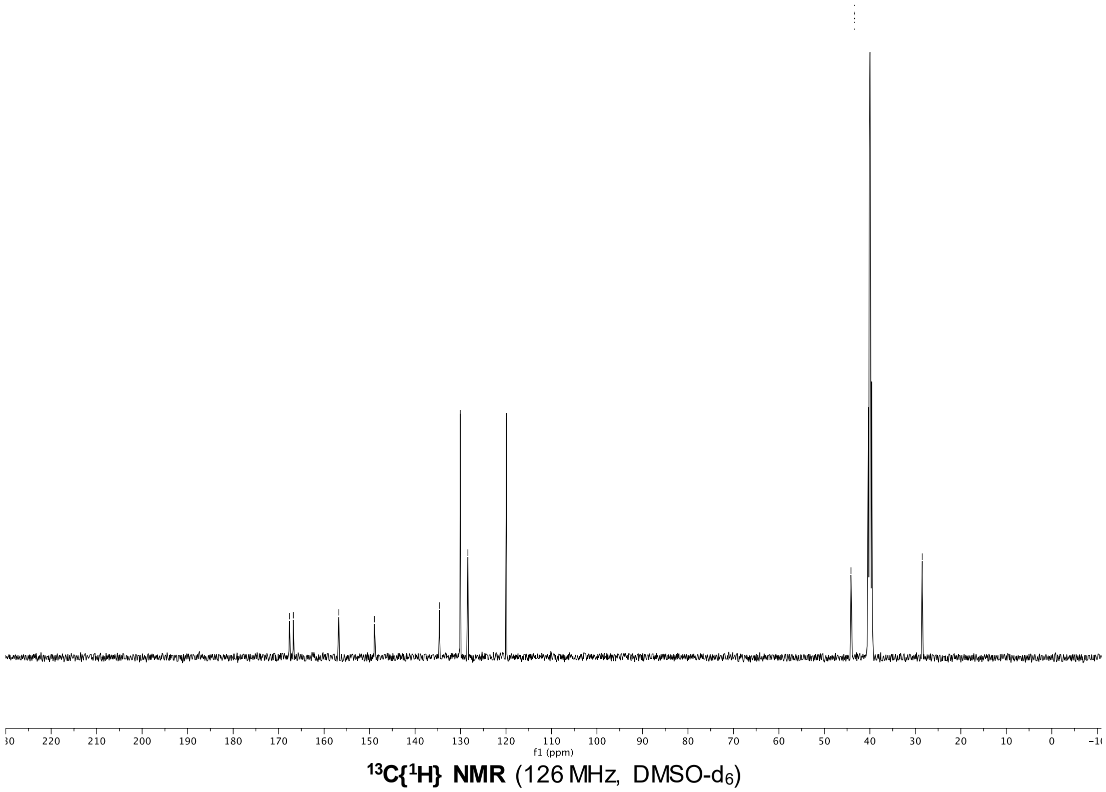

^{13}C{^{1}H} NMR (126 MHz, DMSO-d_6)

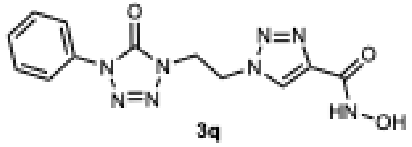

3q

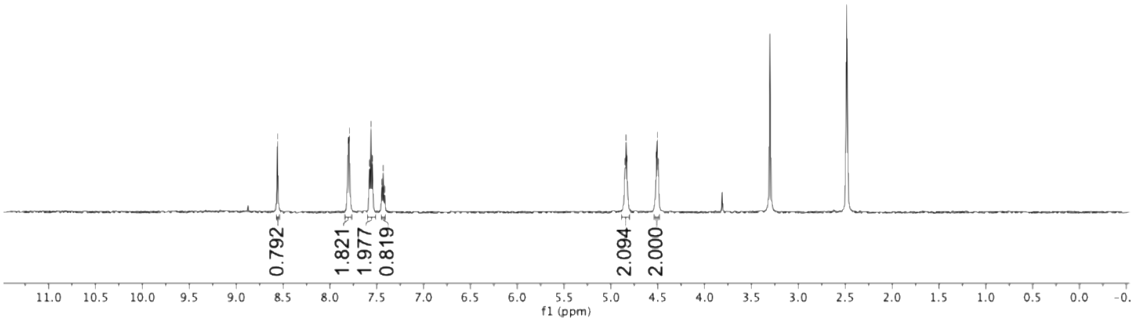

^{1}H NMR (500 MHz, DMSO-d_6)

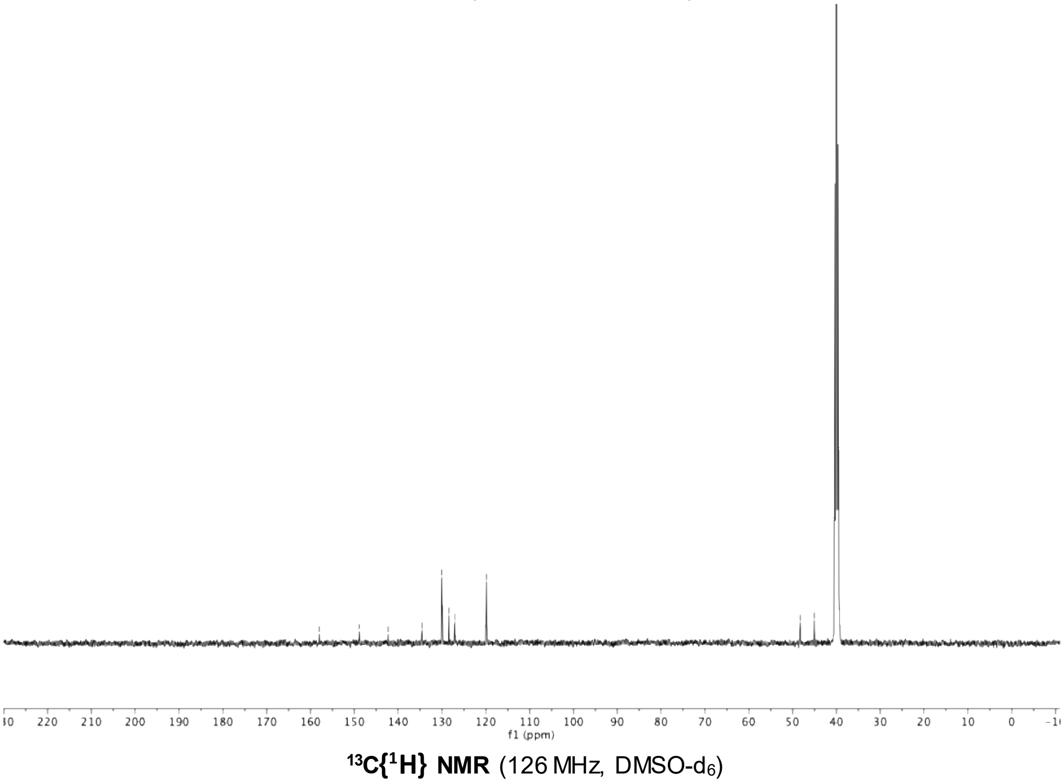

^{13}C{^{1}H} NMR (126 MHz, DMSO-d_6)

4.2 HPLC Chromatograms

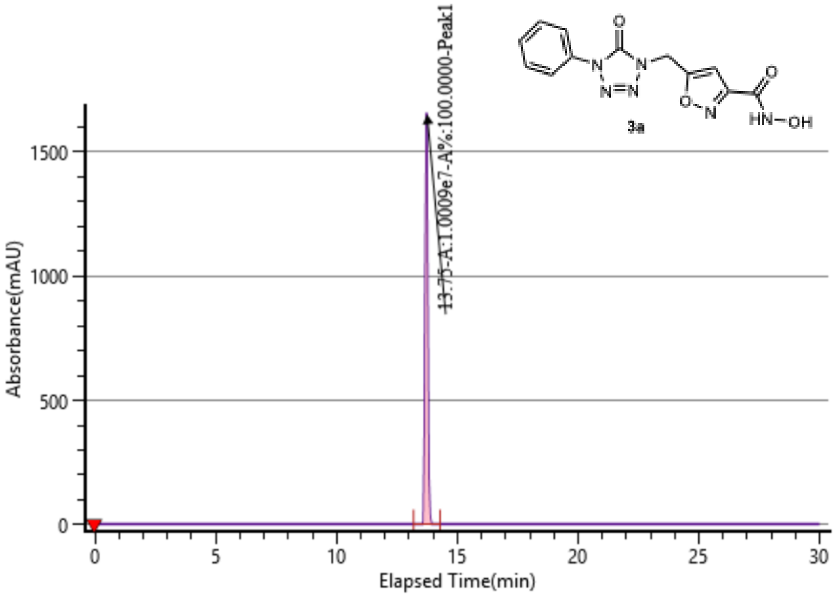

Peak ID	Retention Time (min)	Peak Area (mAU•s)	% Area	Peak Height (mAU)	Purity (%)
1 (**3a**)	13.75	1.00e7	100	1650	>99

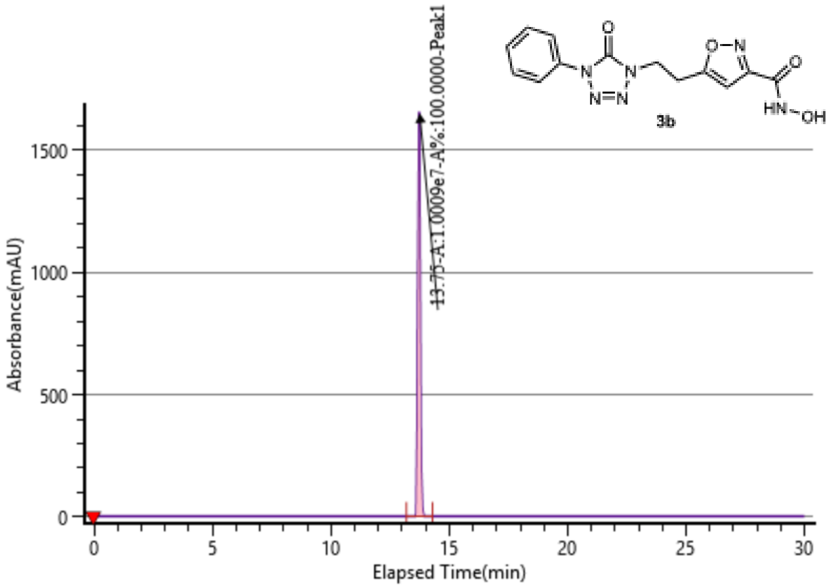

Peak ID	Retention Time (min)	Peak Area (mAU•s)	% Area	Peak Height (mAU)	Purity (%)
1 (**3b**)	13.75	5.83e7	100	3200	>99.0

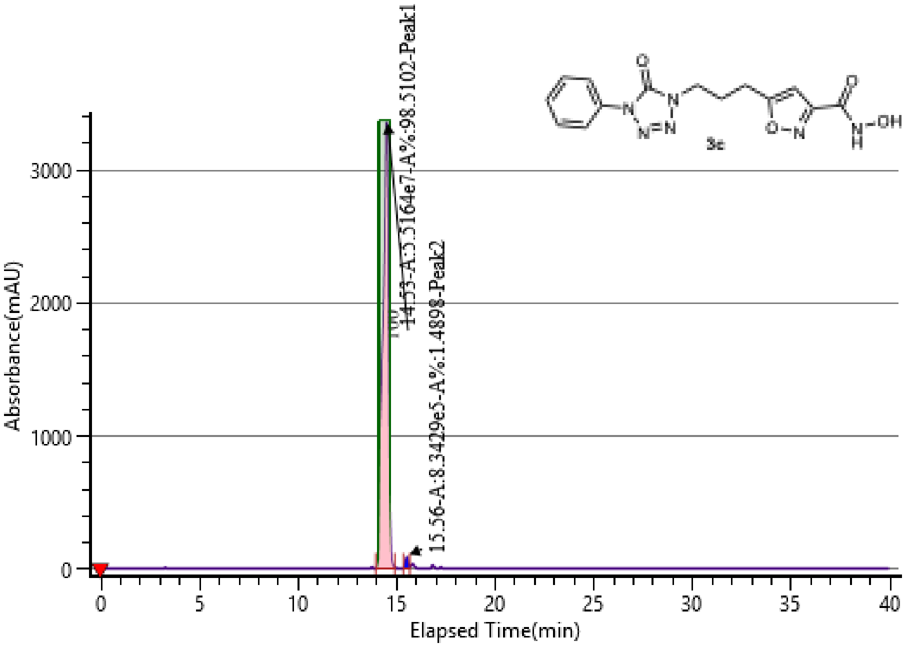

Peak ID	Retention Time (min)	Peak Area (mAU•s)	% Area	Peak Height (mAU)	Purity (%)
1 (**3c**)	14.53	5.51e7	98.5	3200	98.5
2	15.56	8.34e5	1.5	55	

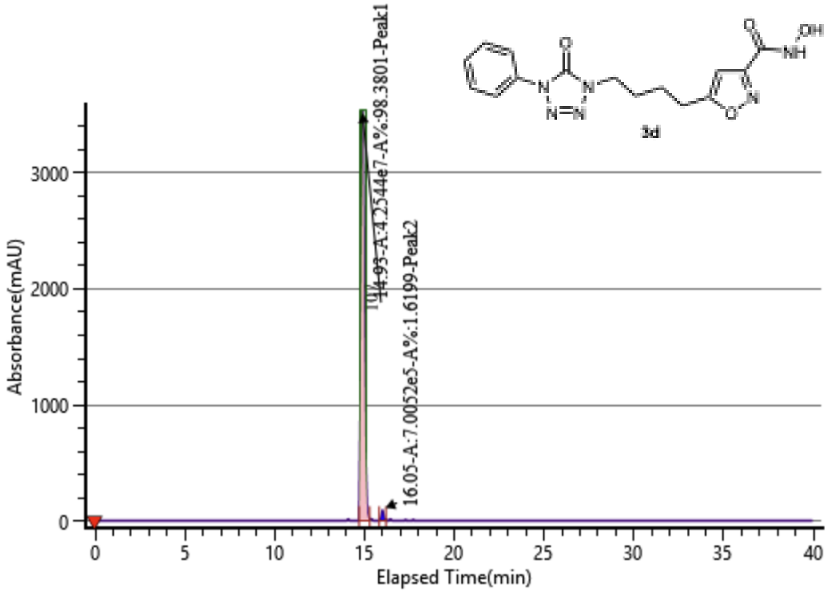

Peak ID	Retention Time (min)	Peak Area (mAU•s)	% Area	Peak Height (mAU)	Purity (%)
1 (**3d**)	14.93	4.25e7	98.4	3245	98.4
2	16.05	7.01e5	1.6	48	

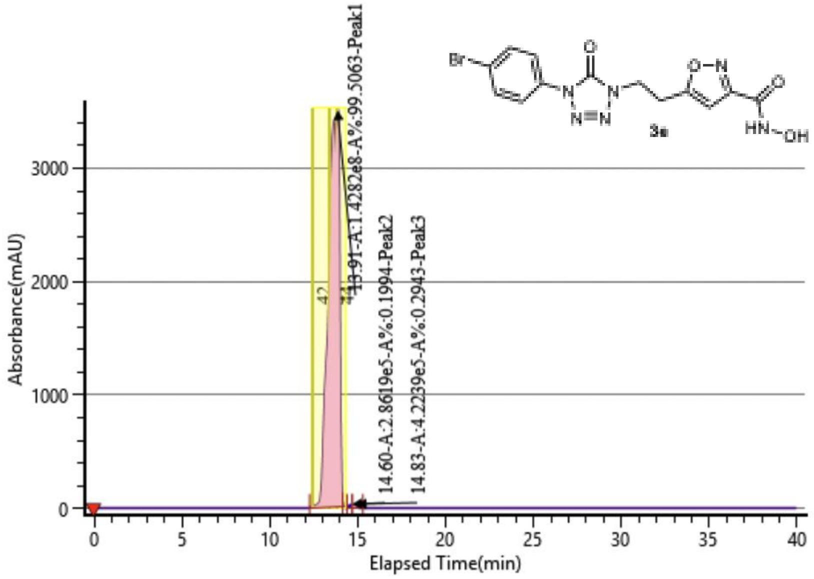

Peak ID	Retention Time (min)	Peak Area (mAU•s)	% Area	Peak Height (mAU)	Purity (%)
1 (**3e**)	13.91	1.43e8	99.5	3210	99.5
2	14.60	2.86e5	0.2	10	
3	14.83	4.22e5	0.3	7	

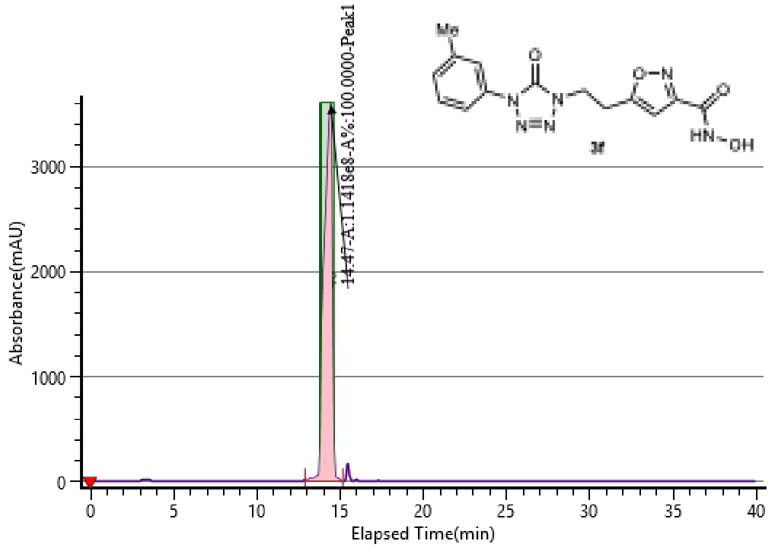

Peak ID	Retention Time (min)	Peak Area (mAU•s)	% Area	Peak Height (mAU)	Purity (%)
1 (**3f**)	14.47	1.14e8	100.0	3290	>99.0

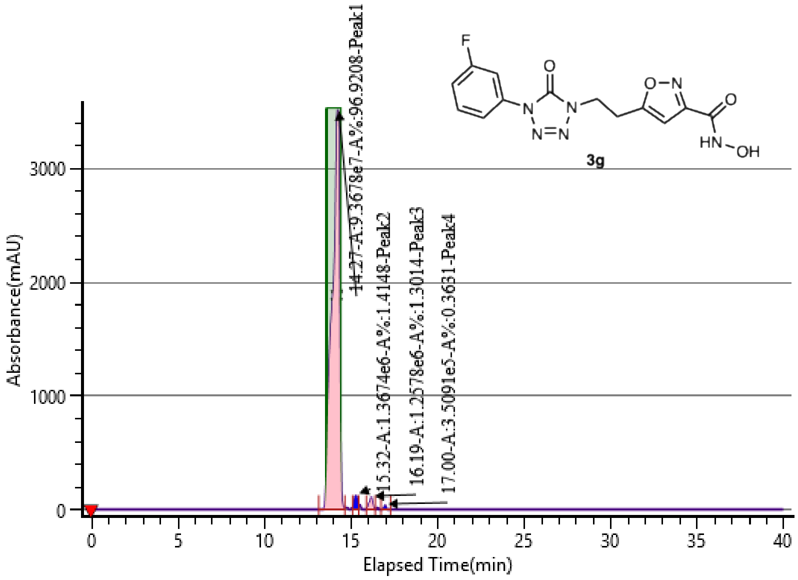

Peak ID	Retention Time (min)	Peak Area (mAU•s)	% Area	Peak Height (mAU)	Purity (%)
1 (**3g**)	14.27	9.37e7	96.9	3274	96.9
2	15.32	1.36e6	1.4	55	
3	16.19	1.25e6	1.3	60	
4	17.00	3.51e5	0.4	15	

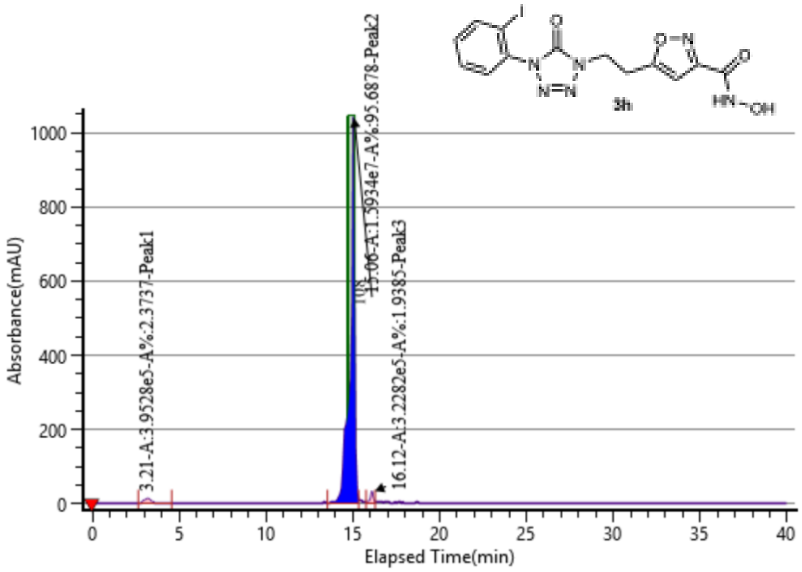

Peak ID	Retention Time (min)	Peak Area (mAU•s)	% Area	Peak Height (mAU)	Purity (%)
1	3.21	3.95e5	2.3	28	
2(**3h**)	15.06	1.59e7	95.6	1096	95.6
3	16.12	3.23e5	1.9	43	

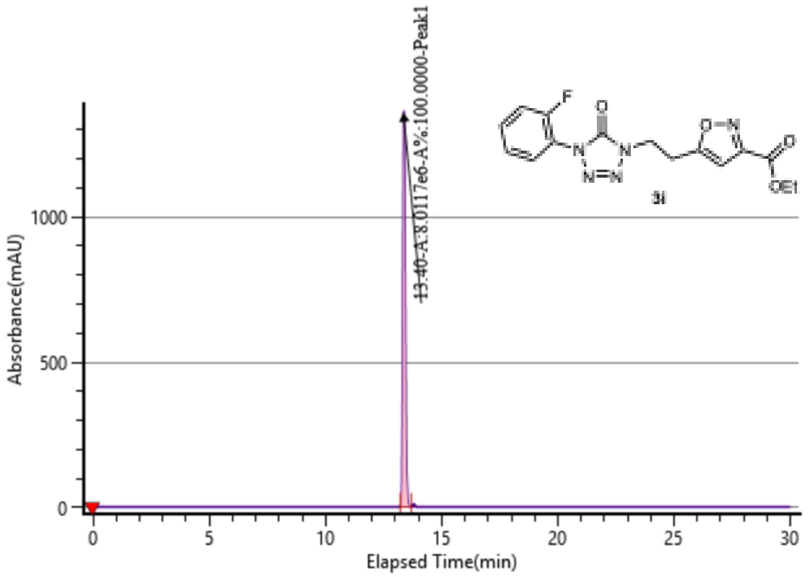

Peak ID	Retention Time (min)	Peak Area (mAU•s)	% Area	Peak Height (mAU)	Purity (%)
1 (**3i**)	13.40	8.01e6	100.0	1350	99.0

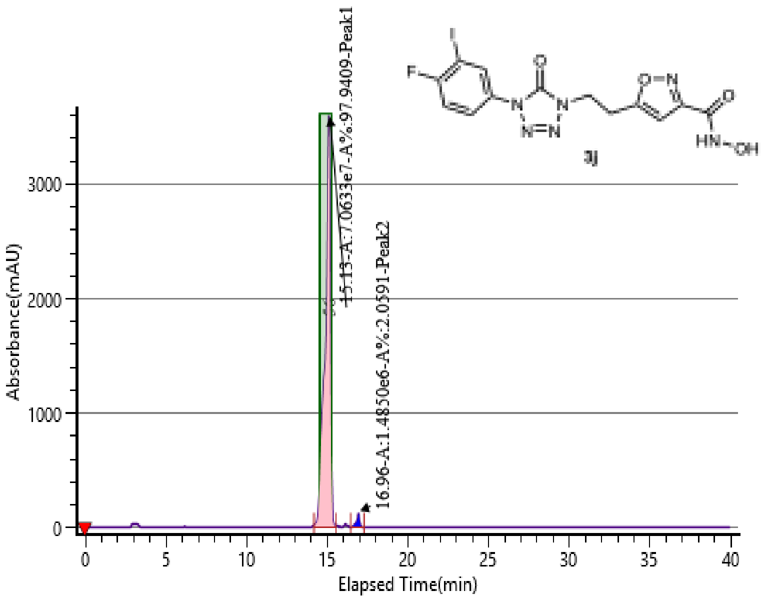

Peak ID	Retention Time (min)	Peak Area (mAU•s)	% Area	Peak Height (mAU)	Purity (%)
1 (**3j**)	15.13	7.06e7	97.9	3305	97.9
2	16.96	1.49e6	2.1	81	

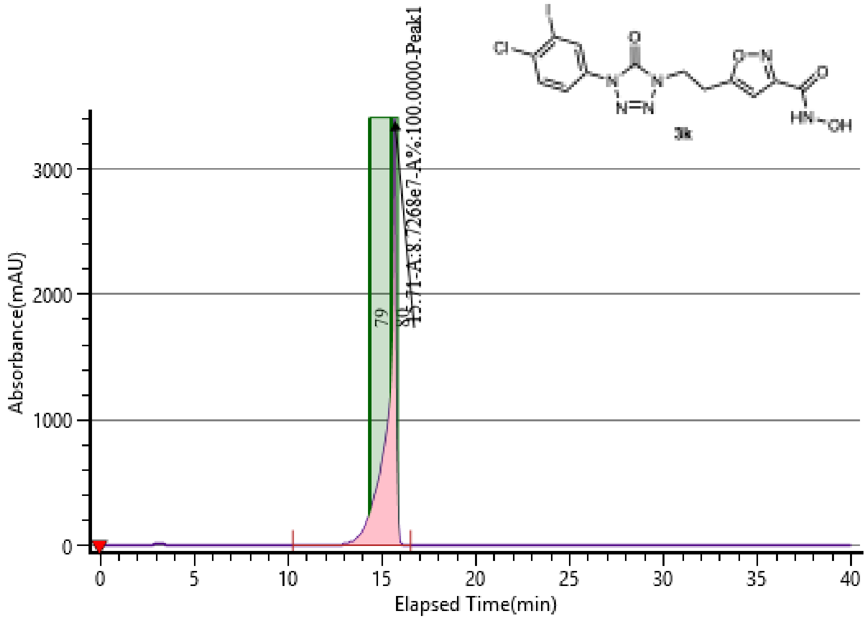

Peak ID	Retention Time (min)	Peak Area (mAU•s)	% Area	Peak Height (mAU)	Purity (%)
1 (**3k**)	15.71	8.72e7	100.0	3210	97.9

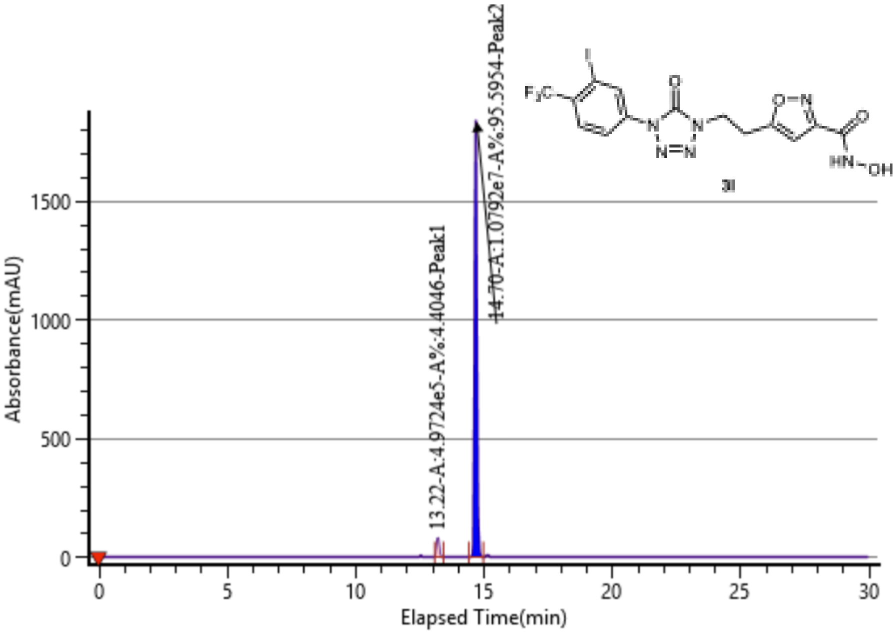

Peak ID	Retention Time (min)	Peak Area (mAU•s)	% Area	Peak Height (mAU)	Purity (%)
1	13.22	4.97e5	4.40	57	
2 (**3l**)	14.70	1.08e7	95.6	2846	95.6

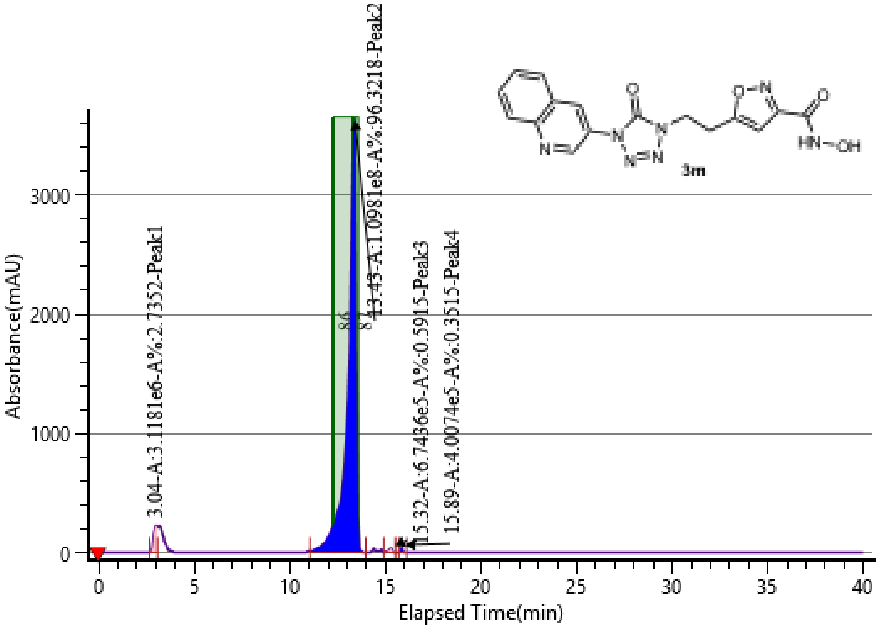

Peak ID	Retention Time (min)	Peak Area (mAU•s)	% Area	Peak Height (mAU)	Purity (%)
1	3.04	3.11e6	2.7	250	
2 (**3m**)	13.43	1.10e8	96.3	3310	96.3
3	15.32	6.74e5	0.6	25	
4	15.89	4.01e5	0.4	19	

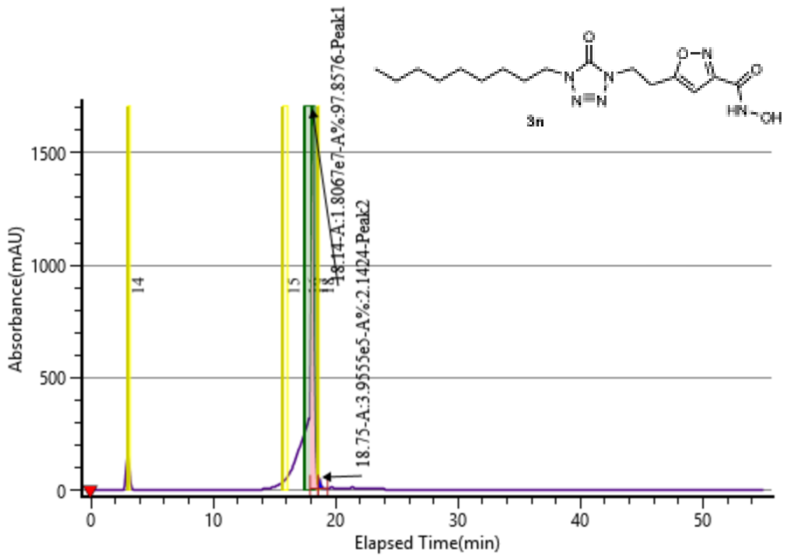

Peak ID	Retention Time (min)	Peak Area (mAU•s)	% Area	Peak Height (mAU)	Purity (%)
1 (**3n**)	18.14	1.81e6	97.9	2021	97.9
2	18.75	3.96e4	2.1	40.5	

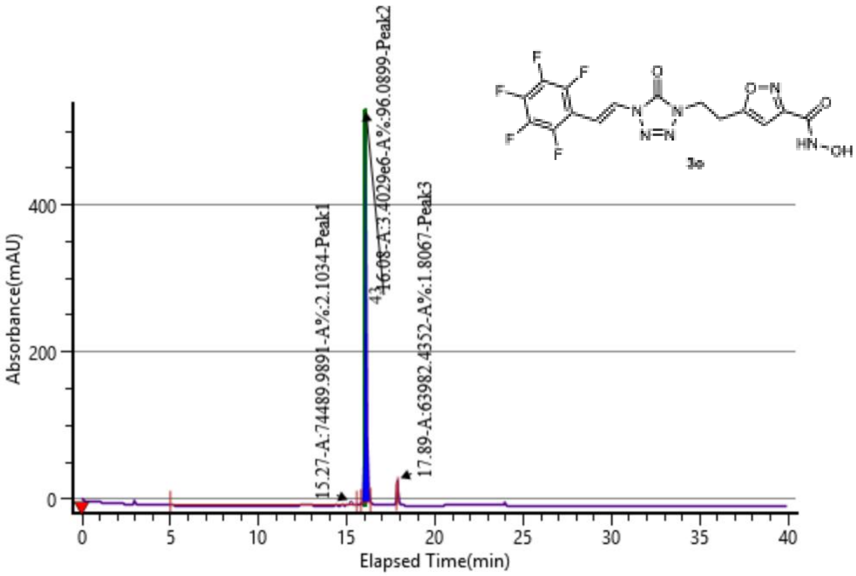

Peak ID	Retention Time (min)	Peak Area (mAU•s)	% Area	Peak Height (mAU)	Purity (%)
1	15.27	7.4e4	2.1	5	
2 (**3o**)	16.08	3.40e6	96.1	3296	96.1
3	17.89	6.3e4	1.8	45	

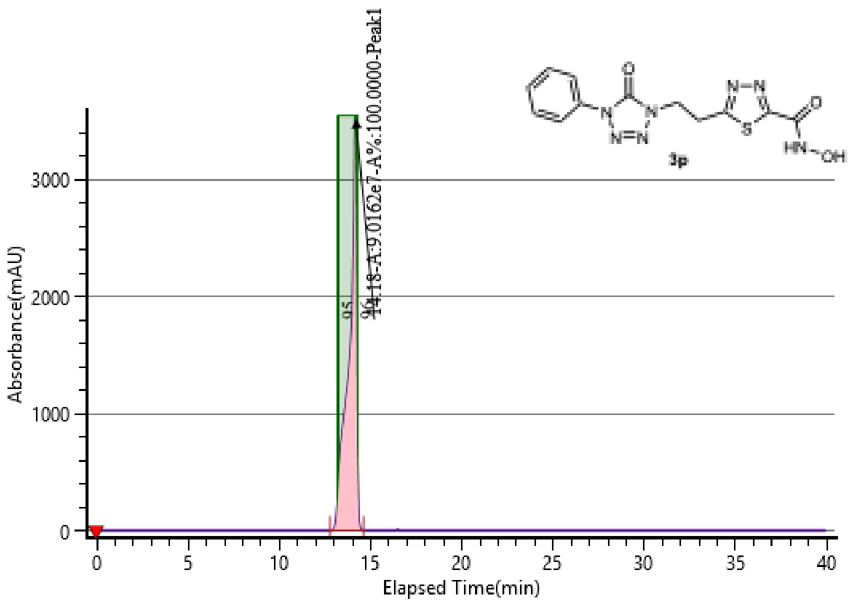

Peak ID	Retention Time (min)	Peak Area (mAU•s)	% Area	Peak Height (mAU)	Purity (%)
1 (**3p**)	14.18	9.02e7	100	3010	>99

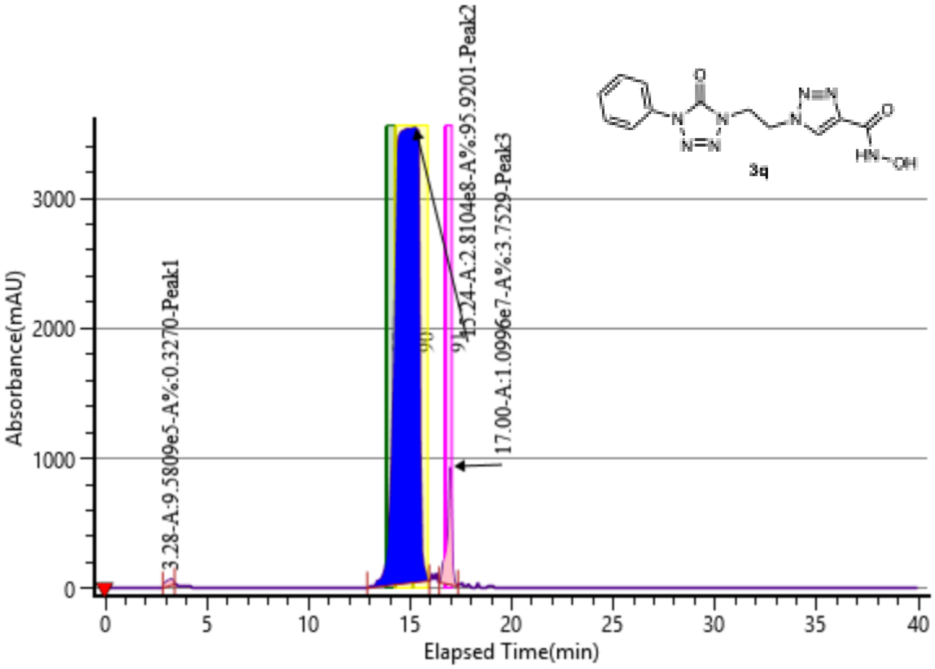

Peak ID	Retention Time (min)	Peak Area (mAU•s)	% Area	Peak Height (mAU)	Purity (%)
1	3.28	9.58e5	0.3	40	
2 (**3q**)	15.24	2.81e8	95.9	3280	95.9
3	17.00	1.1e7	3.8	950	

5. HDAC6 Screening and macrophage functional assay supporting data

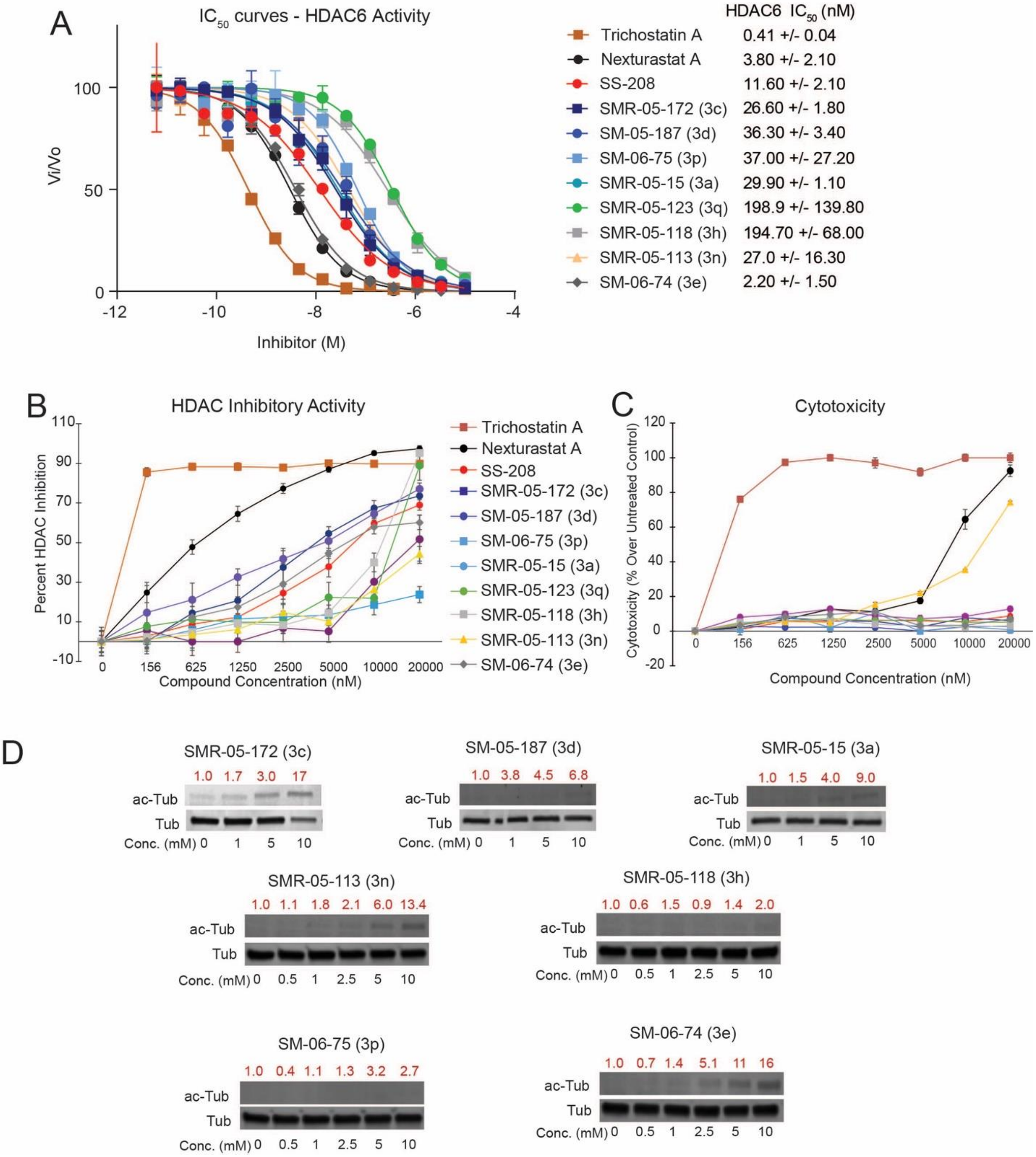

Figure S3. Screening of non-lead tetrazolones for HDAC6 inhibition. (**A**) Full-dose response inhibition curves for compounds with inhibitory potency below 50% under screening conditions. Corresponding IC_{50} values were calculated from the inhibition curves using the GraphPad Prism software (GraphPad Software, San Diego, CA, USA). Data are plotted as mean values ± SD from three independent experiments (n = 3), (**B**) Determination of deacetylase activity using HDAC-Glo (Promega) analysis in RAW264.7 macrophages at indicated concentrations of HDACis. (**C**)

Cytotoxicity assay of HDACis at indicated concentrations using Cytotox (Promega). (**D**) Immunoblot analysis was performed in RAW264.7 macrophages that were treated with different concentrations of candidate HDAC6 inhibitors to assess tubulin and acetylated tubulin levels. The band intensities were quantified in Image Studio.

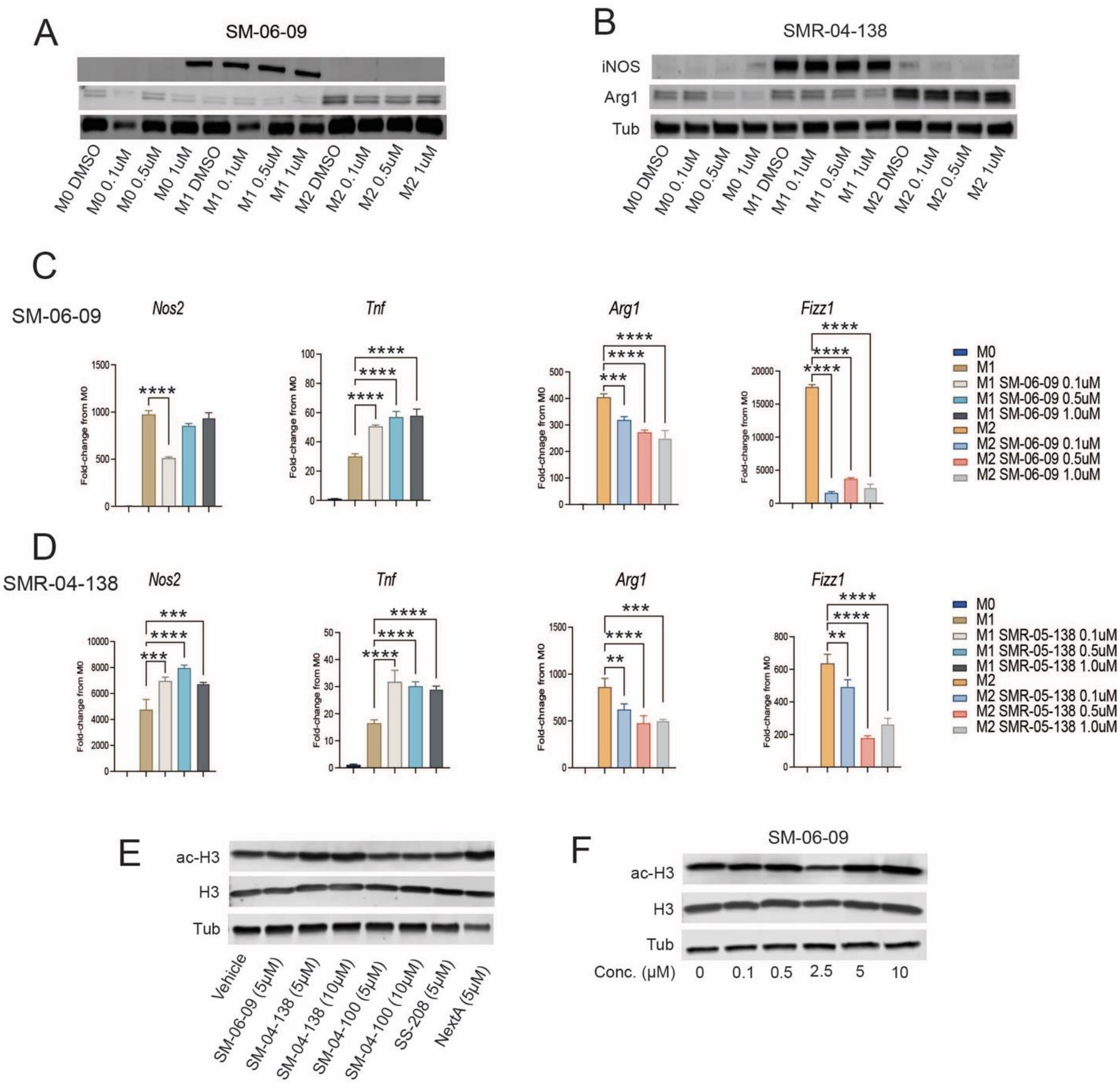

Figure S4. **HDAC6 inhibition improved the pro-inflammatory M1 phenotype in bone marrow-derived macrophages at nanomolar concentrations.** Bone marrow-derived macrophages were treated with lower concentrations (0.1, 0.5, and 1 μM) of SM-06-09 (**3m**) and **3b** and polarized to M1 with 100 ng/mL of LPS and 50 ng/mL of IFN-γ, and M2 (20 ng/mL IL-4 and IL-13). The classical M1 marker, iNos, and M2 marker, Arginase, were detected by Western blot (**A**) SM-06-09 (**3m**), (**B**) **3b**. (**C and D**) qRT-PCR was performed to measure the changes in expression of markers associated with M1 (*Nos2, Tnf*) and M2 (*Arg1 and Fizz1*) phenotype of macrophages. The levels of acetyl histone 3 and total histone 3 levels were quantified in RAW264.7 cells treated with (**E**) 3b, 3m, and 3l, along with SS-208 and NextA and (**F**) different concentrations of SM-06-09. Data plotted are mean values ±SD from technical replicates (n=3); **P<0.01, ***P<0.001, ****P<0.0001, one-way ANOVA.

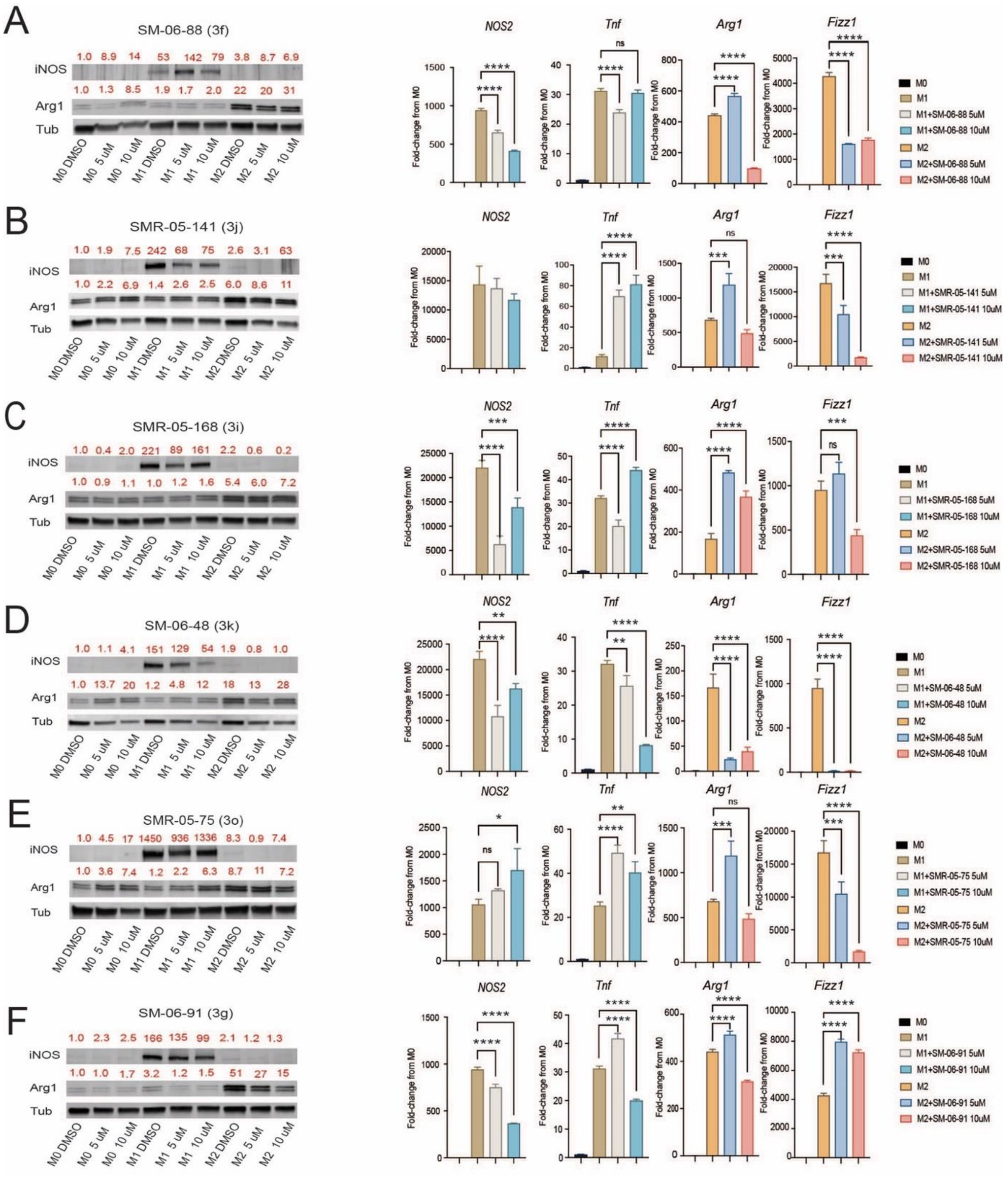

Figure S5. Selected HDAC6 inhibitors with modest effects on the macrophage phenotype. Bone marrow-derived macrophages were treated with selected HDAC6 inhibitors and polarized to M1-like (100ng/mL of LPS and 50ng/mL of IFN-g) and M2-like (20 ng/mL of IL-4 and IL-13 each) phenotypes. Immunoblot and qRT-PCR analyses for (**A**) **3f**, (**B**) **3j**, (**C**) **3k**, (**D**) SM-06-48 (**3k**), (**E**) **3o**, (**F**) **3g** showing changes in levels of iNOS and Arg1 (in immunoblot) and genes associated with M1 (*Nos2, Tnf*) and M2 (*Arg1, Fizz1*) phenotype of macrophages (in qRT-PCR).

Data plotted are mean values $\pm$SD from technical replicates (n = 3): *P<0.05,**P<0.01, ***P<0.001, ****P<0.0001, one-way ANOVA.

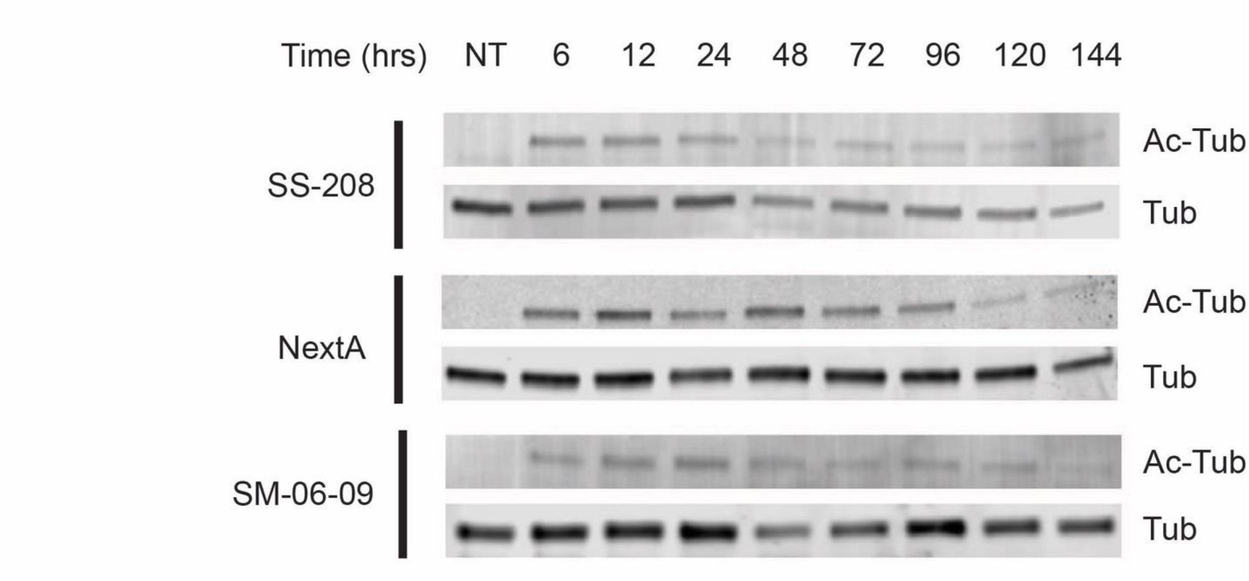

Figure S6. Selected tetrazolones show sustained HDAC6 inhibition after a single treatment dose in BMDMs. Bone marrow-derived macrophages were treated with a single dose of SS-208 (**1**), Next A, and SM-06-09 (**3m**). Cells were harvested post-treatment at different time points (6, 12, 24, 48, 72, 96, 120, and 144 hours) to quantify acetyl alpha-tubulin by Western blot.

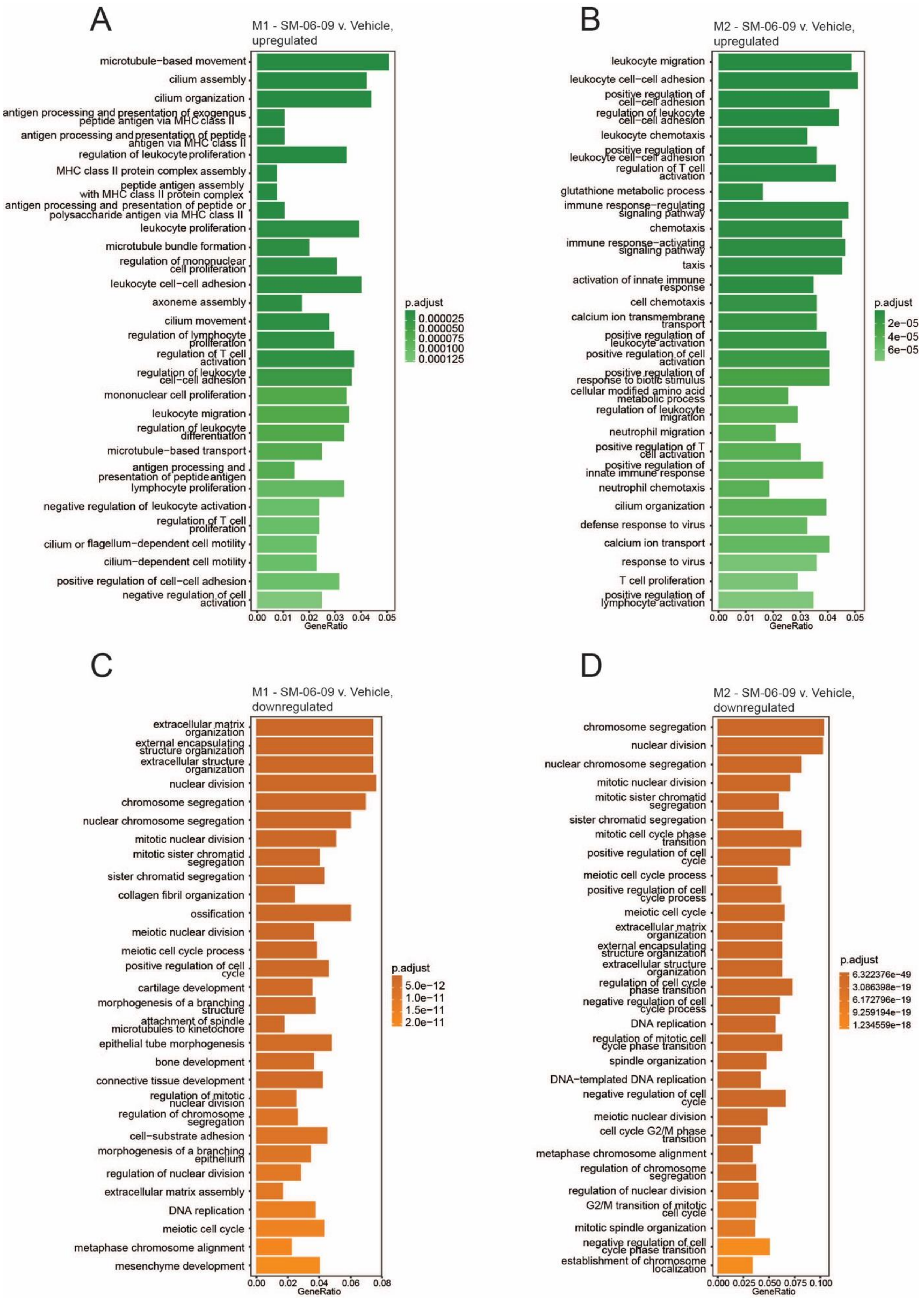
A
M1 - SM-06-09 v. Vehicle, upregulated
microtubule−based movement
cilium assembly
cilium organization
antigen processing and presentation of exogenous peptide antigen via MHC class II
antigen processing and presentation of peptide antigen via MHC class II
regulation of leukocyte proliferation
MHC class II protein complex assembly
peptide antigen assembly with MHC class II protein complex
antigen processing and presentation of peptide or polysaccharide antigen via MHC class II
leukocyte proliferation
microtubule bundle formation
regulation of mononuclear cell proliferation
leukocyte cell−cell adhesion
axoneme assembly
cilium movement
regulation of lymphocyte proliferation
regulation of T cell activation
regulation of leukocyte cell−cell adhesion
mononuclear cell proliferation
leukocyte migration
regulation of leukocyte differentiation
microtubule−based transport
antigen processing and presentation of peptide antigen
lymphocyte proliferation
negative regulation of leukocyte activation
regulation of T cell proliferation
cilium or flagellum−dependent cell motility
cilium−dependent cell motility
positive regulation of cell−cell adhesion
negative regulation of cell activation
GeneRatio
p.adjust
0.000025
0.000050
0.000075
0.000100
0.000125
B
M2 - SM-06-09 v. Vehicle, upregulated
leukocyte migration
leukocyte cell−cell adhesion
positive regulation of cell−cell adhesion
regulation of leukocyte cell−cell adhesion
leukocyte chemotaxis
positive regulation of leukocyte cell−cell adhesion
regulation of T cell activation
glutathione metabolic process
immune response−regulating signaling pathway
chemotaxis
immune response−activating signaling pathway
taxis
activation of innate immune response
cell chemotaxis
calcium ion transmembrane transport
positive regulation of leukocyte activation
positive regulation of cell activation
positive regulation of response to biotic stimulus
cellular modified amino acid metabolic process
regulation of leukocyte migration
neutrophil migration
positive regulation of T cell activation
positive regulation of innate immune response
neutrophil chemotaxis
cilium organization
defense response to virus
calcium ion transport
response to virus
T cell proliferation
positive regulation of lymphocyte activation
GeneRatio
p.adjust
2e−05
4e−05
6e−05
C
M1 - SM-06-09 v. Vehicle, downregulated
extracellular matrix organization
external encapsulating structure organization
extracellular structure organization
nuclear division
chromosome segregation
nuclear chromosome segregation
mitotic nuclear division
mitotic sister chromatid segregation
sister chromatid segregation
collagen fibril organization
ossification
meiotic nuclear division
meiotic cell cycle process
positive regulation of cell cycle
cartilage development
morphogenesis of a branching structure
attachment of spindle microtubules to kinetochore
epithelial tube morphogenesis
bone development
connective tissue development
regulation of mitotic nuclear division
regulation of chromosome segregation
cell−substrate adhesion
morphogenesis of a branching epithelium
regulation of nuclear division
extracellular matrix assembly
DNA replication
meiotic cell cycle
metaphase chromosome alignment
mesenchyme development
GeneRatio
p.adjust
5.0e−12
1.0e−11
1.5e−11
2.0e−11
D
M2 - SM-06-09 v. Vehicle, downregulated
chromosome segregation
nuclear division
nuclear chromosome segregation
mitotic nuclear division
mitotic sister chromatid segregation
sister chromatid segregation
mitotic cell cycle phase transition
positive regulation of cell cycle
meiotic cell cycle process
positive regulation of cell cycle process
meiotic cell cycle
extracellular matrix organization
external encapsulating structure organization
extracellular structure organization
regulation of cell cycle phase transition
negative regulation of cell cycle process
DNA replication
regulation of mitotic cell cycle phase transition
spindle organization
DNA−templated DNA replication
negative regulation of cell cycle
meiotic nuclear division
cell cycle G2/M phase transition
metaphase chromosome alignment
regulation of chromosome segregation
regulation of nuclear division
G2/M transition of mitotic cell cycle
mitotic spindle organization
negative regulation of cell cycle phase transition
establishment of chromosome localization
GeneRatio
p.adjust
6.322376e−49
3.086398e−19
6.172796e−19
9.259194e−19
1.234559e−18

Figure S7. Functional enrichment analysis of differentially expressed genes between SM-06-09-treated and untreated BMDMs reveals enhanced inflammatory and tumor immune responses in both M1- and M2-polarized macrophages. Top 30 GO-BP terms for upregulated (**A**, **B**) genes and downregulated (**C**, **D**) genes after SM-06-09 (**3m**) treatment in M1- (**A**, **C**) and M2- (**B**, **D**) polarized BMDMs.

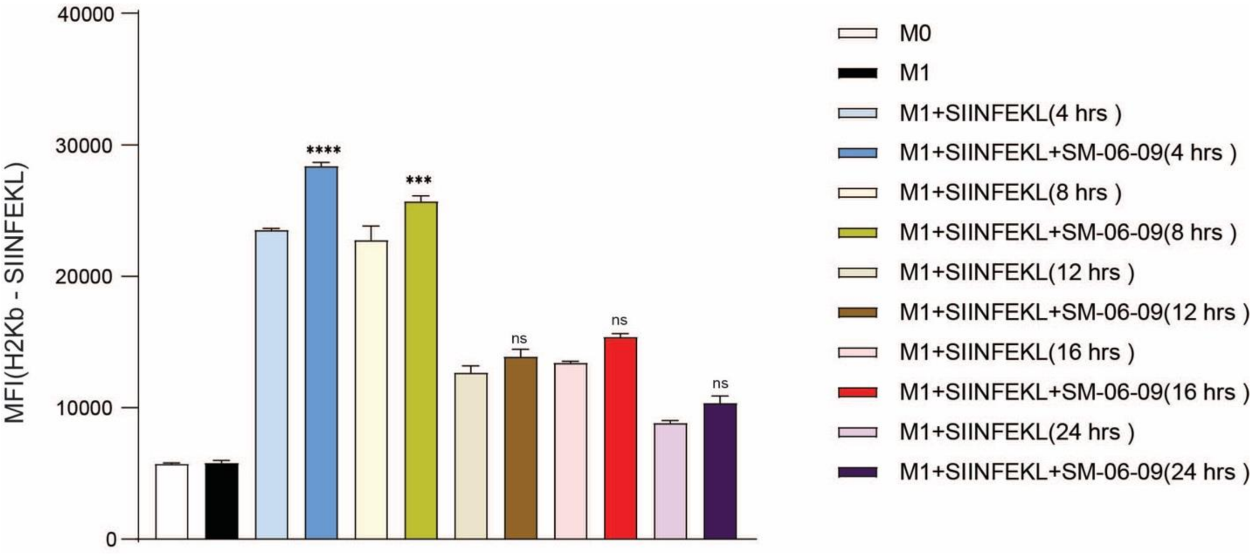

Figure S8. Time course of antigen presentation in M1 macrophages treated with SM-06-09. M1 polarized macrophages were incubated with OVA peptide (40ng/µL) for 4, 8, 16, and 24 hours, while M0 and M1 macrophages served as controls. The expression of the H2Kb-SIINFEKL complex in response to SM-06-09 (**3m**) treatment at the specified time points was evaluated by flow cytometry to assess antigen presentation. Data plotted are mean values +SD from technical replicates (n=3): *P<0.05,**P<0.01, ***P<0.001, ****P<0.0001, one-way ANOVA

6. In vivo studies supporting data

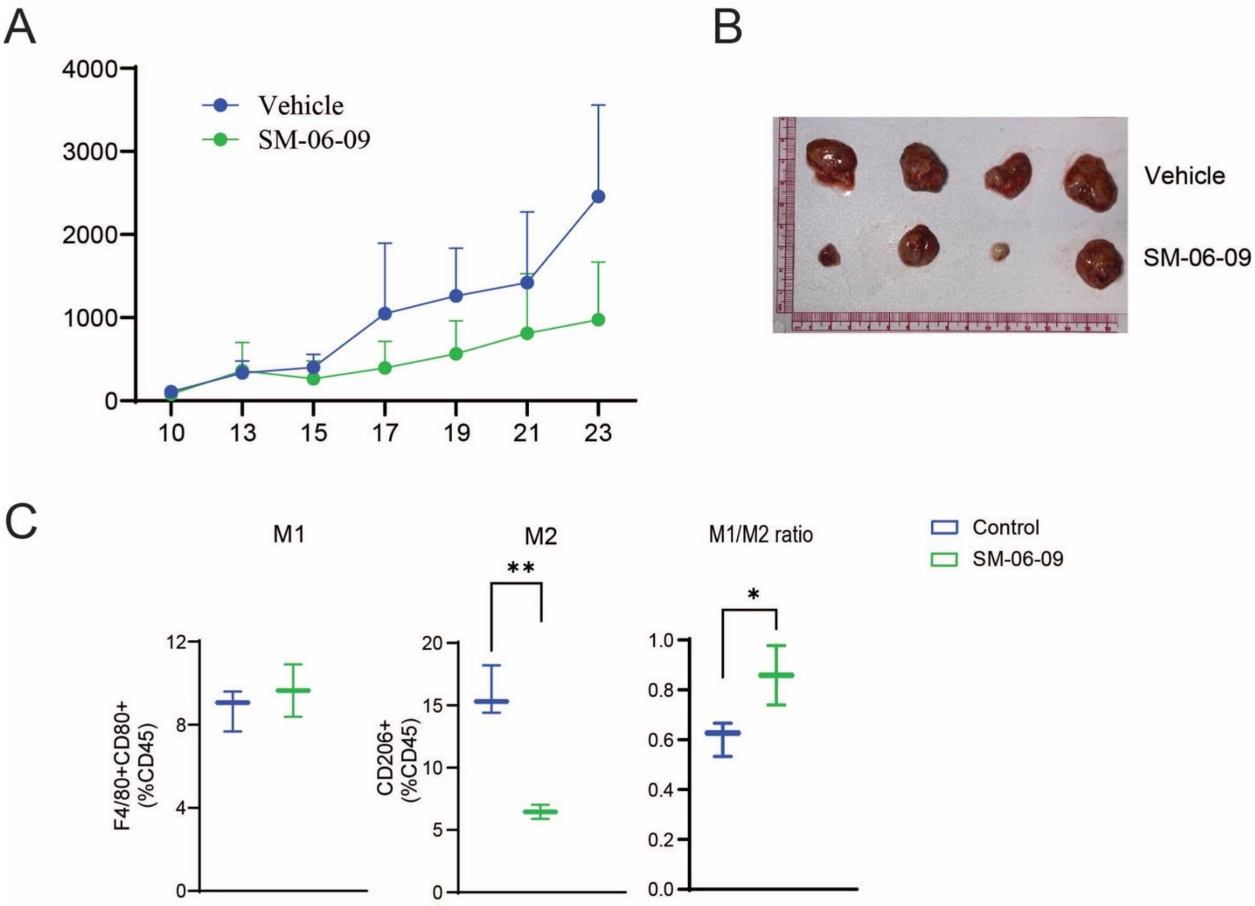

Figure S9. SM-06-09 reduces tumor growth and increases the M1/M2 macrophage ratio in the TME in the SM1 murine melanoma model. SM1 melanoma cells were implanted subcutaneously in the flank area of C57BL/6 mice and treated with vehicle or SM-06-09 (**3m**) (i.p. injections) every other day, starting when the tumors were palpable. Tumor measurements were obtained on alternate days. At the end of the study, tumors were harvested for immunotyping with flow cytometry. (**A**) Kinetics of tumor growth and (**B**) representative tumor images. (**C**) Flow cytometry characterization of tumor-associated macrophages into M1- and M2-like phenotypes. Data correspond to four mice per group; (n=4 mice); *P<0.05, **P<0.01, one-way ANOVA.

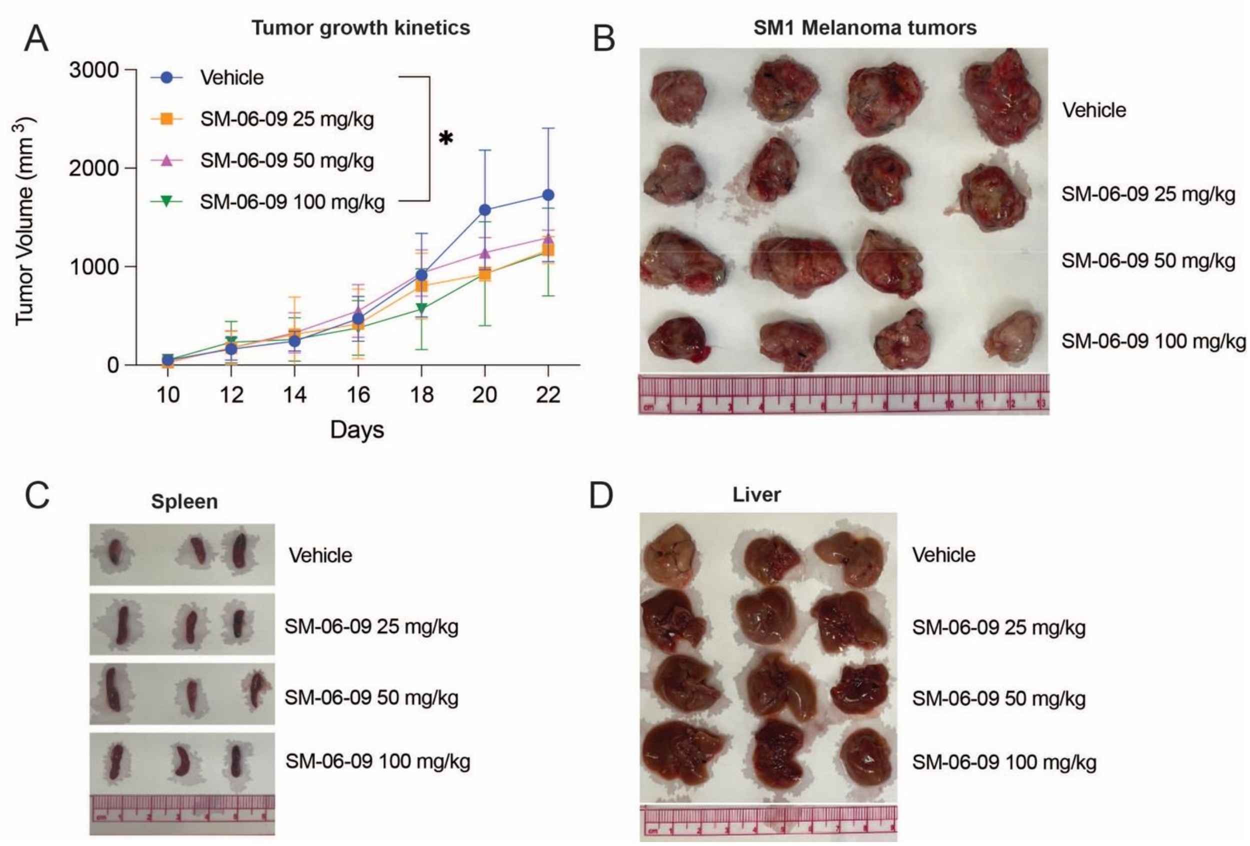

Figure S10. **Dose determination for oral administration of SM-06-09 and the potential side effects in the SM1 melanoma model.** SM1 melanoma-bearing mice received oral daily administration of vehicle or SM-06-09 (**3m**) at 25, 50, or 100mg/kg. Tumor measurements were obtained on alternate days. At the end of the study, tumors were harvested for immunotyping, and organs, including the spleen and liver, were collected to observe potential treatment-associated cytotoxicity. (**A**) Kinetics of tumor growth and (**B**) representative tumor images. The data corresponds to five to six mice per group. (n=5-6 mice); Representative images of (**C**) spleen and (**D**) liver *P<0.05, two-way ANOVA.

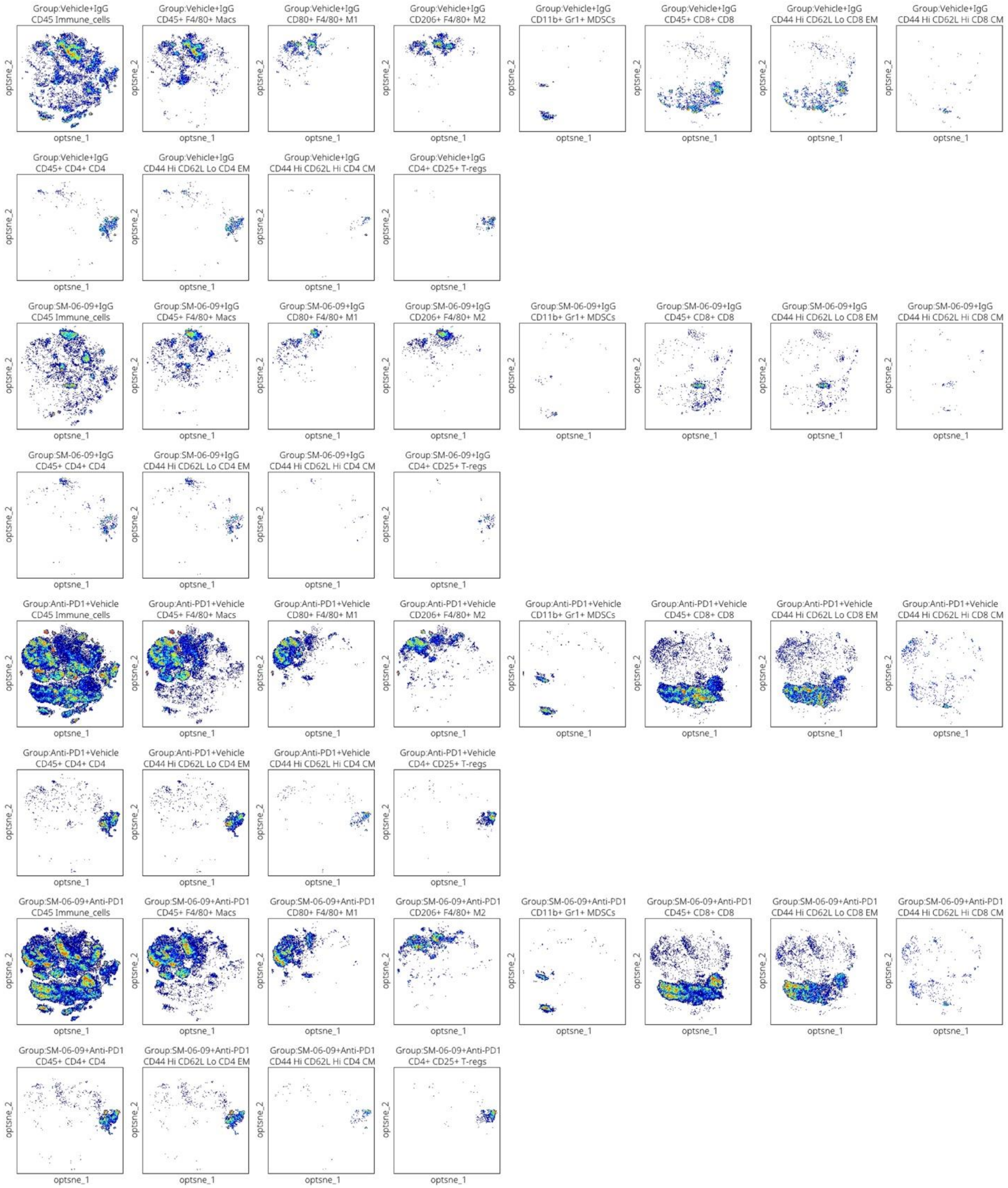

Figure S11. Feature plots representing the distribution of immune cell populations in opt-SNE. The feature plots represent immune cell populations gated as indicated for the treatment groups.

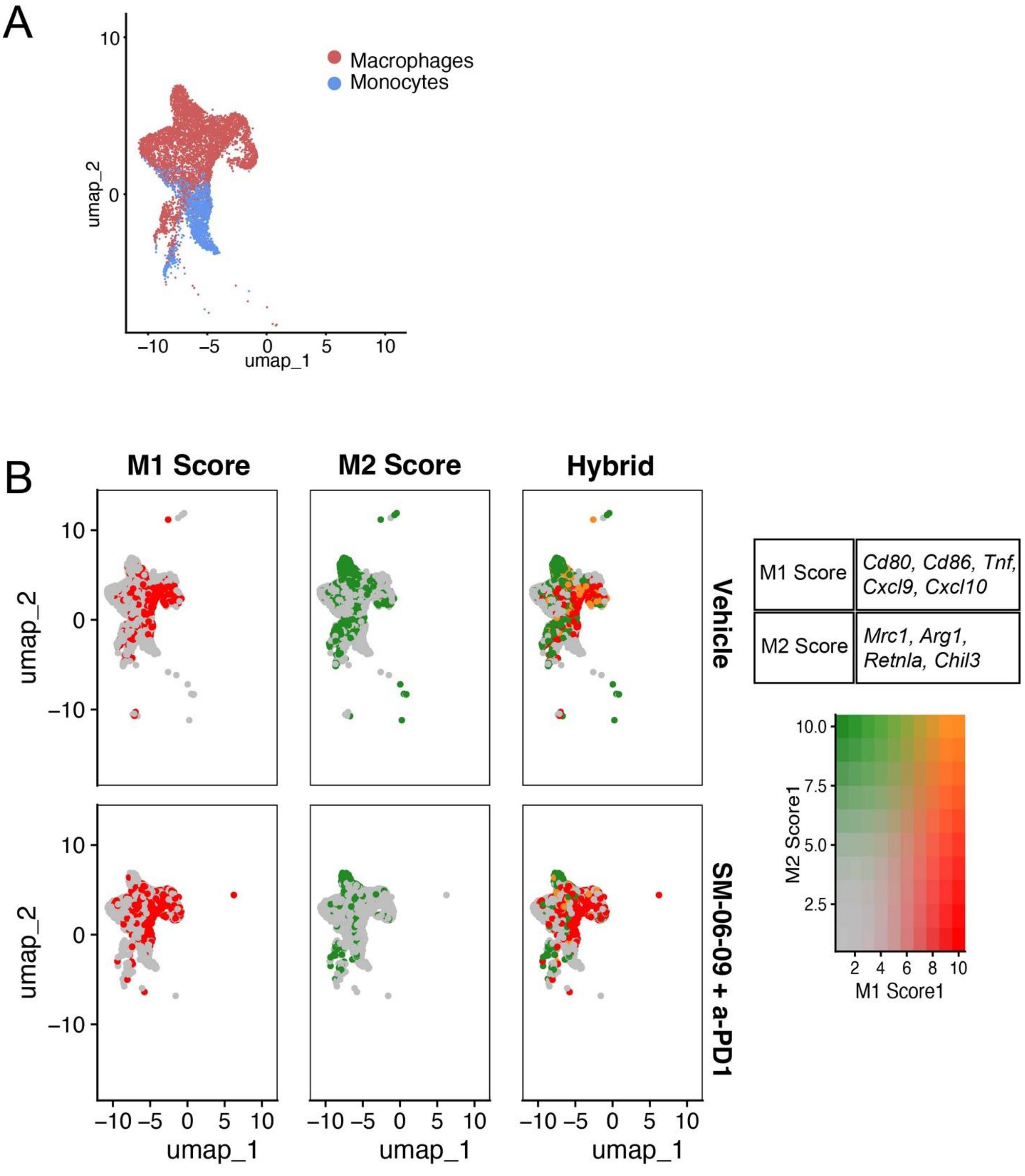

Figure S12. Distinction of monocyte and macrophage nodes for trajectory analysis. (**A**) UMAP showing subset of monocytes and macrophages, labeled using ImmGen through SingleR. (**B**) Blended FeaturePlots showing expression of distinct M1-like and M2-like scores, with a table showing the M1 and M2 marker genes used for the AddModuleScore function.

7. References

(1) Duncton, M. A. J.; Singh, R. A One-Pot Synthesis of Tetrazolones from Acid Chlorides: Understanding Functional Group Compatibility, and Application to the Late-Stage Functionalization of Marketed Drugs. *Org. Biomol. Chem.* **2016**, *14* (39), 9338–9342. https://doi.org/10.1039/c6ob01644h.

(2) Still, W. C.; Kahn, M.; Mitra, A. Rapid Chromatographic Technique for Preparative Separations with Moderate Resolution. *J. Org. Chem.* **1978**, *43* (14), 2923–2925. https://doi.org/10.1021/jo00408a041.

(3) Pedersen, D.; Rosenbohm, C. Dry Column Vacuum Chromatography. *Synthesis (Mass.)* **2004**, *2001* (16), 2431–2434. https://doi.org/10.1055/s-2001-18722.

(4) Jafarzadeh, M. Trimethylsilyl Azide (TMSN3): A Versatile Reagent in Organic Synthesis. *Synlett* **2007**, *2007* (13), 2144–2145. https://doi.org/10.1055/s-2007-984895.

(5) Zammit, S. C.; Cox, A. J.; Gow, R. M.; Zhang, Y.; Gilbert, R. E.; Krum, H.; Kelly, D. J.; Williams, S. J. Evaluation and Optimization of Antifibrotic Activity of Cinnamoyl Anthranilates. *Bioorg. Med. Chem. Lett.* **2009**, *19* (24), 7003–7006. https://doi.org/10.1016/j.bmcl.2009.09.120.

(6) Friesner, R. A.; Banks, J. L.; Murphy, R. B.; Halgren, T. A.; Klicic, J. J.; Mainz, D. T.; Repasky, M. P.; Knoll, E. H.; Shelley, M.; Perry, J. K.; Shaw, D. E.; Francis, P.; Shenkin, P. S. Glide: A New Approach for Rapid, Accurate Docking and Scoring. 1. Method and Assessment of Docking Accuracy. *J. Med. Chem.* **2004**, *47* (7), 1739–1749. https://doi.org/10.1021/jm0306430.

(7) Shen, S.; Hadley, M.; Ustinova, K.; Pavlicek, J.; Knox, T.; Noonepalle, S.; Tavares, M. T.; Zimprich, C. A.; Zhang, G.; Robers, M. B.; Bařinka, C.; Kozikowski, A. P.; Villagra, A. Discovery of a New Isoxazole-3-Hydroxamate-Based Histone Deacetylase 6 Inhibitor SS-208 with Antitumor Activity in Syngeneic Melanoma Mouse Models. *J. Med. Chem.* **2019**, *62* (18), 8557–8577. https://doi.org/10.1021/acs.jmedchem.9b00946.

(8) Sastry, G. M.; Adzhigirey, M.; Day, T.; Annabhimoju, R.; Sherman, W. Protein and Ligand Preparation: Parameters, Protocols, and Influence on Virtual Screening Enrichments. *J. Comput. Aided Mol. Des.* **2013**, *27* (3), 221–234. https://doi.org/10.1007/s10822-013-9644-8.

(9) Harder, E.; Damm, W.; Maple, J.; Wu, C.; Reboul, M.; Xiang, J. Y.; Wang, L.; Lupyan, D.; Dahlgren, M. K.; Knight, J. L.; Kaus, J. W.; Cerutti, D. S.; Krilov, G.; Jorgensen, W. L.; Abel, R.; Friesner, R. A. OPLS3: A Force Field Providing Broad Coverage of Drug-like Small Molecules and Proteins. *J. Chem. Theory Comput.* **2016**, *12* (1), 281–296. https://doi.org/10.1021/acs.jctc.5b00864.

(10) Guimarães, C. R. W.; Cardozo, M. MM-GB/SA Rescoring of Docking Poses in Structure-Based Lead Optimization. *J. Chem. Inf. Model.* **2008**, *48* (5), 958–970. https://doi.org/10.1021/ci800004w.
